# Synthetic Retinoids for the Modulation of Genomic and Nongenomic Processes in Neurodegenerative Diseases

**DOI:** 10.1021/acsomega.5c00934

**Published:** 2025-05-28

**Authors:** Abbey M. Butler, David R. Chisholm, Charles W. E. Tomlinson, Thabat Khatib, Jason Clark, Shunzhou Wan, Peter V. Coveney, Iain R. Greig, Peter McCaffery, Ehmke Pohl, Andrew Whiting

**Affiliations:** † Department of Chemistry, 3057 Durham University, South Road, Durham DH1 3LE, U.K.; ‡ Institute of Medical Sciences, 1019 University of Aberdeen, Foresterhill, Aberdeen, Scotland AB25 2ZD, U.K.; § Health Sciences Department, Faculty of Modern Sciences, 61287 Arab American University, P.O. Box 840009 Ramallah, Palestine; ∥ Centre for Computational Science, Department of Chemistry, 4919 University College London, 20 Gordon Street, London WC1H 0AJ, U.K.; ⊥ Advanced Research Computing Centre, University College London, London WC1H 0AJ, U.K.; # Institute for Informatics, Faculty of Science, University of Amsterdam, 1098XH Amsterdam, The Netherlands; ∇ Department of Biosciences, Durham University, South Road, Durham DH1 3LE, U.K.

## Abstract

Retinoids, such as all-*trans* retinoic acid (ATRA), are the active metabolite forms of endogenous Vitamin A and function as key signaling molecules involved in the regulation of a variety of cellular processes. Due to their highly diverse biological roles, retinoids have been implicated in a wide range of diseases such as neurological disorders and some cancers. However, their therapeutic potential is limited due to their chemical and metabolic instability and adverse side effects. Synthetic retinoid analogues with increased stability and specificity have therefore attracted significant attention. In this study, we developed a scalable synthetic platform to generate a library of novel synthetic retinoids. Twenty-three new compounds were synthesized, and their receptor binding was assessed by an *in vitro* fluorescence competition binding assay, complemented by molecular docking and molecular dynamics (MD) simulations. We show that while computational studies are extremely useful for predicting binding modes and hence can guide synthetic efforts, the binding assays demonstrated that these novel retinoids exhibit strong binding albeit with limited selectivity for the different retinoic acid receptors (RARs). Therefore, their biological activity was measured by assessing their genomic and nongenomic activities in neuroblastoma cells with the goal of correlating binding properties and pathway activation to neuro-regenerative potential measured by neurite outgrowth. Importantly, four of the novel retinoids are shown to bind tightly to RARs and exhibit dual action in the relevant cellular models, with an ability to induce both genomic and nongenomic responses as well as significant neurite outgrowth. The compound with the highest biological activity possesses significant potential to be used as therapeutics for treating a wide range of neurological disorders like Alzheimer’s disease and motor neuron disease.

## Graphical abstract

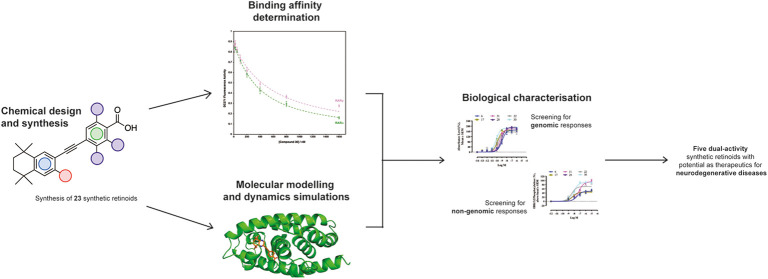

## Introduction

Endogenous retinoids are signaling molecules derived from Vitamin A that influence an enormous variety of cellular signaling pathways by controlling transcription processes in both the cell nucleus and cytoplasm.^1^ These lipophilic fatty acid small molecules, represented chiefly by all-*trans*-retinoic acid (ATRA) (Figure 1), exhibit low to subnanomolar binding affinity for a family of nuclear receptor proteins comprised of the retinoic acid receptors (RARs) and retinoid X receptors (RXRs). The binding of retinoids to the ligand-binding pocket (LBP) of these receptors initiates a conformational change in the protein structure that presents a characteristic protein-binding motif on the exterior surface of the receptor, allowing heterodimerization (RAR/RXR) or homodimerization (RXR/RXR) and the recruitment of cofactors to occur.^1^ These multiprotein complexes act as mediators of transcription processes by binding to short sequences of DNA known as retinoic acid response elements (RAREs). This DNA-bound complex initiates the transcriptional machinery in the nucleus.

**Figure 1:**
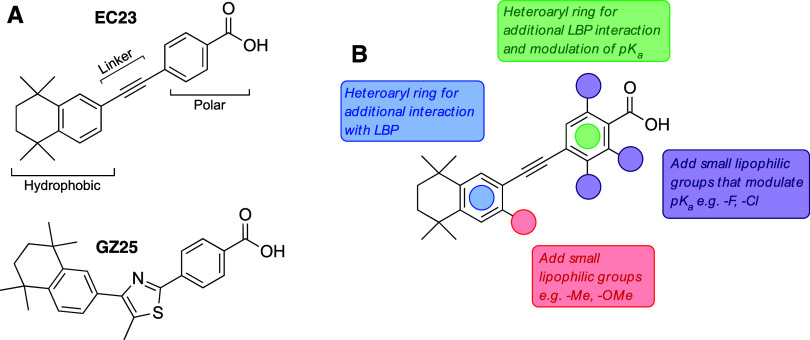
(A) Chemical structures of EC23 and GZ25; synthetic retinoids that elicit both genomic and nongenomic responses.^18^ (B) Proposed modifications to a general template structure, designed to alter, and potentially improve, RAR binding affinity.

This intricate sequence of molecular events, regulated by ATRA and its natural isomers, controls a plethora of cellular processes, including proliferation, differentiation, and homeostasis. These genomic processes are enormously complex, but recent work has shown that retinoids also act to control a range of “nongenomic” processes, including the activation of kinases such as ERK1/2. These nongenomic activities may even involve the RARs^2–4^ or be independent^5–7^ of them, adding further intricacy to the signaling pathway(s) that retinoids control. These effects manifest themselves in a variety of ways; in the brain, for example, they are likely to be important in neurite outgrowth, growth cone turning, and control of neuronal differentiation.^8^ In addition, control of translation through activation of the RARs is vital for homeostatic plasticity, regulating the insertion of AMPA receptors in postsynaptic membranes through ATRA binding to RARα and releasing the GluR1 mRNA for translation.^9^ Many of these actions have a common denominator of disruption in neurodegenerative disease and point to potential involvement of RAR signaling in such disorders.

ATRA undergoes conversion *in vivo* to a variety of isomers including 9-*cis*-retinoic acid (9cRA), which has been shown to exhibit a strong affinity for the RXR receptors^10^ with a very broad range of actions, increasing the potential for adverse effects. However, ATRA and its isomers are also notoriously capricious molecules. The extended, conjugated polyene structures isomerize readily to a mixture of isomers,^11^ and they trigger the expression of catabolic enzymes, thus significantly complicating their observed activities. Synthetic analogues of these endogenous retinoids have been designed that exhibit significantly improved stability,^12^ and furthermore, compounds that exhibit selectivity for binding to the individual isotypes of the RARs (RARα, RARβ, and RARγ) have also been developed by exploiting the subtle, yet significant differences in the active site of the ligand-binding domain (LBD) of these receptors.^12,13^ However, it remains largely unknown how these specificities impact downstream *in vitro* and *in vivo* biological activities and, in particular, how they influence potential engagement with genomic and/or nongenomic processes, and how this relates to the treatment of diseases. Retinoids have been widely utilized in the management of acute skin conditions, such as acne^14^ and psoriasis^15^ as well as in the treatment of some cancers, such as acute promyelocytic leukemia.^16^ Hence, studies that can enhance our understanding of the means to influence genomic and nongenomic processes will enable us to develop the next generation of retinoid-based therapies.

During a recent study into the ability of retinoids to promote neurite outgrowth in SH-SY5Y neuroblastoma cells, we showed that compounds that elicited both a genomic and nongenomic response were also capable of inducing robust neurite outgrowth, while those that triggered only genomic or only nongenomic pathways were significantly less effective.^11,17,18^ These compounds were thought to exhibit strong binding to each of the RARs. We hypothesized that the origins of this dual genomic and nongenomic activity may be caused by the relative contributions of binding to each RAR isoform.

We designed and synthesized an extended compound library of 23 new synthetic retinoids to rationalize these observations. Initial predictions about their binding modes and potential RAR isoform specificity were made using MD simulations and molecular docking studies. These predictions were then verified experimentally using *in vitro* fluorescence competition assays against the RAR isoforms to determine accurate binding affinities. Further to this, the genomic and nongenomic activities of the compounds were also assessed, by measuring the levels of ERK1/2 phosphorylation, and finally, their impact upon neurite outgrowth in SH-SY5Y cells to characterize the phenotypic effect of the compounds. This study aimed to rationalize the subtle structure–activity relationships at play between the RARs and retinoids. As well as this, this study aimed to identify potential new lead compounds, providing a correlative guide for future synthetic retinoid design toward new treatments for neurodegenerative diseases.

## Results and Discussion

### Design and Synthesis

Our previous studies have highlighted that diphenylacetylene (EC23) and thiazole (GZ25) compounds (Figure 1A) could act as robust initiators of neurite outgrowth through mediating both genomic and nongenomic processes in SH-SY5Y cells.^18^ Therefore, we identified these compounds as an ideal starting point for structural modification with a view to identifying key structural and conformational motifs that affect RAR binding affinity, specificity and selectivity, and how these impact downstream biological signaling and cellular development.

Through computational analysis of the ligand-binding pockets (LBP) of RARα, RARβ, and RARγ from our previous molecular docking studies,^19,20^ we identified four areas of the compound template structure (Figure 1B) that we anticipated could be modified to improve, or modulate the binding affinity for RARs. Synthetic retinoids are generally comprised of a bulky hydrophobic region, a short linker region, and a polar region substituted with a carboxylate that can interact with a cluster of polar residues at the end of the LBP (Figure 1A). We envisaged the introduction of small lipophilic substituents on the hydrophobic region could favor the binding pockets of RARβ and RARγ, while incorporating a heteroaromatic hydrophobic region could favor binding to RARα while also having beneficial effects on the overall physicochemical properties of the compounds–indeed, retinoid compounds typically exhibit poor aqueous solubility. Modulation of the p*K*_a_ of the polar region has been shown in other retinoid classes to modulate the key salt bridge interaction between retinoid carboxylic acid and an arginine residue buried deep at the bottom of the pocket, and we envisaged that heteroaromatic groups and the addition of fluorine/chlorine atoms could achieve this while also enabling potential new interactions with the narrower region around this key arginine.^19^

Accordingly, we set out to synthesize a series of analogues of EC23 ^11^ and GZ25 ^21^ that incorporated these structural modifications. We first prepared a set of 1,2,3,4-tetrahydro-1,1,4,4-tetramethylnaphthalene (TTN) synthetic building blocks (Scheme 1) that incorporated a reactive iodide (**3a**–**3c**) or alkyne (**4a**–**4c**) by initial Friedel–Crafts alkylation of benzene/toluene/anisole with dichloride **1**, followed by iodination using I_2_/H_5_IO_6_ and subsequent Sonogashira coupling with trimethylsilylacetylene and removal of the silyl protecting group.

**Scheme 1:**
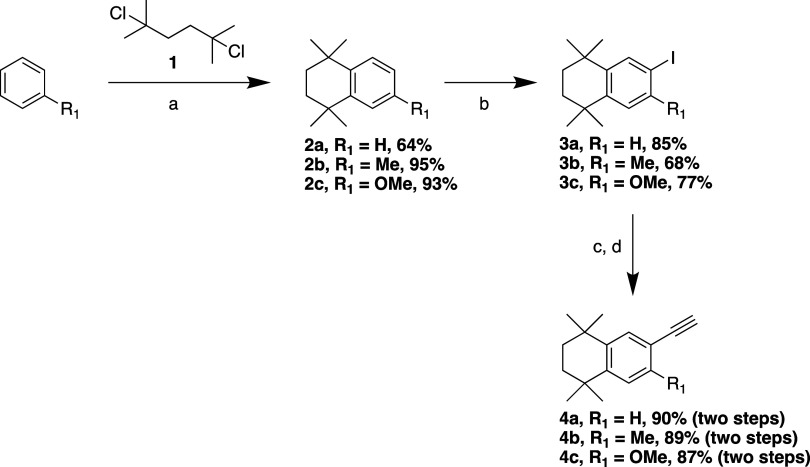
Synthesis of TTN Hydrophobic Regions^a^

a Reaction conditions: (a) AlCl_3_, DCM, RT, 5 h or 2b, AlCl_3_, toluene, RT, 3 h; (b) I_2_, H_5_IO_6_, H_2_SO_4_, AcOH, H_2_O, 70 °C, 16 h; (c) trimethylsilylacetylene, 1 mol % Pd­(PPh_3_)_2_Cl_2_/CuI, Et_3_N, RT, 16 h followed by aq NaOH, MeOH/MTBE, RT, 16 h.

We also developed a synthesis of the corresponding ethynyl-quinoxaline **10** from diester **5** (Scheme 2). This involved an initial acyloin cyclization of **5** using sodium in toluene, which was isolated as disiloxy derivative **6**, utilizing an approach described in the literature.^22^ Deprotection using bromine afforded diketone **7** which was subsequently condensed with d l-2,3-diaminopropionic acid under basic conditions to provide the intermediate quinoxaline-2-carboxylate. This was esterified under Fischer conditions in the same pot to give ester **8**.^23^ Functional group interconversion to aldehyde **9** was achieved through a facile reduction of the ester using NaBH_4_ in MeOH/THF,^24^ followed by Swern oxidation. Conversion of the aldehyde to the desired ethynyl-quinoxaline **10** proved to be intractable under a variety of Corey-Fuchs conditions,^25^ but was straightforward when the Bestmann–Ohira reagent was applied, providing **10** in a 73% yield.^26,27^

**Scheme 2:**
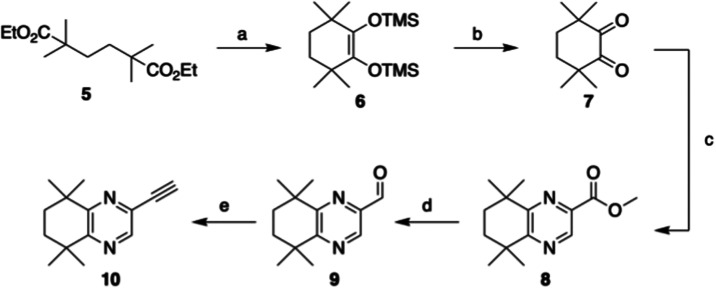
Synthesis of Ethynyl-Quinoxaline Hydrophobic Region 10^a^

a Reaction conditions: (a) Na, TMSCl, toluene, reflux, 16 h, 81%; (b) Br_2_, DCM, RT, 1 h, 78%; (c) d l-2,3-diaminopropionic acid HCl, 4 equiv NaOH, MeOH, reflux, 48 h, followed by H_2_SO_4_, reflux, 6 h, 61% (two steps); (d) NaBH_4_, MeOH/THF, reflux, 16 h, 91%, followed by (COCl)_2_, DMSO, Et_3_N, DCM, −78 °C to RT, 2 h, 86%; (e) dimethyl (1-diazo-2-oxopropyl)­phosphonate, K_2_CO_3_, MeOH, RT, 16 h, 73%.

A range of substituted polar region ester coupling partners (Scheme 3) were also prepared from the commercially available acids using typical Fischer conditions or alkylation with iodomethane.

**Scheme 3:**
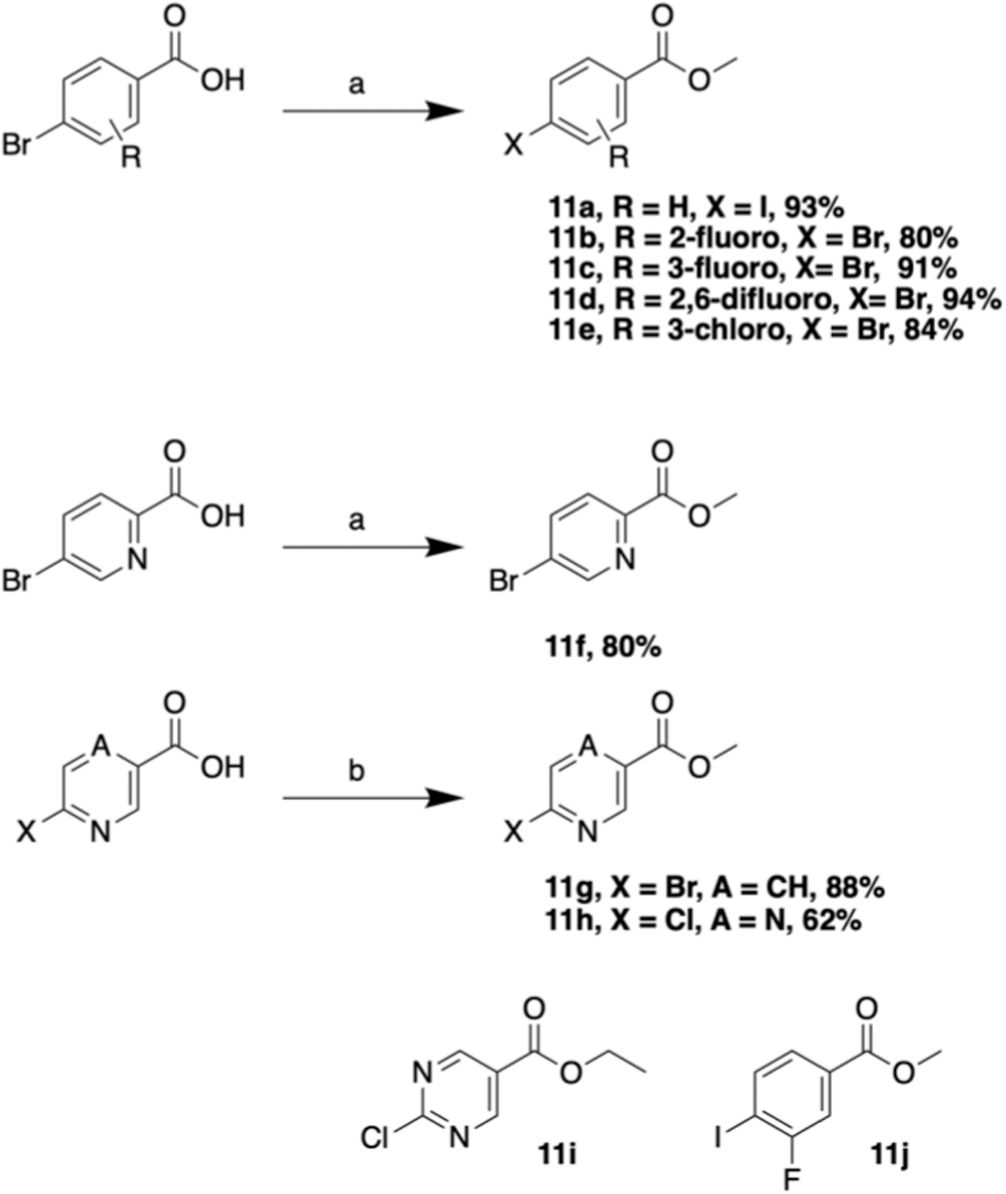
Synthesis of Polar Regions^a^

a Reaction conditions: (a) conc. H_2_SO_4_, MeOH, reflux, 16–24 h; (b) MeI, K_2_CO_3_, DMF, RT, 16 h.

With a range of complementary ethynyl and halide coupling partners in hand, we prepared a series of diphenylacetylene retinoid esters via Sonogashira coupling reactions employing the widely employed Pd­(PPh_3_)_2_Cl_2_/CuI catalyst system, generally by reacting **4a**–**c** or **10** with halo-polar regions **11a**–**g**, although occasionally between iodides **3a**–**c** and ethynyl polar regions (**12a**–**c**) when the ethynyl hydrophobic regions proved poorly reactive. We also prepared pyrimidine derivatives (**28–29**) by the reaction of **4a**–**c** with the commercially available ethyl 2-chloropyrimidine-5-carboxylate (**11i**) using a catalyst system developed by Köllhofer et al.^28^ Saponification of the isolated esters provided the desired retinoids **13–31** (Scheme 4).

**Scheme 4:**
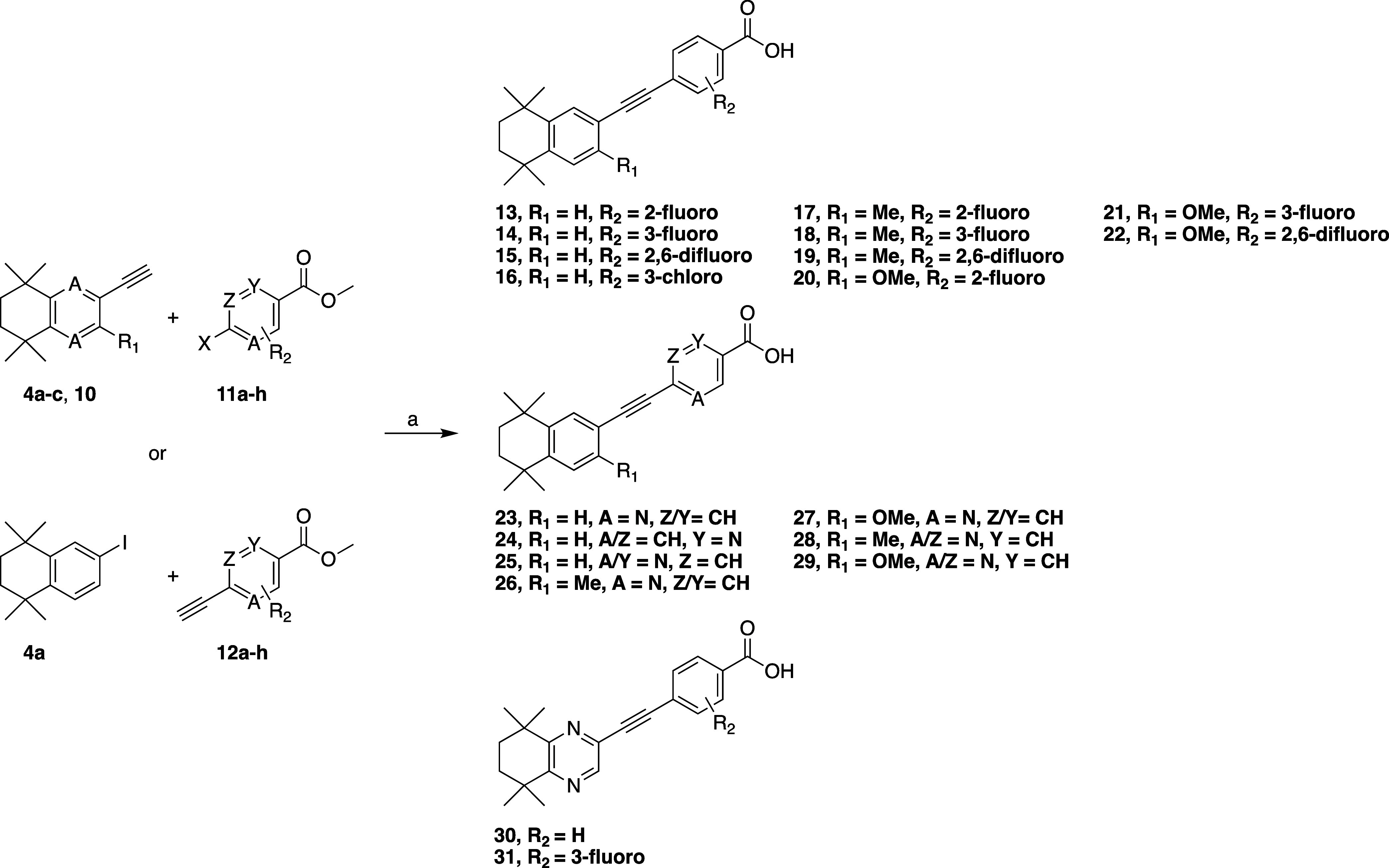
Synthesis of Diphenylacetylene Retinoids via Sonogashira Coupling Followed by Saponification^a^

a Reaction conditions: (a) 5–10 mol % Pd­(PPh_3_)_2_Cl_2_/CuI, Et_3_N or THF/Et_3_N, RT or 60 °C, 16–72 h, or for 11h 2 mol % Na_2_PdCl_4_, 4 mol % [(*t*-Bu)_3_PH]­BF_4_, 1.5 mol % CuI, 1.4 equiv Na_2_CO_3_, 100 °C, 16 h, followed by 20% aq NaOH, THF, reflux, 16–40 h, followed by 1 M HCl, RT.

In addition, we developed a series of quinoxaline analogues of GZ25, by employing the existing aldehyde intermediate **9** (Scheme 5). Grignard reaction with EtMgBr in refluxing THF provided a racemic mixture of secondary alcohol **32** in a 36% yield along with a significant amount of primary alcohol **33**, ostensibly due to ß-hydride elimination of the Grignard reagent.^29^ An array of conditions were tested in order to suppress this side reaction, including low temperature (0 to −78 °C), alternative solvents, generation of the corresponding EtMgI reagents, and several commercial solutions of EtMgBr or EtMgCl, however only addition of EtMgBr at elevated temperature afforded a reasonable amount of **32**, while lower temperature significantly increased the extent of formation of **33**. Nevertheless, **32** could be successfully oxidized under Swern conditions to give the corresponding ketone **34**, and bromination with 1.8 equiv of Cu­(II)­Br_2_ provided α-bromo-ketone **35** in an excellent yield as a key intermediate for a Hantzsch-type synthesis of the corresponding methyl thiazole.^23^

**Scheme 5:**
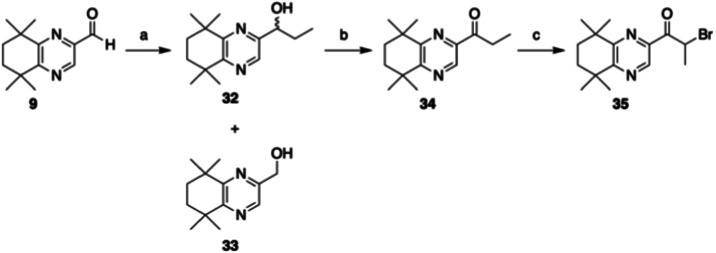
Synthesis of Quinoxaline Hantzsch Coupling Partner^a^

a Reaction conditions: (a) EtMgBr, THF, reflux, 1 h, 36%; (b) (COCl)_2_, DMSO, Et_3_N, DCM, −78 °C to RT, 2 h, 94%; (c) CuBr_2_, CHCl_3_/EtOAc, reflux, 16 h, 92%.

A variety of thioamide coupling partners **36–39** were generated (Scheme 6) via a literature method employing sodium hydrogensulfide^30^ and, with these in hand, we developed a convenient and effective Hantzsch synthesis method for the coupling of **35** with the thioamides, involving simply stirring **35** and thioamides **36**–**39** in DMF at elevated temperature for 16–24 h until conversion to the thiazole was observed. The Hantzsch reactions proceeded in good to excellent yields, and subsequent chromatographic purification and saponification provided the desired thiazole retinoids **40–43** (Scheme 7). This synthetic campaign provided 23 novel synthetic retinoids with variations around the hydrophobic, linker, and polar regions.

**Scheme 6:**
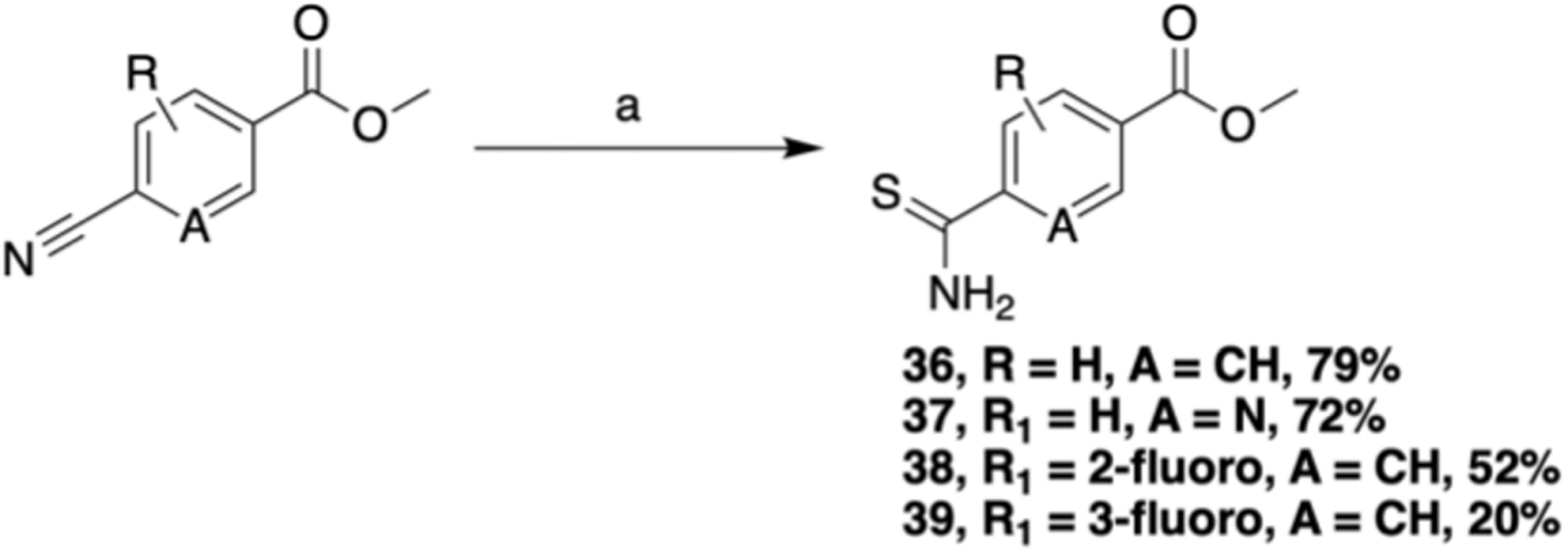
Synthesis of Thioamide Polar Regions^a^

a Reaction conditions: (a) NaHS, MgCl_2_.6H_2_O, DMF, RT, 5 h.

**Scheme 7:**
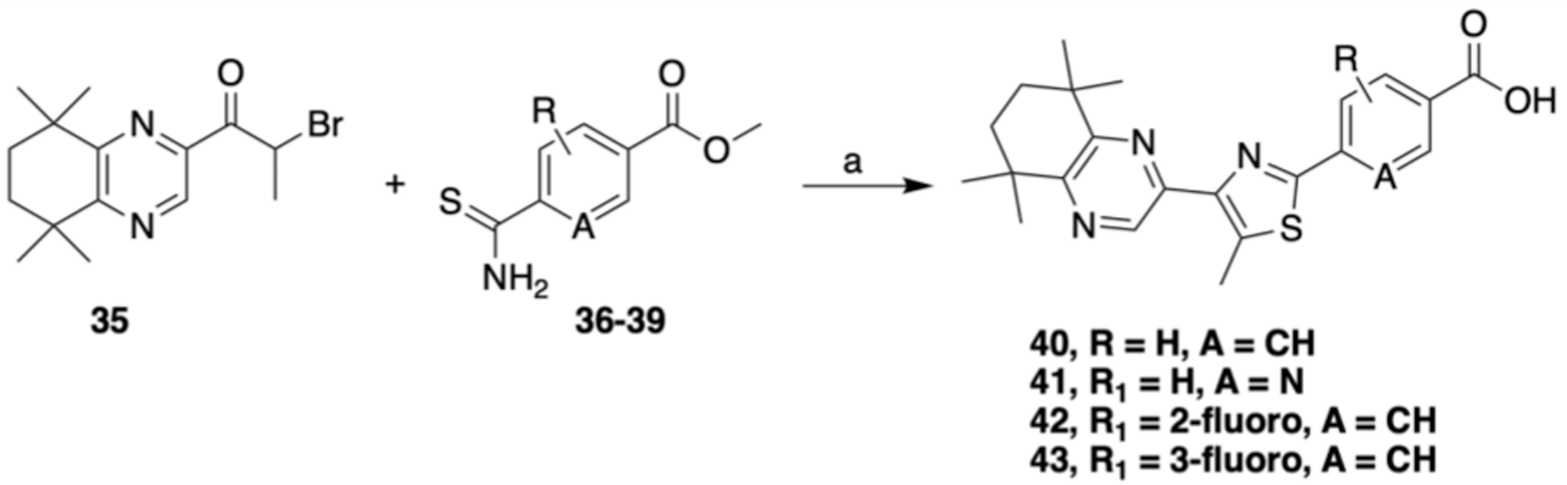
Synthesis of Thiazole Retinoids via Hantzsch Coupling Followed by Saponification^a^

a Reaction conditions: (a) DMF, 110 °C, 16–24 h, followed by 20% aq NaOH, THF, reflux, 16–40 h, followed by 1 M HCl, RT.

All 23 novel synthetic retinoids were then taken forward for biophysical characterization using *in vitro* binding assays, which were complemented by predictions about their binding specificities using molecular docking and MD simulations. The synthetic retinoids were also assessed for their genomic and nongenomic biological activities, along with their potential ability to induce neurite outgrowth in cells.

### Determination of Binding Affinity

Fluorescence competition assays were employed to determine the relative binding affinities of each synthetic retinoid toward the LBD of the RARs.^31^ The assay is based on the potential for competitive displacement of the inherently fluorescent retinoid DC271 from the LBP by a test nonfluorescent synthetic retinoid.^32^ Changes in the fluorescence were measured following the addition of a serial dilution of the test compound and the results were plotted to generate a binding curve. Using **30** as an example, the binding curves generated for both RARα and RARγ (Figure 2) showed that **30** was able to displace DC271 at low concentrations and thus bind competitively to the LBP in both RARs. Assays were performed for both RARα and RARγ. However, those for RARβ were unable to be determined as RARβ proved challenging to purify in sufficient quantity and purity required for the assay. Nonlinear least-squares regression analyses were performed to calculate binding affinities and reported as binding *K*_D_, the equilibrium dissociation constant between the RAR isoform and the test synthetic retinoid. The binding affinities for each synthetic retinoid toward RARα and RARγ are reported in Table S1 (Supporting Information (SI)).

**Figure 2:**
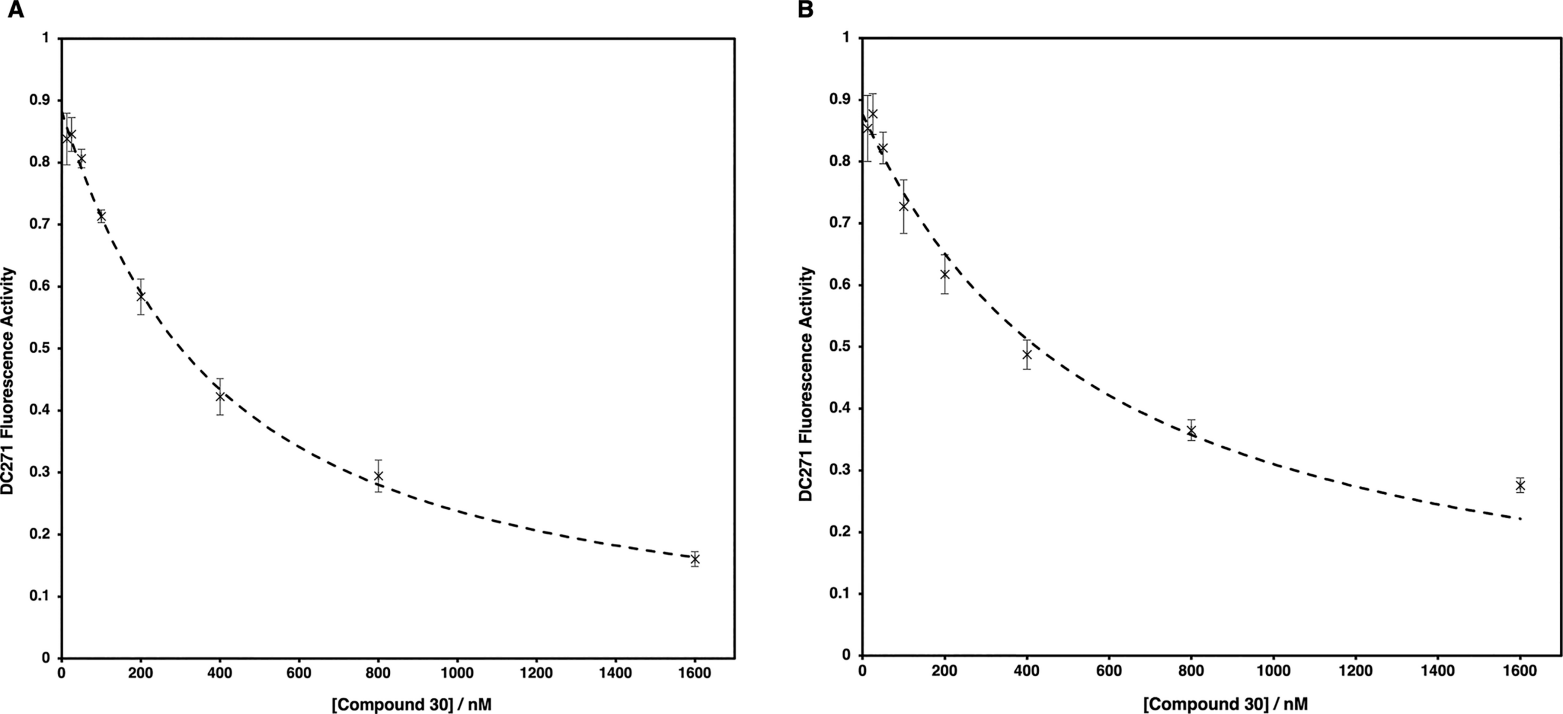
Fluorescence displacement curve of DC271 through the binding of **30** to RARα (A) or RARγ (B).

For **29**, **41** (RARα and RARγ), and **25** (RARγ), no change in fluorescence was detected which suggested that these retinoids showed noncompetitive binding with DC271. Compounds **20** and **21** were found to exhibit intrinsic fluorescence at high concentrations and, so, binding affinities were unable to be accurately calculated. A wide range of binding affinities were calculated for the remaining compounds, exhibiting low-nanomolar to very high nanomolar affinity toward RARα and RARγ. Ten of the retinoids (**13**, **14**, **16**, **17**, **18**, **19**, **23, 24**, and **26**) appeared to bind to the RARs with low-nanomolar affinity (*K*_D_ < 100 nM), similar to the binding affinities observed for EC23. **15** had the lowest binding affinity for RARα (0.37 nM) as well as a comparatively low binding affinity for RARγ (22.7 nM). Seven retinoids (**22**, **28**, **30**, **31**, **40**, **42**, and GZ25) appeared to bind to the RARs with medium to high nanomolar affinity (100 < *K*_D_ < 1000 nM), whereas **27** and **43** were bound to the RARs with very high nanomolar affinity (*K*_D_ > 1000 nM). These differences in binding affinities across the retinoids can be attributed to the subtle differences between their chemical structures, including halogenation of the carboxyl head groups and minor ring substitutions to the hydrophobic tail groups (Table 1). To understand any potential isoform specificity, the calculated binding affinities for both RARα and RARγ were plotted on a logarithmic scale (Figure 3). Nine retinoids (**13, 17, 18, 23, 27, 30, 40, 42**, and **43**) were found close to the plotted line representing equal binding affinity for RARα and RARγ. Notably, **15** strongly binds both RARα and RARγ with *K*_D_ values of 0.37 and 22.7 nM, respectively. Compounds **19**, **22**, **24, 26**, and **28** bind RARα preferentially over RARγ, whereas **14**, **16**, **31**, and GZ25 bind RARγ stronger than RARα. The differences in binding affinity values across the library of retinoids tested is small and generally in the same order of magnitude (Figure 3), which suggests that the retinoids may have low selectivity for either of the RAR isoforms *in vivo*.

**Figure 3:**
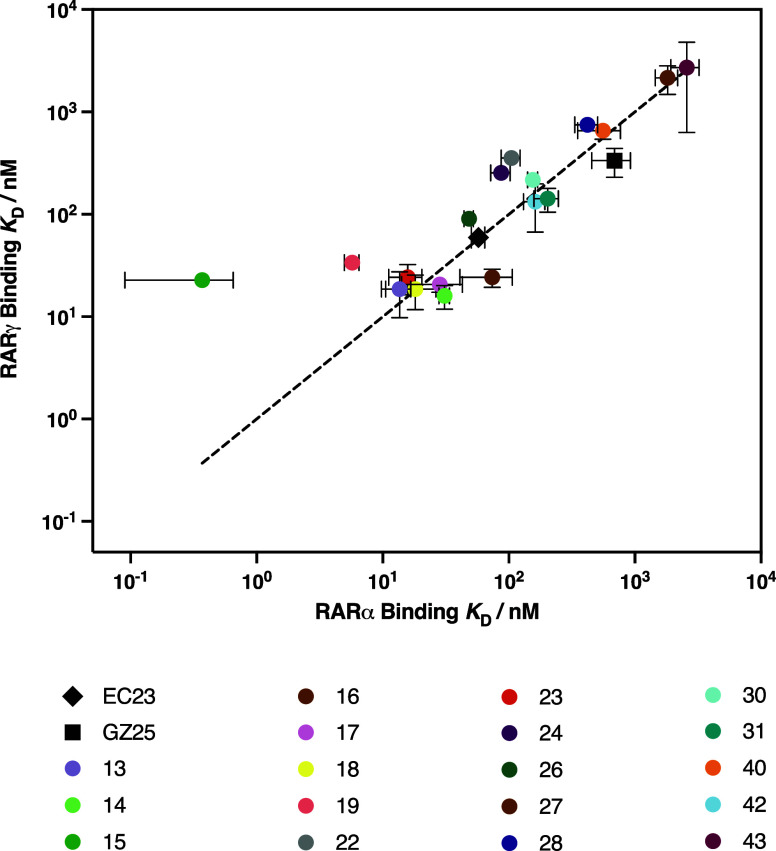
Log–log plot of binding affinities (*K*_D_) of synthetic retinoids toward RARα and RARγ. Dashed line represents an equal binding affinity toward RARα and RARγ isoforms. EC23 is shown as a black diamond, GZ25 as a black square, and the synthetic retinoids as colored circles. Error bars represent the standard deviation in K_D_ values for each synthetic retinoid and are shown for both RARα (horizontal) and RARγ (vertical).

**Table 1:**
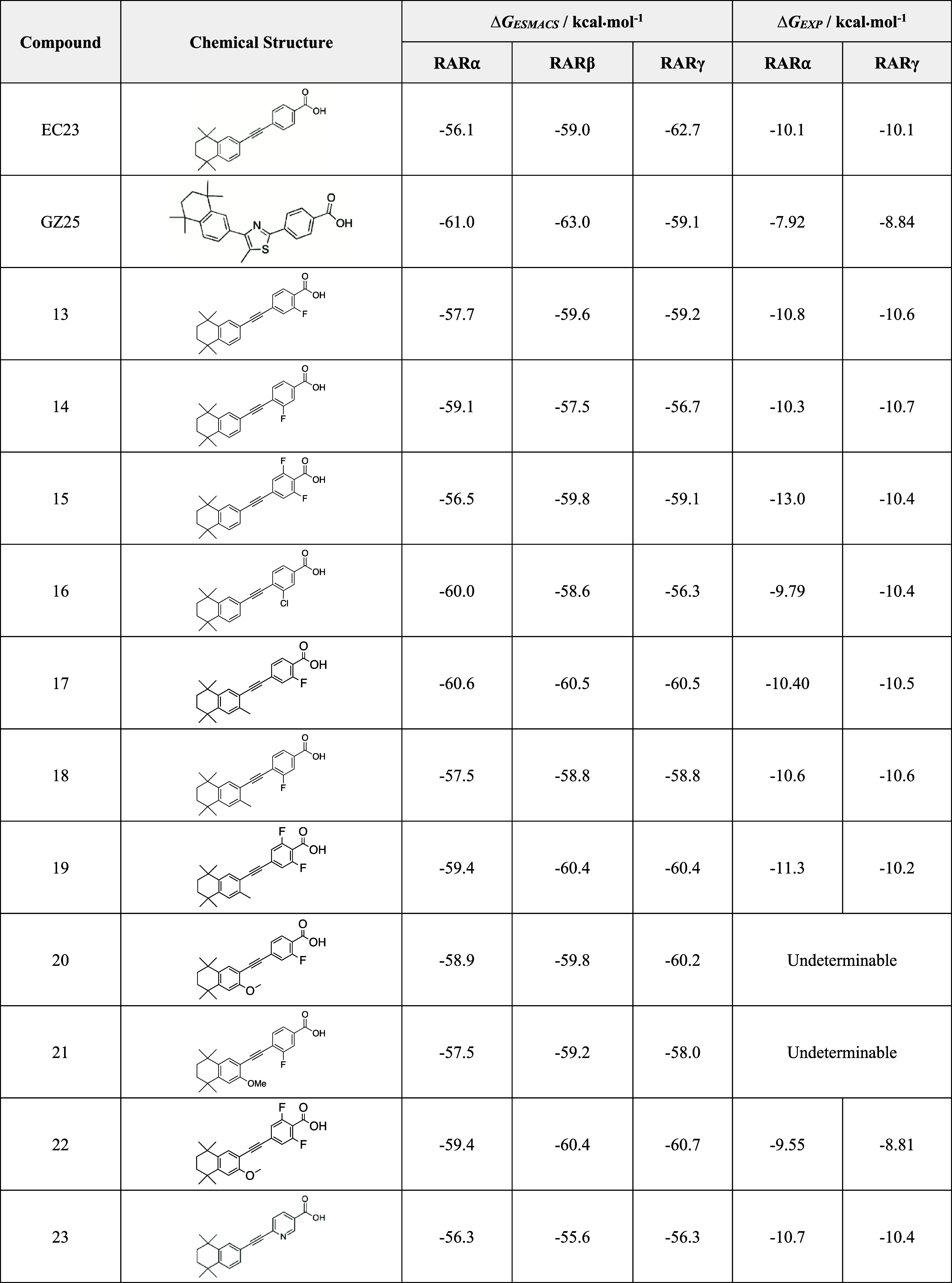

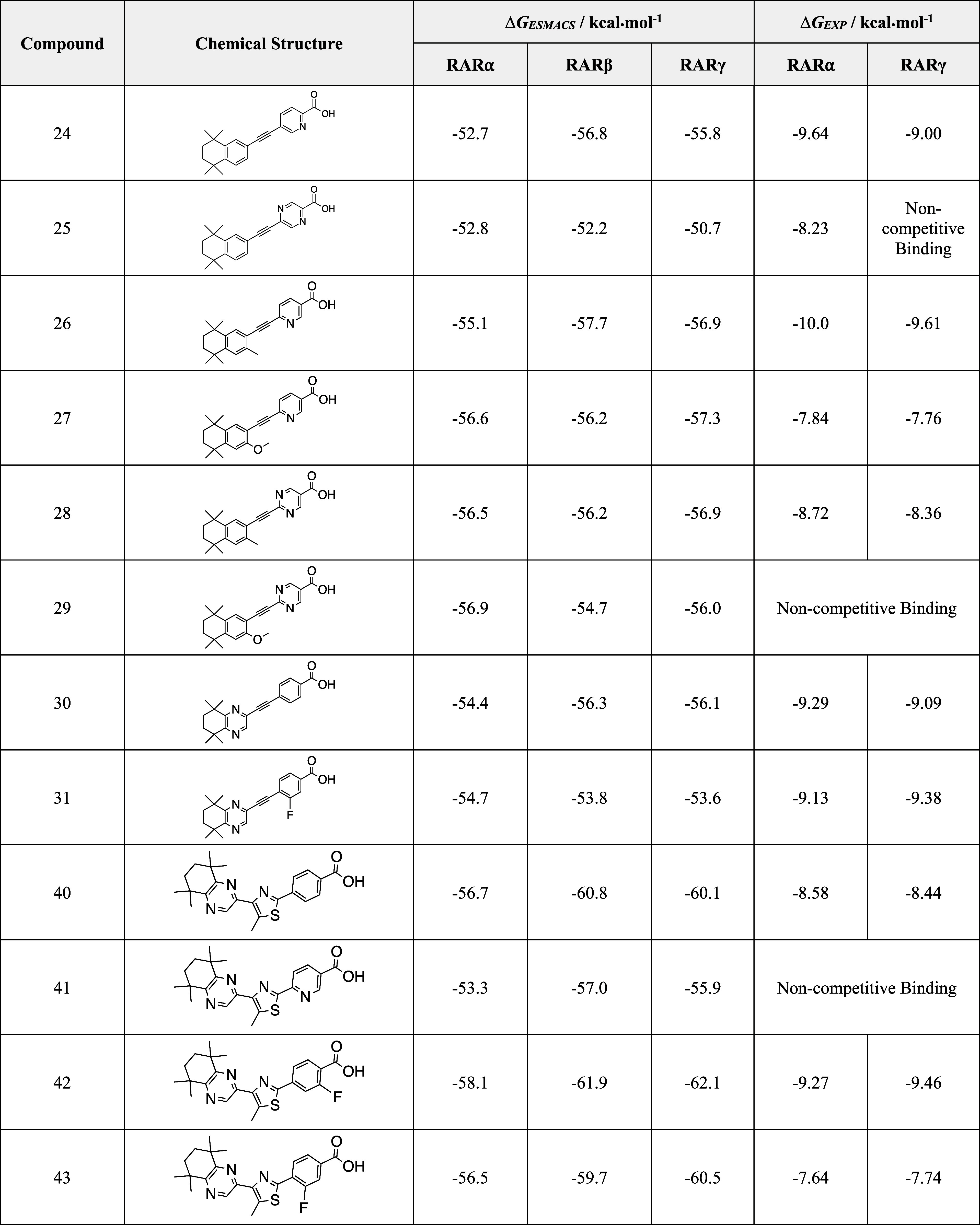
Chemical Structures; Predicted Binding Free Energies (Δ*G*_ESMACS_); and Experimental Binding Free Energies (Δ*G*_EXP_) for the Synthetic Retinoids towards RARα, RARβ, and RARγ^a^

a All values are reported to three significant figures. Noncompetitive binding refers to no detectable change in fluorescence of DC271. Binding affinities for compounds **20** and **21** were undeterminable due to intrinsic fluorescence at high concentrations.

### Prediction of Binding Specificity Using Computational Studies

Alongside the binding affinity assays, the potential binding specificities of the synthetic retinoids were predicted using both molecular docking and MD simulations. First, using GOLD,^33^ each synthetic retinoid was docked into a subset of RAR crystal structures that were obtained from the RCSB Protein Data Bank (PDB). The docking solutions for each synthetic retinoid were ranked by ChemScore and reported as a scale, where higher scores correlate to the increased likelihood of the ligand-protein docking pose. The scoring function involves the summation of all hydrogen bonding donor–acceptor pairs between the protein and ligand, considering any hydrophobic interactions, metal interactions, and potential ligand flexibility. ChemScores for the highest-ranked solution for each synthetic retinoid were reported for RARα, RARβ, and RARγ respectively (Tables S2–S4, SI). The three highest-ranking docking scores for each RAR isoform were then taken forward for MD simulations. This enables the estimation of the binding free energy (Δ*G*_ESMACS_) between each synthetic retinoid and RARα, RARβ, or RARγ using Enhanced Sampling of Molecular Dynamics with Approximation of Continuum Solvent (ESMACS) protocols.^34^ ESMACS protocols use ensemble MD, as described in Experimental Section, to produce reliable estimations of the binding free energies for each of the RAR-retinoid complexes (Tables 1 and S2–S4, SI). ChemScores for the most likely protein–ligand docking poses between all three RAR isoforms, as well as across the synthetic retinoids, were found to be similar. Most of the synthetic retinoids had a lower score compared to EC23, with the largest differences observed for **29** (RARα and RARβ) and **43** (RARγ). This is also observed in the ESMACS binding free energy estimations, with the largest differences in binding free energy for GZ25 (RARα) and **25** (RARβ and RARγ) in comparison to EC23. These results suggest that the synthetic retinoids can bind favorably to all the RAR isoforms and, since the predicted free energy values were close to that for EC23, they appear to use a similar binding mode. When ChemScores and binding free energies were expressed as ratios of RARβ:RARα (Figure 4A,B) or RARβ:RARγ (Figure 4C–D), or RARα:RARγ (Figure 4E,F), the synthetic retinoids showed no overall preferential binding toward RARα over RARβ. This was reflected by the small differences in ratio values. This was also reflected in the binding free energy ratios for RARβ:RARα. The ChemScore ratios calculated for RARβ:RARγ and RARα:RARγ suggested that most of the synthetic retinoids exhibited preferential binding to RARγ or RARα, respectively. However, when the binding free energy ratios were considered for these isoforms, the results suggested that there is no significant preferential binding toward either RARβ or RARγ, or RARα or RARγ. These findings can be exemplified using compound **30** as the binding free energies were comparable for RARα, RARβ, and RARγ. This suggested that **30** could bind to all the RAR isoforms in a near-identical manner. However, the binding free energies for RARβ and RARγ were closer in value compared to RARα. These results suggested that there may be subtle differences in the precise interactions of **30** with the LBP of RARα in comparison to RARβ and RARγ.

**Figure 4:**
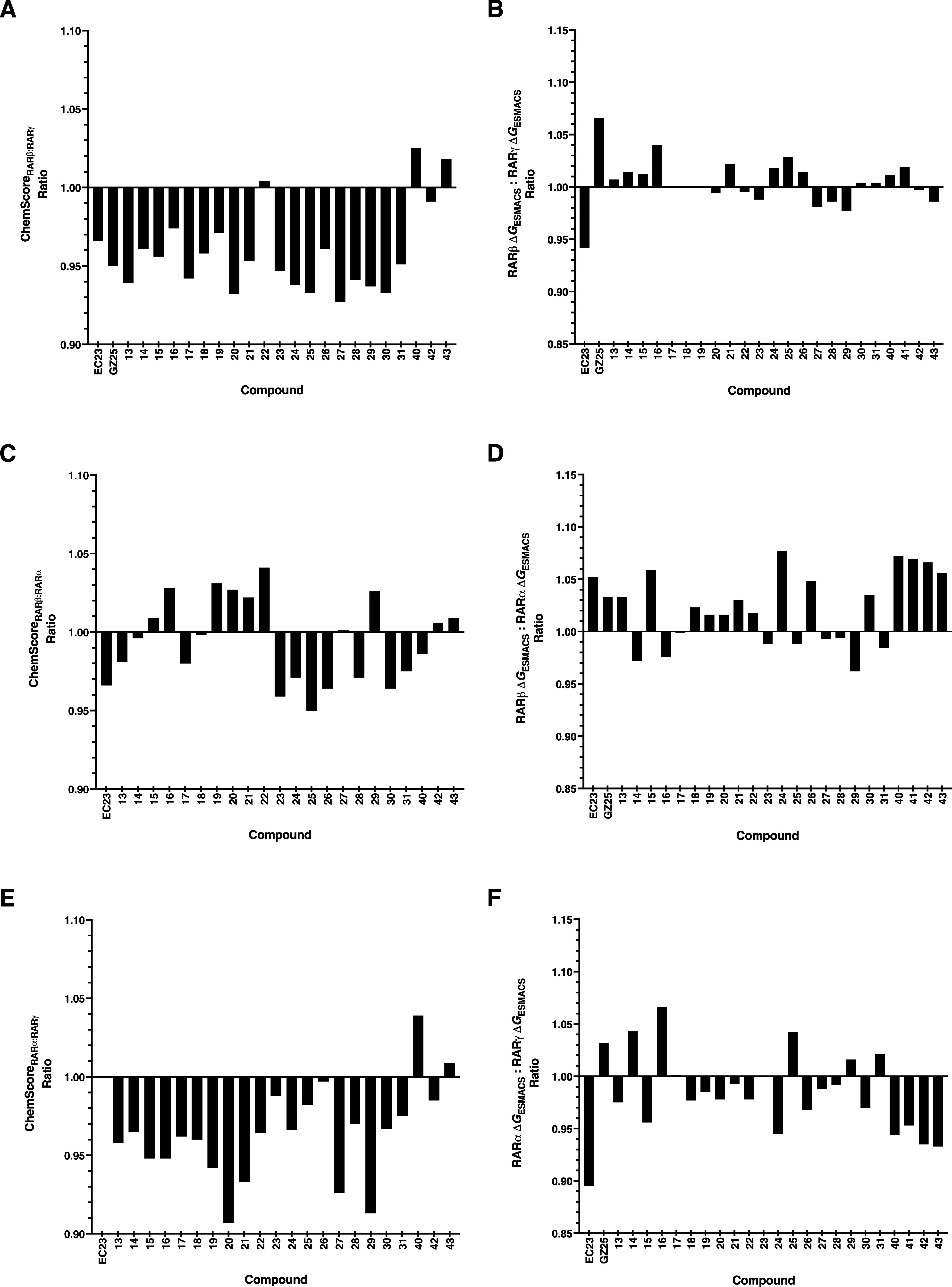
ChemScore ratios (A, C, and E) and binding free energy (Δ*G*_ESMACS_) (B, D, and F) ratios for synthetic retinoids toward (RARβ/RARα, RARβ/RARγ, and RARα/RARγ).

Binding affinities determined from the fluorescence competition assay for RARα and RARγ were expressed as binding free energies (Δ*G*_EXP_) (Table 1). These values were compared to the predicted binding free energies (Δ*G*_ESMACS_) from the MD simulations to better understand the correlations between the two methods (Figure 5). The binding free energies determined for all of the synthetic retinoids were similar for both RARα and RARγ. This was also reflected in the predicted binding free energies. However, the Δ*G*_ESMACS_ values were consistently more negative than Δ*G*_EXP_ values across the synthetic retinoid library, with differences of approximately 43.2 to 50.9 kcal/mol (RARα) and 44.7 to 52.5 kcal/mol (RARγ).

**Figure 5:**
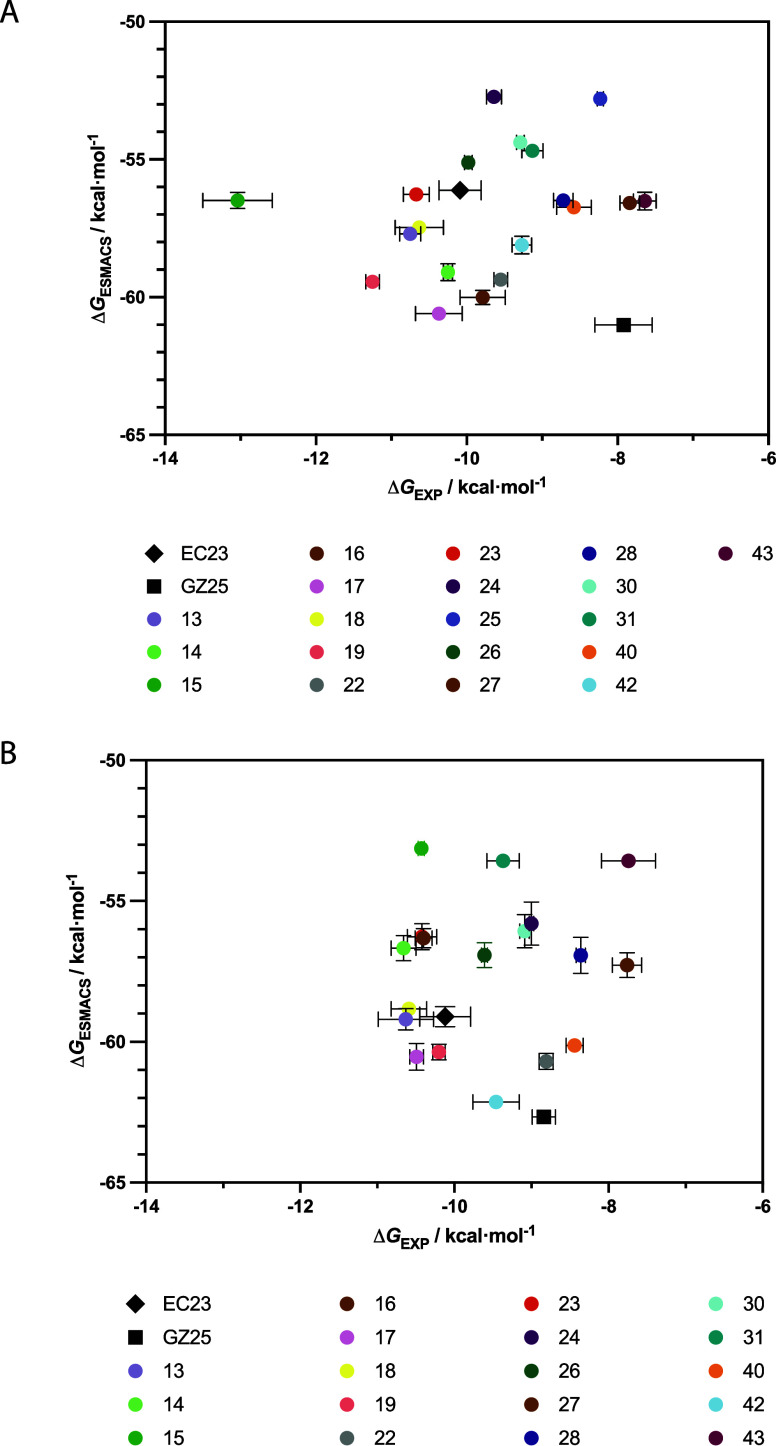
Comparison of experimental (Δ*G*_EXP_) and predicted free energies (Δ*G*_ESMACS_) for synthetic retinoids for RARα (A) and RARγ (B). EC23 is shown as a black diamond, GZ25 as a black square, and the synthetic retinoids as colored circles. Error bars represent the standard deviation in binding free energies for each synthetic retinoid and are shown for both ΔG_EXP_ (horizontal) and ΔG_ESMACS_ (vertical).

The predicted ligand-bound structures generated from both molecular docking and MD simulations for all the synthetic retinoids were analyzed and visualized using the molecular graphics software, PyMOL. Analysis of the predicted ligand-bound structure of **30** shows that the retinoid fully engages with the LBP, formed from helices α3, α5, and α7 (Figure S1, SI), of the LBD in RARα (Figure 6A,B), RARβ and RARγ. This is analogous to other retinoic acid–based analogue-RAR complexes that have been previously published in the PDB.^35–37^

**Figure 6:**
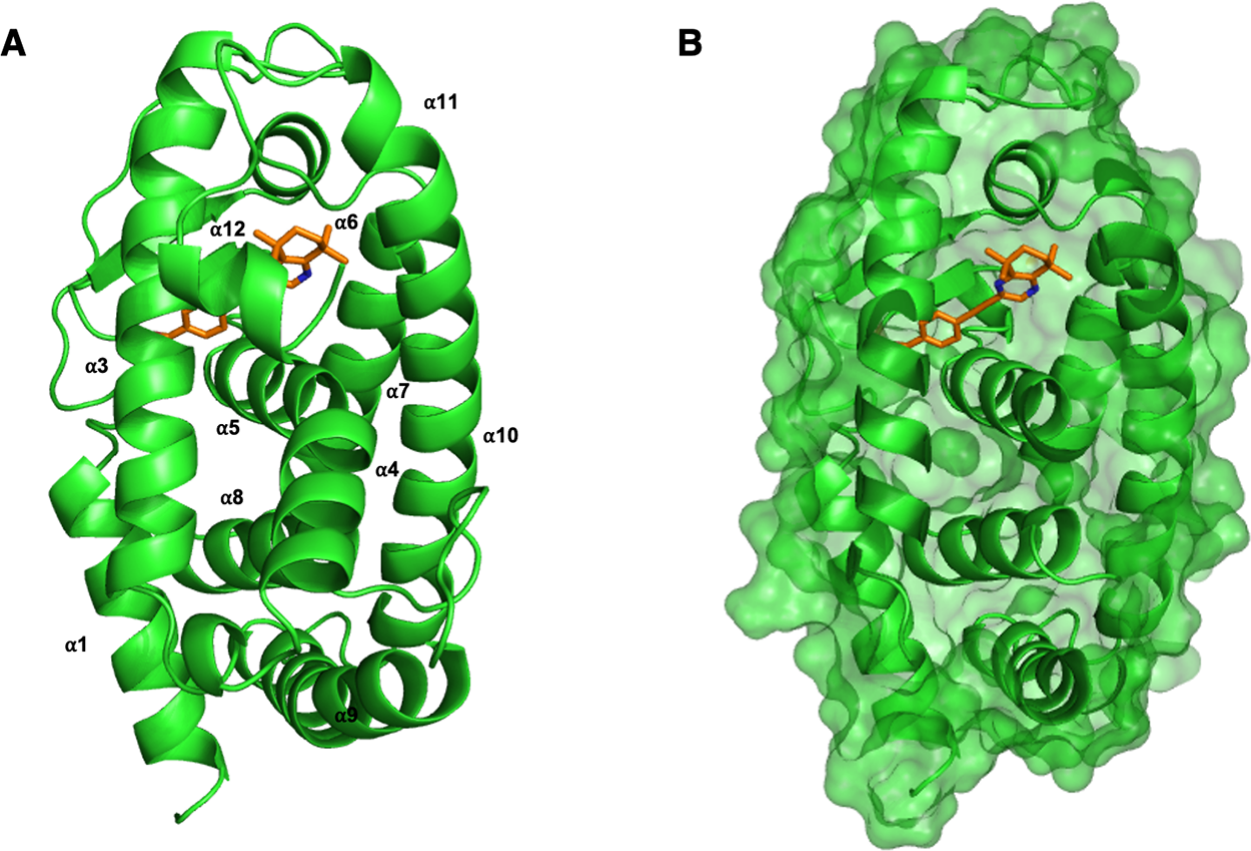
Overall view of the modeled ligand-bound structure from the MD simulation of RARα with **30** in the expected orientation (A) or in the optimal orientation to see the binding into LBP of RARα (B). RARα is shown in both cartoon and surface representation (green) and **30** is shown in stick representation (orange). α-Helices labeled, with key binding residues shown in stick representation and hydrogen bonds as dashed, black lines.

The predicted structures show that **30** is orientated into the LBP of RARα, as expected, in the canonical orientation with the polar carboxylate tail forming a hydrogen bond to R276 (Figure 7). However, there are some subtle differences in the distances between **30** and the ligand-stabilizing residues, as well as slight differences in the angles of residue side chains. This is particularly noticeable for the side chains of R276 and S232. Either alongside or in the absence of experimentally determined values for binding affinity, it is clear that molecular docking and MD simulations were both useful in predicting potential interactions of synthetic retinoids toward the LBP of the RARs. Molecular docking can be used to qualitatively predict the binding potential of the retinoid in the first instance, whereas MD simulations optimize the exact position of the ligand to achieve more chemically favorable distances between the ligand and the residues that form the LBP. These findings of the computational studies also support the prediction that the binding mode of the retinoids to the LBP does not significantly change across the synthetic retinoid library.

**Figure 7:**
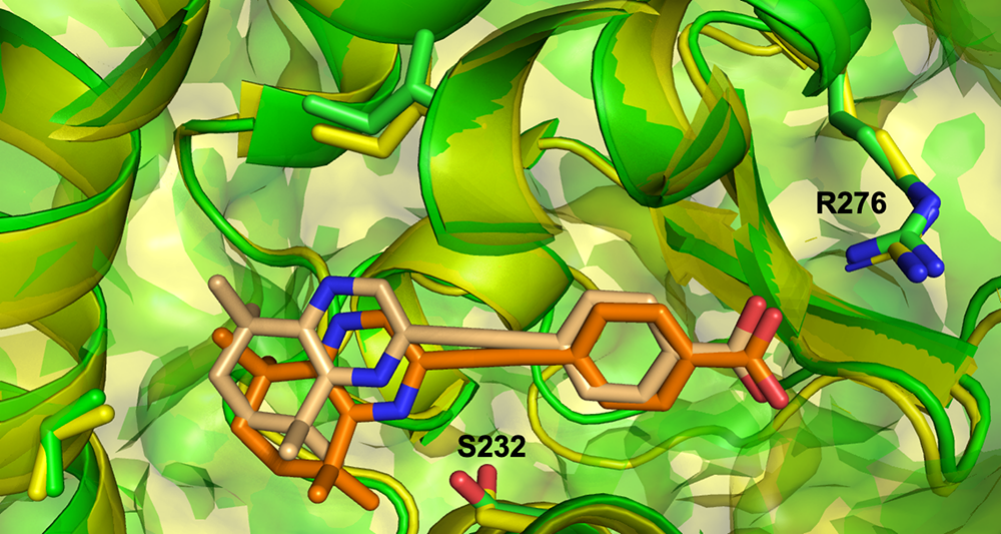
Close-up view of the modeled ligand-bound structure of RARα with **30** from MD simulation aligned with the ligand-bound structure predicted from molecular docking. Key binding residues are show in stick representation. RARα is shown in both cartoon and surface representation (green, MD simulation; yellow, molecular docking), and **30** is shown in stick representation (orange, MD simulation; peach, molecular docking).

In addition, the ligand-bound structure predictions for RARβ and RARγ show that **30** binds to the LBP of both isoforms comparably to RARα (Figure 8A–C). The polar carboxylate is orientated toward the far end of the pocket, forming a hydrogen bond to R276 (RARβ) or R278 (RARγ), found within the α5 helix. As expected, the hydrophobic quinoxaline-based headgroup of **30** is predominantly stabilized in the LBP of all three isoforms through hydrophobic interactions. Although there are some key binding residues that are involved in this region of the LBP that are isoform-specific. In RARβ, stabilization of **30** is mediated through interactions with A232, I270, and V395. This appears to be similar in RARγ, where A234 (analogous to RARβ^A232^) M272 (RARγ-specific), and A397 (RARγ-specific). However, in RARα, in addition to the hydrophobic interactions of I270 and V395, S232 (RARα-specific) can form an additional hydrogen bond to the quinoxaline ring, which further stabilizes **30** inside the LBP. Therefore, the subtle differences in residues between RARα, RARβ, and RARγ may contribute to the small differences that were observed in the calculated binding affinities and binding free energy estimations.

**Figure 8:**
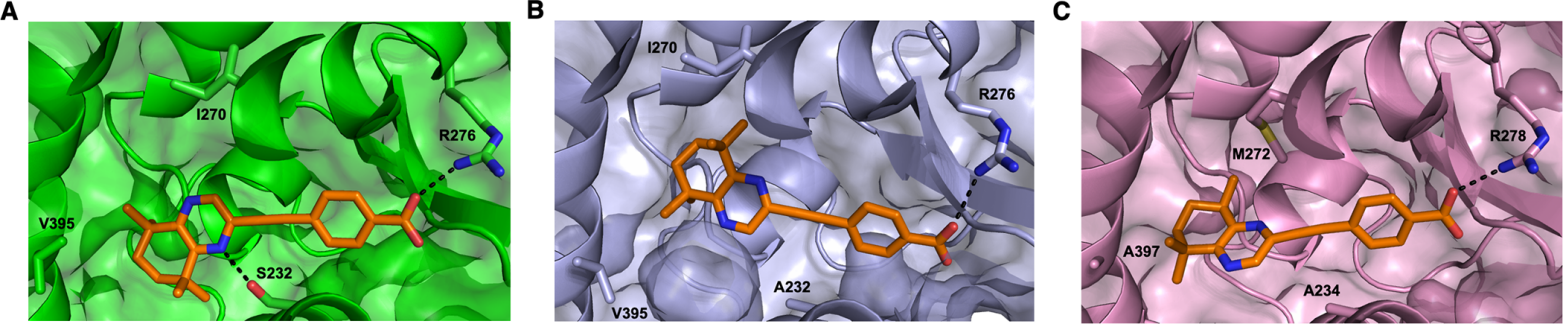
Close-up views of the modeled ligand-bound structures of RARα, RARβ, and RARγ with **30** (orange, sticks) from MD simulations. RARα (A), RARβ (C), and RARγ (C) are shown in both cartoon and surface representation (green, lilac, and pink, respectively) and **30** is shown in stick representation (orange). Isoform-specific residues are shown in stick representation and hydrogen bonds as dashed, black line.

### Biological Characterization

Following chemical design and synthesis, the synthetic retinoid library was screened for genomic and nongenomic biological activities and compared to the response of the endogenous retinoid, ATRA. In these assays, the EC_50_ value represented the concentration of retinoid that gave half-maximal response (potency), while the *E*_max_ value represented the maximum response achievable from the retinoid (efficacy). The potency and efficacy of each ligand along with the 95% confidence intervals (CI) in inducing genomic activity are summarized in Table 2.

**Table 2:** Calculated EC_50_ and *E*_max_ Values Following the Induction of Genomic and Nongenomic Response, as well as Fold Increase in Induced Neurite Outgrowth, by Synthetic Retinoids^a^

	genomic response		nongenomic response		
compound	EC_50_ (±95% CI) (nM)	*E*_max_ (±95% CI)	EC_50_ (±95% CI) (nM)	*E*_max_ (±95% CI)	fold increase in neurite outgrowth
ATRA	1.04 (±0.451)	170 (±5.65)	33.1 (±10.5)	48.6 (±1.85)	0.00
EC23	0.128 (±0.0616)	169 (±10.8)	30.6 (±14.5)	92.5 (±6.96)	2.20
GZ25	0.184 (±0.0533)	209 (±15.5)	3.57 (±0.825)	76.6 (±2.61)	1.80
13	0.210 ± (0.0724)	190 (±8.2)	2.44 × 10^–9^ (±2.29 × 10^–9^)	43.4 (±1.81)	1.90
14	0.0581 (±0.0543)	149 (±14.9)	2.74 × 10^–8^ (±2.52 × 10^–8^)	50.7 (±2.51)	2.10
15	0.0150 ± (0.00339)	185 (±4.9)	1.25 × 10^–7^ (±9.88 × 10^–8^)	76.2 (±5.47)	1.70
16	13.6 (±6.22)	97.2 (±7.9)	9.08 × 10^–8^ (±1.09 × 10^–7^)	36.9 (±2.77)	1.60
17	0.134 (±0.0519)	170 (±9.35)	5.73 × 10^–9^ (±7.24 × 10^–9^)	44.1 (±2.69)	0.00
18	1.38 (±0.743)	140 (±12.1)	1.31 × 10^–8^ (±1.34 × 10^–8^)	40.1 (±1.86)	0.00
19	0.27 (±0.0734)	171 ± (6.10)	1.44 × 10^–7^ (±1.16 × 10^–7^)	77.6 (±2.92)	1.60
20	undeterminable		undeterminable		undeterminable
21	5.31 (±1.53)	177 ± (9.10)	9.08 × 10^–8^ (±1.09 × 10^–7^)	36.9 (±2.86)	0.00
22	0.146 (±0.0809)	155 (±10.6)	3.37 × 10^–9^ (±4.34 × 10^–9^)	71.2 (±3.9)	0.00
23	0.00348 ± (0.00120)	210 (±8.45)	2.39 × 10^–8^ (±5.05 × 10^–8^)	85.7 (±9.23)	1.90
24	0.146 (±0.133)	207 (±19.5)	1.36 × 10^–8^ (±1.51 × 10^–8^)	59.7 (±3.34)	2.20
25	3.98 (±1.09)	174 (±8.25)	2.07 × 10^–8^ (±3.06 × 10^–8^)	27.9 (±1.66)	0.00
26	0.427 (±0.104)	177 (±6.65)	4.59 × 10^–7^ (±6.74 × 10^–7^)	56.4 (±6.36)	no response
27	3.16 (±0.892)	176 (±8.55)	no response		1.70
28	0.826 (±0.372)	192 (±12.7)	5.42 × 10^–9^ (±8.10 × 10^–9^)	44.7 (±3.15)	0.00
29	603 (±306)	266 (±110)	8.74 × 10^–8^ (±2.59 × 10^–9^)	37.5 (±2.12)	0.00
30	0.697 ± (0.0170)	186 ± (5.70)	8.54 (±2.02)	91.6 (±3.21)	2.30
31	0.382 (±0.0907)	181 ± (6.60)	5.84 × 10^–8^ (±5.35 × 10^–8^)	91.6 (±3.84)	0.00
40	0.00232 (±0.000751)	181 (±7.45)	0.0532 (±0.0474)	40.7 (±1.02)	1.90
41	16.9 (±16.8)	144 (±16.5)	15.4 (±0.00)	55.26 (±0.00)	1.70
42	1.55 × 10^–11^ (±6.30 × 10^–12^)	188 ± (8.70)	12.5 (±6.39)	54.1 (±4.07)	1.70
43	1.64 (±1.53)	195 (±35.2)	no response		1.80

a Genomic and nongenomic responses for compounds **20** were undeterminable, and there was no measurable nongenomic response for compounds **27** and **43**. Fold increase in neurite outgrowth relates to the measurable neurite outgrowth in comparison to nontreated cells following administration of 10 nM compound. All values are reported to three significant figures.

The transcriptional genomic activity of the retinoids was quantified using Sil-15 cells containing a RARE driving a lacZ reporter.^38^ Cells were treated with retinoids with concentrations ranging from 10–6 to 10–14 M, and EC_50_ and *E*_max_ values were calculated (Figure 9). Twenty-two of the synthetic retinoids were effective in inducing genomic activity. Fifteen of them (EC23, GZ25, **13**–**15**, **17**, **19**, **22**–**24**, **26**, **30**, **31**, **40**, and **42**) had significantly lower EC_50_ than ATRA. **18**, **28**, and **43** had similar potencies to ATRA; while the remaining six retinoids (**16**, **21**, **25**, **27**, **29**, and **41**) had significantly higher EC_50_ values than ATRA. In addition, the retinoids varied in their efficacies. Twelve retinoids (**13**, **15**, **23**, **24**, **28–31**, **40**, **42**, **43**, and GZ25) had significantly higher *E*_max_ than ATRA, five retinoids (**14**, **16**, **18**, **22**, and **41**) had significantly lower *E*_max_ than ATRA, and the rest exhibited the same potency as ATRA. The remaining five retinoids were significantly less potent compared to ATRA.

**Figure 9:**
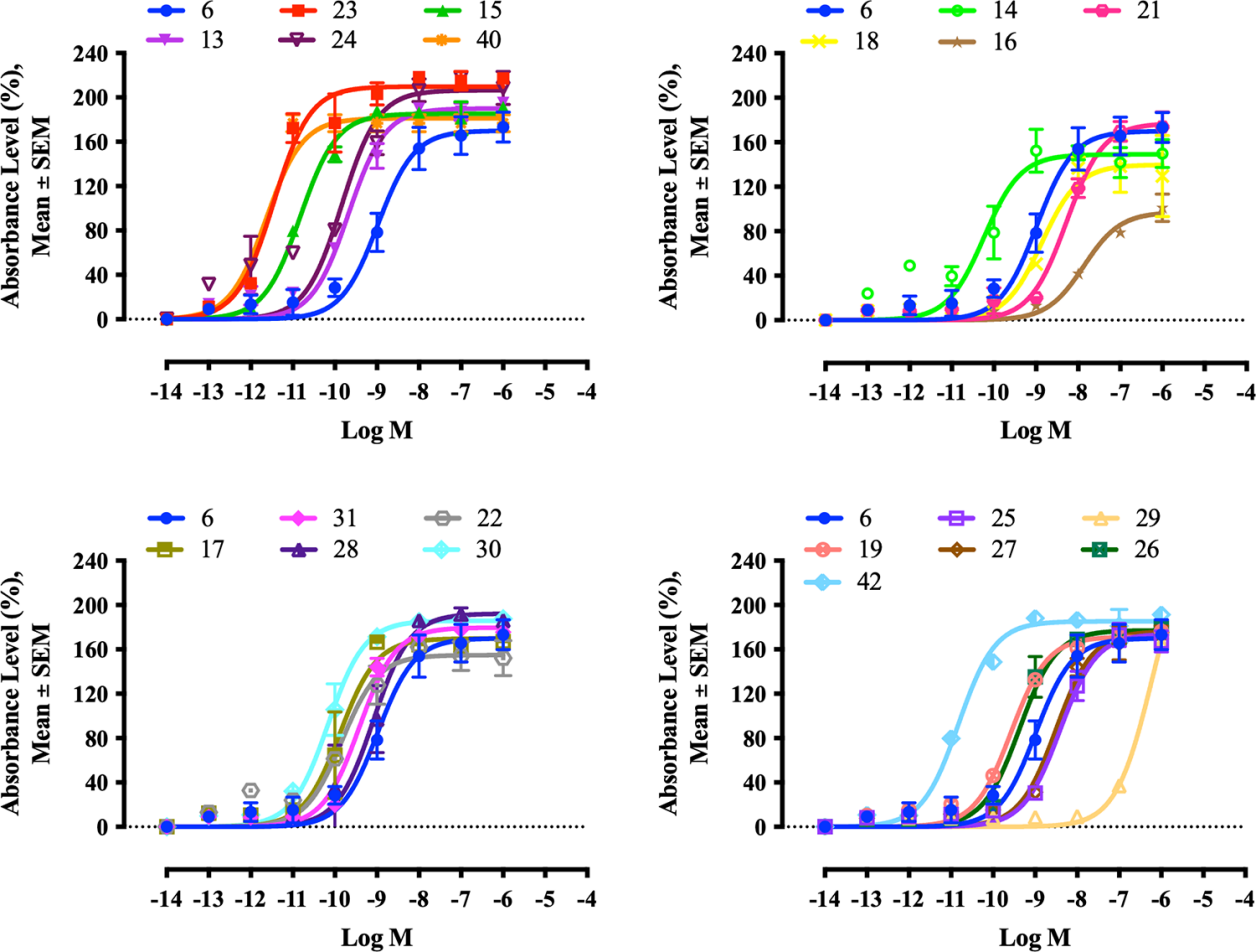
Concentration–response graph for log (agonist) vs sigmoidal dose–response to evaluate ATRA versus retinoid capacity to induce genomic response of Sil-15 reporter cells. Absorbance values of different retinoid doses were measured at 650 nm and analyzed using sigmoidal dose–response curves. Shown are the average absorbance of three independent experiments. Error bars indicate the standard error of the mean (SEM). Statistically significant differences are indicated by nonoverlapping 95% CI.

After screening the retinoids for genomic responses, they were tested for their nongenomic ability to rapidly phosphorylate ERK1/2 in the SH-SY5Y cell line. ATRA is known as a potent activator of ERK1/2 ^39–41^ and was used as the standard for comparison. The cells were treated with retinoids at concentrations ranging from 10^–5^ to 10^–11^ M for 1 h, and then potency and efficacy values were calculated for each retinoid (Figure 10). All retinoids induced ERK1/2 phosphorylation, except **27** and **43** which lacked nongenomic activity. The remaining retinoids were significantly more potent than ATRA, apart from EC23 which exhibited similar potency to ATRA. With respect to efficacy, 11 retinoids (EC23, GZ25, **15**, **19**, **22–24**, **26**, **30**, **31**, and **41**) had *E*_max_ values significantly higher than ATRA. Seven retinoids (**13**, **16**, **18**, **21**, **25**, **29**, and **40**) had *E*_max_ values significantly lower than ATRA. The efficacies of the remaining seven retinoids were similar to ATRA.

**Figure 10:**
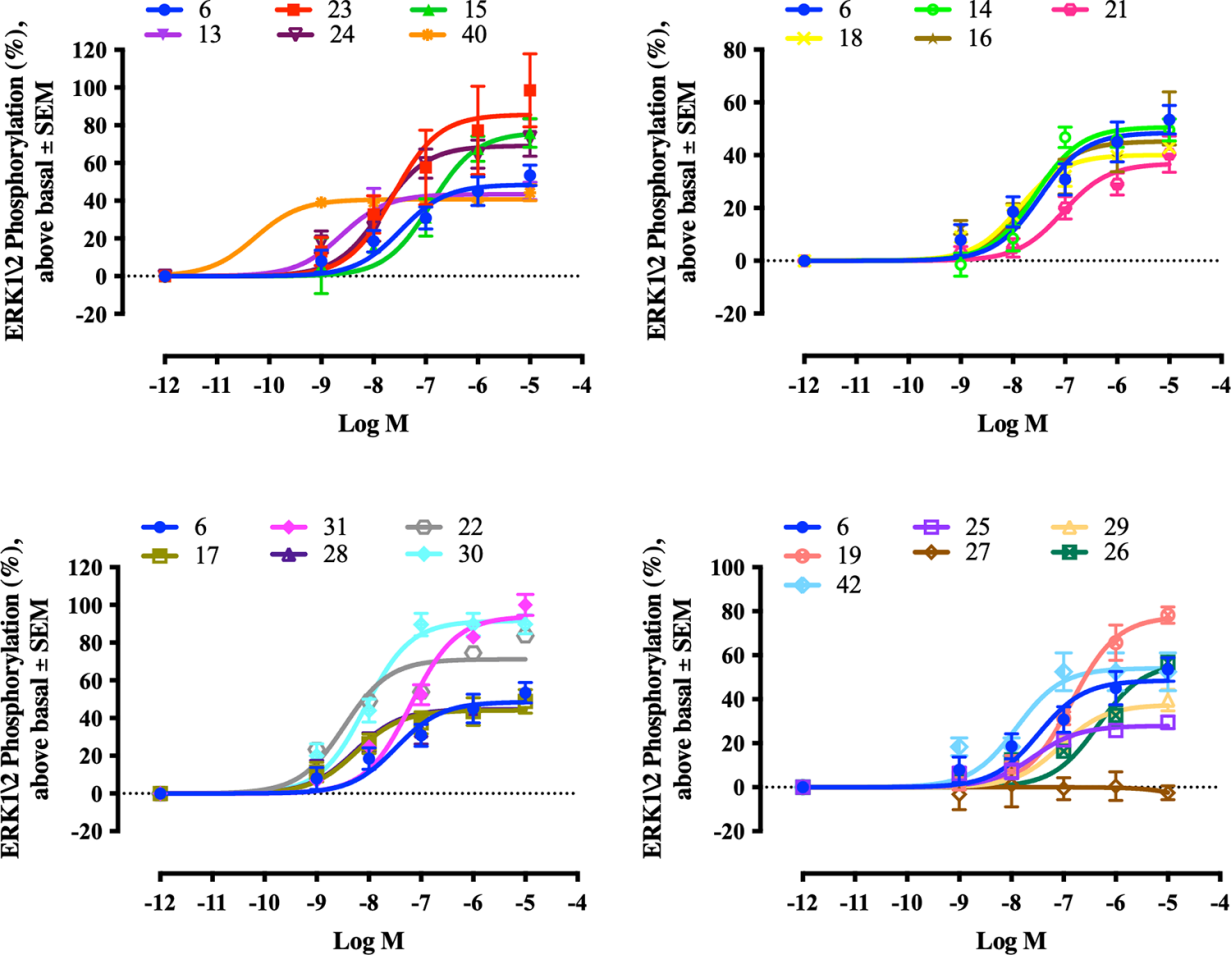
Sigmoidal concentration–response graphs for induction of ERK1/2 phosphorylation in SH-SY5Y cells. Relative activities of different retinoid doses were measured at 570 nm. The average absorbances in three independent experiments are shown. Error bars indicate SEM. Statistically significant differences are indicated by nonoverlapping 95% CI.

Following the characterization of retinoids for their genomic and nongenomic activities, the ability of retinoids to differentiate and induce neurite outgrowth in the neuroblastoma cell line, SH-SY5Y cells, was examined (Figure 11). Neurite outgrowth can be induced in this cell line by ATRA at 10 μM.^42^ Twenty-two retinoids were examined for their activity to induce neurite outgrowth at 10 nM concentration, as ATRA does not significantly induce such outgrowth at this low concentration (Table S5, ESI). The results showed that while **26** was unable to induce neurite outgrowth, eight retinoids (**17**, **18**, **21**, **22**, **25**, **28**, **29**, and **31**) induced neurite outgrowth but not significantly compared to ATRA. However, compounds **13–16**, **19**, **23, 24, 30**, and **40–43** significantly induced neurite outgrowth compared to ATRA, with compound **30** showing even higher activity compared to EC23 and GZ25.

**Figure 11:**
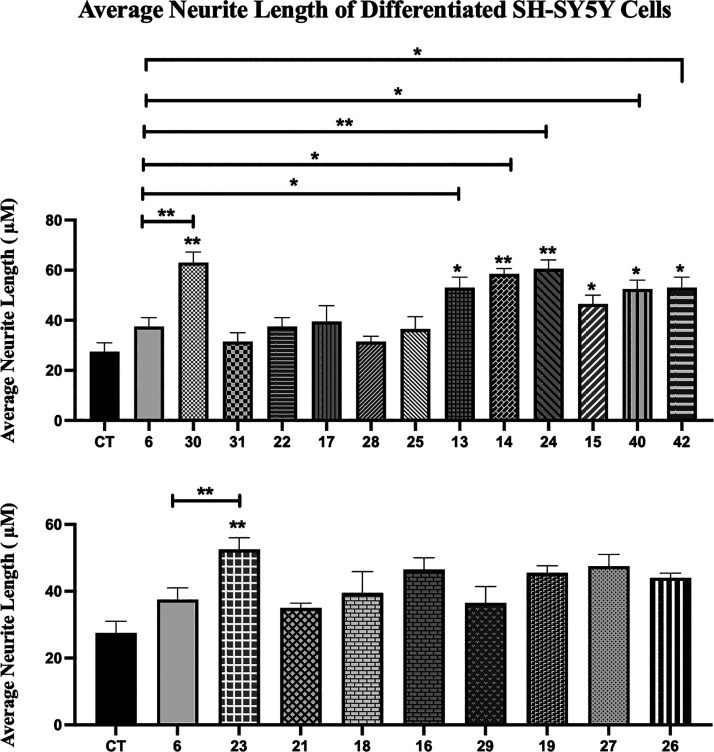
Neurite outgrowth of SH-SY5Y cells treated with retinoids. SH-SY5Y cells were treated with retinoids or DMSO CT treatment for 5 days. The average length of neurites extending from SH-SY5Y cells after 10 nM retinoid treatment was measured and compared with the control and ATRA. Shown are the mean values of three independent experiments. The average neurite length was calculated by dividing the total neurite length by the total number of neurites in each micrograph. Error bars indicate SEM (*p < 0.05; **p < 0.01; one-way ANOVA with Newman-Keuls multiple comparison test).

## Conclusions

We utilized a convergent synthesis approach involving the development of several unique building blocks to successfully synthesize 23 novel synthetic retinoids. These procedures are practical and scalable and represent a versatile platform with which further modifications for new retinoids could be made in the future. All 23 novel synthetic retinoids that were designed and synthesized were subsequently compared via biological characterization using both *in vitro* and cellular studies, which were complemented by binding affinity measurements, molecular docking studies, and MD simulations.

### Binding Mode and Relative Binding Strengths of the Synthetic Retinoid Library toward the RAR Isoforms

It has been shown that molecular docking can be used solely as an initial qualitative assessment of the potential of the retinoid to bind to the LBP of the RARs. Indeed, enhanced experimental and MD binding affinity studies are required to further understand the specific interactions between the synthetic retinoid and RARs. There were some significant differences between the docking scores, binding free energy estimations and measured binding affinities, which could be brought into better agreement with improved structural information on the binding poses. Armed with this information, more detailed and accurate absolute and relative binding affinity predictions could be made.^43,44^ It is important to note that all the synthetic retinoids used in these studies share similar overall chemical scaffolds, either based on EC23 or GZ25. However, subtle changes were made to their bulky hydrophobic head groups and polar carboxylate tails to identify potential key chemical functional groups that improve both binding affinity and selectivity to the distinct LBP configuration of each RAR isoforms. As a result of this, it was expected that the predictions from MD simulations were near-identical, as all 23 novel synthetic retinoids were designed to bind to the well-defined and largely hydrophobic LBP of the LBD in all three RAR isoforms. Therefore, the small differences that were observed in binding free energy estimations and binding affinities could be attributed to these small changes that were made across their chemical structures. This was exemplified in the modeled ligand-bound structures of **30** with RARα, RARβ, and RARγ through the differences in predictions about the hydrogen bonding networks made between the synthetic retinoid and each isoform.

### Genomic and Nongenomic Activities and Phenotypic Effects of the Synthetic Retinoid Library

Moreover, for a synthetic retinoid to stimulate the activation of the genomic and/or, nongenomic responses, it must participate in several essential preceding and successive biological events that occur as part of the retinoid signaling pathway. For instance, transport across the cell membrane is required before the synthetic retinoid can be subsequently bound to the Cellular Retinoic Acid Binding Proteins (CRABPs), a family of retinoid transporter proteins.^1^ For the genomic response pathway, it must be bound by the highly promiscuous CRABPII and shuttled across the nuclear membrane before it can be bound by the RARs. Subsequent conformational changes in the structure of the RARs are also required to facilitate the binding of the RXRs to form RAR/RXR heterodimers, as well as for successful coactivator recruitment. Similarly, to illicit any nongenomic activity, the synthetic retinoids must be bound by CRABPI before activating the recruitment of other regulatory proteins involved in pathways such as ERK1/2 phosphorylation. The cellular genomic activity and neurite outgrowth assays are, therefore, essential to fully contextualise the biological activities of the synthetic retinoids. It has been demonstrated that 10 of the novel synthetic retinoids, as well as EC23 and GZ25, induced significant neurite outgrowth compared to ATRA. Some of these compounds also showed comparable levels of high genomic activity. Since previous studies have suggested that dual-acting compounds have the greatest promise for use in therapeutics, it is important to highlight that a total of four (**15**, **23**, **24**, and **30**) out of the 23 novel compounds also showed activity toward the nongenomic pathway. Importantly, these compounds also showed a significant increase in neurite outgrowth with compound 30 showing even higher activity compared to EC23 and GZ25. Further to this, recent pharmacokinetic/pharmacodynamic studies using **30** (also known as NVG0645, Ellorarxine^45^) have confirmed its promise in preclinical trials for its use as a potential therapeutic in the treatment of neurodegenerative diseases.^46^

## Experimental Section

### Molecular Biology

#### Protein Expression and Purification

RARα/γ proteins were expressed in BL21­(DE3) cells (NEB) transformed into pET50b (Novagen) and pOPINS3C vectors, respectively, carrying the gene construct and containing a His_6_-3C-NusA tag or His_6_-3C-SUMO tag used for solubility.^47^ Transformations were carried out under standard conditions using Quick Transformation Protocol (New England Biolabs). Successful transformants were grown overnight in 2xYT media (Melford) and frozen for storage at −80 °C in 50% glycerol. Expression cultures were inoculated from glycerol stocks into 25 mL of 2xYT media (Melford) with 50 μg/mL kanamycin or 100 μg/mL ampicillin (Sigma-Aldrich) and grown overnight with shaking (37 °C, 150 rpm). The overnight cultures were transferred into 1 L expression flasks (2xYT/ampicillin 100 μg/mL or 2xYT/kanamycin 50 μg/mL) and grown with shaking at 37 °C, 150 rpm. Induction was carried out at an OD600 of 0.6–0.8, with 1 mL of 1 M IPTG (1 mM in culture), before shaking overnight (20 h) at 18 °C. The resulting cultures were spun down into pellets using an Avanti Hi-Speed centrifuge (JLA 8.1000, 4000 rpm, 30 min, 4 °C), before the supernatant was removed and the bacterial pellet were frozen at −80 °C.

RARα/γ (pET50b or pOPINS3C, BL21­(DE3) (NEB)) were resuspended from pellet with 20 mL Wash Buffer (20 mM Tris-HCl, 300 mM NaCl and 20 mM imidazole, pH 8) and the resulting suspension was sonicated on ice (40% power, 2 min, repeated twice with 1 min rest in-between) before centrifuging (Avanti Hi-Speed JA25.50, 20,000 rpm, 1 h, 4 °C). Supernatants were clarified using 0.45 μm syringe filters (Thermo Fisher Scientific). Affinity chromatography and size-exclusion (SEC) chromatography were carried out on AKTA Pure25 (Cytiva). Supernatants were loaded onto the HisTrap HP column (5 mL, Cytiva) and washed using 25 mL of wash Buffer. The column was washed with Wash Buffer until UV trace returned to baseline, before RARα/γ was eluted using a gradient of Elution Buffer (20 mM Tris-HCl, 300 mM NaCl, 500 mM imidazole pH 8). The resulting protein-containing fractions were analyzed by SDS-PAGE (200 V, 30 min) using SurePAGE Bis-Tris 12% precast gels (Genscript). Protein concentrations were estimated using absorbance at 280 nm on a DS-11 spectrophotometer (Denovix) with calculated molecular weights and extinction coefficients (Expasy). RAR proteins were cleaved overnight from 3C-NusA tag or 3C-SUMO-His tag using 1 Unit/100 μg protein of HRV3C protease (Thermo Fisher Scientific) at 4 °C. Solution after digestion was loaded onto a HiLoad 16/600 Superdex 75 prep-grade column (Cytiva) and cleaved RARα/γ was separated from the tag based on molecular weight and eluted from the column using SEC Buffer (20 mM Tris-HCl, 300 mM NaCl and 0.5 mM TCEP, pH 8) into 2 mL fractions. The resulting protein fractions were analyzed by SDS PAGE (200 V, 30 min) using SurePAGE Bis-Tris 12% precast gels (Genscript), before being concentrated to 5 mg/mL using centrifugal concentrators (Sartorius) and frozen at −80 °C in small aliquots for use in binding assays.

#### Fluorescence Competition Binding Assays

Solutions of DC271 (300 nM, <1% EtOH), RARα/γ (300 nM, in SEC Buffer) and EC23 (600 nM, <1% EtOH) were prepared immediately before use. A 96-well black, NBS fluorescence plate (Corning) was cleaned using compressed air, and maximum signal control, mid signal control, and all test compound wells were loaded with 50 μL of RAR and DC271 solutions. Minimum signal control wells were loaded with 50 μL of DC271 and assay buffer solutions. Mid signal control wells were then loaded with 50 μL EC23, and maximum and minimum control wells with 1% EtOH solution (50 μL). A dilution series of test compounds were prepared and aliquoted to the plate (50 μL). Plates were spun using a Hettich benchtop centrifuge (1500 rpm, 2 min, 4 °C) to ensure thorough mixing, before readings using a Synergy H4 plate reader (excitation/emission 355/460 nm) were taken. The total well volume was 150 μL, and the on-plate concentration of RAR protein and DC271 was 100 nM. Nonlinear least-squares regression analyses were performed in BioKin Dynafit^47^ and results plotted in Microsoft Office Excel 365. Binding *K*_D_ values and standard deviations reported were calculated in Microsoft Office Excel 365 from an average of 3–9 replicates measured across 1–3 independent assay experiments.

Binding *K*_D_ values were also expressed as binding free energies (Δ*G*_EXP_) using

$$\Delta G_{\exp}=-\mathrm{RT}\ln K$$

where *R* is the gas constant (8.314 J/K/mol), *T* is the temperature of the reaction (298.1K), and *K* is the equilibrium constant. Values were expressed in kcal/mol to be comparable to the free energy estimations using ESMACS protocols.

#### Cell Lines

The Sil-15 cell line^38^ was used in the X-gal RA reporter assay described below. The cells were grown in Dulbecco’s modified Eagle’s medium (DMEM) containing 10% fetal bovine serum (FCS) (Invitrogen/Gibco), 0.6% penicillin-streptomycin (Gibco) and 0.8 mg/mL G418 sulfate (Sigma) for selection. They were maintained in a humidified atmosphere containing 5% CO_2_ at 37 °C. The medium was changed three times a week and the cells were passaged at 70% confluence using 0.05% trypsin-EDTA.

SH-SY5Y cell line^48^ as used in ERK phosphorylation and neurite outgrowth assays described below. The cells were grown in DMEM containing 10% FCS at 37 °C with 5% CO_2_. The medium was changed three times a week and the cells were passaged at 70% confluence using 0.05% trypsin-EDTA.

#### X-Gal-Based RA Reporter Assays

Sil-15 cells are F9 teratocarcinoma cells stably transfected with a plasmid containing the LacZ gene under the control of a DR5-type RA response element (RARE). The reporter cell line was used to detect the transcriptional genomic activity of ATRA and other retinoids by colorimetrically quantifying the insoluble blue product generated by β-galactosidase from X-gal during the assay.^49^ The assay was performed by plating 100,000 cells per well in the 0.1% gelatin-coated 96-well plates and leaving them to attach overnight. The ATRA/retinoids dilutions ranging from 10^–6^ to 10^–14^ M were incubated on the reporter cells overnight at 37 °C, in 5% CO_2_. A standard curve of ATRA was included in each experiment to ensure the reproducibility of the results between assays performed on different days. All the assays for ATRA as well as the retinoids were quantified in triplicate. Serial dilutions of retinoids were prepared from a stock solution under red/orange light. The stock solutions prepared in DMSO for each retinoid varied depending on the solubility of each compound. Each stock was stored at −20 °C and protected from light. The next day, Sil-15 cells were washed with PBS, fixed with 1% glutaraldehyde and 1 mM MgCl_2_ solution for 15 min, and developed in X-gal developing solution (0.2% X-Gal, 1 mM MgCl_2_, 3.3 mM potassium ferricyanide and 3.3 mM potassium ferrocyanide in PBS). The plates were read on an *E*_max_ microplate reader (Molecular Devices) at 650 nm.

#### ERK1/2 Phosphorylation

To quantify ERK1/2 phosphorylation, SH-SY5Y cells (100,000 cells/well) were plated onto 96-well plates and serum-starved for 24 h in DMEM. Cells were assayed in serum-free DMEM and stimulated for 60 min at 37 °C in a humidified atmosphere. Retinoids were tested at concentrations ranging from 10 to 4 to 10–11 M and at a final concentration of 0.1% DMSO. At the end of the assay, the medium was removed, and cells were lysed with lysis buffer supplied in the AlphaScreen SureFire ERK1/2 kit (PerkinElmer). The assay was performed in 384-well white Proxiplates according to the manufacturer’s instructions and using the beads supplied in the protein A general IgG detection kit (PerkinElmer). Briefly, 4 μL samples were incubated with 7 μL of the freshly prepared mixture containing donor and acceptor beads. Plates were incubated at room temperature and read with the Envision system (PerkinElmer Life Sciences) using AlphaScreen settings.

### Neurite Outgrowth

SH-SY5Y cells were plated at 10,000 cells/well in 12-well plates containing 16 mm acid-treated/poly l-lysine-coated glass coverslips. After 24 h, retinoids were added to the medium and tested at two different concentrations, 10 μM and 10 nM, with a final DMSO concentration of 0.01 or 0.0001%, respectively. The plates were incubated for 5 days at 5% CO2/37 °C. All retinoid concentrations were tested in triplicate. Cells were fixed using 4% paraformaldehyde and stained for β-III tubulin (1:1000; Sigma). For each neurite outgrowth experiment, 3 coverslips (in 3 wells) were used. ImageJ software with the NeuronJ plugin was used to quantify neurite outgrowth on stained cells in each of the 10 randomly selected images of each coverslip taken using a Nikon Eclipse E400 fluorescence microscope. The average neurite length was calculated for each image by dividing total neurite length by the total number of neurites per image.

### Statistical Analysis

All data are presented as mean ± SEM of three independent experiments with biological triplicates. Statistical analyses were performed in Microsoft Office Excel 2019 or GraphPad Prism 7.0c version (Prism, GraphPad Software, San Diego, CA). Neurite outgrowth data were analyzed by one-way ANOVA with Newman-Keuls multiple comparison test as appropriate; P value <0.05 was considered statistically significant. *P < 0.05, **P < 0.01. Data for X-Gal and ERK1/2 phosphorylation were analyzed using sigmoidal dose–response analysis of log (agonist) versus response curve (stimulation). The results of analyses were presented as Emax and EC50 with 95% confidence interval limits (95% CI).

### Multiple Sequence Alignments

Multiple sequence alignments of human Retinoic Acid Receptors, RARα^182–417^ (UniProt ID: P10276), RARβ^182–417^ (UniProt ID: P10826), and RARγ^182–417^ (UniProt ID: P13631), were prepared using ESPript 3.0.^50^ Secondary structure of RARα between residues 182–417 were annotated as α-helices (α1–9) and β-sheets (β1–2). Both Identical residues and isoform-specific residues across all the RAR isoforms were also annotated.

### Computational Studies

#### Initial Docking Studies

Molecular structures of all retinoid compounds were generated using Spartan 14 and minimized using a molecular mechanics force field. AM1 semiempirical methods were then used to produce conformer distributions, which were then subject to an equilibrium geometry calculation (Hartee-Fock, 3–21G) for further reminimization. All conformations were then exported as. mol2 files.

All conformations of all compounds were carried forward for docking into RAR crystal structures obtained from the RCSB protein data bank (RARα: KMR3, RARβ: 1XAP, RARγ: 2LBD), with bound ligands removed. Water and solvent molecules were also removed, and hydrogens were added to protein residues using GOLD default settings. The binding site was defined as within a 15 Å sphere from the center of the existing bound ligand, encapsulating the entire binding pocket. Genetic algorithm parameters were set to default, and ChemScore was selected as the target function. Search efficiency was set to the maximum value of 200% to allow for the highest degree of ligand flexibility, alongside the selection of the additional ligand parameters “flip ring corners” and “match template conformations”. All docking solutions were retained and ranked by ChemScore. For each compound, the 3 highest ChemScore-ranked binding poses with unique conformational structures were carried into molecular dynamics to provide a variety of starting points. ChemScore ratios for RARβ: RARα, RARβ:RARγ, RARα: RARγ were plotted in GraphPad Prism 7.0c version (Prism, GraphPad Software, San Diego, CA). Modeled ligand-protein structure for **30** was visualized in PyMOL v2.6.^51^

#### Molecular Dynamics Simulations

##### ESMACS

We use the ESMACS (enhanced sampling of molecular dynamics with approximation of continuum solvent)^52^ protocol for the binding free energy calculations. The protocols use ensembles of replicas to obtain reproducible binding affinity estimates with robust uncertainty estimates. ESMACS is based on MMPBSA (molecular mechanics Poisson–Boltzmann surface area) calculations but incorporates a variety of sampling approaches and entropic calculations.^53,54^ It is an end-point free energy calculation, in which the binding free energy, Δ*G*_binding_ (reported as Δ*G*_ESMACS_), is calculated using

$$\Delta G_{\mathrm{binding}}=G_{\mathrm{com}}-G_{\mathrm{pro}}-G_{\mathrm{lig}}$$

where *G*_*i*_ is the free energy of component *i* which corresponds to either complex (com), protein (pro), or ligand (lig), and is calculated from a set of structures from MD simulations. Here conformations of the complex, receptor, and compound are all extracted from simulation of the complex, a protocol termed 1traj.^55^ This is the commonly used approach for the end-point free energy methods. It achieves good convergence because of the cancellation between the noisy terms of the internal energies of the ligand, receptor and complex. Upon binding, however, conformational changes occur for both protein and ligands, associated with adaptation-free energies.^52^ They are the energy differences between the free state and the bound state. When a set of compounds is investigated for the binding of the same protein, the adaptation energy can be calculated in relative terms using the average energy of the protein,^53^ a protocol designated as 1traj-ar.^55^ Studies have shown that the inclusion of adaptation energies clearly improves the predictions of binding free energy ranking for some molecular systems,^52,53,56^ while the situation may be more complicated for others.^54,55^ The ESMACS protocol has options to include configurational entropy to the free energy calculations, obtained from normal model analyses or other approximations. Although the inclusion of entropic contribution can bring the estimated free energies closer to more physically realistic values, it fails to improve correlations in most cases.^54,55,57,58^ For a rational drug development project, the correct ranking of binding affinities is more important for the selection of compounds for further investigation. In the current study, the entropic contribution is omitted in the ESMACS free energy calculations.

##### Model Preparation

A set of 63 compounds was used for each of the 3 retinoid receptors (RARα, RARβ, and RARγ). The compounds were docked into the proteins, using structures from the protein databank (PDB IDs: 3KMR, 1XAP, and 2LBD, respectively). The X-ray structures were solved with different lengths of residues. To make the simulations comparable, the same number of residues were selected for the molecular modeling, containing 234 amino acids each. The missing residues in the X-ray structure for RARβ were inserted using those for RARγ after aligning the two structures. All water molecules in the pdb files were retained.

Preparation and setup of the simulations were implemented using BAC (binding affinity calculator),^59^ including parametrization of the compounds, solvation of the complexes, electrostatic neutralization of the systems by adding counterions, and generation of configurations files for the simulations. The Amber package^60^ was used for the setup of the systems and the analyses of the results. Ligand parametrizations were produced using the general Amber force field 2 (GAFF2). The partial charges of the compounds were generated using the AM1-BCC method. The protonation states of the residues were assigned using the “reduce” module of AmberTools.^61^ The Amber ff14SB force field was used for the protein, and TIP3P for water molecules.^61^ All ligand-protein complexes were solvated in orthorhombic water boxes with a minimum extension from the protein of 14 Å. Counterions were added to electrostatically neutralize the systems.

##### Molecular Dynamics Simulation

The standard ESMACS protocol^52^ was used, in which an ensemble of 25 replicas was simulated for each compound-protein complex. NAMD^62^ was used as the MD engine for all of the equilibration and production runs. For each individual simulation, energy minimizations were first performed with heavy protein atoms restrained at their initial positions. The initial velocities were then generated independently from a Maxwell–Boltzmann distribution at 50 K, and the systems were heated up to and kept at 300 K within 60 ps. Finally, 10 ns production simulations were run for each replica for the ESMACS simulations. All simulations were performed on SuperMUC-NG at the Leibniz Supercomputing Centre in Germany. For one nanosecond simulation, it took ∼1.1 h on 48 CPUs on SuperMUC-NG. Ratios for ESMACs free energy estimations (Δ*G*_binding_) for RARβ:RARα, RARβ:RARγ, RARα:RARγ were plotted in GraphPad Prism 7.0c version (Prism, GraphPad Software, San Diego, CA). Modeled ligand-protein structures for **30** were visualized in PyMOL v2.6.^51^

### Chemistry

Reagents were purchased from Sigma-Aldrich, Acros Organics, Alfa-Aesar, and Fluorochem. Reagents were purified, if required, by recrystallization or distillation/sublimation under vacuum. Solvents were used as supplied by Fisher Scientific or Sigma-Aldrich, and dried before use if required with appropriate drying agents. Thin-layer chromatography (TLC) was conducted using Merck Millipore silica gel 60G F254 25 glass plates and/or TLC-PET foils of aluminum oxide with fluorescent indicator 254 nm (40 mm × 80 mm) with visualization with a UV lamp or appropriate staining agents. Flash column chromatography was performed using SiO_2_ from Sigma-Aldrich (230–400 mesh, 40–63 μM, 60 Å), and monitored using TLC. Sublimation/distillation was performed using a Buchi Glass Oven B-585 Kugelrohr operating at a pressure between 4 and 10 Torr. NMR spectra were recorded using Varian VNMRS-700, Varian VNMRS-600, Bruker Avance-400, or Varian Mercury-400 spectrometers operating at ambient probe temperature. NMR peaks are reported as singlet (s), doublet (d), triplet (t), quartet (q), broad (br), septet (sept), combinations thereof, or as a multiplet (m), with reference to the following deuterated solvent signals: CDCl_3_ (^1^H = 7.26 ppm, ^13^C = 77.0 ppm), (CD_3_)_2_SO (^1^H = 2.50 ppm, ^13^C = 39.5 ppm). ESMS was performed using a TQD (Waters Ltd., U.K.) mass spectrometer with an Acquity UPLC (Waters Ltd., U.K.) system, and accurate mass measurements were obtained using a QtoF Premier mass spectrometer with an Acquity UPLC (Waters Ltd., U.K.). ASAP measurements were performed using an LCT Premier XE mass spectrometer and an Acquity UPLC (Waters Ltd., U.K.). IR spectra were recorded using a PerkinElmer FTIR spectrometer. Unless otherwise noted, all tested compounds were found to be ≥95% pure according to HPLC analysis.

#### 2,5-Dichloro-2,5-dimethylhexane, **1**

Conc. HCl (600 mL) was slowly added to 2,5-dimethyl-hexane-2,5-diol (70 g, 205.16 mmol), and the resultant slurry was stirred for 2 h. The mixture was filtered, and the filter cake was washed thoroughly with H_2_O. The isolated solid was dissolved in EtOAc, washed with sat. NaHCO_3_, H_2_O, and brine, dried (MgSO_4_), and evaporated to give compound **1** as a colorless solid (70.3 g, 80%): 1H NMR (400 MHz, CDCl_3_) δ 1.59 (s, 12H), 1.94 (s, 4H); 13C NMR (151 MHz, CDCl_3_) δ 32.7, 41.4, 70.5; all other data matched the literature.^63^

#### 1,1,4,4-Tetramethyl-1,2,3,4-tetrahydronaphthalene, **2a**

Compound **1** (30.0 g, 163.8 mmol) and benzene (29.3 mL, 327.6 mmol) were added to anhydrous DCM (250 mL) and the solution was cooled to 0 °C. AlCl_3_ (13.0 g, 97.5 mmol) was added carefully, in portions, and the resultant solution was stirred at RT for 5 h. The solution was poured into crushed ice, and extracted with DCM. The organics were washed with sat. NaHCO_3_ and H_2_O, dried (MgSO_4_), and evaporated to give a crude oil (19 g). This was purified by SiO_2_ chromatography (100% heptane) to give compound **2a** as a colorless oil which slowly crystallized on standing (19.69 g, 64%): all analytical data matched the literature.^64^

#### 1,1,4,4,6-Pentamethyl-1,2,3,4-tetrahydronaphthalene, **2b**

Compound **1** (10.0 g, 54.7 mmol) was dissolved in toluene (270 mL) under N_2_. AlCl_3_ (5.47 g, 41 mmol) was added portionwise over 15 min and the resultant solution was stirred at RT for 3 h. H_2_O (100 mL) was slowly added over 10 min, and the solution was then diluted with EtOAc. The organics were washed with 5% aq NaOH, H_2_O, and brine, dried (MgSO_4_), and evaporated to give a crude brown oil (12.5 g). This was purified by dry column vacuum chromatography (100% heptane to 9:1, heptane/EtOAc) to give compound **2b** as a light yellow oil which slowly crystallized on standing (10.94 g, 95%): all analytical data matched the literature.^65^

#### 6-Methoxy-1,1,4,4-tetramethyl-1,2,3,4-tetrahydronaphthalene, **2c**

Compound **1** (10.0 g, 54.7 mmol) and anisole (15.2 mL, 140 mmol) were added to anhydrous DCM (70 mL) and the solution was cooled to 0 °C. AlCl_3_ (0.1 g, 0.75 mmol) was added carefully, in portions, and the resultant solution was stirred at RT for 5 h. The solution was poured into crushed ice, and extracted with DCM. The organics were washed with sat. NaHCO_3_ and H_2_O, dried (MgSO_4_), and evaporated to give a crude oil (19 g). This was purified by dry column vacuum chromatography (100% heptane to 95:5, heptane/EtOAc) to give **2c** as a colorless oil (11.14 g, 93%): all analytical data matched the literature.^66^

#### Method A. 6-Iodo-1,1,4,4-tetramethyl-1,2,3,4-tetrahydronaphthalene, **3a**

Compound **2a** (11.46 g, 60.9 mmol), I_2_ (7.77 g, 30.6 mmol), and H_5_IO_6_ (3.49 g, 15.3 mmol) were added to a mixture of AcOH (250 mL), H_2_O (25 mL), and H_2_SO_4_ (13 mL), and the resultant solution was stirred at 70 °C for 16 h. The solution was cooled, and extracted with EtOAc. The organics were washed with sat. Na_2_S_2_O_3_, H_2_O, and brine, dried (MgSO_4_), and evaporated to give a crude orange oil (17 g). This was purified by dry column vacuum chromatography (eluting with heptane) to give compound **3a** as a light yellow oil which slowly crystallizes on standing (16.25 g, 85%): all data matched the literature.^11^

#### 6-Iodo-1,1,4,4,7-pentamethyl-1,2,3,4-tetrahydronaphthalene, **3b**

Compound **2b** (9.62 g, 47.5 mmol) was reacted with I_2_ (6.03 g, 23.8 mmol) and H_5_IO_6_ (2.71 g, 11.9 mmol) in AcOH (250 mL), H_2_O (25 mL) and H_2_SO_4_ (13 mL) according to Method A to give compound 3b as a light yellow oil which slowly crystallizes on standing (10.61 g, 68%): ^1^H NMR (600 MHz, CDCl_3_) δ 1.28 (s, 12H), 1.68 (s, 4H), 2.40 (s, 3H), 7.18 (s, 1H), 7.72 (s, 1H); ^13^C NMR (151 MHz, CDCl_3_) δ 27.6, 31.7, 31.8, 33.8, 34.0, 34.9, 98.2, 127.9, 137.0, 138.1, 144.8, 145.2; IR (ATR) *v*_max_/cm^–1^ 2955s, 2921m, 2856m, 1478m, 1456m, 1362s, 1071s, 882s, 698m; MS (ASAP) *m*/*z* = 328.1 [M + H]^+^; HRMS (ASAP) calcd. for C_15_H_21_I [M + H]^+^: 328.0688, found 328.0675.

#### 6-Iodo-7-methoxy-1,1,4,4-tetramethyl-1,2,3,4-tetrahydronaphthalene, **3c**

Compound **2c** (10.0 g, 45.8 mmol) was reacted with I_2_ (5.81 g, 22.9 mmol) and H5IO_6_ (2.61 g, 11.5 mmol) in AcOH (250 mL), H_2_O (25 mL) and H_2_SO_4_ (13 mL) according to Method A to give compound **3c** as a colorless crystalline solid (12.20 g, 77%): 1H NMR (600 MHz, CDCl_3_) δ 1.25 (s, 6H), 1.29 (s, 6H), 1.63–1.70 (m, 4H), 3.86 (s, 3H), 6.74 (s, 1H), 7.66 (s, 1H); 13C NMR (151 MHz, CDCl_3_) δ 31.7, 31.8, 33.7, 34.6, 34.9, 35.0, 56.3, 83.3, 108.8, 137.5, 139.7, 146.7, 155.8; IR (ATR) *v*_max_/cm^–1^ 3000w, 2958m, 2925m, 2864w, 1586m, 1485m, 1456m, 1363m, 1245s, 1039s; MS­(ASAP): *m*/*z* = 344.1 [M + H]^+^; HRMS (ASAP) calcd. for C_15_H_21_OI [M + H]^+^: 344.0637, found 344.0641.

#### 6-Ethynyl-1,1,4,4-tetramethyl-1,2,3,4-tetrahydronaphthalene, **4a**

Et_3_N (150 mL) was added to a 3-neck, 250 mL RBF under N_2_. The solution was degassed by sparging with N_2_ for 1 h. Compound **2a** (10.0 g, 31.8 mmol), Pd­(PPh_3_)_2_Cl_2_ (0.22 g, 0.32 mmol), CuI (0.061 g, 0.32 mmol), and trimethylsilylacetylene (5.3 mL, 38.2 mmol) were then added under N_2_ and the suspension was stirred at RT for 20 h. The mixture was diluted with heptane and passed through Celite/SiO_2_ (eluting with heptane), and the resultant solution was evaporated to give a crude brown oil (10.36 g). This was purified by SiO_2_ chromatography (100% heptane) to give trimethyl­[2-(5,5,8,8-tetramethyl-5,6,7,8-tetrahydronaphthalen-2-yl)­ethynyl]­silane as a yellow oil (9.99 g, >100%). This was dissolved in dissolved in MeOH/MTBE (1:1, 160 mL). A solution of NaOH (0.96 g, 24.0 mmol) in H_2_O (15 mL) was then added and the resultant solution stirred at RT for 72 h. The solution was diluted with MTBE, washed with H_2_O and brine, dried (MgSO_4_), and evaporated to give a crude yellow oil (6.6 g). This was purified by SiO_2_ chromatography (100% heptane) to give compound **4a** as a colorless oil that slowly solidified (6.06 g, 90% over two steps): all data matched the literature.^11,11^

#### 6-Ethynyl-1,1,4,4,7-pentamethyl-1,2,3,4-tetrahydronaphthalene, **4b**

Anhydrous toluene (130 mL) was degassed by sparging with N_2_ for 1 h. Compound **3b** (5.5 g, 16.8 mmol), trimethylsilylacetylene (2.8 mL, 20.1 mmol), Et_3_N (5.6 mL, 40.2 mmol), Pd­(PPh_3_)_2_Cl_2_ (294 mg, 0.42 mmol) and CuI (80 mg, 0.42 mmol) were then added under N_2_ and the resultant suspension was stirred at RT for 72 h. The suspension was diluted with heptane, passed through Celite/SiO_2_ and the extracts were evaporated to give a crude oil (6 g). This was purified by dry column vacuum chromatography (100% heptane to 95:5, heptane/EtOAc) to give trimethyl­[2-(3,5,5,8,8-pentamethyl-5,6,7,8-tetrahydronaphthalen-2-yl)­ethynyl]­silane as a yellow oil (5.80 g, >100%). This was dissolved in MTBE/MeOH (100 mL, 1:1), whereupon a solution of NaOH (0.52 g, 12.95 mmol) in H_2_O (10 mL) was added and the resultant solution was stirred at RT for 16 h. The solution was diluted with EtOAc, and the organics were washed with H_2_O and brine, dried (MgSO_4_), and evaporated to give a crude yellow oil (3.55 g). This was purified by SiO_2_ chromatography (100% heptane) to give compound **4b** as a yellow oil that slowly solidified (3.37 g, 89% over two steps): ^1^H NMR (600 MHz, CDCl_3_) 1.30 and 1.31 (s, 12H), 1.71 (s, 4H), 2.44 (s, 3H), 3.23 (s, 1H), 7.17 (s, 1H), 7.46 (s, 1H); ^13^C NMR (151 MHz, CDCl_3_) δ 20.2, 31.7, 31.8, 33.8, 34.2, 35.0, 35.0, 79.6, 83.0, 119.2, 127.5, 130.8, 137.3, 142.3, 145.9; IR (ATR) *v*_max_/cm^–1^ 3308m, 2952m, 2922m, 2863m, 2104m, 1494m, 1461m, 1389m, 1273m, 899m; MS­(ASAP): *m*/*z* = 227.2 [M + H]^+^; HRMS (ASAP) calcd. for C_17_H_23_ [M + H]^+^: 227.1800, found 227.1790.

#### 6-Ethynyl-7-methoxy-1,1,4,4-tetramethyl-1,2,3,4-tetrahydronaphthalene, **4c**

Anhydrous toluene (100 mL) was degassed by sparging with N_2_ for 1 h. Compound **3c** (8 g, 23.23 mmol), trimethylsilylacetylene (4.02 mL, 29.05 mmol), Et_3_N (8.1 mL, 58.1 mmol), Pd­(PPh_3_)_2_Cl_2_ (407 mg, 0.58 mmol) and CuI (110 mg, 0.58 mmol) were then added under N_2_ and the resultant suspension was stirred at RT for 40 h. The suspension was diluted with heptane and passed through Celite/SiO_2_ and the extracts were evaporated to give a crude oil (10 g). This was purified by dry column vacuum chromatography (100% heptane to 95:5, heptane/EtOAc) to give [2-(3-methoxy-5,5,8,8-tetramethyl-5,6,7,8-tetrahydronaphthalen-2-yl)­ethynyl]­trimethylsilane as a white solid (6.96 g, 95%). This was dissolved in MTBE/MeOH (100 mL, 1:1), whereupon a solution of NaOH (0.59 g, 14.75 mmol) in H_2_O (10 mL) was added and the resultant solution was stirred at RT for 16 h. The solution was diluted with EtOAc, and the organics were washed with H_2_O and brine, dried (MgSO_4_), and evaporated to give a crude brown solid (5 g). This was purified by dry column vacuum chromatography (100% heptane to 9:1, heptane/EtOAc) to give compound **4c** as a colorless crystalline solid (4.88 g, 91%): ^1^H NMR (600 MHz, CDCl_3_) δ 1.25 (s, 6H), 1.29 (s, 6H), 1.63–1.70 (m, 4H), 3.25 (s, 1H), 3.88 (s, 3H), 6.78 (s, 1H), 7.40 (s, 1H); 13C NMR (151 MHz, CDCl3) δ 31.6, 31.8, 33.5, 34.7, 34.9, 34.9, 55.7, 79.8, 80.6, 108.2, 108.7, 132.4, 137.1, 147.6, 158.1; IR (ATR) *v*_max_/cm^–1^ 3308m, 2952m, 2865m, 2111m, 1608w, 1599w, 1498m, 1457m, 1256s, 1154m, 1047m, 665s; MS­(ASAP): *m*/*z* = 243.2 [M + H]^+^; HRMS (ASAP) calcd. for C17H23O [M + H]^+^: 243.1749, found 243.1738.

#### Trimethyl­({3,3,6,6-tetramethyl-2-[(trimethylsilyl)­oxy]­cyclohex-1-en-1-yl}­oxy)­silane, **6**

To anhydrous toluene (50 mL) was added sodium (1.20 g, 52.1 mmol) under N_2_, and the resultant mixture was heated to reflux until the sodium melted. The flask was then removed from the heat, and then **5** (2.69 g, 10.41 mmol) and chlorotrimethylsilane (6.72 mL, 53.0 mmol) were added and the resultant suspension was then stirred at reflux overnight. The purple suspension was then cooled, and filtered under a flow of N_2_, washing with toluene, then THF. The filtrate was then evaporated to give a crude light yellow oil (3.2 g), which was purified by Kugelrohr distillation (120 °C, 3.6 Torr) to give **6** as a clear oil (2.64 g, 81%): ^1^H NMR (400 MHz, CDCl_3_) δ 0.19 (s, 18H), 1.03 (s, 12H), 1.44 (s, 4H). All other data matched the literature.^67^

#### 3,3,6,6-Tetramethylcyclohexane-1,2-dione, **7**

To a solution of 6 (2.6 g, 8.2 mmol) in DCM was added bromine (0.42 mL, 8.2 mmol) dropwise over 5 min. The resultant yellow solution was stirred at RT for 1 h, before being diluted with DCM, and treated with sat. Na_2_S_2_O_3_, then washed with H_2_O, dried (MgSO_4_), and evaporated to give a crude yellow solid (1.5 g). This was purified by recrystallization from heptane to give 7 as a yellow crystalline solid (1.07 g, 78%): 1H NMR (700 MHz, CDCl_3_) δ 1.14 (s, 4H), 1.85 (s, 12H); 13C NMR (176 MHz, CDCl3) δ 22.9, 34.7, 48.6, 207.3; IR (ATR) *v*_max_/cm^–1^ 2973m, 2940w, 2870w, 1706s, 1599w, 1459m, 1372m, 1102m, 931m; MS­(ES): *m*/*z* = 169.3 [M + H]^+^. All other data matched the literature.^67^

#### Methyl 5,5,8,8-Tetramethyl-5,6,7,8-tetrahydroquinoxaline-2-carboxylate, **8**

Compound **7** (0.80 g, 4.76 mmol) and d l-2,3-diaminopropionic acid hydrochloride (0.67 g, 4.76 mmol) were combined in MeOH (30 mL). NaOH (0.76 g, 19.04 mmol) was added and the resultant mixture was stirred at reflux for 24 h. The solution was then cooled to 0 °C, H_2_SO_4_ carefully added, and the solution was stirred at reflux for a further 6 h. The solution was cooled, and the solvent evaporated to give a crude residue which was dissolved with EtOAc, washed with sat. NaHCO_3_, H_2_O, and brine, dried (MgSO_4_), and evaporated to give a crude yellow oil (0.9 g). This was purified by SiO_2_ chromatography (95:5, heptane/EtOAc) to give compound **8** as a colorless oil (0.633 g, 54%): ^1^H NMR (400 MHz, CDCl_3_) δ 1.33 and 1.36 (s, 12H), 1.81 (s, 4H), 3.98 (s, 3H), 9.00 (s, 1H). All other data matched the literature.^23^

#### 5,5,8,8-Tetramethyl-5,6,7,8-tetrahydroquinoxaline-2-carbaldehyde, **9**

To a solution of compound **8** (5.09 g, 20.5 mmol) in THF (80 mL) was added NaBH_4_ (2.33 g, 61.5 mmol). The solution was then heated to reflux, whereupon MeOH (16 mL) was slowly added over 1 h. The resultant solution was then stirred at reflux overnight. The solution was cooled, quenched with 1 M HCl, and the solvent then evaporated. The residue was dissolved in DCM, washed with water, dried (MgSO_4_), and evaporated to give a crude yellow oil (4 g). This was purified by SiO_2_ chromatography (8:2, heptane/EtOAc, as eluent) to give alcohol **33** as a colorless oil (3.96 g, 88%): ^1^H NMR (400 MHz, CDCl_3_) δ 1.33 and 1.36 (s, 12H), 1.80 (s, 4H), 3.50 (br, 1H), 4.75 (s, 2H), 8.32 (s, 1H).^22^ Oxalyl chloride (2.28 mL, 26.96 mmol) was added to anhydrous DCM (100 mL) under N_2_. The resultant solution was cooled to −78 °C, whereupon DMSO (3.83 mL, 53.92 mmol) was added dropwise so as to maintain the temperature below −60 °C. The solution was stirred for 15 min before DC614, as a solution in anhydrous DCM (3.96 g, 17.97 mmol, in 20 mL) was added dropwise so as to main the temperature below −60 °C. The solution was stirred for a further 15 min before Et_3_N (18.03 mL, 129.38 mmol) was added. The solution was then stirred for 10 min, before being allowed to reach RT over 30 min. H_2_O was added, and the resultant mixture was diluted with DCM, washed with H_2_O, dried (MgSO_4_), and evaporated to give a crude oil (4 g). This was purified by DCVC (heptane to 9:1, heptane/EtOAc) to give compound **9** as a colorless oil that slowly crystallizes (3.39 g, 86%): ^1^H NMR (700 MHz, CDCl_3_) δ 1.35 and 1.37 (s, 12H), 1.83 (s, 4H), 8.90 (s, 1H), 10.08 (s, 1H); ^13^C NMR (176 MHz, CDCl_3_) δ 29.7, 29.7, 33.8, 33.8, 37.4, 37.9, 139.8, 144.2, 159.0, 163.7, 193.4; IR (ATR) *v*_max_/cm^–1^ 2979m, 2964m, 2928m, 2862m, 2823w, 1707s, 1553m, 1457m, 1126s, 1078s, 737s; MS­(ES): *m*/*z* = 219.3 [M + H]^+^; HRMS (ES) calcd. for C_13_H_19_ON_2_ [M + H]^+^: 216.1497, found 216.1503.^23^

#### 2-Ethynyl-5,5,8,8-tetramethyl-5,6,7,8-tetrahydroquinoxaline, **10**

To a solution of compound **9** (1.18 g, 5.40 mmol) in anhydrous MeOH (50 mL) under N_2_ was added K_2_CO_3_ (1.49 g, 10.80 mmol) and dimethyl-1-diazo-2-oxopropylphosphonate (0.98 mL, 6.48 mmol), and the resultant suspension was stirred at RT for 16 h. The solution was diluted with EtOAc, washed with 5% NaHCO_3_, H_2_O, and brine, dried (MgSO_4_), and evaporated to give a crude orange oil (0.4 g). This was purified by SiO_2_ chromatography (95:5, heptane/EtOAc, as eluent) to give compound **10** as a colorless oil that slowly crystallized (0.84 g, 73%): ^1^H NMR (700 MHz, CDCl_3_) δ 1.30 and 1.31 (s, 12H), 1.77 (s, 4H), 3.22 (s, 1H), 8.45 (s, 1H); ^13^C NMR (176 MHz, Chloroform-*d*) δ 29.6, 29.7, 33.8, 34.0, 37.2, 37.3, 79.0, 81.0, 135.4, 144.5, 158.1, 158.6; IR (ATR) *v*_max_/cm^–1^ 3279s, 2986w, 2945m, 2917m, 2863w, 2110w, 1519w, 1470m, 1459m,1274m, 1078s, 674s; MS­(ES): *m*/*z* = 215.3 [M + H]^+^; HRMS (ES) calcd. for C_14_H_19_N_2_ [M + H]^+^: 215.1548, found 215.1548.

#### Methyl 4-Bromo-2-fluorobenzoate, **11b**

4-Bromo-2-fluorobenzoic acid (25.0 g, 114.2 mmol) was suspended in MeOH (250 mL), whereupon conc. H_2_SO_4_ (4 mL) was added and the resultant solution was stirred at reflux overnight. The clear solution was then cooled, and H_2_O (100 mL) was added, whereupon a white precipitate formed. This was filtered, washed with H_2_O and dried to give a crude white solid. This was recrystallized from heptane to give compound **11b** as a colorless crystalline solid (21.34 g, 80%): ^1^H NMR (600 MHz, CDCl_3_) δ 3.91 (s, 3H), 7.31 – 7.36 (m, 2H), 7.80 (t, *J* = 8.0 Hz, 1H); ^13^C NMR (151 MHz, CDCl_3_) δ 52.4, 117.6 (d, *J* = 9.9 Hz), 120.6 (d, *J* = 25.6 Hz), 127.5 (d, *J* = 3.9 Hz), 127.9 (d, *J* = 9.6 Hz), 133.1, 161.56 (d, *J* = 264.9 Hz), 164.1 (d, *J* = 3.9 Hz); ^19^F NMR (376 MHz, CDCl_3_) δ −106.6; IR (ATR) *v*_max_/cm^–1^ 3104w, 3086w, 2961w, 1712s, 1599s, 1571m, 1403m, 1215s, 882s; MS (ASAP): *m*/*z* = 233.0, 235.0 [M + H]^+^; HRMS (ASAP) calcd. for C_8_H_7_O_2_BrF [M + H]^+^: 232.9613, found: 232.9621.

#### Methyl 4-Bromo-3-fluorobenzoate, **11c**

4-Bromo-3-fluorobenzoic acid (20.0 g, 91.32 mmol) was suspended in MeOH (100 mL), whereupon conc. H_2_SO_4_ (3 mL) was added and the resultant solution was stirred at reflux overnight. The mixture was cooled, then the solvent evaporated to give a crude residue. This was dissolved in EtOAc, and the organics were washed with sat. NaHCO_3_, H_2_O, and brine, dried (MgSO_4_), and evaporated to give a crude brown oil (20 g). This was purified by recrystallization from heptane to give compound **11c** as a colorless crystalline solid (19.3 g, 91%): ^1^H NMR (700 MHz, CDCl_3_) δ 3.92 (s, 3H), 7.62 (dd, *J* = 8.3, 6.6 Hz, 1H), 7.69 (dd, *J* = 8.3, 1.9 Hz, 1H), 7.75 (dd, *J* = 8.9, 1.9 Hz, 1H); ^13^C NMR (176 MHz, CDCl_3_) δ 52.5, 114.8 (d, *J* = 21.0 Hz), 117.4 (d, *J* = 24.0 Hz), 126.2 (d, *J* = 3.7 Hz), 131.3 (d, *J* = 6.7 Hz), 133.7, 158.9 (d, *J* = 248.6 Hz), 165.3 (d, *J* = 2.7 Hz); ^19^F NMR (376 MHz, CDCl_3_) δ −106.0; IR (ATR) *v*_max_/cm^–1^ 3004w, 2955w, 1717s, 1573m, 1439m, 1297s, 1213s, 1097s, 759s; MS (ASAP): *m*/*z* = 233.0, 235.0 [M + H]^+^; HRMS (ASAP) calcd. for C_8_H_7_O_2_BrF [M + H]^+^: 232.9613, found: 232.9618.

#### Methyl 4-Bromo-2,6-difluorobenzoate, **11d**

4-Bromo-2,6-difluorobenzoic acid (15.0 g, 63.29 mmol) was suspended in MeOH (100 mL), whereupon conc. H_2_SO_4_ (5 mL) was added and the resultant solution was stirred at reflux for 16 h. The clear solution was then cooled, and the solvent evaporated to give a crude residue. This was dissolved with EtOAc, and washed with sat. NaHCO_3_, H_2_O, and brine, dried (MgSO_4_), and evaporated to give a crude white solid (16 g). This was purified by dry column vacuum chromatography (100% heptane to 8:2, heptane/EtOAc) to give compound **11d** as a colorless oil that slowly crystallizes (14.87 g, 94%): ^1^H NMR (700 MHz, CDCl_3_) δ 3.93 (s, 3H), 7.12–7.17 (m, 2H); ^13^C NMR (176 MHz, CDCl_3_) δ 52.8, 110.1 (t, *J* = 17.8 Hz), 116.0 (d, *J* = 4.3 Hz), 116.2 (d, *J* = 4.3 Hz), 125.5 (t, *J* = 12.2 Hz), 160.6 (dd, *J* = 260.9, 7.1 Hz), 161.2 (t, *J* = 1.6 Hz); ^19^F NMR (376 MHz, CDCl_3_) δ −108.2; IR (ATR) *v*_max_/cm^–1^ 3090w, 2968m, 1732s, 1600m, 1512m, 1416s, 1262m, 1047s, 854s; MS­(ASAP): *m*/*z* = 250.9, 252.9 [M + H]^+^; HRMS (ASAP) calcd. for C_8_H_6_O_2_BrF_2_ [M + H]^+^: 250.9519, found 250.9512.

#### Methyl 4-Bromo-3-chlorobenzoate, **11e**

4-Bromo-3-chlorobenzoic acid (20.0 g, 84.94 mmol) was suspended in MeOH (100 mL), whereupon conc. H_2_SO_4_ (3 mL) was added and the resultant solution was stirred at reflux overnight. The mixture was cooled, then the solvent evaporated to give a crude residue. This was dissolved in EtOAc, and the organics were washed with sat. NaHCO_3_, H_2_O, and brine, dried (MgSO_4_), and evaporated to give crude brown oil (19 g). This was purified by recrystallization from heptane to give compound **11e** as a colorless crystalline solid (17.7 g, 84%): ^1^H NMR (400 MHz, CDCl_3_) δ 3.91 (s, 3H), 7.68 (d, *J* = 8.3 Hz, 1H), 7.74 (dd, *J* = 8.3, 2.0 Hz, 1H), 8.08 (d, *J* = 2.0 Hz, 1H); ^13^C NMR (101 MHz, CDCl_3_) δ 52.5, 127.9, 128.6, 130.6, 131.2, 133.8, 134.9, 165.2; IR (ATR) *v*_max_/cm^–1^ 3007w, 2955w, 2849w, 1714s, 1585w, 1437m, 1291s, 1272s, 1103s, 754s; MS (ASAP): *m*/*z* = 248.9, 250.9 [M + H]^+^; HRMS (ASAP) calcd. for C_8_H_7_O_2_ClBr [M + H]^+^: 248.9318, found: 248.9317.

#### Methyl 5-Bromopyridine-2-carboxylate, **11f**

5-Bromopyridine-2-carboxylic acid (20.0 g, 99.0 mmol) was suspended in MeOH (150 mL), whereupon conc. H_2_SO_4_ (5 mL) was carefully added and the resultant solution was stirred at reflux for 6 h. The solution was cooled, diluted with EtOAc, washed with H_2_O and brine (50 mL), dried (MgSO_4_), and evaporated to give a crude colorless solid. This was purified by Kugelrohr distillation (200 °C, 7.4 Torr), and the resultant white solid was further recrystallized from heptane/MeOH (10:1) to give compound **11f** as a white solid (17.21 g, 80%): ^1^H NMR (700 MHz, CDCl_3_) δ 3.90 (s, 4H), 7.86 – 7.93 (m, 2H), 8.68 (d, *J* = 2.0 Hz, 1H); ^13^C NMR (176 MHz, CDCl_3_) δ 52.8, 124.8, 126.0, 139.5, 146.0, 150.7, 164.7; *v*_max_/cm^–1^ 3059w, 3008w, 2957w, 1710s, 1571w, 1558w, 1436m, 1305s, 1131s, 696s; MS (ASAP): *m*/*z* = 216.0, 218.0 [M + H]^+^; HRMS (ASAP) calcd. for C_7_H_7_NO_2_Br [M + H]^+^: 215.9660, found: 215.9664.

#### Methyl 6-Bromopyridine-3-carboxylate, **11g**

A solution of 6-bromopyridine-3-carboxylic acid (3.0 g, 14.85 mmol) in anhydrous DMF (50 mL) was cooled to 0 °C under N_2_, whereupon K_2_CO_3_ (5.13 g, 37.13 mmol) was added, and the resultant slurry was stirred for 1 h at RT. Iodomethane (2.31 mL, 37.13 mmol) was added, and the solution was stirred at RT for 16 h. The resultant solution was diluted with H_2_O and extracted with EtOAc (3×). The organics were washed with H_2_O and brine, dried (MgSO_4_), and evaporated to give a crude yellow solid (5.5 g). This was purified by SiO_2_ chromatography (7:3, heptane/EtOAc) to give compound **11g** as a white solid (2.83 g, 88%): ^1^H NMR (700 MHz, CDCl_3_) δ 3.95 (s, 3H), 7.58 (dd, *J* = 8.3, 0.6 Hz, 1H), 8.12 (dd, *J* = 8.3, 2.4 Hz, 1H), 8.95 (d, *J* = 2.4 Hz, 1H); ^13^C NMR (176 MHz, CDCl_3_) δ 52.6, 125.3, 128.0, 139.1, 146.8, 151.4, 165.0; IR (ATR) *v*_max_/cm^–1^ 3063w, 2957w, 2853w, 1714s, 1580s, 1436m, 1275s, 1117s, 762s; MS­(ASAP): *m*/*z* = 216.0, 218.00 [M + H]^+^; HRMS (ASAP) calcd. for C_7_H_7_NO_2_Br [M + H]^+^: 215.9660, found 215.9656.

#### Methyl 5-Chloropyrazine-2-carboxylate, **11h**

A solution of 5-chloropyrazine-2-carboxylic acid (3.0 g, 18.92 mmol) was reacted in anhydrous DMF (50 mL) with K_2_CO_3_ (6.54 g, 47.31 mmol) and iodomethane (2.95 mL, 47.31 mmol) according to Method C to give compound **11h** as a white solid (2.04 g, 62%): ^1^H NMR (700 MHz, CDCl_3_) δ 4.01 (s, 3H), 8.66 (d, *J* = 1.3 Hz, 1H), 9.05 (d, *J* = 1.3 Hz, 1H); ^13^C NMR (176 MHz, CDCl_3_) δ 53.2, 141.2, 144.3, 145.6, 152.6, 163.5; IR (ATR) *v*_max_/cm^–1^ 3076w, 3043w, 3004w, 2952w, 2847w, 1718s, 1560m, 1520m, 1432m, 1283s, 1142s, 798m; MS­(ASAP): *m*/*z* = 173.0 [M + H]^+^; HRMS (ASAP) calcd. for C_6_H_6_N_2_O_2_Cl [M + H]^+^: 173.0118, found 173.0113.

#### Methyl 4-Ethynyl-3-fluorobenzoate, **12a**

Et_3_N (120 mL) was degassed by sparging with N_2_ for 1 h. **11c** (5.0 g, 21.45 mmol), trimethylsilylacetylene (3.56 mL, 25.74 mmol), Pd­(PPh_3_)_2_Cl_2_ (301 mg, 0.43 mmol) and CuI (82 mg, 0.43 mmol) were then added under N_2_ and the resultant suspension was stirred at RT for 16 h. The suspension was diluted with heptane and passed through Celite/SiO_2_ and the extracts were evaporated to give a crude oil (6.44 g). This was purified by dry column vacuum chromatography (100% heptane, to 9:1, heptane/EtOAc), and the isolated product further purified by Kugelrohr distillation (150 °C, 7.4 Torr) to give the intermediate-protected alkyne as a yellow oil (5.58 g, >100%). This was dissolved in a MeOH/MTBE solution (5:50, 55 mL), K_2_CO_3_ (6.16 g, 44.6 mmol) was added, and the resultant mixture was stirred under N_2_ for 6 h at RT. The solution was then diluted with EtOAc, washed with sat. NH_4_Cl, H_2_O, and brine, dried (MgSO_4_), and evaporated to give a crude solid (3.6 g). This was purified by dry column vacuum chromatography (100% heptane, to 8:2, heptane/EtOAc) to give **12a** as a white solid (3.07 g, 77%): ^1^H NMR (600 MHz, CDCl_3_) δ 3.45 (s, 1H), 3.92 (s, 3H), 7.53 (dd, *J* = 8.0, 6.8 Hz, 1H), 7.72 (dd, *J* = 9.6, 1.6 Hz, 1H), 7.77 (dd, *J* = 8.0, 1.6 Hz, 1H); ^13^C NMR (151 MHz, CDCl_3_) δ 52.5, 76.3, 85.1 (d, *J* = 3.3 Hz), 115.3 (d, *J* = 16.0 Hz), 116.5 (d, *J* = 22.9 Hz), 124.9 (d, *J* = 3.7 Hz), 132.2 (d, *J* = 7.4 Hz), 133.9 (d, *J* = 1.3 Hz), 162.9 (d, *J* = 253.5 Hz), 165.3 (d, *J* = 2.7 Hz); ^19^F NMR (376 MHz, CDCl_3_) δ −109.3; IR (ATR) *v*_max_/cm^–1^ 3238m, 3090w, 2967w, 2111w, 1710s, 1564m, 1501m, 1440m, 1308s, 1212s, 766s; MS (ASAP) *m*/*z* = 179.0 [M + H]^+^; HRMS (ASAP) calcd. for C_10_H_8_O_2_F [M + H]^+^: 179.0508, found 179.0495.

#### Methyl 4-Ethynyl-2,6-difluorobenzoate, **12b**

Et_3_N (100 mL) was degassed by sparging with N_2_ for 1 h. **11d** (4.0 g, 15.93 mmol), trimethylsilylacetylene (2.65 mL, 19.12 mmol), Pd­(PPh_3_)_2_Cl_2_ (224 mg, 0.32 mmol) and CuI (61 mg, 0.32 mmol) were then added under N_2_ and the resultant suspension was stirred at RT for 16 h. The suspension was diluted with heptane and passed through Celite/SiO_2_ and the extracts were evaporated to give a crude oil (5 g). This was purified by dry column vacuum chromatography (100% heptane to 8:2, heptane/EtOAc) to give the intermediate-protected alkyne as a light yellow oil (4.31 g, >100%). This was dissolved in a MeOH/MTBE solution (1:5, 60 mL), K_2_CO_3_ (4.44 g, 32.1 mmol) was added, and the resultant mixture was stirred under N_2_ for 4 h at RT. The solution was then diluted with EtOAc, washed with sat. NH_4_Cl, H_2_O, and brine, dried (MgSO_4_), and evaporated to give a crude oil (3 g). This was purified by dry column vacuum chromatography (100% heptane, to 9:1, heptane/EtOAc) to give compound **12b** as a white solid (2.70 g, 86% over two steps): ^1^H NMR (700 MHz, CDCl_3_) δ 3.26 (s, 1H), 3.94 (s, 3H), 7.02–7.08 (m, 2H); ^13^C NMR (176 MHz, CDCl_3_) δ 52.9, 80.5, 80.5, 80.5, 81.5, 111.5, 111.6, 111.7, 115.6, 115.7, 115.8, 115.8, 126.9, 126.9, 127.0, 159.6, 159.6, 161.0, 161.1, 161.4, 161.4, 161.4; ^19^F NMR (376 MHz, CDCl_3_) δ −109.8; IR (ATR) *v*_max_/cm^–1^ 3289m, 3085w, 2972w, 2121w, 1732s, 1627s, 1555m, 1416m, 13940m, 1272s, 1046s, 858s, 687s; MS (ASAP) *m*/*z* = 197.0 [M + H]^+^; HRMS (ASAP) calcd. for C_10_H_7_O_2_F_2_ [M + H]^+^: 197.0414, found 197.0404.

#### Methyl 4-Ethynyl-3-chlorobenzoate, **12c**

Et_3_N (120 mL) was degassed by sparging with N_2_ for 1 h. **11e** (5.0 g, 20.04 mmol), trimethylsilylacetylene (3.33 mL, 24.05 mmol), Pd­(PPh_3_)_2_Cl_2_ (286 mg, 0.408 mmol), and CuI (78 mg, 0.408 mmol) were then added under N_2_ and the resultant suspension was stirred at RT for 16 h. The suspension was diluted with heptane and passed through Celite/SiO_2_ and the extracts were evaporated to give a crude oil (5.2 g). This was purified by Kugelrohr distillation (150–160 °C, 7.3 Torr) to give in the intermediate-protected alkyne as a clear oil (5.07 g, 95%). This was dissolved in a MeOH/MTBE solution (5:50, 55 mL), K_2_CO_3_ (5.25 g, 38.0 mmol) was added, and the resultant mixture was stirred under N_2_ for 6 h at RT. The solution was then diluted with EtOAc, washed with sat. NH_4_Cl, H_2_O, and brine, dried (MgSO_4_), and evaporated to give a crude solid (3.3 g). This was purified by dry column vacuum chromatography (100% heptane, to 9:1, heptane/EtOAc), and the isolated product was further recrystallized from heptane to give compound **12c** as a white solid (2.43 g, 66%): ^1^H NMR (400 MHz, CDCl_3_) δ 3.53 (s, 1H), 3.92 (s, 3H), 7.59 (d, *J* = 8.1 Hz, 1H), 7.87 (dd, *J* = 8.1, 1.6 Hz, 1H), 8.07 (dd, *J* = 1.6, 0.3 Hz, 1H).

#### 2-Fluoro-4-[2-(5,5,8,8-tetramethyl-5,6,7,8-tetrahydronaphthalen-2-yl)­ethynyl]­benzoic Acid, **13**

Et_3_N/THF (1:1, 120 mL) was added to an oven-dried 100 mL RBF under N_2_, and the solution was degassed by sparging with N_2_ for 1 h. Pd­(PPh_3_)_2_Cl_2_(0.260 g, 0.37 mmol), CuI (0.070 g, 0.37 mmol), **4a** (1.02 g, 4.80 mmol) and **11b** (0.86 g, 3.69 mmol) were added under N_2_ and the resultant solution was stirred at 50 °C for 40 h. The solution was diluted with heptane, eluted through a Celite/SiO_2_ plug and the extracts were evaporated to give a crude brown solid (1.7 g). This was purified by dry column vacuum chromatography (100% heptane to heptane/EtOAc, 9:1), and further recrystallized from MeOH to give the intermediate ester as a white solid (0.63 g, 47%). The ester (0.60 g, 1.65 mmol) was dissolved in THF (30 mL), 20% NaOH (3 mL) was added, and the resultant solution was stirred at reflux for 16 h. The mixture was cooled, acidified to pH 1 with 5% HCl, extracted with EtOAc, washed with H_2_O and brine, dried (MgSO_4_), and evaporated to give a crude white solid. This was recrystallized from MeCN to give compound **13** as a colorless crystalline solid (0.52 g, 90%): ^1^H NMR (700 MHz, DMSO-*d*_6_) δ 1.24 and 1.25 (s, 12H), 1.64 (s, 4H), 7.31 (dd, *J* = 8.2, 1.8 Hz, 1H), 7.39 (d, *J* = 8.2 Hz, 1H), 7.45 (dd, *J* = 8.0, 1.5 Hz, 1H), 7.50 (dd, *J* = 11.3, 1.5 Hz, 1H), 7.54 (d, *J* = 1.8 Hz, 1H), 7.88 (t, *J* = 8.0 Hz, 1H), 13.39 (br, 1H); ^13^C NMR (176 MHz, DMSO-*d*_6_) δ 31.3, 31.4, 33.9, 34.1, 34.2, 34.3, 86.5 (d, *J* = 2.7 Hz), 93.4, 118.5, 119.1 (d, *J* = 10.5 Hz), 119.4 (d, *J* = 24.2 Hz), 127.0, 127.3 (d, *J* = 3.6 Hz), 128.6 (d, *J* = 10.4 Hz), 128.7, 129.8, 132.3 (d, *J* = 1.9 Hz), 145.1, 146.3, 160.8 (d, *J* = 257.9 Hz), 164.5 (d, *J* = 3.2 Hz); ^19^F NMR (376 MHz, DMSO-*d*_6_) δ −110.1; IR (ATR) *v*_max_/cm^–1^ 2956m, 2928m, 2858m, 2209m, 1677s, 1614s, 1556m, 1440m, 1300m, 835s; MS­(ASAP): *m*/*z* = 351.2 [M + H]^+^; HRMS (ASAP) calcd. for C_23_H_24_O_2_F [M + H]^+^: 351.1760, found 351.1765.

#### 3-Fluoro-4-[2-(5,5,8,8-tetramethyl-5,6,7,8-tetrahydronaphthalen-2-yl)­ethynyl]­benzoic Acid, **14**

Et_3_N (80 mL) was degassed by sparging with N_2_ for 1 h. **3a** (0.80 g, 2.55 mmol), **12a** (0.54 g, 3.05 mmol), Pd­(PPh_3_)_2_Cl_2_ (179 mg, 0.26 mmol) and CuI (49 mg, 0.26 mmol) were then added under N_2_ and the resultant suspension was stirred at RT for 72 h. The suspension was diluted with MTBE and passed through Celite/SiO_2_ and the extracts were evaporated to give a crude solid (1.1 g). This was purified by dry column vacuum chromatography (100% heptane to heptane/EtOAc, 95:5) to give the intermediate ester as a colorless oil which slowly crystallized (0.82 g, 88%). This was dissolved in THF (40 mL), 20% NaOH (3 mL) was added, and the resultant solution was stirred at reflux for 16 h. The mixture was cooled, acidified to pH 1 with 5% HCl, extracted with EtOAc, washed with H_2_O and brine, dried (MgSO_4_), and evaporated to give a crude white solid which was recrystallized from MeCN to give compound **14** as a colorless crystalline solid (0.60 g, 77%): ^1^H NMR (600 MHz, DMSO-*d*_6_) 1.24 and 1.26 (s, 12H), 1.64 (s, 4H), 7.32 (dd, *J* = 8.2, 1.8 Hz, 1H), 7.40 (d, *J* = 8.2 Hz, 1H), 7.53 (d, *J* = 1.8 Hz, 1H), 7.70–7.78 (m, 2H), 7.80 (dd, *J* = 7.9, 1.6 Hz, 1H), 13.44 (br, 1H); ^13^C NMR (151 MHz, DMSO-*d*_6_) δ 31.3, 31.4, 33.9, 34.1, 34.2, 34.3, 81.0, 97.4 (d, *J* = 3.1 Hz), 115.2, 115.3 (d, *J* = 15.8 Hz), 116.0 (d, *J* = 22.3 Hz), 118.5, 125.4 (d, *J* = 3.4 Hz), 127.1, 128.7, 129.6, 132.7 (d, *J* = 7.1 Hz), 133.6, 145.2, 146.5, 161.4 (d, *J* = 250.3 Hz), 165.7 (d, *J* = 2.5 Hz); ^19^F NMR (376 MHz, DMSO-*d*_6_) δ −110.0; IR (ATR) *v*_max_/cm^–1^ 2967m, 2928m, 2857m, 2210w, 1686s, 1617m, 1566m, 1421m, 1307m, 1218m, 834s, 764m; MS­(ASAP): *m*/*z* = 351.2 [M + H]^+^; HRMS (ASAP) calcd. for C_23_H_24_O_2_F [M + H]^+^: 351.1760, found 351.1766.

#### 2,6-Difluoro-4-[2-(5,5,8,8-tetramethyl-5,6,7,8-tetrahydronaphthalen-2-yl)­ethynyl]­benzoic Acid, **15**

Et_3_N (80 mL) was added to an oven-dried 100 mL RBF under N_2_, and the solution was degassed by sparging with N_2_ for 1 h. Pd­(PPh_3_)_2_Cl_2_ (223 mg, 0.32 mmol), CuI (61 mg, 0.32 mmol), **3a** (1.0 g, 3.18 mmol) and **12b** (0.79 g, 4.00 mmol) were added under N_2_ and the resultant solution was stirred at RT for 72 h. The solution was diluted with heptane, eluted through a Celite/SiO_2_ plug, and the extracts were evaporated to give a crude orange solid (1.25 g). This was purified by dry column vacuum chromatography (100% heptane to heptane/EtOAc, 9:1) to give the intermediate ester as a colorless oil that slowly solidifed (0.97 g, 82%). This was dissolved in THF (30 mL), 20% NaOH (3 mL) was added, and the resultant solution was stirred at reflux for 16 h. The mixture was cooled, acidified to pH 1 with 5% HCl, extracted with EtOAc, washed with H_2_O and brine, dried (MgSO_4_), and evaporated to give a crude white solid which was recrystallized from MeCN to give compound **15** as a colorless crystalline solid (0.68 g, 77%): ^1^H NMR (700 MHz, DMSO-*d*_6_) δ 1.23 and 1.24 (s, 12H), 1.63 (s, 4H), 7.30 (dd, *J* = 8.2, 1.9 Hz, 1H), 7.38 (d, *J* = 8.2 Hz, 1H), 7.39 – 7.43 (m, 2H), 7.54 (d, *J* = 1.9 Hz, 1H); ^13^C NMR (176 MHz, DMSO-*d*_6_) δ 31.3, 31.4, 33.9, 34.1, 34.2, 34.3, 85.7 (t, *J* = 3.6 Hz), 93.7, 112.2 (t, *J* = 20.1 Hz), 115.0 (d, *J* = 4.3 Hz), 115.1 (d, *J* = 4.5 Hz), 118.3, 126.9 (t, *J* = 12.6 Hz), 127.0, 128.7, 129.9, 145.1, 146.5, 159.3 (dd, *J* = 252.6, 8.3 Hz), 161.7; ^19^F NMR (376 MHz, DMSO-*d*_6_) δ −111.6; IR (ATR) *v*_max_/cm^–1^ 2962m, 2915w, 2870w, 2204m, 1694s, 1620s, 1552m, 1424m, 1273s, 1049m, 855s; MS­(ASAP): *m*/*z* = 369.2 [M + H]^+^; HRMS (ASAP) calcd. for C_23_H_23_O_2_F_2_ [M + H]^+^: 369.1666, found 369.1672.

#### 3-Chloro-4-[2-(5,5,8,8-tetramethyl-5,6,7,8-tetrahydronaphthalen-2-yl)­ethynyl]­benzoic Acid, **16**

Et_3_N (90 mL) was degassed by sparging with N_2_ for 1 h. **3a** (0.83 g, 2.64 mmol), **12c** (0.62 g, 3.16 mmol), Pd­(PPh_3_)_2_Cl_2_ (185 mg, 0.26 mmol) and CuI (50 mg, 0.26 mmol) were then added under N_2_ and the resultant suspension was stirred at RT for 40 h. The suspension was diluted with heptane, passed through a Celite/SiO_2_ plug and the extracts were evaporated to give a crude solid (0.6 g). This was purified by dry column vacuum chromatography (100% heptane to heptane/EtOAc, 9:1) to give the intermediate ester as a colorless oil which slowly crystallized (0.79 g, 79%). The ester (0.55 g, 1.44 mmol) was dissolved in THF (30 mL), 20% NaOH (3 mL) was added, and the resultant solution was stirred at reflux for 16 h. The mixture was cooled, acidified to pH 1 with 5% HCl, extracted with EtOAc, washed with H_2_O and brine, dried (MgSO_4_), and evaporated to give a crude white solid which was recrystallized from MeCN to give compound **16** as a colorless solid (0.40 g, 76%): ^1^H NMR (600 MHz, DMSO-*d*_6_) δ ^1^H NMR (600 MHz, DMSO-*d*_6_) δ 1.24 (s, 6H), 1.25 (s, 6H), 1.64 (s, 4H), 7.33 (dd, *J* = 8.1, 1.8 Hz, 1H), 7.40 (d, *J* = 8.1 Hz, 1H), 7.53 (d, *J* = 1.8 Hz, 1H), 7.78 (d, *J* = 8.1 Hz, 1H), 7.90 (dd, *J* = 8.1, 1.6 Hz, 1H), 8.01 (d, *J* = 1.6 Hz, 1H), 13.46 (s, 1H); ^13^C NMR (151 MHz, DMSO-*d*_6_) δ 31.3, 31.4, 33.9, 34.1, 34.2, 34.3, 84.6, 97.7, 118.5, 126.3, 127.1, 127.9, 128.7, 129.6, 129.6, 131.9, 133.5, 134.6, 145.1, 146.5, 165.6 IR (ATR) *v*_max_/cm^–1^ 2960m, 2924m, 2857m, 2206m, 1684s, 1594m, 1544m, 1423m, 1306s 1243m, 828s, 767s; MS­(ASAP): *m*/*z* = 367.2 [M + H]^+^; HRMS (ASAP) calcd. for C_23_H_24_O_2_Cl [M + H]^+^: 367.1465, found 367.1449.

#### 2-Fluoro-4-[2-(3,5,5,8,8-pentamethyl-5,6,7,8-tetrahydronaphthalen-2-yl)­ethynyl]­benzoic Acid, **17**

Et_3_N (25 mL) was degassed by sparging with Ar for 1 h. **11b** (0.26 g, 1.13 mmol), **4b** (0.3 g, 1.33 mmol), Pd­(PPh_3_)_2_Cl_2_ (79 mg, 0.11 mmol) and CuI (21 mg, 0.11 mmol) were then added under Ar and the resultant suspension was stirred at RT for 72 h. The suspension was diluted with MTBE and passed through Celite/SiO_2_ and the extracts were evaporated to give a crude solid. This was purified by dry column vacuum chromatography (100% heptane to heptane/EtOAc, 95:5), and further recrystallized from MeOH to give the intermediate ester as an off-white solid (0.34 g, 78%). This was dissolved in THF (30 mL), 20% NaOH (3 mL) was added, and the resultant solution was stirred at reflux for 72 h. The mixture was cooled, acidified to pH 1 with 5% HCl, extracted with EtOAc, washed with H_2_O and brine, dried (MgSO_4_), and evaporated to give a crude white solid. This was purified by SiO_2_ chromatography (95:5, DCM/MeOH with 0.5% AcOH), and further recrystallized from MeCN to give compound **17** as a white solid (0.76 g, 76%): ^1^H NMR (700 MHz, DMSO-*d*_6_) δ 1.24 (s, 12H), 1.63 (s, 4H), 2.40 (s, 3H), 7.28 (s, 1H), 7.46 (dd, *J* = 8.0, 1.5 Hz, 1H), 7.47 (s, 1H), 7.51 (dd, *J* = 11.3, 1.5 Hz, 1H), 7.89 (t, *J* = 8.0 Hz, 1H), 13.38 (br, 1H); ^13^C NMR (176 MHz, DMSO-*d*_6_) δ 19.9, 31.3, 31.4, 33.5, 34.0, 34.4, 34.4, 90.2 (d, *J* = 2.4 Hz), 92.4, 118.6, 119.2 (d, *J* = 24.2 Hz), 127.2 (d, *J* = 3.5 Hz), 127.7, 128.7 (d, *J* = 10.0 Hz), 129.9, 132.3 (d, *J* = 1.9 Hz), 136.8, 142.3, 146.3, 160.9 (d, *J* = 257.9 Hz), 164.4 (d, *J* = 3.3 Hz); ^19^F NMR (376 MHz, DMSO-*d*_6_) δ −110.14; IR (ATR) *v*_max_/cm^–1^ 2956m, 2906m, 2856w, 2209m, 1694s, 1614s, 1557m, 1438m, 1408m, 1295s, 1274s, 1226m, 1047s, 867s, 774s; MS­(ES): *m*/*z* = 365.2 [M + H]^+^; HRMS (ES) calcd. for C_24_H_26_O_2_F [M + H]^+^: 365.1917, found 365.1908.

#### 3-Fluoro-4-[2-(3,5,5,8,8-pentamethyl-5,6,7,8-tetrahydronaphthalen-2-yl)­ethynyl]­benzoic Acid, **18**

Et_3_N (80 mL) was degassed by sparging with N_2_ for 1 h. Compound **3b** (0.8 g, 2.44 mmol), **12a** (0.52 g, 2.92 mmol), Pd­(PPh_3_)_2_Cl_2_ (171 mg, 0.24 mmol) and CuI (46 mg, 0.24 mmol) were then added under N_2_ and the resultant suspension was stirred at RT for 72 h. The suspension was diluted with heptane and passed through Celite/SiO_2_ and the extracts were evaporated to give a crude solid (1.2 g). This was purified by dry column vacuum chromatography (100% heptane to heptane/EtOAc, 9:1) to give the intermediate ester as a crystalline white solid (0.17 g, 18%). This was dissolved in THF (30 mL), 20% NaOH (3 mL) was added, and the resultant solution was stirred at reflux for 16 h. The mixture was cooled, acidified to pH 1 with 5% HCl, extracted with EtOAc, washed with H_2_O and brine, dried (MgSO_4_), and evaporated to give a crude white solid which was recrystallized from MeCN to give compound **18** as colorless solid (0.13 g, 78%): ^1^H NMR (600 MHz, DMSO-*d*_6_)­1.24 (s, 12H), 1.63 (s, 4H), 2.40 (s, 3H), 7.30 (s, 1H), 7.46 (s, 1H), 7.72 – 7.78 (m, 2H), 7.80 (dd, J = 7.9, 1.6 Hz, 1H), 13.44 (s, 1H); ^13^C NMR (151 MHz, DMSO-*d*_6_) δ 19.8, 31.3, 31.4, 33.5, 34.0, 34.4, 34.4, 84.8, 96.5 (d, *J* = 3.1 Hz), 115.5 (d, *J* = 15.9 Hz), 116.0 (d, *J* = 22.3 Hz), 118.6, 125.5 (d, *J* = 3.4 Hz), 127.7, 129.7, 132.6 (d, *J* = 7.1 Hz), 133.4, 136.8, 142.4, 146.5, 161.3 (d, *J* = 250.2 Hz), 165.7 (d, *J* = 2.3 Hz); ^19^F NMR (376 MHz, DMSO-*d*_6_) δ −110.1**;** IR (ATR) *v*_max_/cm^–1^ 2960m, 2926m, 2858m, 2212m, 1690s, 1616m, 1565m, 1490m, 1428m, 1242m, 1223m, 890m, 763s; MS­(ASAP): *m*/*z* = 365.2 [M + H]^+^; HRMS (ASAP) calcd. for C_24_H_26_O_2_F [M + H]^+^: 365.1917, found 365.1902.

#### 2,6-Difluoro-4-[2-(3,5,5,8,8-pentamethyl-5,6,7,8-tetrahydronaphthalen-2-yl)­ethynyl]­benzoic Acid, **19**

Et_3_N (20 mL) was degassed by sparging with Ar for 1 h. **11d** (0.28 g, 1.13 mmol), **4b** (0.3 g, 1.33 mmol), Pd­(PPh_3_)_2_Cl_2_ (79 mg, 0.11 mmol) and CuI (21 mg, 0.11 mmol) were then added under Ar and the resultant suspension was stirred at RT for 72 h. The suspension was diluted with MTBE, passed through Celite/SiO_2_ and the extracts were evaporated to give a crude solid. This was purified by dry column vacuum chromatography (100% heptane to heptane/EtOAc, 9:1) to give the intermediate ester as an off-white solid (0.50 g, >100%). This was dissolved in THF (30 mL), 20% NaOH (3 mL) was added, and the resultant solution was stirred at reflux for 72 h. The mixture was cooled, acidified to pH 1 with 5% HCl, extracted with EtOAc, washed with H_2_O and brine, dried (MgSO_4_), and evaporated to give a crude white solid. This was purified by recrystallization from MeCN to give **19** as a white solid (0.23 g, 53% over two steps): ^1^H NMR (600 MHz, DMSO-*d*_6_) δ 1.23 (s, 12H), 1.62 (s, 4H), 2.40 (s, 3H), 7.28 (s, 1H), 7.40–7.45 (m, 2H), 7.47 (s, 1H); ^13^C NMR (151 MHz, DMSO-*d*_6_) δ 19.9, 31.3, 31.4, 33.5, 34.0, 34.4, 34.4, 89.4 (t, *J* = 3.5 Hz), 92.6, 112.1 (t, *J* = 20.1 Hz), 114.9 (d, *J* = 4.6 Hz), 115.0 (d, *J* = 4.8 Hz), 118.3, 127.1 (t, *J* = 12.6 Hz), 127.7, 130.1, 137.0, 142.3, 146.5, 159.3 (dd, *J* = 252.5, 8.3 Hz), 161.7; ^19^F NMR (376 MHz, DMSO-*d*_6_) δ −111.6; IR (ATR) *v*_max_/cm^–1^ 2956m, 2923m, 2857w, 2209m, 1694s, 1622s, 1553m, 1428m, 1407m, 1281s, 1047s, 856s; MS­(ASAP): *m*/*z* = 383.2 [M + H]^+^; HRMS (ASAP) calcd. for C_24_H_25_O_2_F_2_ [M + H]^+^: 383.1800, found 383.1810. Note: ^1^H resonance for COOH was not detected.

#### 2-Fluoro-4-[2-(3-methoxy-5,5,8,8-tetramethyl-5,6,7,8-tetrahydronaphthalen-2-yl)­ethynyl]­benzoic Acid, **20**

Et_3_N (12 mL) was degassed by sparging with Ar for 1 h. **11b** (0.26 g, 1.13 mmol), **4c** (0.30 g, 1.24 mmol), Pd­(PPh_3_)_2_Cl_2_ (79 mg, 0.11 mmol) and CuI (22 mg, 0.11 mmol) were then added under Ar and the resultant suspension was stirred at RT for 40 h. The suspension was diluted with MTBE and passed through Celite/SiO_2_ and the extracts were evaporated to give a crude solid. This was purified by dry column vacuum chromatography (100% heptane to heptane/EtOAc, 9:1) to give the intermediate ester as a white solid (0.47 g, >100%). This was dissolved in THF (30 mL), 20% NaOH (3 mL) was added, and the resultant solution was stirred at reflux for 72 h. The mixture was cooled, acidified to pH 1 with 5% HCl, extracted with EtOAc, washed with H_2_O and brine, dried (MgSO_4_), and evaporated to give a crude white solid. This was purified by SiO_2_ chromatography (DCM/MeOH, 95:5, with 0.25% AcOH) to give compound **20** as white solid (0.33 g, 77% over three steps): ^1^H NMR (700 MHz, DMSO-*d*_6_) δ 1.22 (s, 6H), 1.27 (s, 6H), 1.56 – 1.70 (m, 4H), 3.84 (s, 3H), 6.94 (s, 1H), 7.41 (dd, *J* = 8.0, 1.5 Hz, 1H), 7.43 – 7.46 (m, 2H), 7.88 (t, *J* = 7.9 Hz, 1H), 13.38 (br, 1H); ^13^C NMR (176 MHz, DMSO-*d*_6_) δ 31.2, 31.5, 33.3, 34.4, 34.5, 34.6, 55.7, 90.0 (d, *J* = 2.7 Hz), 90.5, 108.2, 108.9, 118.9 (d, *J* = 10.3 Hz), 119.1 (d, *J* = 24.1 Hz), 127.2 (d, *J* = 3.5 Hz), 128.9 (d, *J* = 10.4 Hz), 131.4, 132.2 (d, *J* = 1.9 Hz), 136.8, 148.2, 157.5, 160.8 (d, *J* = 257.9 Hz), 164.4 (d, *J* = 3.2 Hz); ^19^F NMR (376 MHz, DMSO-*d*_6_) δ −110.1; IR (ATR) *v*_max_/cm^–1^ 2958m, 2920m, 2851m, 2208m, 1685s, 1614m, 1556m, 1439s, 1292s, 1245s, 869m, 773s; MS­(ASAP): *m*/*z* = 381.2 [M + H]^+^; HRMS (ASAP) calcd. for C_24_H_25_O_3_F [M]^+^: 380.1788, found 380.1790.

#### 3-Fluoro-4-[2-(3-methoxy-5,5,8,8-tetramethyl-5,6,7,8-tetrahydronaphthalen-2-yl)­ethynyl]­benzoic Acid, **21**

Anhydrous toluene (70 mL) was degassed by sparging with N_2_ for 1 h. **3c** (0.8 g, 2.32 mmol), **12a** (0.50 g, 2.79 mmol), Et_3_N (0.79 mL, 5.58 mmol), Pd­(PPh_3_)_2_Cl_2_ (163 mg, 0.23 mmol) and CuI (44 mg, 0.23 mmol) were then added under N_2_ and the resultant suspension was stirred at RT for 72 h. The suspension was diluted with heptane, passed through Celite/SiO_2_ and the extracts were evaporated to give a crude solid (1.12 g). This was purified by dry column vacuum chromatography (100% heptane to heptane/EtOAc, 9:1) to give the intermediate ester as a crystalline white solid (0.13 g, 14%). This was dissolved in THF (30 mL), 20% NaOH (3 mL) was added, and the resultant solution was stirred at reflux for 16 h. The mixture was cooled, acidified to pH 1 with 5% HCl, extracted with EtOAc, washed with H_2_O and brine, dried (MgSO_4_), and evaporated to give a crude white solid which was recrystallized from MeCN to give compound **21** as a colorless solid (78 mg, 62%): ^1^H NMR (600 MHz, DMSO-*d*_6_) δ 1.23 (s, 6H), 1.28 (s, 6H), 1.50 – 1.76 (m, 4H), 3.84 (s, 3H), 6.95 (s, 1H), 7.42 (s, 1H), 7.70 (t, *J* = 7.6 Hz, 1H), 7.74 (dd, *J* = 9.9, 1.6 Hz, 1H), 7.79 (dd, *J* = 7.6, 1.6 Hz, 1H), 13.42 (br, 1H); ^13^C NMR (151 MHz, DMSO-*d*_6_) δ 31.3, 31.5, 33.3, 34.4, 34.5, 34.6, 55.7, 84.6, 94.5 (d, *J* = 3.1 Hz), 108.2, 109.0, 115.7 (d, *J* = 15.9 Hz), 116.0 (d, *J* = 22.3 Hz), 125.4 (d, *J* = 3.4 Hz), 131.2, 132.5 (d, *J* = 6.9 Hz), 133.5, 136.9, 148.3, 157.5, 161.2 (d, *J* = 250.2 Hz), 165.7 (d, *J* = 2.6 Hz); ^19^F NMR (376 MHz, DMSO-*d*_6_) δ −109.8; IR (ATR) *v*_max_/cm^–1^ 2960m, 2921m, 2859m, 2213m, 1689s, 1607m, 1564m, 1494m, 1427m, 1248m, 762s; MS­(ASAP): *m*/*z* = 381.2 [M + H]^+^; HRMS (ASAP) calcd. for C_24_H_26_O_3_F [M + H]^+^: 381.1866, found 381.1866.

#### 2,6-Difluoro-4-[2-(3-methoxy-5,5,8,8-tetramethyl-5,6,7,8-tetrahydronaphthalen-2-yl)­ethynyl]­benzoic Acid, **22**

Et_3_N (20 mL) was degassed by sparging with Ar for 1 h. **11d** (0.28 g, 1.13 mmol), **4c** (0.3 g, 1.24 mmol), Pd­(PPh_3_)_2_Cl_2_ (79 mg, 0.11 mmol) and CuI (21 mg, 0.11 mmol) were then added under Ar and the resultant suspension was stirred at RT for 16 h. The suspension was diluted with MTBE and passed through Celite/SiO_2_ and the extracts were evaporated to give a crude solid. This was purified by dry column vacuum chromatography (100% heptane to heptane/EtOAc, 9:1) to give an off-white solid which was further recrystallized from MeOH to give the intermediate ester as a white solid (0.25 g, 53%). This was dissolved in THF (30 mL), 20% NaOH (3 mL) was added, and the resultant solution was stirred at reflux for 32 h. The mixture was cooled, acidified to pH 1 with 5% HCl, extracted with EtOAc, washed with H_2_O and brine, dried (MgSO_4_), and evaporated to give a crude white solid. This was purified by SiO_2_ chromatography (DCM/MeOH, 95:5, with 0.5% AcOH) to give compound **22** as a white solid (0.19 g, 80% over two steps): ^1^H NMR (700 MHz, DMSO-*d*_6_) δ 1.22 (s, 6H), 1.27 (s, 6H), 1.59–1.67 (m, 4H), 3.84 (s, 3H), 6.94 (s, 1H), 7.34–7.40 (m, 2H), 7.45 (s, 1H); ^13^C NMR (176 MHz, DMSO-*d*_6_) δ 31.2, 31.5, 33.3, 34.4, 34.4, 34.6, 55.7, 89.2 (t, *J* = 3.4 Hz), 90.8, 107.9, 108.9, 112.0 (d, *J* = 19.6 Hz), 114.8 (d, *J* = 4.2 Hz), 114.9 (d, *J* = 4.5 Hz), 127.2 (t, *J* = 12.5 Hz), 131.5, 136.9, 148.4, 157.5, 159.2 (dd, *J* = 252.6, 8.4 Hz), 161.6; ^19^F NMR (376 MHz, DMSO-*d*_6_) δ −111.6; IR (ATR) *v*_max_/cm^–1^ 2965m, 2907m, 2855w, 2211m, 1693s, 1621s, 1552m, 1432m, 1306m, 1287m, 1047s, 856s; MS­(ES): *m*/*z* = 399.2 [M + H]^+^; HRMS (ES) calcd. for C_24_H_24_O_3_F_2_ [M + H]^+^: 398.1694, found 398.1708. Note: ^1^H resonance for COOH was not detected.

#### 6-[2-(5,5,8,8-Tetramethyl-5,6,7,8-tetrahydronaphthalen-2-yl)­ethynyl]­pyridine-3-carboxylic Acid, **23**

Et_3_N/THF (1:1, 120 mL) was added to an oven-dried 100 mL RBF under N_2_, and the solution was degassed by sparging with N_2_ for 1 h. Pd­(PPh_3_)_2_Cl_2_ (138 mg, 0.20 mmol), CuI (38 mg, 0.20 mmol) compound **4a** (1.0 g, 4.71 mmol) and compound **11g** (0.85 g, 3.93 mmol) were added under N_2_ and the resultant solution was stirred at 50 °C for 40 h. The solution was diluted with heptane, and eluted through a Celite/SiO_2_ plug and the extracts were evaporated to give a crude white solid (1.6 g). This was purified by SiO_2_ chromatography (heptane/EtOAc, 8:2) to give the intermediate ester as a white solid (1.18 g, 86%). The ester (0.65 g, 1.87 mmol) was dissolved in THF (30 mL), 20% NaOH (3 mL) was added, and the resultant solution was stirred at reflux for 16 h. The mixture was cooled, acidified to pH 1 with 5% HCl, extracted with EtOAc, washed with H_2_O and brine, dried (MgSO_4_), and evaporated to give a crude white solid which was recrystallized from MeCN to give compound **23** as a colorless crystalline solid (0.56 g, 90%): ^1^H NMR (700 MHz, DMSO-*d*_6_) δ 1.25 and 1.27 (s, 12H), 1.65 (s, 4H), 7.37 (dd, *J* = 8.1, 1.8 Hz, 1H), 7.42 (d, *J* = 8.2 Hz, 1H), 7.58 (d, *J* = 1.8 Hz, 1H), 7.75 (dd, *J* = 8.1, 0.9 Hz, 1H), 8.28 (dd, *J* = 8.1, 2.2 Hz, 1H), 9.06 (dd, *J* = 2.2, 0.9 Hz, 1H), 13.54 (br, 1H); ^13^C NMR (176 MHz, DMSO-*d*_6_) δ 31.3, 31.4, 33.9, 34.1, 34.2, 34.3, 87.7, 91.8, 118.0, 125.4, 126.9, 127.1, 128.9, 130.1, 137.4, 145.2, 145.8, 146.8, 150.6, 165.7; IR (ATR) *v*_max_/cm^–1^ 2960m, 2928m, 2925m, 2859m, 2207m, 1686s, 1588s, 1421m, 1294s, 1294s, 1275s, 831s, 779s; MS­(ES): *m*/*z* = 334.2 [M + H]^+^; HRMS (ES) calcd. for C_22_H_24_NO_2_ [M + H]^+^: 334.1807, found 334.1811.

#### 5-[2-(5,5,8,8-Tetramethyl-5,6,7,8-tetrahydronaphthalen-2-yl)­ethynyl]­pyridine-2-carboxylic Acid, **24**

Et_3_N/THF (1:1, 120 mL) was added to an oven-dried 100 mL RBF under N_2_, and the solution was degassed by sparging with N_2_ for 1 h. Pd­(PPh_3_)_2_Cl_2_ (0.265 g, 0.38 mmol), CuI (0.072 g, 0.38 mmol), **4a** (0.8 g, 4.80 mmol) and **11f** (0.98 g, 4.52 mmol) were added under N_2_ and the resultant solution was stirred at 50 °C for 40 h. The solution was diluted with heptane, eluted through a Celite/SiO_2_ plug and the extracts were evaporated to give a crude brown solid (1.9 g). This was purified by dry column vacuum chromatography (100% heptane to heptane/EtOAc, 8:2), to give the intermediate ester as a white solid (0.36 g, 27%). The ester (0.33 g, 0.95 mmol) was dissolved in THF (30 mL), 20% NaOH (3 mL) was added, and the resultant solution was stirred at reflux for 16 h. The mixture was cooled, acidified to pH 1 with 5% HCl, extracted with EtOAc, washed with H_2_O and brine, dried (MgSO_4_), and evaporated to give a crude white solid. This was recrystallized from MeCN to give compound **24** as a colorless crystalline solid (0.30 g, 96%): ^1^H NMR (700 MHz, DMSO-*d*_6_) δ 1.25 and 1.27 (s, 12H), 1.65 (s, 4H), 7.35 (dd, *J* = 8.1, 1.8 Hz, 1H), 7.41 (d, *J* = 8.1 Hz, 1H), 7.57 (d, *J* = 1.8 Hz, 1H), 8.06 (dd, *J* = 8.1, 0.9 Hz, 1H), 8.12 (dd, *J* = 8.1, 2.1 Hz, 1H), 8.85 (dd, *J* = 2.1, 0.9 Hz, 1H), 13.35 (s, 1H);^13^C NMR (176 MHz, DMSO-*d*_6_) δ 31.3, 31.4, 33.9, 34.1, 34.2, 34.3, 39.5, 84.8, 95.4, 118.4, 122.7, 124.3, 127.1, 128.7, 129.8, 139.5, 145.2, 146.4, 146.9, 1S551.3, 165.6; IR (ATR) *v*_max_/cm^–1^ 3283br, 2955m, 2920m, 2856m, 2208m, 1752s, 1586m, 1336s, 1247m, 1017m, 833m; MS­(ES): *m*/*z* = 334.2 [M + H]^+^; HRMS (ES) calcd. for C_22_H_24_NO_2_ [M + H]^+^: 334.1807, found 334.1808.

#### 5-[2-(5,5,8,8-Tetramethyl-5,6,7,8-tetrahydronaphthalen-2-yl)­ethynyl]­pyrazine-2-carboxylic Acid, **25**

Et_3_N/THF (1:1, 120 mL) was added to an oven-dried 100 mL RBF under N_2_, and the solution was degassed by sparging with N_2_ for 1 h. Pd­(PPh_3_)_2_Cl_2_ (276 mg, 0.39 mmol), CuI (75 mg, 0.39 mmol), **4a** (1.0 g, 4.71 mmol) and **11h** (0.68 g, 3.93 mmol) were added under N_2_ and the resultant solution was stirred at 50 °C for 40 h. The solution was diluted with heptane, eluted through a Celite/SiO_2_ plug and the extracts were evaporated to give a crude white solid (1.3 g). This was purified by dry column vacuum chromatography (100% heptane to heptane/EtOAc, 8:2), and further recrystallized from EtOH to give the intermediate ester as a white solid (0.42 g, 31%). This was dissolved in THF (30 mL), 20% NaOH (3 mL) was added, and the resultant solution was stirred at reflux for 16 h. The mixture was cooled, acidified to pH 1 with 5% HCl, extracted with EtOAc, washed with H_2_O and brine, dried (MgSO_4_), and evaporated to give a crude white solid. This was purified by SiO_2_ chromatography (DCM/MeOH, 95:5, with 1% AcOH) to give compound **25** as a colorless crystalline solid (0.15 g, 37%): ^1^H NMR (600 MHz, DMSO-*d*_6_) δ 1.26 (d, *J* = 11.0 Hz, 12H), 1.65 (s, 4H), 7.41 (dd, *J* = 8.2, 1.7 Hz, 1H), 7.45 (d, *J* = 8.2 Hz, 1H), 7.64 (d, *J* = 1.7 Hz, 1H), 8.96 (s, 1H), 9.17 (s, 1H), 12.93 (s, 1H); ^13^C NMR (151 MHz, DMSO-*d*_6_) δ 31.3, 31.4, 34.0, 34.2, 34.2, 34.3, 85.5, 95.6, 117.5, 127.3, 129.1, 130.3, 141.4, 141.7, 145.3, 145.4, 146.7, 147.4, 164.7, 172.0; IR (ATR) *v*_max_/cm^–1^ 2962m, 2924m, 2857m, 2211m, 1726s, 1567w, 1496m, 1258m, 1171s, 1033m; MS­(ES): *m*/*z* = 335.2 [M + H]^+^; HRMS (ES) calcd. for C_21_H_23_N_2_O_2_ [M + H]^+^: 335.1760, found 335.1764.

#### 6-[2-(3,5,5,8,8-Pentamethyl-5,6,7,8-tetrahydronaphthalen-2-yl)­ethynyl]­pyridine-3-carboxylic Acid, **26**

Et_3_N (20 mL) was degassed by sparging with Ar for 1 h. **11g** (0.26 g, 1.18 mmol), compound **4b** (0.32 g, 1.40 mmol), Pd­(PPh_3_)_2_Cl_2_ (83 mg, 0.12 mmol) and CuI (22 mg, 0.12 mmol) were then added under Ar and the resultant suspension was stirred at RT for 20 h. The suspension was diluted with MTBE and passed through Celite/SiO_2_ and the extracts were evaporated to give a crude solid (0.5 g). This was purified by dry column vacuum chromatography (100% heptane to heptane/EtOAc, 8:2) to give an off-white solid which was subsequently recrystallized from MeOH to give the intermediate ester as a white solid (0.25 g, 58%). This was dissolved in THF (30 mL), 20% NaOH (3 mL) was added, and the resultant solution was stirred at reflux for 16 h. The mixture was cooled, acidified to pH 1 with 5% HCl, extracted with EtOAc, washed with H_2_O and brine, dried (MgSO_4_), and evaporated to give a crude white solid which was recrystallized from MeCN to give compound **26** as colorless crystalline solid (0.20 g, 83%): ^1^H NMR (600 MHz, DMSO-*d*_6_) δ 1.24 (s, 12H), 1.63 (s, 4H), 2.42 (s, 3H), 7.30 (s, 1H), 7.51 (s, 1H), 7.75 (dd, *J* = 8.1, 0.9 Hz, 1H), 8.28 (dd, *J* = 8.1, 2.2 Hz, 1H), 9.06 (dd, *J* = 2.2, 0.9 Hz, 1H), 13.53 (br, 1H); ^13^C NMR (151 MHz, DMSO-*d*_6_) δ 19.8, 31.3, 31.4, 33.6, 34.0, 34.4, 34.4, 90.8, 91.5, 118.1, 125.3, 126.9, 127.8, 130.3, 137.1, 137.4, 142.4, 146.0, 146.8, 150.7, 165.8; IR (ATR) *v*_max_/cm^–1^ 2958w, 2925w, 2858w, 2206w, 1705s, 1591s, 1564w, 1449m, 1296s, 1267s, 778m; MS­(ES): *m*/*z* = 348.2 [M + H]^+^; HRMS (ES) calcd. for C_23_H_26_NO_2_ [M + H]^+^: 348.1964, found 348.1949.

#### 6-[2-(3-Methoxy-5,5,8,8-tetramethyl-5,6,7,8-tetrahydronaphthalen-2-yl)­ethynyl]­pyridine-3-carboxylic Acid, **27**

Et_3_N (25 mL) was degassed by sparging with Ar for 1 h. **11g** (0.30 g, 1.38 mmol), compound **4c** (0.4 g, 1.65 mmol), Pd­(PPh_3_)_2_Cl_2_ (97 mg, 0.14 mmol) and CuI (26 mg, 0.14 mmol) were then added under Ar and the resultant suspension was stirred at RT for 16 h. The suspension was diluted with MTBE and passed through Celite/SiO_2_ and the extracts were evaporated to give a crude solid (0.8 g). This was purified by dry column vacuum chromatography (100% heptane to heptane/EtOAc, 8:2) to give an off-white solid which was subsequently recrystallized from MeOH to give the intermediate ester as a colorless crystalline solid (0.40 g, 76%). The ester (0.38 g, 1.01 mmol) was dissolved in THF (20 mL), 20% NaOH (2 mL) was added, and the resultant solution was stirred at reflux for 16 h. The mixture was cooled, acidified to pH 1 with 5% HCl, extracted with EtOAc, washed with H_2_O and brine, dried (MgSO_4_), and evaporated to give a crude light yellow solid which was purified by SiO_2_ chromatography (DCM/MeOH, 95:5, 1% AcOH) to give a yellow solid which was further recrystallized from MeCN to give compound **27** as a light yellow crystalline solid (0.30 g, 83%): ^1^H NMR (600 MHz, DMSO-*d*_6_) δ 1.23 and 1.28 (s, 12H), 1.60–1.67 (m, 4H), 3.86 (s, 3H), 6.96 (s, 1H), 7.47 (s, 1H), 7.70 (dd, *J* = 8.1, 0.9 Hz, 1H), 8.26 (dd, *J* = 8.1, 2.2 Hz, 1H), 9.05 (dd, *J* = 2.2, 0.9 Hz, 1H), 13.34 (br, 1H); ^13^C NMR (151 MHz, DMSO-*d*_6_) δ 31.3, 31.5, 33.3, 34.4, 34.5, 34.7, 55.7, 89.0, 91.4, 107.7, 109.0, 125.3, 126.8, 131.7, 137.0, 137.4, 146.1, 148.7, 150.6, 157.8, 165.8; IR (ATR) *v*_max_/cm^–1^ 2958m, 2925m, 2957w, 2211m, 1711m, 1590s, 1504m, 1460m, 1272m, 1241s, 779m; MS­(ASAP): *m*/*z* = 364.2 [M + H]^+^; HRMS (ASAP) calcd. for C_23_H_26_NO_3_ [M + H]^+^: 364.1913, found 364.1916.

#### 2-[2-(3,5,5,8,8-Pentamethyl-5,6,7,8-tetrahydronaphthalen-2-yl)­ethynyl]­pyrimidine-5-carboxylic Acid, **28**

Anhydrous toluene (8 mL) was degassed by sparging with Ar for 1 h. Na_2_CO_3_ (0.22 g, 2.1 mmol), Na_2_PdCl_4_ (8.8 mg, 0.03 mmol), CuI (4.4 mg, 0.023 mmol), [(*t*-Bu)_3_PH]­BF_4_ (17 mg, 0.06 mmol), **11i** (0.28 g, 1.50 mmol) and compound **4b** (0.45 g, 2.00 mmol) were then added under Ar and the resultant suspension was stirred at 100 °C for 20 h. The solvent was evaporated and the crude residue was purified by dry column vacuum chromatography (100% heptane to heptane/EtOAc, 9:1) to give the intermediate ester as a clear oil that slowly crystallized (0.75 g, >100%). This was dissolved in THF (30 mL), 20% NaOH (3 mL) was added, and the resultant solution was stirred at reflux for 16 h. The mixture was cooled, acidified to pH 1 with 5% HCl, extracted with EtOAc, washed with H_2_O and brine, dried (MgSO_4_), and evaporated to give a crude white solid. This was purified by SiO_2_ chromatography (DCM/MeOH, 95:5, with 0.2% AcOH), and further recrystallized from MeCN to give compound **28** as a white solid (0.29 g, 56% over three steps): ^1^H NMR (700 MHz, DMSO-*d*_6_) δ 1.25 (s, 12H), 1.63 (s, 4H), 2.44 (s, 3H), 7.33 (s, 1H), 7.55 (s, 1H), 9.20 (s, 2H), 13.88 (br, 1H); ^13^C NMR (176 MHz, DMSO-*d*_6_) δ 19.7, 31.2, 31.4, 33.6, 34.1, 34.3, 34.3, 89.0, 91.3, 117.3, 122.7, 127.9, 130.9, 137.8, 142.6, 147.6, 154.5, 158.2, 164.5; IR (ATR) *v*_max_/cm^–1^ 2958m, 2925m, 2860m, 2213m, 1713s, 1578s, 1496m, 1280s, 798m; MS­(ASAP): *m*/*z* = 349.2 [M + H]^+^; HRMS (ASAP) calcd. for C_22_H_25_N_2_O_2_ [M + H]^+^: 349.1916, found 349.1909.

#### 2-[2-(3-Methoxy-5,5,8,8-tetramethyl-5,6,7,8-tetrahydronaphthalen-2-yl)­ethynyl]­pyrimidine-5-carboxylic Acid, **29**

Anhydrous toluene (8 mL) was degassed by sparging with Ar for 1 h. Na_2_CO_3_ (0.22 g, 2.1 mmol), Na_2_PdCl_4_ (8.8 mg, 0.03 mmol), CuI (4.4 mg, 0.023 mmol), [(*t*-Bu)_3_PH]­BF_4_ (17 mg, 0.06 mmol), **11i** (0.28 g, 1.50 mmol) and compound **4c** (0.48 g, 2.00 mmol) were then added under Ar and the resultant suspension was stirred at 100 °C for 20 h. The solvent was evaporated and the crude residue was purified by dry column vacuum chromatography (100% heptane to 9:1, heptane/EtOAc) to give a yellow solid which was further recrystallized from MeOH to give the intermediate ester as a light yellow crystalline solid (0.33 g, 55%). The ester (0.30 g, 0.76 mmol) was dissolved in THF (30 mL), 20% NaOH (3 mL) was added, and the resultant solution was stirred at reflux for 16 h. The mixture was cooled, acidified to pH 1 with 5% HCl, extracted with EtOAc, washed with H_2_O and brine, dried (MgSO_4_), and evaporated to give a crude white solid. This was purified by SiO_2_ chromatography (DCM/MeOH, 95:5, with 0.3% AcOH), and further recrystallized from MeCN to give compound **29** as a light yellow solid (0.23 g, 83%): ^1^H NMR (600 MHz, DMSO-*d*_6_) δ 1.24 and 1.29 (s, 12H), 1.59–1.68 (m, 4H), 3.87 (s, 3H), 6.99 (s, 1H), 7.51 (s, 1H), 9.19 (s, 2H), 13.80 (br, 1H); ^13^C NMR (151 MHz, DMSO-*d*_6_) δ 31.2, 31.5, 33.3, 34.4, 34.4, 34.8, 55.7, 87.5, 91.3, 106.9, 109.1, 122.6, 132.2, 137.2, 149.7, 154.6, 158.2, 158.4, 164.6; IR (ATR) *v*_max_/cm^–1^ 2958m, 2927m, 2857m, 2213m, 1720m, 1579s, 1502m, 1232s, 1045m, 801m; MS­(ASAP): *m*/*z* = 365.2 [M + H]^+^; HRMS (ASAP) calcd. for C_22_H_25_N_2_O_3_ [M + H]^+^: 365.1865, found 365.1876.

#### 4-[2-(5,5,8,8-Tetramethyl-5,6,7,8-tetrahydroquinoxalin-2-yl)­ethynyl]­benzoic Acid, **30**

Et_3_N (20 mL) was degassed by sparging with Ar for 1 h. **11a** (0.31 g, 1.20 mmol), **10** (0.30 g, 1.40 mmol), Pd­(PPh_3_)_2_Cl_2_ (83 mg, 0.12 mmol) and CuI (22 mg, 0.12 mmol) were then added under Ar and the resultant suspension was stirred at RT for 16 h. The suspension was diluted with MTBE and passed through Celite/SiO_2_ and the extracts were evaporated to give a crude solid (0.5 g). This was purified by dry column vacuum chromatography (100% heptane to 85:15, heptane/EtOAc) to give an off-white solid which was subsequently recrystallized from MeOH to give the intermediate ester as a colorless crystalline solid (0.29 g, 71%). The ester (0.28 g, 0.8 mmol) was dissolved in THF (20 mL), 20% NaOH (2 mL) was added, and the resultant solution was stirred at reflux for 16 h. The mixture was cooled, acidified to pH 1 with 5% HCl, extracted with EtOAc, washed with H_2_O and brine, dried (MgSO_4_), and evaporated to give a crude white solid which was recrystallized from MeCN to give compound **30** as a colorless crystalline solid (0.24 g, 89%): ^1^H NMR (700 MHz, DMSO-*d*_6_) δ 1.29 (s, 12H), 1.78 (s, 4H), 7.73–7.79 (m, 2H), 7.98–8.02 (m, 2H), 8.68 (s, 1H), 13.24 (br, 1H); ^13^C NMR (176 MHz, DMSO-*d*_6_) δ 29.4, 29.4, 33.1, 33.2, 37.0, 37.1, 88.9, 89.8, 125.2, 129.6, 131.3, 131.9, 135.2, 144.4, 157.6, 158.0, 166.5; IR (ATR) *v*_max_/cm^–1^ 2961w, 2925w, 2958w, 2223w, 1683s, 1606m, 1558w, 1428m, 1282s, 862s, 769m; MS­(ASAP): *m*/*z* = 334.2 [M]^+^; HRMS (ASAP) calcd. for C_21_H_22_N_2_O_2_ [M]^+^: 334.1681, found 334.1686.

#### 3-Fluoro-4-[2-(5,5,8,8-tetramethyl-5,6,7,8-tetrahydroquinoxalin-2-yl)­ethynyl]­benzoic Acid, **31**

Et_3_N (12 mL) was degassed by sparging with Ar for 1 h. **11j** (0.28 g, 1.00 mmol), compound **10** (0.26 g, 1.20 mmol), Pd­(PPh_3_)_2_Cl_2_ (70 mg, 0.10 mmol) and CuI (19 mg, 0.10 mmol) were then added under Ar and the resultant suspension was stirred at RT for 20 h. The suspension was diluted with MTBE and passed through Celite/SiO_2_ and the extracts were evaporated to give a crude solid (0.6 g). This was purified by SiO_2_ chromatography (95:5, heptane/EtOAc) to give DC710 as a colorless oil which slowly crystallized (0.41 g, >100%). This was dissolved in THF (30 mL), 20% NaOH (3 mL) was added, and the resultant solution was stirred at reflux for 16 h. The mixture was cooled, acidified to pH 1 with 5% HCl, extracted with EtOAc, washed with H_2_O and brine, dried (MgSO_4_), and evaporated to give a crude white solid which was recrystallized from MeCN to give compound **31** as a colorless crystalline solid (0.28 g, 79% over three steps): ^1^H NMR (700 MHz, DMSO-*d*_6_) δ 1.29 (s, 12H), 1.78 (s, 2H), 7.80 (dd, *J* = 9.8, 1.5 Hz, 1H), 7.82–7.88 (m, 2H), 8.70 (s, 1H), 13.55 (br, 1H); ^13^C NMR (176 MHz, DMSO-*d*_6_) δ 29.4, 29.4, 33.0, 33.2, 37.0, 37.1, 83.2, 93.5 (d, *J* = 3.2 Hz), 113.8 (d, *J* = 15.6 Hz), 116.2 (d, *J* = 22.1 Hz), 125.5 (d, *J* = 3.4 Hz), 133.9 (d, *J* = 7.1 Hz), 134.1, 134.8, 144.4, 158.0, 158.2, 161.8 (d, *J* = 251.8 Hz), 165.5 (d, *J* = 2.5 Hz); ^19^F NMR (376 MHz, DMSO-*d*_6_) δ −108.9; IR (ATR) *v*_max_/cm^–1^ 2968m, 2944m, 2925m, 2863m, 2214w, 1692s, 1617m, 1566m, 1426s, 1294s, 1219s, 892s, 763m; MS­(ASAP): *m*/*z* = 353.1 [M + H]^+^; HRMS (ASAP) calcd. for C_21_H_22_N_2_O_2_F [M + H]^+^: 353.1665, found 353.1663.

#### 1-(5,5,8,8-Tetramethyl-5,6,7,8-tetrahydroquinoxalin-2-yl)­propan-1-one, **34**

Compound **9** (3.9 g, 17.86 mmol) was dissolved in anhydrous THF (100 mL) under N_2_, and the solution was heated to reflux. EtMgBr (3.0 M in Et_2_O, 5.95 mL, 17.86 mmol) was carefully added dropwise, and the resultant solution was stirred at reflux for 1 h. The solution was cooled, diluted with sat. NH_4_Cl and extracted with EtOAc. The organics were washed with H_2_O and brine, dried (MgSO_4_), and evaporated to give a crude oil (3.9 g). This was purified by SiO_2_ chromatography (8:2, heptane/EtOAc) to give compound **32** as a clear oil (1.58 g, 36%): ^1^H NMR (400 MHz, CDCl_3_) δ 0.98 (t, *J* = 7.4 Hz, 3H), 1.33–1.36 (4*s*, 12H), 1.69–1.75 (m, 1H), 1.80 (s, 4H), 1.86–1.95 (m, 1H), 4.72 (dd, *J* = 7.3, 4.6 Hz, 1H), 8.37 (s, 1H); IR (ATR) *v*_max_/cm^–1^ 3431br, 2959m, 2928m, 2863m, 1456m, 1360m, 1130s, 1079s; MS (ESI) *m*/*z* = 249.4 [M + H]^+^; HRMS (ESI) calcd. for C_15_H_25_O_2_N [M + H]^+^: 249.1969, found 249.1967. Oxalyl chloride (0.93 mL, 11.00 mmol) was added to anhydrous DCM (50 mL) under N_2_, and the resultant solution was cooled to −78 °C. DMSO (1.56 mL, 22.00 mmol) was added dropwise, and the solution was stirred for 15 min at −78 °C. A solution of **32** (1.82 g, 7.33 mmol) in DCM (20 mL) was added slowly so as to maintain the internal temperature below −60 °C. The resultant solution was stirred for 15 min, before triethylamine (7.36 mL, 52.79 mmol) was added. The solution was stirred for 15 min at −78 °C, before being allowed to reach RT and stirred for a further 30 min. H_2_O and DCM was added, and the organics were washed with H_2_O, dried (MgSO_4_), and evaporated to give a crude brown oil (2.0 g). This was purified by SiO_2_ chromatography (95:5, heptane/EtOAc) to give compound **34** as a colorless oil (1.70 g, 94%): ^1^H NMR (700 MHz, CDCl_3_) δ 1.21 (t, *J* = 7.3 Hz, 3H), 1.34 and 1.35 (s,12H), 1.83 (s, 4H), 3.19 (q, *J* = 7.3 Hz, 2H), 8.95 (s, 1H); ^13^C NMR (176 MHz, CDCl_3_) δ 7.9, 29.6, 29.8, 31.2, 33.8, 33.9, 37.3, 37.7, 139.7, 144.5, 157.3, 162.3, 202.7: IR (ATR) *v*_max_/cm^–1^ 2960m, 2930m, 2865m, 1701s, 1552w, 1457m, 1355m, 1128m, 1078m; MS­(ES): *m*/*z* = 247.3 [M + H]^+^; HRMS (ASAP) calcd. for C_15_H_23_N_2_O [M + H]^+^: 247.1810, found 247.1803.

#### 2-Bromo-1-(5,5,8,8-tetramethyl-5,6,7,8-tetrahydroquinoxalin-2-yl)­propan-1-one, **35**

To a solution of CuBr_2_ (2.22 g, 9.94 mmol) in EtOAc (25 mL) was added compound **34** (1.36 g, 5.52 mmol) as a solution in CHCl_3_ (25 mL), and the resultant suspension was stirred vigorously at reflux for 16 h. The mixture was cooled, diluted with H_2_O and extracted with EtOAc. The organics were washed with H_2_O and brine, dried (MgSO_4_), and evaporated to give a crude brown oil (1.67 g). This was purified by SiO_2_ chromatography (9:1, heptane/EtOAc) to give compound **35** as a yellow oil (1.65 g, 92%): ^1^H NMR (700 MHz, CDCl_3_) δ 1.34 (d, *J* = 6.4 Hz, 6H), 1.37 (d, *J* = 12.6 Hz, 6H), 1.82–1.85 (m, 4H), 1.90 (d, *J* = 6.8 Hz, 3H), 5.89 (q, *J* = 6.8 Hz, 1H), 9.02 (s, 1H); ^13^C NMR (176 MHz, CDCl_3_) δ 19.5, 29.6, 29.6, 29.8, 29.8, 33.6, 33.8, 37.4, 37.9, 41.0, 141.2, 142.3, 157.6, 163.3, 194.3; IR (ATR) *v*_max_/cm^–1^ 2960m, 2928m, 2864w, 1703s, 1551w, 1456m, 1342m, 1127m, 1078s, 712m; MS­(ES): *m*/*z* = 325.5, 327.5 [M + H]^+^; HRMS (ES) calcd. for C_15_H_22_N_2_OBr [M + H]^+^: 325.0915, found 325.0912.

#### Methyl 4-Carbamothioylbenzoate, **36**

To a solution of methyl-4-cyanobenzoate (3.0 g, 18.62 mmol) in DMF (30 mL) was added NaHS hydrate (3.46 g, approximately 37.24 mmol) and MgCl_2_.6H_2_O (3.79 g, 18.62 mmol), and the resultant suspension was stirred at RT for 4 h. The suspension was diluted with H_2_O, then 1 M HCl, and the resultant precipitate isolated by filtration. This was purified by recrystallization from EtOH to give **36** as a yellow crystalline solid (2.88 g, 79%): ^1^H NMR (400 MHz, DMSO-*d*_6_) δ 3.87 (s, 3H), 7.96 (s, 4H), 9.68 (br, 1H), 10.07 (br, 1H); all other data matched the literature.^53^

#### Methyl 6-Cyanopyridine-3-carboxylate

Methyl nicotinate 1-oxide (6.2 g, 40.5 mmol) and TMSCN (5.07 mL, 40.5 mmol) were added to DCM (150 mL), and the resultant solution was stirred at RT for 5 min. Dimethylcarbamoyl chloride (3.73 mL, 40.5 mmol) was then added, and the solution was stirred at RT for 16 h. 10% aq K_2_CO_3_ (100 mL) was added and the resultant solution was stirred for 10 min, before being diluted with DCM. The organics were washed with H_2_O, dried (MgSO_4_), and evaporated to give a crude yellow solid (6.5 g). This was purified by SiO_2_ chromatography (8:2, heptane/EtOAc) to give methyl 6-cyanopyridine-3-carboxylate as a white solid (3.02 g, 46%): ^1^H NMR (700 MHz, CDCl_3_) δ 4.00 (s, 3H), 7.80 (dd, *J* = 8.0, 0.9 Hz, 1H), 8.44 (dd, *J* = 8.0, 2.1 Hz, 1H), 9.29 (dd, *J* = 2.1, 0.9 Hz, 1H); ^13^C NMR (176 MHz, CDCl_3_) δ 53.0, 116.5, 128.0, 128.5, 137.0, 138.1, 151.8, 164.1; IR (ATR) *v*_max_/cm^–1^ 3091m, 3058w, 2962w, 1725s, 1589s, 1536m, 1437m, 1294s, 1019s, 777s; MS­(ES): *m*/*z* = 163.2 [M + H]^+^; HRMS (ES) calcd. for C_8_H_7_O_2_N_2_ [M + H]^+^: 163.0508, found 163.0505.^54^

#### Methyl 6-Carbamothioylpyridine-3-carboxylate, **37**

To a solution of methyl 6-cyanopyridine-3-carboxylate (0.22 g, 1.36 mmol) in DMF (10 mL) was added NaHS (60%, 0.25 g, 2.21 mmol) and MgCl_2_·6H_2_O (0.28 g, 1.36 mmol). The resultant mixture was stirred at RT for 2 h whereupon H_2_O was added. The resultant mixture was extracted with EtOAc, washed with sat. NH_4_Cl, H_2_O, and brine, dried (MgSO_4_), and evaporated to give a crude solid (1 g). This was purified by dry column vacuum chromatography (9:1 to 6:4, heptane/EtOAc) to give compound **37** as a bright yellow solid (0.17 g, 64%): ^1^H NMR (700 MHz, DMSO-*d*_6_) δ 3.92 (s, 3H), 8.45 (dd, *J* = 2.2, 8.3 Hz, 1H), 8.59 (dd, *J* = 8.3, 0.8 Hz, 1H), 9.05 (dd, *J* = 2.2, 0.8 Hz, 1H), 10.06 (br, 1H), 10.36 (br, 1H); ^13^C NMR (176 MHz, DMSO-*d*_6_) δ 52.63, 124.49, 127.13, 137.97, 148.19, 154.60, 164.67, 193.67; IR (ATR) *v*_max_/cm^–1^ 3321s, 3235m, 3142s, 2955w, 1728s, 1595s, 1435m, 1295s, 874s; MS­(ES): *m*/*z* = 197.2 [M + H]^+^; HRMS (ES) calcd. for C_8_H_9_N_2_O_2_S [M + H]^+^: 197.0385, found 197.0394.

#### Methyl 4-Carbamothioyl-2-fluorobenzoate, **38**

Methyl 4-cyano-2-fluorobenzoate (2.0 g, 11.16 mmol) was dissolved in pyridine (10 mL), before Et_3_N (1.71 mL, 12.28 mmol) and (NH_4_)_2_S (20% in H_2_O, 4.18 mL, 12.28 mmol) were added, and the resultant solution was stirred at 50 °C for 16 h. The solution was cooled, and extracted with EtOAc. The organics were washed with H_2_O and brine, dried (MgSO_4_), and evaporated to give a crude yellow solid (2.1 g). This was purified by dry column vacuum chromatography (100% heptane to 6:4, heptane/EtOAc) to give compound **38** as a yellow solid (0.68 g, 29%): ^1^H NMR (700 MHz, DMSO-*d*_6_) δ 3.87 (s, 3H), 7.73 (dd, *J* = 11.9, 1.7 Hz, 1H), 7.78 (dd, *J* = 8.2, 1.8 Hz, 1H), 7.92 (t, *J* = 7.8 Hz, 1H), 9.73 (s, 1H), 10.19 (s, 1H); ^13^C NMR (176 MHz, DMSO-*d*_6_) δ 52.5, 115.4 (d, *J* = 24.5 Hz), 119.7 (d, *J* = 10.5 Hz), 123.3 (d, *J* = 3.5 Hz), 131.4, 145.1 (d, *J* = 7.9 Hz), 160.0 (d, *J* = 257.6 Hz), 163.5 (d, *J* = 3.6 Hz), 197.3; ^19^F NMR (376 MHz, DMSO-*d*_6_) δ −110.2; IR (ATR) *v*_max_/cm^–1^ 3276w, 3127w, 2954w, 1717s, 1638m, 1618s, 1567m, 1425s, 1244s, 1082m, 873m, 776m; MS (ESI) *m*/*z* = 214.2 [M + H]^+^; HRMS (ESI) calcd. for C_9_H_9_O_2_NSF [M + H]^+^: 214.0338, found 214.0352.

#### Methyl 4-Carbamothioyl-3-fluorobenzoate, **39**

To a solution of methyl-4-cyano-3-fluorobenzoate (2.5 g, 14.0 mmol) in DMF (30 mL) was added NaHS hydrate (2.61 g, approximately 27.9 mmol) and MgCl_2_·6H_2_O (2.84 g, 14.0 mmol), and the resultant suspension was stirred at RT for 4 h. The suspension was diluted with H_2_O, then 1 M HCl, and extracted with EtOAc. The organics were washed with H_2_O and brine, dried (MgSO_4_), and evaporated to give a crude oily yellow solid (3 g). This was purified by recrystallization from EtOH to give compound **39** as an orange crystalline solid (0.91 g, 31%): ^1^H NMR (400 MHz, DMSO-*d*_6_) δ 3.88 (s, 3H), 7.65–7.74 (m, 2H), 7.79 (dd, *J* = 8.0, 1.6 Hz, 1H), 9.78 (s, 1H), 10.30 (s, 1H); ^13^C NMR (101 MHz, DMSO-*d*_6_) δ 52.6, 116.3 (d, *J* = 24.0 Hz), 124.9 (d, *J* = 3.5 Hz), 130.4 (d, *J* = 2.6 Hz), 131.9 (d, *J* = 7.6 Hz), 134.8 (d, *J* = 15.1 Hz), 156.0 (d, *J* = 249.6 Hz), 164.7 (d, *J* = 2.6 Hz), 195.2; ^19^F NMR (376 MHz, DMSO-*d*_6_) δ −115.3; IR (ATR) *v*_max_/cm^–1^ 3329m, 3293m, 3161m, 2954w, 1702s, 1642s, 1570m, 1436m, 1401m, 1287s, 1212s, 1082m, 768s; MS­(ASAP): *m*/*z* = 214.0 [M + H]^+^; HRMS (ASAP) calcd. for C_9_H_9_NO_2_SF [M + H]^+^: 214.0338, found 214.0334.

#### 4-[5-Methyl-4-(5,5,8,8-tetramethyl-5,6,7,8-tetrahydroquinoxalin-2-yl)-1,3-thiazol-2-yl]­benzoic Acid, **40**

To a solution of compound **35** (0.27 g, 0.83 mmol) in anhydrous DMF (10 mL) under N_2_, was added compound **36** (0.20 g, 1.00 mmol) and the resultant solution was stirred at 110 °C for 20 h. The solution was cooled, diluted with H_2_O and extracted with EtOAc. The organics were washed with sat. NaHCO_3_, H_2_O, and brine, dried (MgSO_4_), and evaporated to give a crude yellow solid (0.5 g). This was purified by SiO_2_ chromatography (95:5, heptane/EtOAc) to give the intermediate ester as a white solid (0.30 g, 86%). The ester (0.29 g, 0.69 mmol) was dissolved in THF (30 mL), 20% NaOH (3 mL) was added, and the resultant solution was stirred at reflux for 16 h. The mixture was cooled, acidified to pH 1 with 5% HCl, extracted with EtOAc, washed with H_2_O and brine, dried (MgSO_4_), and evaporated to give a crude white solid which was recrystallized from MeCN to give compound **40** as a white solid (0.24 g, 84%): ^1^H NMR (700 MHz, DMSO-*d*_6_) δ 1.31 and 1.34 (s, 12H), 1.81 (s, 4H), 2.89 (s, 3H), 8.03–8.09 (m, 4H), 9.14 (s, 1H), 13.16 (br, 1H); ^13^C NMR (176 MHz, DMSO-*d*_6_) δ 13.3, 29.5, 29.6, 33.3, 33.4, 36.8, 37.0, 126.0, 130.2, 131.9, 136.0, 136.2, 140.5, 146.0, 147.1, 155.7, 156.1, 161.6, 166.7**;** IR (ATR) *v*_max_/cm^–1^ 2965m, 2925m, 2863m, 1680s, 1608m, 1570w, 1426m, 1290s, 861m, 774m; MS­(ASAP): *m*/*z* = 408.1 [M + H]^+^; HRMS (ASAP) calcd. for C_23_H_26_N_3_O_2_S [M + H]^+^: 408.1746, found 408.1752.

#### 6-[5-Methyl-4-(5,5,8,8-tetramethyl-5,6,7,8-tetrahydroquinoxalin-2-yl)-1,3-thiazol-2-yl]­pyridine-3-carboxylic Acid, **41**

To a solution of compound **35** (0.32 g, 0.97 mmol) in anhydrous DMF (10 mL) under N_2_, was added compound **37** (0.23 g, 1.16 mmol) and the resultant solution was stirred at 110 °C for 20 h. The solution was cooled, diluted with H_2_O and extracted with EtOAc. The organics were washed with sat. NaHCO_3_, H_2_O, and brine, dried (MgSO_4_), and evaporated to give a crude orange solid (0.6 g). This was purified by SiO_2_ chromatography (95:5 to 9:1, heptane/EtOAc) to give the intermediate ester as a white solid (0.26 g, 63%). This was dissolved in THF (30 mL), 20% NaOH (3 mL) was added, and the resultant solution was stirred at reflux for 16 h. The mixture was cooled, acidified to pH 1 with 5% HCl, extracted with EtOAc, washed with H_2_O and brine, dried (MgSO_4_), and evaporated to give a crude white solid which was recrystallized from MeOH to give compound **41** as a white solid (0.18 g, 75%): ^1^H NMR (500 MHz, DMSO-*d*_6_, 80 °C) δ 1.33 and 1.36 (s, 12H), 1.83 (s, 4H), 2.91 (s, 3H), 8.29 (d, *J* = 8.2 Hz, 1H), 8.40 (dt, *J* = 8.2, 1.8 Hz, 1H), 9.07 (s, 1H), 9.14 (d, *J* = 1.8 Hz, 1H); ^13^C NMR (126 MHz, DMSO-*d*_6_, 80 °C) δ 13.0, 29.0, 29.1, 29.2, 29.2, 33.2, 33.3, 36.3, 36.6, 118.3, 126.9, 138.0, 138.0, 140.0, 145.6, 147.4, 150.0, 152.9, 155.4, 155.7, 162.4, 165.2**;** IR (ATR) *v*_max_/cm^–1^ 2965m, 2925m, 2860m, 1685s, 1591m, 1563w, 1423m, 1299s, 995m, 784m; MS­(ASAP): *m*/*z* = 409.1 [M + H]^+^; HRMS (ASAP) calcd. for C_23_H_25_N_4_O_2_S [M + H]^+^: 409.1698, found 409.1697. Note: ^1^H NMR resonance for COOH proton was not observed.

#### 2-Fluoro-4-[5-methyl-4-(5,5,8,8-tetramethyl-5,6,7,8-tetrahydroquinoxalin-2-yl)-1,3-thiazol-2-yl]­benzoic Acid, **42**

To a solution of compound **35** (0.30 g, 0.92 mmol) in anhydrous DMF (10 mL) under N_2_, was added compound **38** (0.24 g, 1.10 mmol) and the resultant solution was stirred at 110 °C for 18 h. The solution was cooled, diluted with H_2_O and extracted with EtOAc. The organics were washed with sat. NaHCO_3_, H_2_O, and brine, dried (MgSO_4_), and evaporated to give a crude white solid (0.7 g). This was purified by SiO_2_ chromatography (95:5 to 9:1, heptane:EtOAc) to give the intermediate ester as a white solid (0.46 g, >100%). This was dissolved in THF (30 mL), 20% NaOH (3 mL) was added, and the resultant solution was stirred at reflux for 40 h. The mixture was cooled, acidified to pH 1 with 5% HCl, extracted with EtOAc, washed with H_2_O and brine, dried (MgSO_4_), and evaporated to give a crude white solid which was recrystallized from MeOH to give compound **42** as a white solid (0.27 g, 68% over three steps): ^1^H NMR (500 MHz, DMSO-*d*_6_, 80 °C) δ 1.30 and 1.32 (s, 12H), 1.79 (s, 4H), 2.88 (s, 3H), 7.79–7.87 (m, 3H), 7.97 (t, *J* = 7.7 Hz, 1H), 9.13 (s, 1H), 13.41 (br, 1H); ^13^C NMR (126 MHz, DMSO-*d*_6_, 80 °C) δ 13.4, 29.5, 29.6, 33.3, 33.4, 36.8, 37.0, 113.8 (d, *J* = 24.8 Hz), 120.1 (d, *J* = 10.5 Hz), 121.8 (d, *J* = 3.6 Hz), 133.0, 136.7, 138.1 (d, *J* = 9.0 Hz), 140.6, 145.9, 147.2, 155.7, 156.0, 160.0 (d, *J* = 2.6 Hz), 161.4 (d, *J* = 258.0 Hz), 164.4 (d, *J* = 3.1 Hz); ^19^F NMR (376 MHz, DMSO-*d*_6_) δ −109.2; IR (ATR) *v*_max_/cm^–1^ 2972w, 2914m, 2857m, 1686s, 1616s, 1425m, 1298s, 868m; MS­(ASAP): *m*/*z* = 426.1 [M + H]^+^; HRMS (ASAP) calcd. for C_23_H_25_N_3_O_2_SF [M + H]^+^: 426.1652, found 426.1646.

#### 3-Fluoro-4-[5-methyl-4-(5,5,8,8-tetramethyl-5,6,7,8-tetrahydroquinoxalin-2-yl)-1,3-thiazol-2-yl]­benzoic Acid, **43**

To a solution of compound **35** (0.38 g, 1.17 mmol) in anhydrous DMF (10 mL) under N_2_, was added compound **39** (0.30 g, 1.40 mmol) and the resultant solution was stirred at 110 °C for 24 h. The solution was cooled, diluted with H_2_O and extracted with EtOAc. The organics were washed with sat. NaHCO_3_, H_2_O, and brine, dried in (MgSO_4_), and evaporated to give a crude yellow solid. This was purified by SiO_2_ chromatography (9:1, heptane/EtOAc) to give the intermediate ester as a white solid (0.42 g, 82%). The ester (0.37 g, 0.84 mmol) was dissolved in THF (30 mL), 20% NaOH (3 mL) was added, and the resultant solution was stirred at reflux for 40 h. The mixture was cooled, acidified to pH 1 with 5% HCl, extracted with EtOAc, washed with H_2_O and brine, dried (MgSO_4_), and evaporated to give a crude white solid which was recrystallized from MeOH to give compound **43** as white solid (0.22 g, 60%): ^1^H NMR (700 MHz, DMSO-*d*_6_) δ 1.31 and 1.32 (s, 12H), 1.79 (s, 4H), 2.86 (s, 3H), 7.80 (dd, *J* = 11.6, 1.6 Hz, 1H), 7.88 (dd, *J* = 7.8, 1.6 Hz, 1H), 8.39 (t, *J* = 7.8 Hz, 1H), 9.12 (s, 1H), 13.43 (br, 1H)**;** ^13^C NMR (176 MHz, DMSO-*d*_6_) δ 13.05, 29.47, 29.58, 33.26, 33.37, 36.74, 36.94, 116.88 (d, *J* = 23.2 Hz), 123.85 (d, *J* = 11.4 Hz), 125.73 (d, *J* = 2.9 Hz), 128.17, 136.97 (d, *J* = 8.7 Hz), 140.48, 145.92, 146.12, 153.95 (d, *J* = 5.3 Hz), 155.67, 156.00, 158.70 (d, *J* = 250.8 Hz), 165.70; ^19^F NMR (376 MHz, DMSO-*d*_6_) δ −113.0; IR (ATR) *v*_max_/cm^–1^ 2971w, 2924m, 2860m, 1690s, 1618w, 1574w, 1414s, 1299m, 762s; MS­(ASAP): *m*/*z* = 426.1 [M + H]^+^; HRMS (ASAP) calcd. for C_23_H_25_N_3_O_2_SF [M + H]^+^: 426.1652, found 426.1649.

## Supplementary Material



## Notes

Synthesis of intermediates; ^1^H and ^13^C NMR spectra; fluorescence competition assay data; molecular docking and dynamics simulations; and multiple sequence alignments (PDF)

## Abbreviations

ATRA: all-*trans* retinoic acid
CI: confidence interval
CRABP: Cellular Retinoic Acid Binding Protein
DNA: deoxyribose nucleic acid
ESMACS: Enhanced Sampling of Molecular Dynamics with Approximation of Continuum Solvent
LBD: ligand-binding domain
LBP: ligand-binding pocket
MD: molecular dynamics
nM: nanomolar
PDB: Protein Data Bank
RA: retinoic acid
RAR: retinoic acid receptor
RNA: ribonucleic acid
RXR: retinoid X receptor
SD: standard deviation
