# Supplementary material for: Synthetic Retinoids for the Modulation of Genomic and Nongenomic Processes in Neurodegenerative Diseases
Source: ACS Omega. 2025 May 28;10(22):23709–38. doi: 10.1021/acsomega.5c00934 (PMC12163635; doi:10.1021/acsomega.5c00934)
Supplement: Supplementary file 1 [file ao5c00934_si_001.pdf]

Electronic supporting information

Synthetic retinoids for the modulation of genomic and non-genomic processes in neurodegenerative diseases

Abbey M. Butler,[a] David R. Chisholm,[a] Charles W.E. Tomlinson,[a] Thabat Khatib,[b,c] Jason Clark,[b] Shunzhou Wan,[d] Peter V. Coveney,[d,e,f] Iain R. Greig,[b] Peter McCaffery,[b] Ehmke Pohl,[a,g]* and Andrew Whiting[a]*

[a]Department of Chemistry, Durham University, South Road, Durham, DH1 3LE, United Kingdom

[b]Institute of Medical Sciences, University of Aberdeen, Foresterhill, Aberdeen, Scotland, AB25 2ZD, United Kingdom

[c]Health Sciences Department, Faculty of Modern Sciences, Arab American University, Ramallah, Palestine

[d]Centre for Computational Science, Department of Chemistry, University College London, 20 Gordon Street, London, WC1H 0AJ, United Kingdom

[e]Advanced Research Computing Centre, University College London, London, WC1H 0AJ, United Kingdom

[f]Institute for Informatics, Faculty of Science, University of Amsterdam, 1098XH Amsterdam, The Netherlands

[g]Department of Biosciences, Durham University, South Road, Durham, DH1 3LE, United Kingdom

Table of Contents

1. ^{1}H and ^{13}C NMR spectra 4

Figure. S1. Chemical structure and NMR spectra of 2,5-Dichloro-2,5-dimethylhexane, 1 4

Figure. S2. Chemical structure and NMR spectra of 6-Iodo-1,1,4,4,7-pentamethyl-1,2,3,4-tetrahydronaphthalene, 3b 5

Figure. S3. Chemical structure and NMR spectra of 6-Iodo-7-methoxy-1,1,4,4-tetramethyl-1,2,3,4-tetrahydronaphthalene, 3c 6

Figure. S4. Chemical structure and NMR spectra of 6-Ethynyl-1,1,4,4,7-pentamethyl-1,2,3,4-tetrahydronaphthalene, 4b 7

Figure. S5. Chemical structure and NMR spectra of 6-Ethynyl-7-methoxy-1,1,4,4-tetramethyl-1,2,3,4-tetrahydronaphthalene, 4c 8

Figure. S6. Chemical structure and NMR spectra of Trimethyl({3,3,6,6-tetramethyl-2-[(trimethylsilyl)oxy]cyclohex-1-en-1-yl}oxy)silane, 6 9

Figure. S7. Chemical structure and NMR spectra of 3,3,6,6-Tetramethylcyclohexane-1,2-dione, 7 10

Figure. S8. Chemical structure and NMR spectra of Methyl 5,5,8,8-tetramethyl-5,6,7,8-tetrahydroquinoxaline-2-carboxylate, 8 11

Figure. S9. Chemical structure and NMR spectra of 5,5,8,8-Tetramethyl-5,6,7,8-tetrahydroquinoxaline-2-carbaldehyde, 9 12

Figure. S10. Chemical structure and NMR spectra of 2-Ethynyl-5,5,8,8-tetramethyl-5,6,7,8-tetrahydroquinoxaline, 10 13

Figure. S11. Chemical structure and NMR spectra of Methyl 4-bromo-2-fluorobenzoate, 11b 14

Figure. S12. Chemical structure and NMR spectra of Methyl 4-bromo-3-fluorobenzoate, 11c 16

Figure. S13. Chemical structure and NMR spectra of Methyl 4-bromo-2,6-difluorobenzoate, 11d 18

Figure. S14. Chemical structure and NMR spectra of Methyl 4-bromo-3-chlorobenzoate, 11e 20

Figure. S15. Chemical structure and NMR spectra of Methyl 5-bromopyridine-2-carboxylate, 11f 22
Figure. S16. Chemical structure and NMR spectra of Methyl 6-bromopyridine-3-carboxylate, 11g 23
Figure. S17. Chemical structure and NMR spectra of Methyl 5-chloropyrazine-2-carboxylate, 11h 24
Figure. S18. Chemical structure and NMR spectra of Methyl 4-ethynyl-3-fluorobenzoate, 12a 25
Figure. S19. Chemical structure and NMR spectra of Methyl 4-ethynyl-2,6-difluorobenzoate, 12b 27
Figure. S20. Chemical structure and NMR spectra of Methyl 4-ethynyl-3-chlorobenzoate, 12c 29
Figure. S21. Chemical structure and NMR spectra of 2-Fluoro-4-[2-(5,5,8,8-tetramethyl-5,6,7,8-tetrahydronaphthalen-2-yl)ethynyl]benzoic acid, 13 30
Figure. S22. Chemical structure and NMR spectra of 3-Fluoro-4-[2-(5,5,8,8-tetramethyl-5,6,7,8-tetrahydronaphthalen-2-yl)ethynyl]benzoic acid, 14 32
Figure. S23. Chemical structure and NMR spectra of 2,6-Difluoro-4-[2-(5,5,8,8-tetramethyl-5,6,7,8-tetrahydronaphthalen-2-yl)ethynyl]benzoic acid, 15 34
Figure. S24. Chemical structure and NMR spectra of 3-Chloro-4-[2-(5,5,8,8-tetramethyl-5,6,7,8-tetrahydronaphthalen-2-yl)ethynyl]benzoic acid, 16 36
Figure. S25. Chemical structure and NMR spectra of 2-Fluoro-4-[2-(3,5,5,8,8-pentamethyl-5,6,7,8-tetrahydronaphthalen-2-yl)ethynyl]benzoic acid, 17 37
Figure. S26. Chemical structure and NMR spectra of 3-Fluoro-4-[2-(3,5,5,8,8-pentamethyl-5,6,7,8-tetrahydronaphthalen-2-yl)ethynyl]benzoic acid, 18 39
Figure. S27. Chemical structure and NMR spectra of 2,6-Difluoro-4-[2-(3,5,5,8,8-pentamethyl-5,6,7,8-tetrahydronaphthalen-2-yl)ethynyl]benzoic acid, 19 41
Figure. S28. Chemical structure and NMR spectra of 2-Fluoro-4-[2-(3-methoxy-5,5,8,8-tetramethyl-5,6,7,8-tetrahydronaphthalen-2-yl)ethynyl]benzoic acid, 20 43
Figure. S29. Chemical structure and NMR spectra of 3-Fluoro-4-[2-(3-methoxy-5,5,8,8-tetramethyl-5,6,7,8-tetrahydronaphthalen-2-yl)ethynyl]benzoic acid, 21 45
Figure. S30. Chemical structure and NMR spectra of 2,6-Difluoro-4-[2-(3-methoxy-5,5,8,8-tetramethyl-5,6,7,8-tetrahydronaphthalen-2-yl)ethynyl]benzoic acid, 22 47
Figure. S31. Chemical structure and NMR spectra of 6-[2-(5,5,8,8-Tetramethyl-5,6,7,8-tetrahydronaphthalen-2-yl)ethynyl]pyridine-3-carboxylic acid, 23 49
Figure. S32. Chemical structure and NMR spectra of 5-[2-(5,5,8,8-Tetramethyl-5,6,7,8-tetrahydronaphthalen-2-yl)ethynyl]pyridine-2-carboxylic acid, 24 50
Figure. S33. Chemical structure and NMR spectra of 5-[2-(5,5,8,8-Tetramethyl-5,6,7,8-tetrahydronaphthalen-2-yl)ethynyl]pyrazine-2-carboxylic acid, 25 51
Figure. S34. Chemical structure and NMR spectra of 6-[2-(3,5,5,8,8-Pentamethyl-5,6,7,8-tetrahydronaphthalen-2-yl)ethynyl]pyridine-3-carboxylic acid, 26 53
Figure. S35. Chemical structure and NMR spectra of 6-[2-(3-Methoxy-5,5,8,8-tetramethyl-5,6,7,8-tetrahydronaphthalen-2-yl)ethynyl]pyridine-3-carboxylic acid, 27 54
Figure. S36. Chemical structure and NMR spectra of 2-[2-(3,5,5,8,8-Pentamethyl-5,6,7,8-tetrahydronaphthalen-2-yl)ethynyl]pyrimidine-5-carboxylic acid, 28 55
Figure. S37. Chemical structure and NMR spectra of 2-[2-(3-Methoxy-5,5,8,8-tetramethyl-5,6,7,8-tetrahydronaphthalen-2-yl)ethynyl]pyrimidine-5-carboxylic acid, 29 56

Figure. S38. Chemical structure and NMR spectra of 4-[2-(5,5,8,8-Tetramethyl-5,6,7,8-tetrahydroquinoxalin-2-yl)ethynyl]benzoic acid, 30 57

Figure. S39. Chemical structure and NMR spectra of 3-Fluoro-4-[2-(5,5,8,8-tetramethyl-5,6,7,8-tetrahydroquinoxalin-2-yl)ethynyl]benzoic acid, 31 58

Figure. S40. Chemical structure and NMR spectra of 1-(5,5,8,8-Tetramethyl-5,6,7,8-tetrahydroquinoxalin-2-yl)propan-1-ol, 32 60

Figure. S41. Chemical structure and NMR spectra of 1-(5,5,8,8-Tetramethyl-5,6,7,8-tetrahydroquinoxalin-2-yl)propan-1-one, 34 61

Figure. S42. Chemical structure and NMR spectra of 2-Bromo-1-(5,5,8,8-tetramethyl-5,6,7,8-tetrahydroquinoxalin-2-yl)propan-1-one, 35 62

Figure. S43. Chemical structure and NMR spectra of Methyl 4-carbamothioylbenzoate, 36 63

Figure. S44. Chemical structure and NMR spectra of Methyl 6-carbamothioylpyridine-3-carboxylate, 37 64

Figure. S45. Chemical structure and NMR spectra of Methyl 4-carbamothioyl-2-fluorobenzoate, 38 65

Figure. S46. Chemical structure and NMR spectra of Methyl 4-carbamothioyl-3-fluorobenzoate, 39 67

Figure. S47. Chemical structure and NMR spectra of 4-[5-Methyl-4-(5,5,8,8-tetramethyl-5,6,7,8-tetrahydroquinoxalin-2-yl)-1,3-thiazol-2-yl]benzoic acid, 40 69

Figure. S48. Chemical structure and NMR spectra of 6-[5-Methyl-4-(5,5,8,8-tetramethyl-5,6,7,8-tetrahydroquinoxalin-2-yl)-1,3-thiazol-2-yl]pyridine-3-carboxylic acid, 41 70

Figure. S49. Chemical structure and NMR spectra of 2-Fluoro-4-[5-methyl-4-(5,5,8,8-tetramethyl-5,6,7,8-tetrahydroquinoxalin-2-yl)-1,3-thiazol-2-yl]benzoic acid, 42 71

Figure. S50. Chemical structure and NMR spectra of 3-Fluoro-4-[5-methyl-4-(5,5,8,8-tetramethyl-5,6,7,8-tetrahydroquinoxalin-2-yl)-1,3-thiazol-2-yl]benzoic acid, 43 73

Figure. S51. Chemical structure and NMR spectra of methyl 6-cyanopyridine-3-carboxylate 75

3. Fluorescence competition assays 76

Table S1. Binding affinities between synthetic retinoids and RARα and RARγ. 76

4. Molecular docking and dynamics simulations 77

Table S2. ChemScores and binding free energies between synthetic retinoids and RARα 77

Table S3. ChemScores and binding free energies between synthetic retinoids and RARβ 78

Table S4. ChemScores and binding free energies between synthetic retinoids and RARγ. 79

5. Multiple sequence alignments 80

Figure S50. Multiple sequence alignment of human Retinoic Acid Receptors, RARα$^{182-417}$, RARβ$^{182-417}$ and RARγ$^{182-417}$ 80

7. References 81

1. ^{1}H and ^{13}C NMR spectra

Figure. S1. Chemical structure and NMR spectra of 2,5-Dichloro-2,5-dimethylhexane, 1

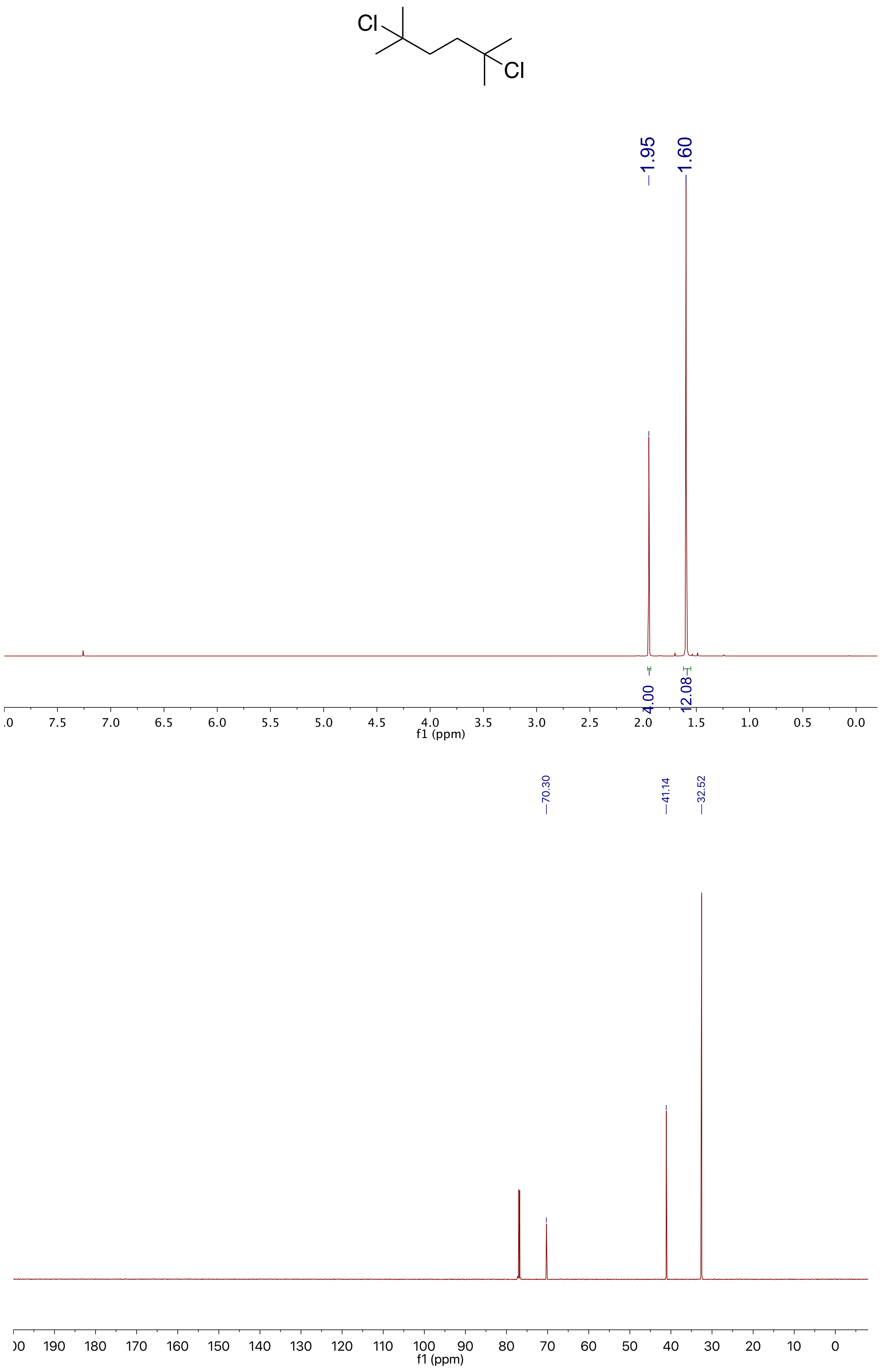

Figure. S2. Chemical structure and NMR spectra of 6-Iodo-1,1,4,4,7-pentamethyl-1,2,3,4-tetrahydronaphthalene, 3b

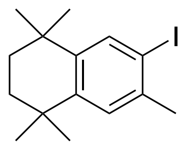

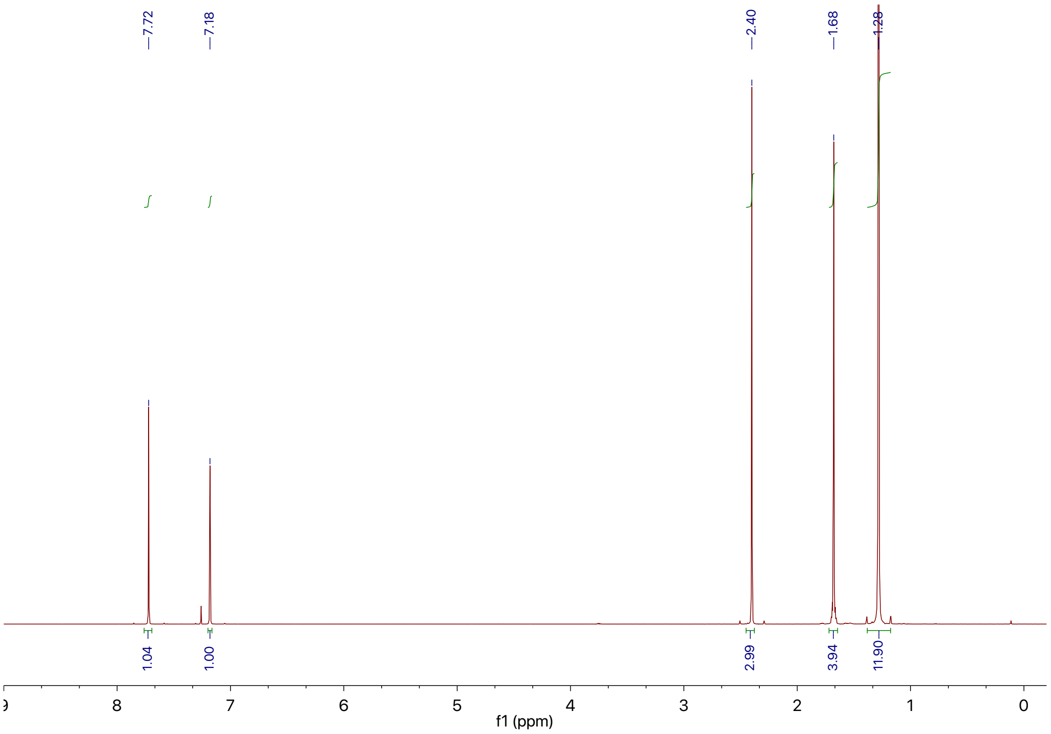

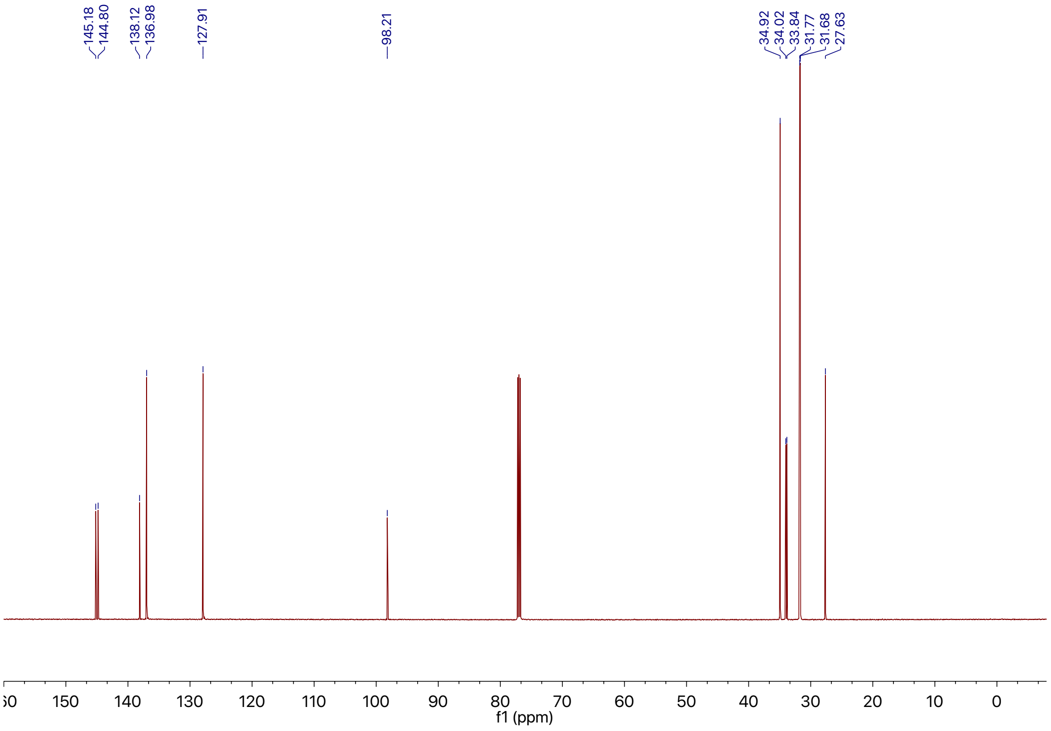

Figure. S3. Chemical structure and NMR spectra of 6-Iodo-7-methoxy-1,1,4,4-tetramethyl-1,2,3,4-tetrahydronaphthalene, 3c

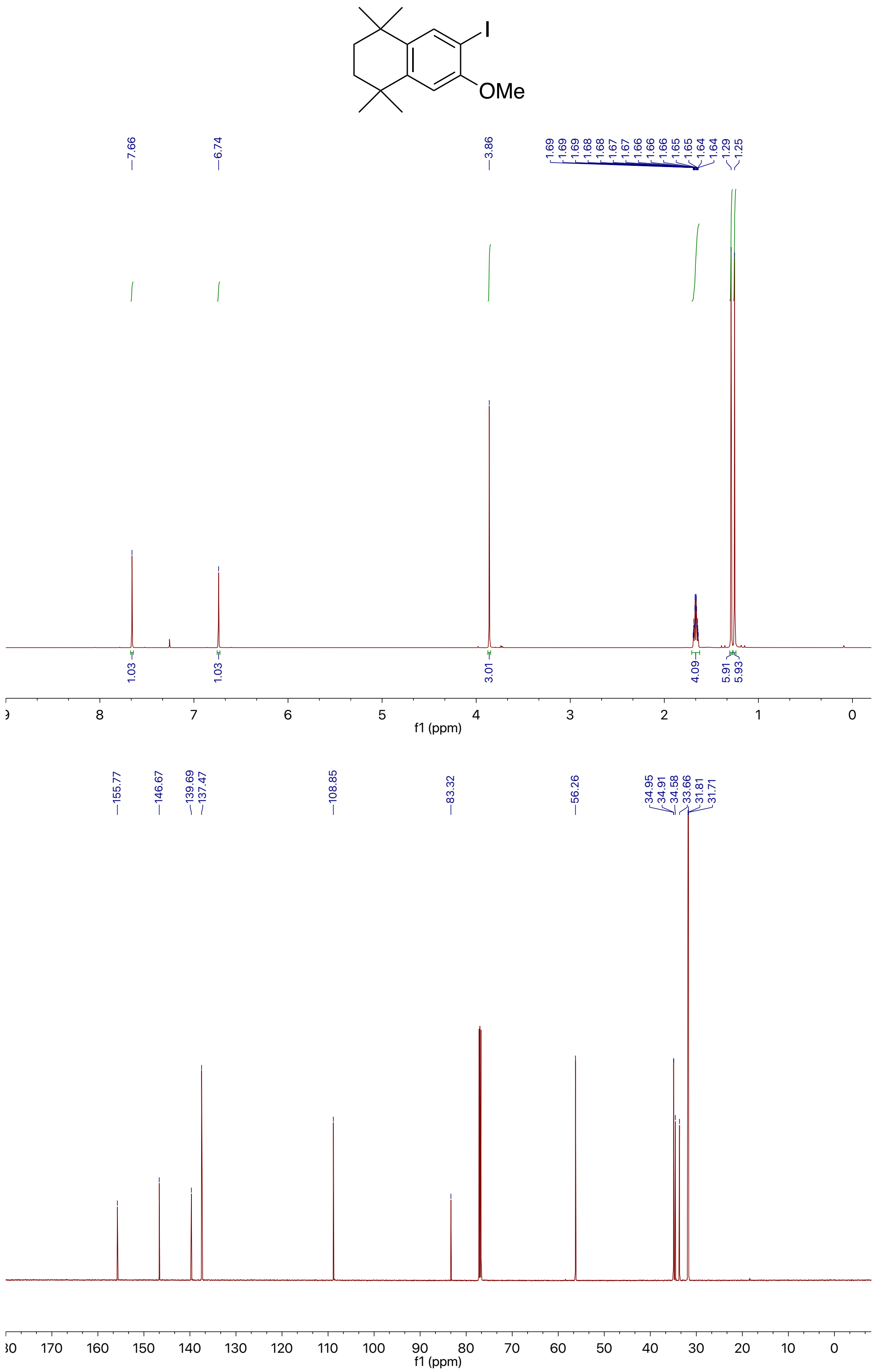

Figure. S4. Chemical structure and NMR spectra of 6-Ethynyl-1,1,4,4,7-pentamethyl-1,2,3,4-tetrahydronaphthalene, 4b

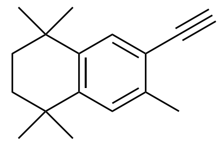

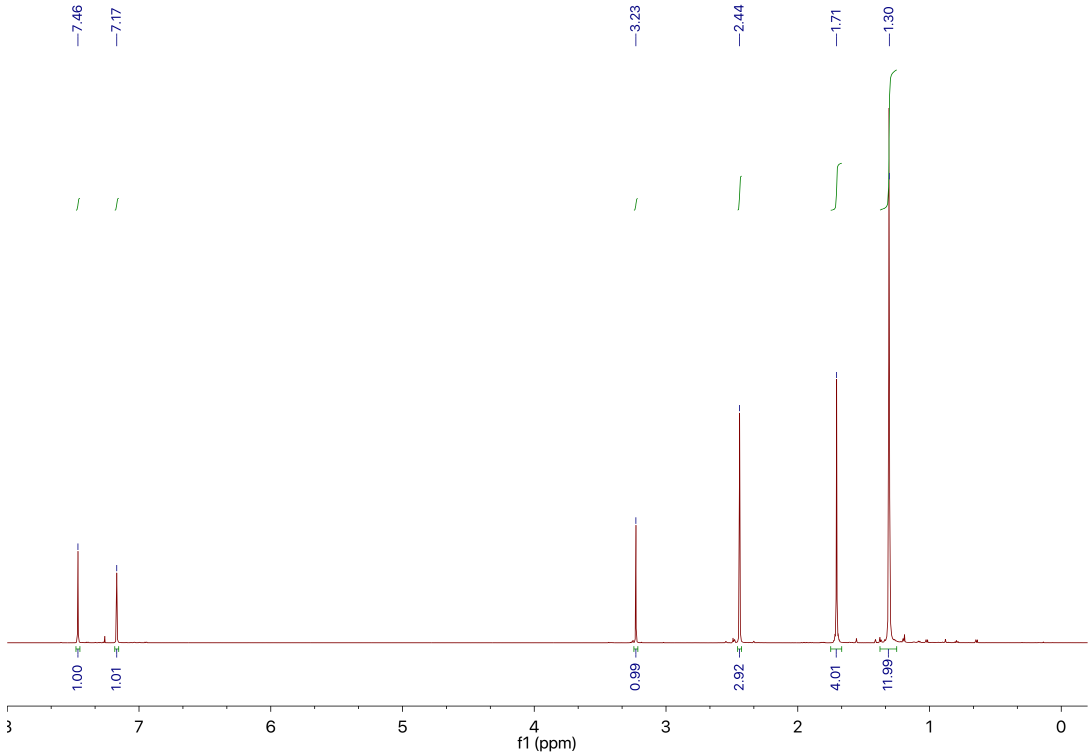

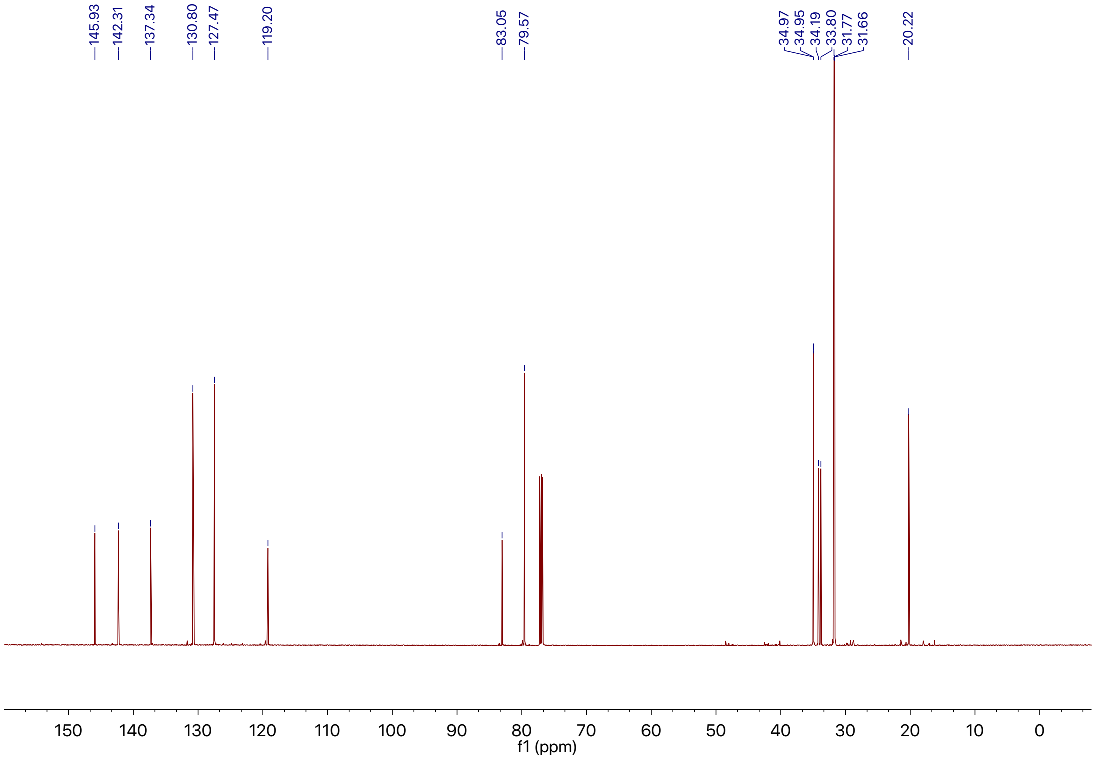

Figure. S5. Chemical structure and NMR spectra of 6-Ethynyl-7-methoxy-1,1,4,4-tetramethyl-1,2,3,4-tetrahydronaphthalene, 4c

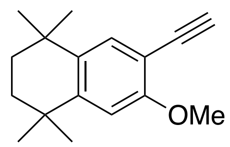

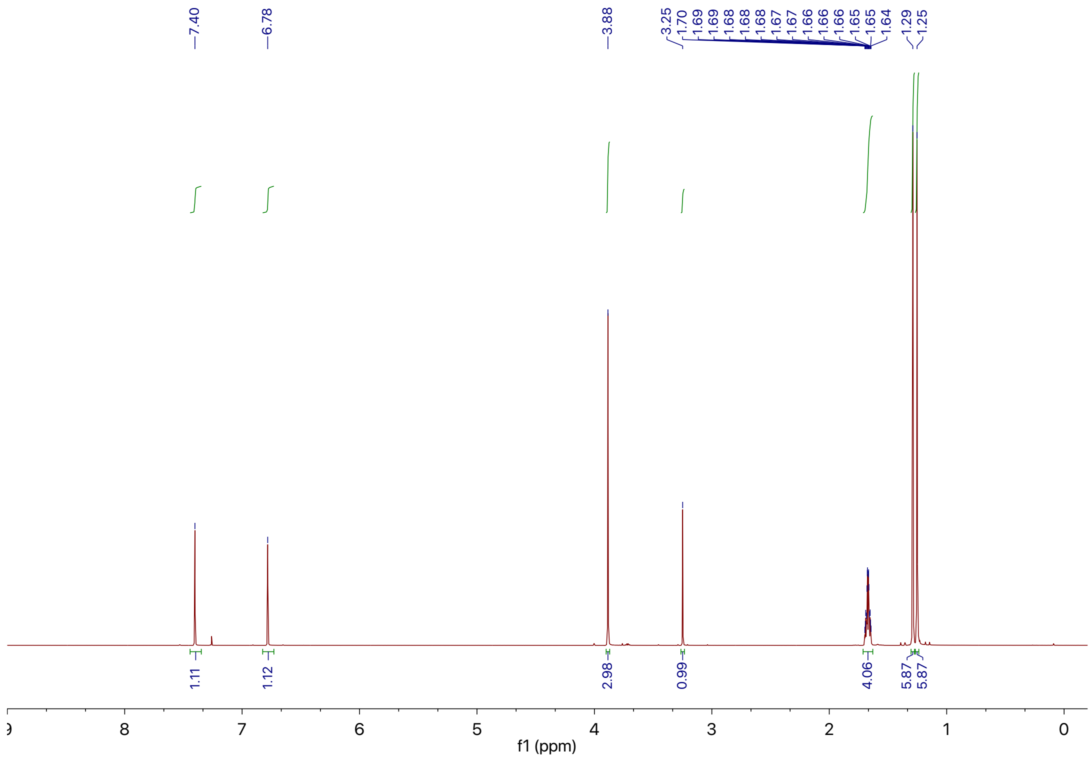

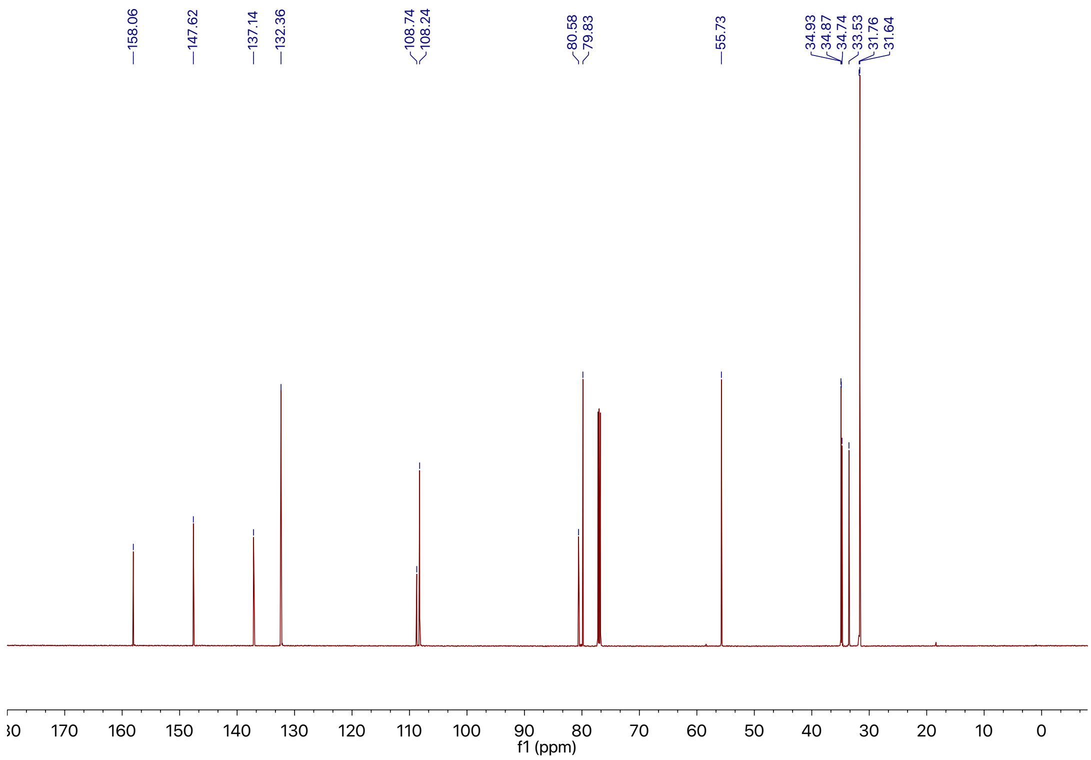

Figure. S6. Chemical structure and NMR spectra of Trimethyl({3,3,6,6-tetramethyl-2-[(trimethylsilyl)oxy]cyclohex-1-en-1-yl}oxy)silane, 6

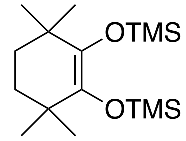

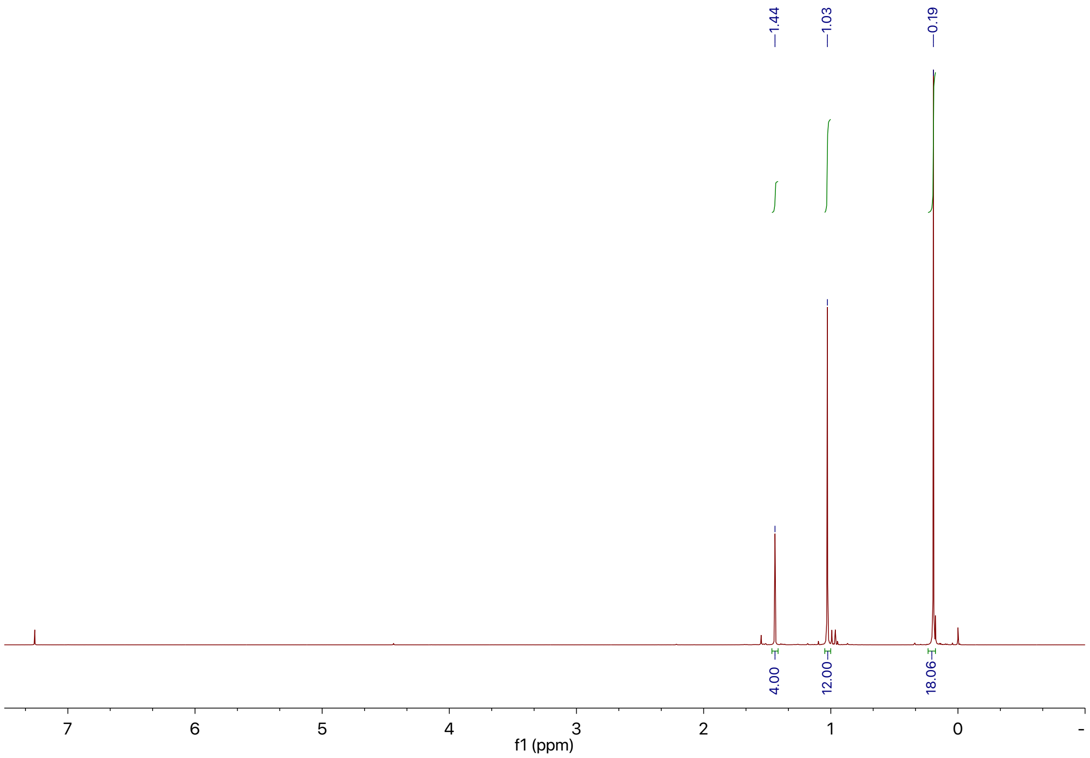

Figure. S7. Chemical structure and NMR spectra of 3,3,6,6-Tetramethylcyclohexane-1,2-dione, 7

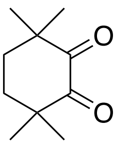

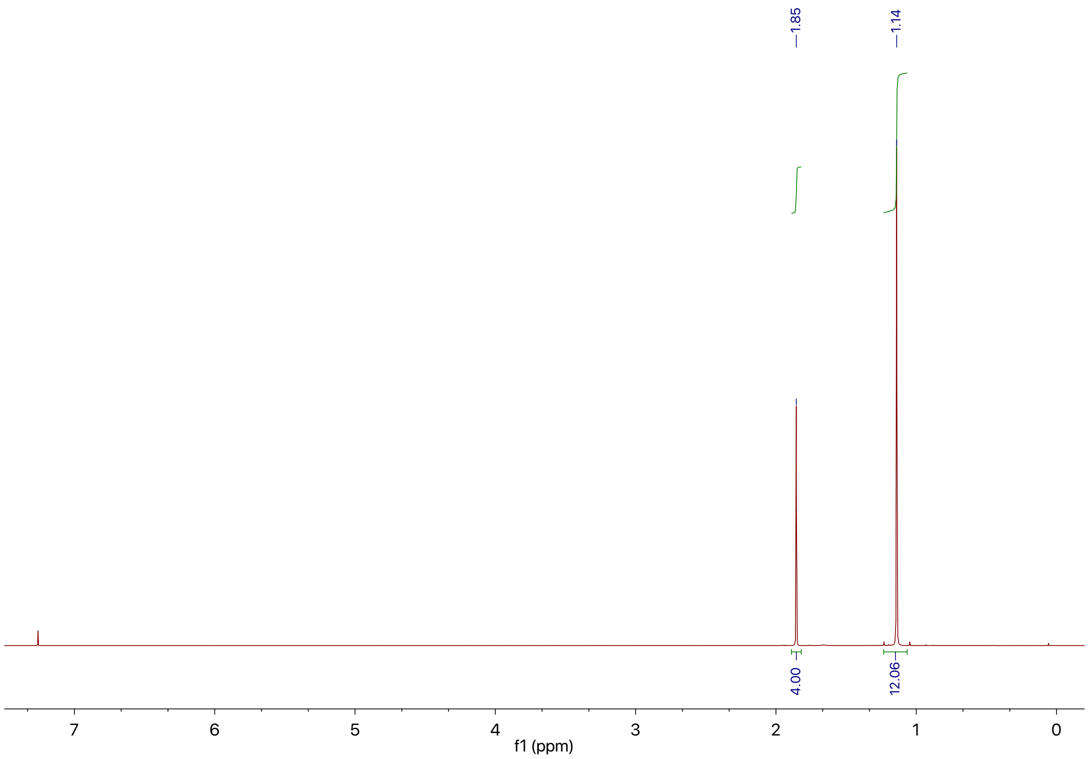

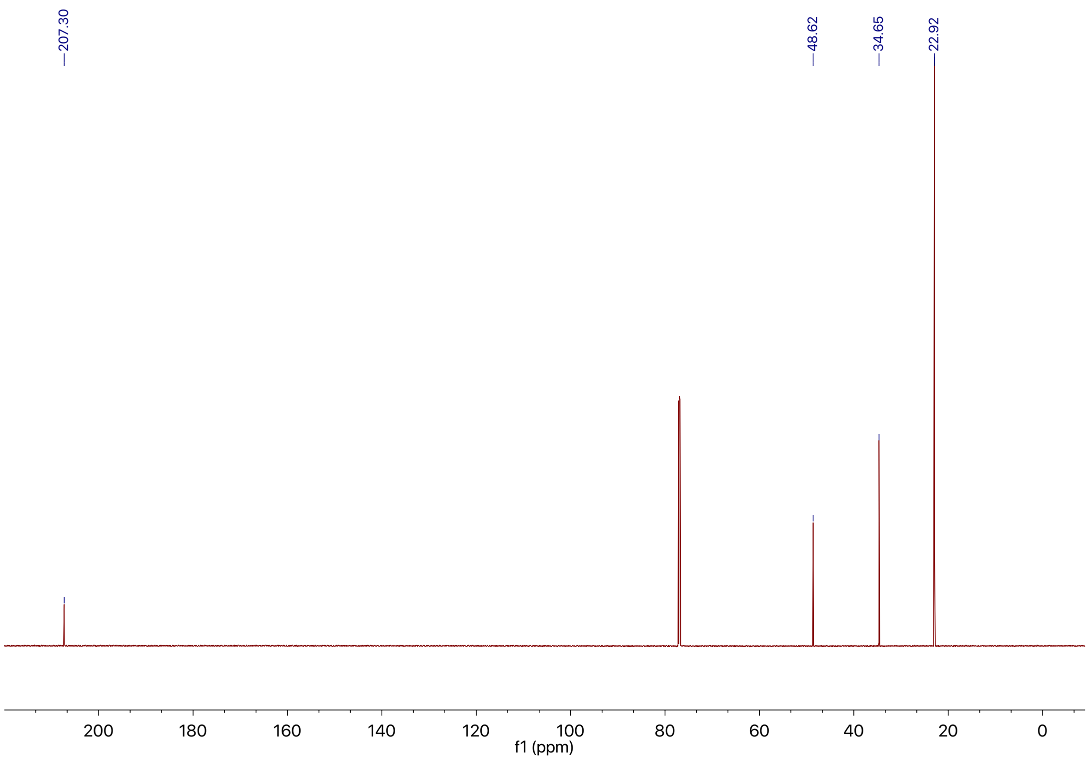

Figure. S8. Chemical structure and NMR spectra of Methyl 5,5,8,8-tetramethyl-5,6,7,8-tetrahydroquinoxaline-2-carboxylate, 8

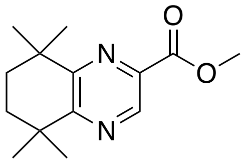

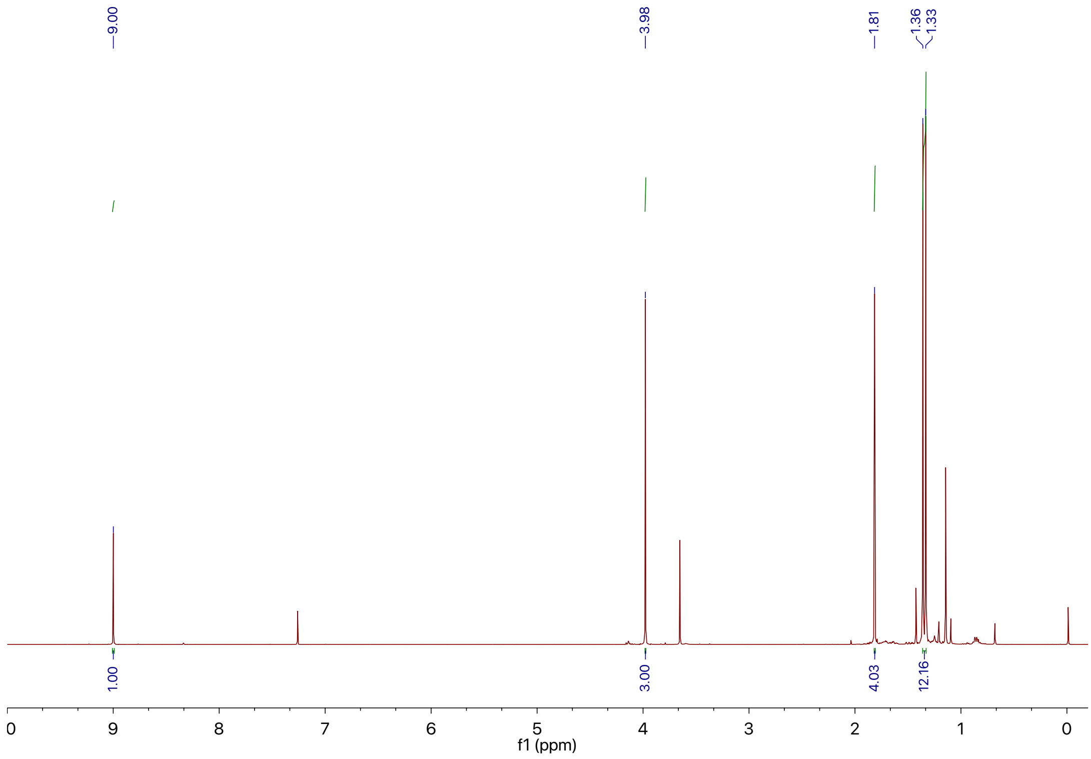

Figure. S9. Chemical structure and NMR spectra of 5,5,8,8-Tetramethyl-5,6,7,8-tetrahydroquinoxaline-2-carbaldehyde, 9

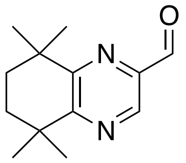

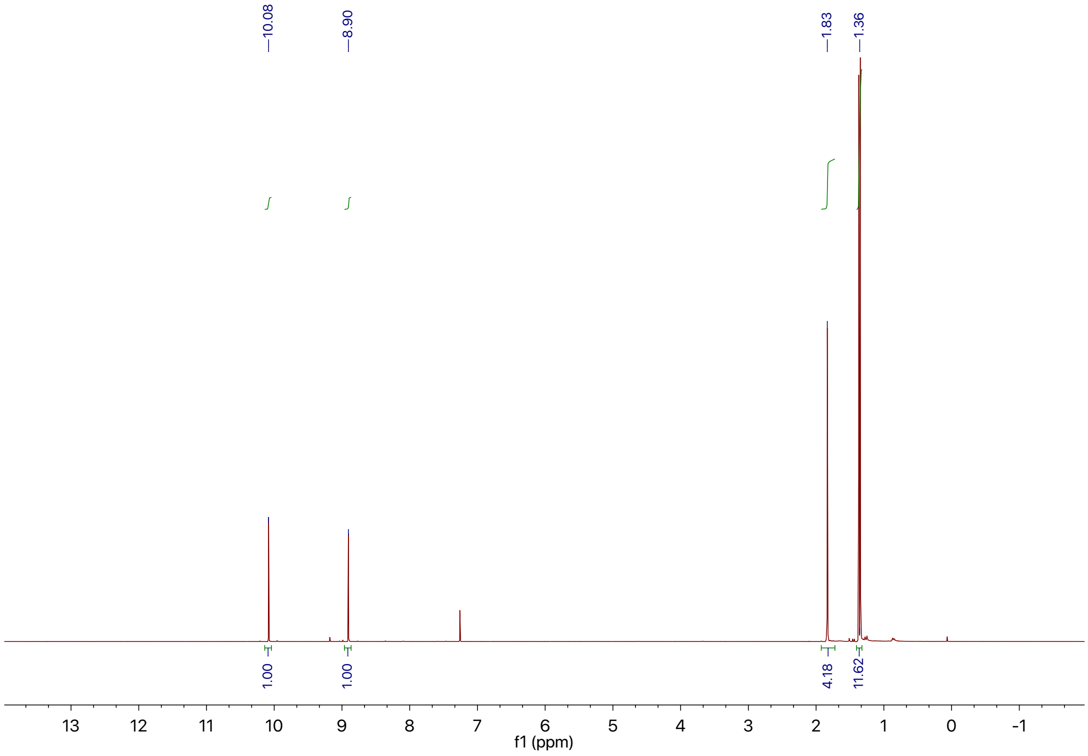

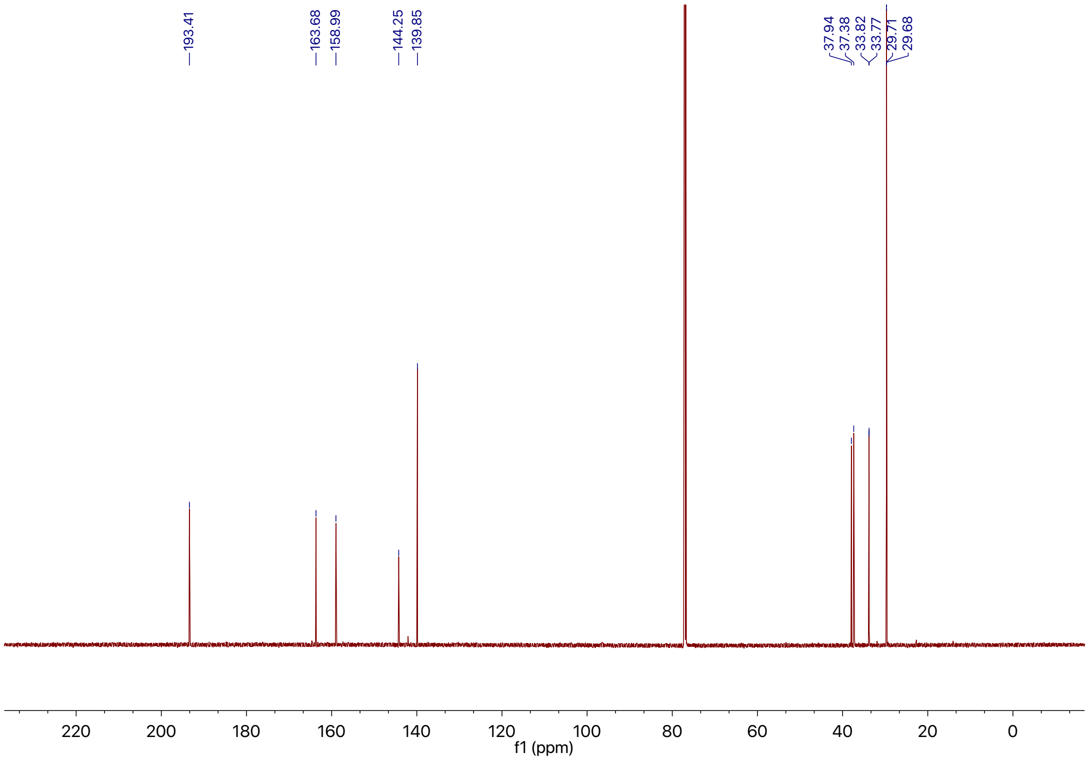

Figure. S10. Chemical structure and NMR spectra of 2-Ethynyl-5,5,8,8-tetramethyl-5,6,7,8-tetrahydroquinoxaline, 10

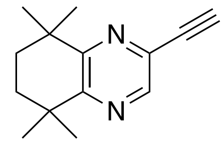

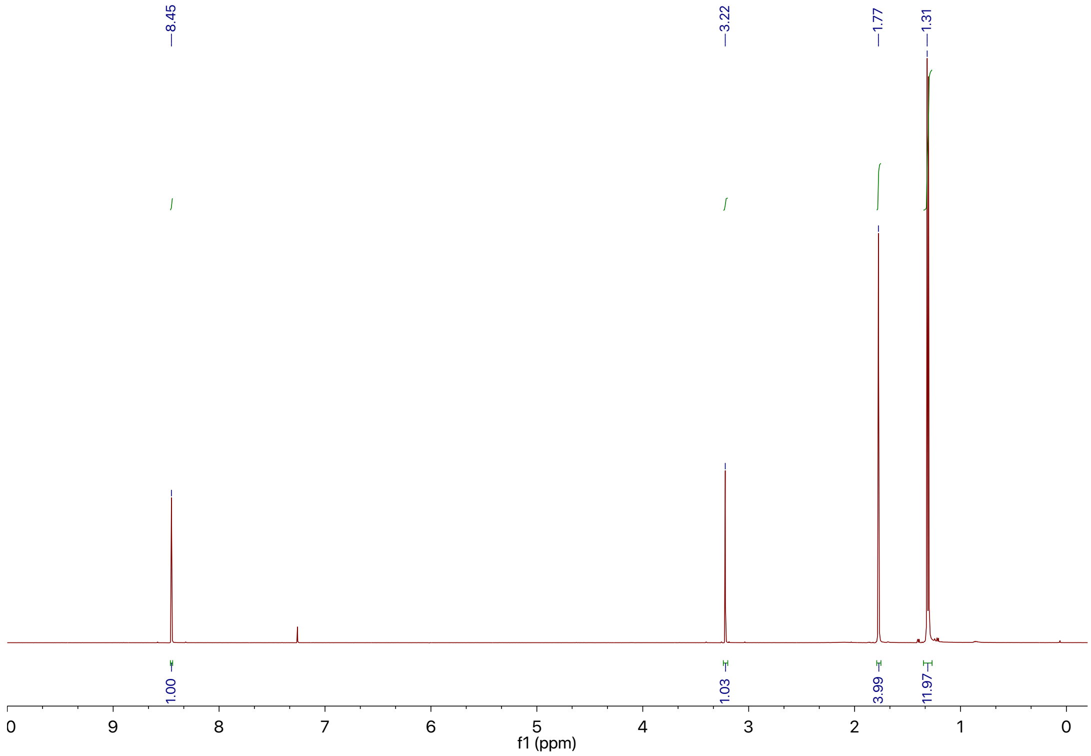

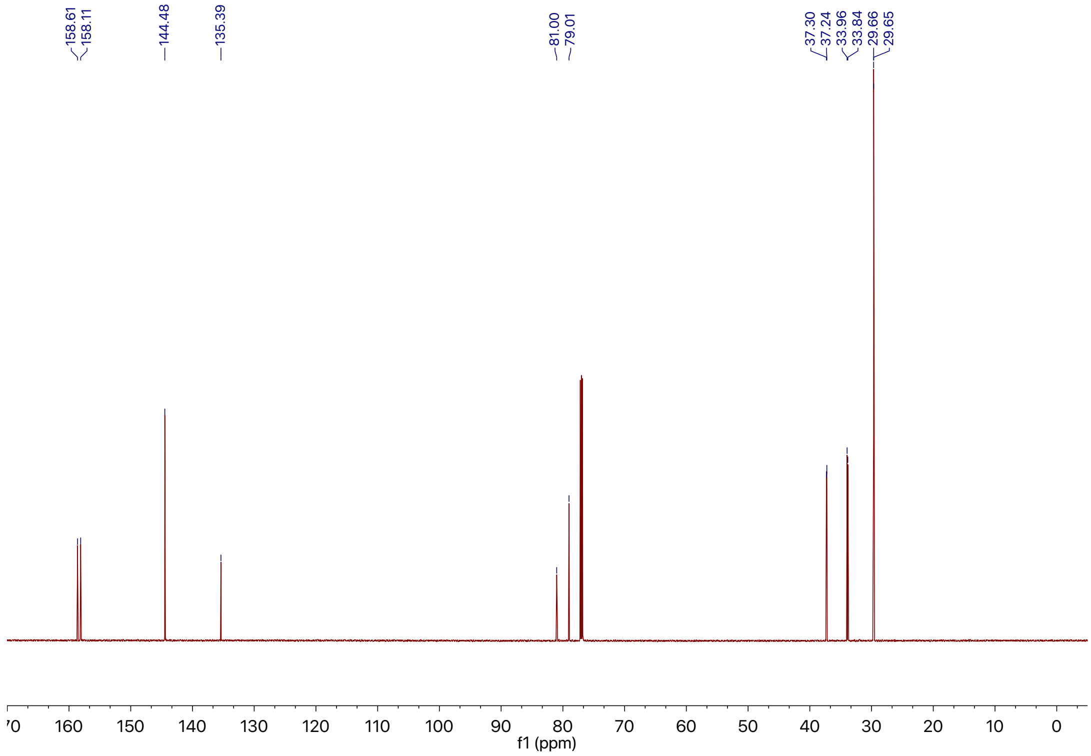

Figure. S11. Chemical structure and NMR spectra of Methyl 4-bromo-2-fluorobenzoate, 11b

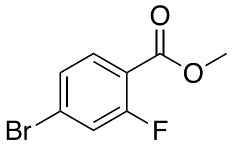

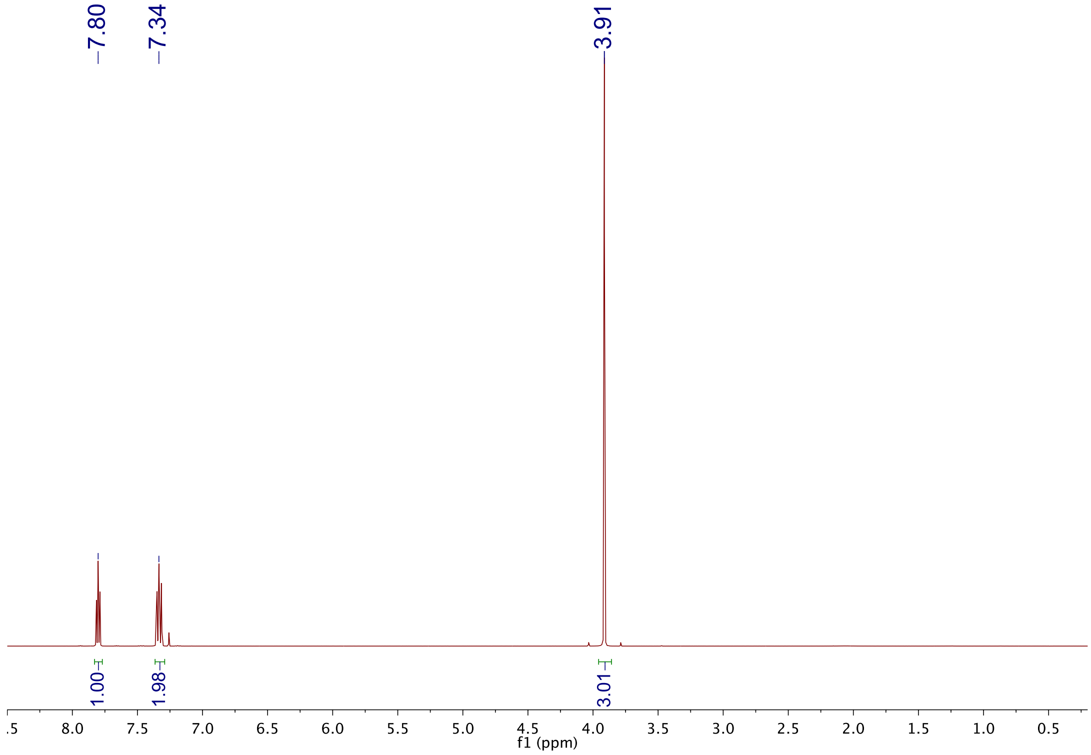

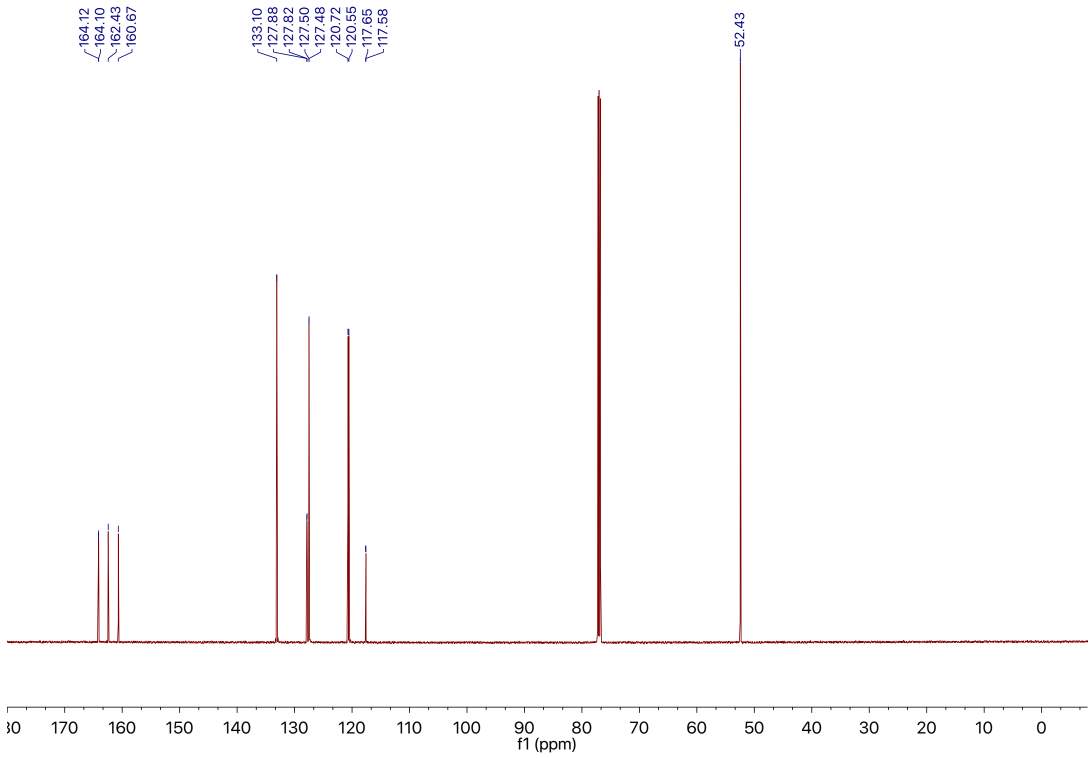

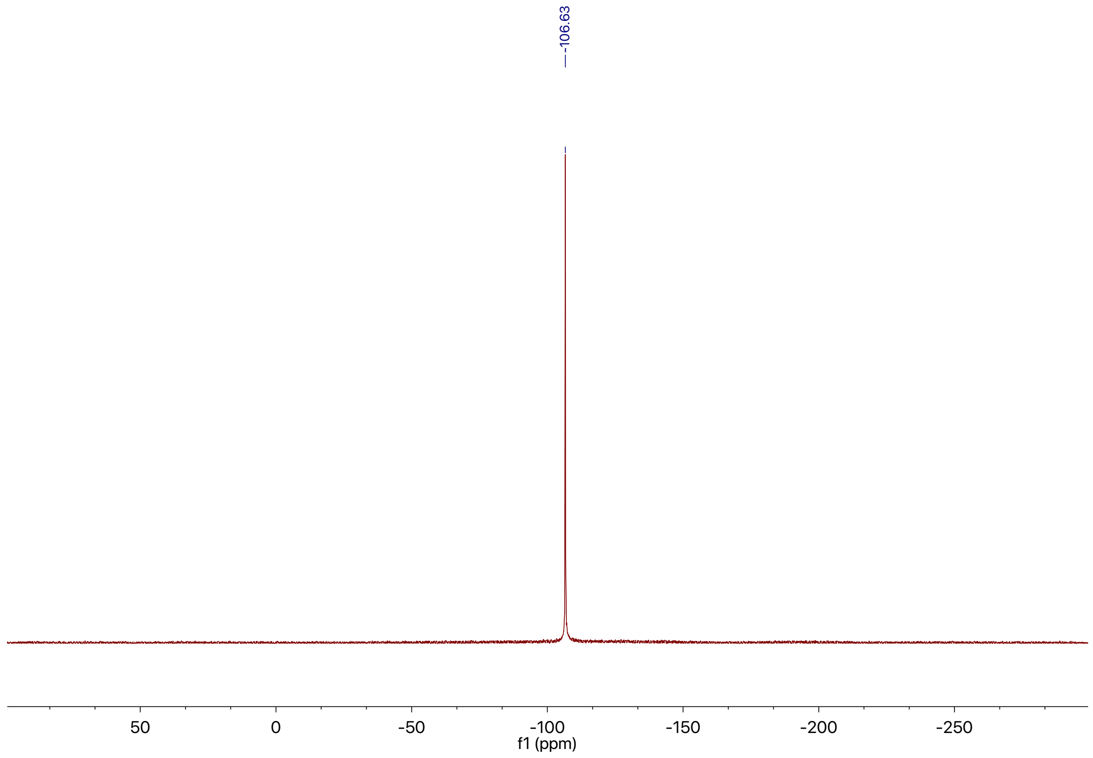
-106.63
50
0
-50
-100
-150
-200
-250
f1 (ppm)

Figure. S12. Chemical structure and NMR spectra of Methyl 4-bromo-3-fluorobenzoate, 11c

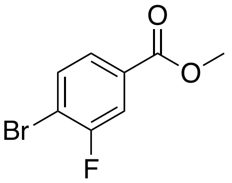

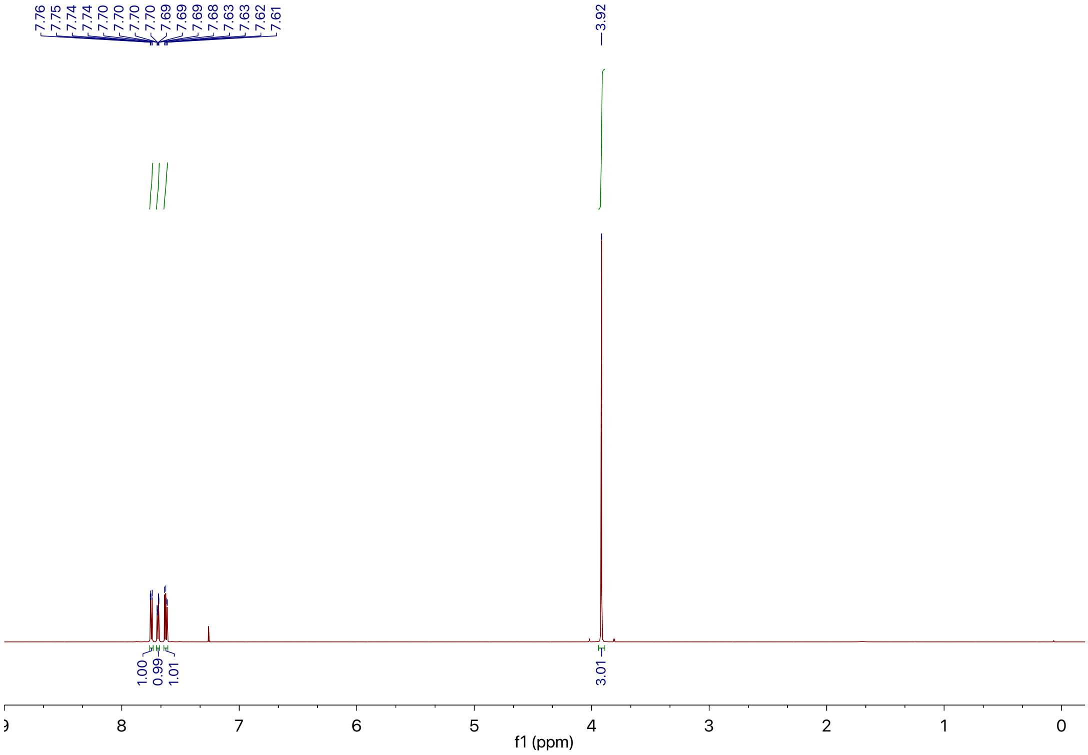

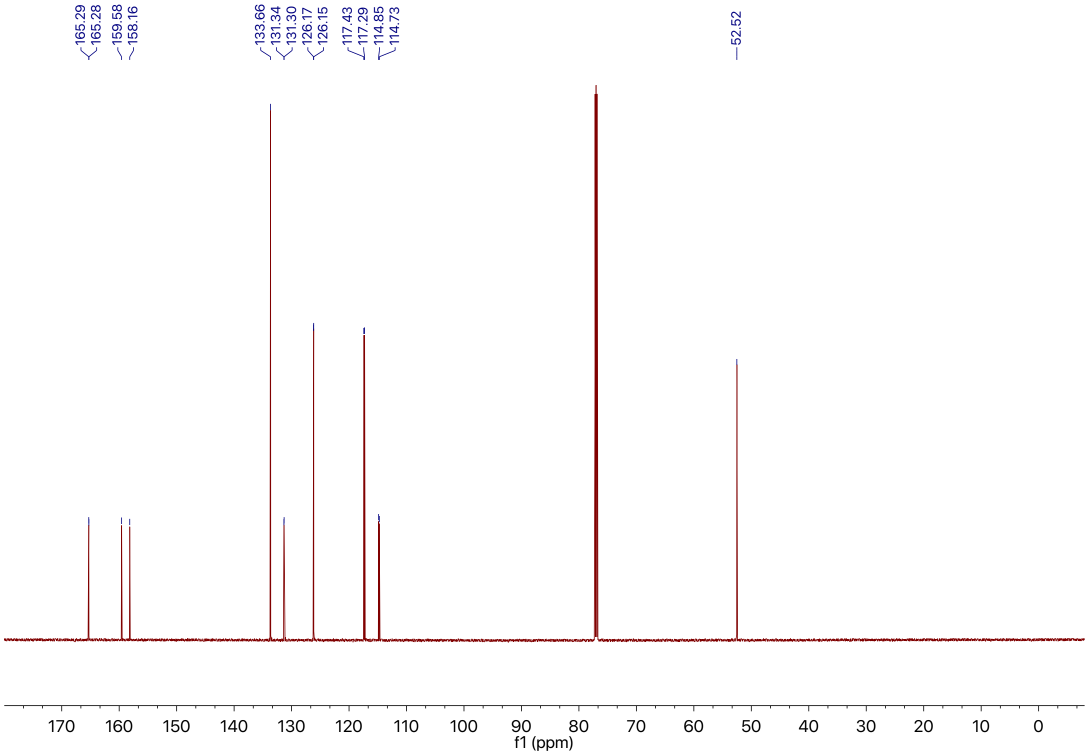

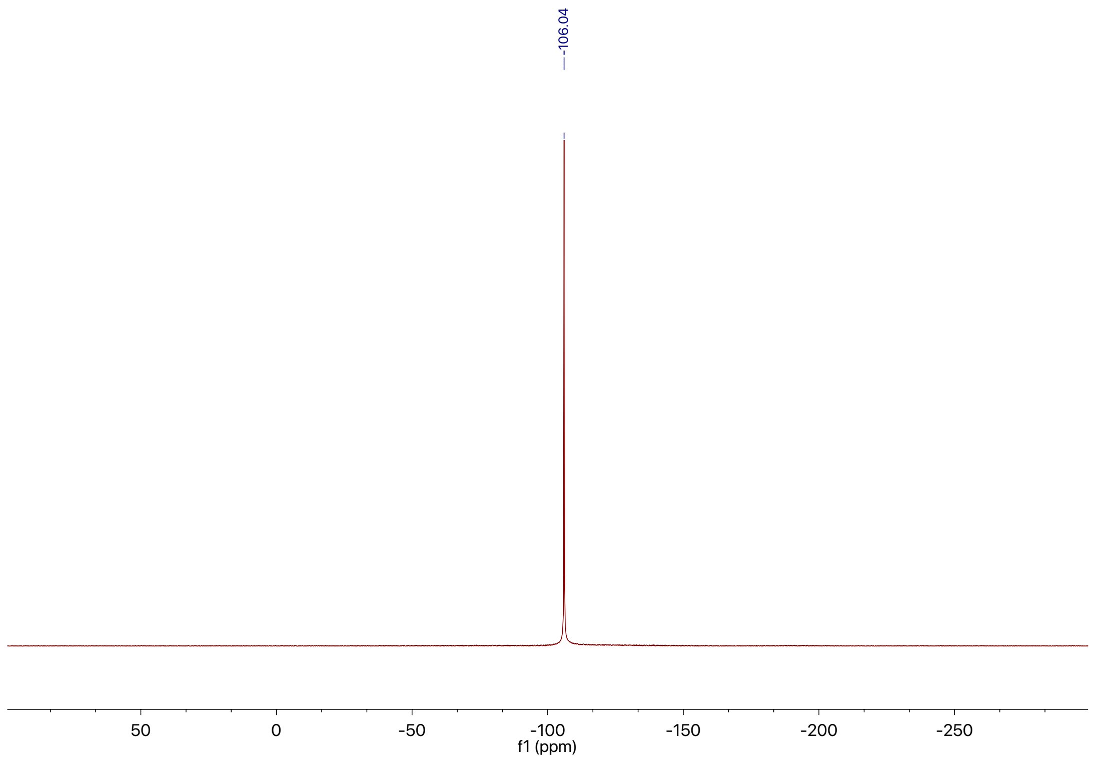
-106.04
50
0
-50
-100
-150
-200
-250
f1 (ppm)

Figure. S13. Chemical structure and NMR spectra of Methyl 4-bromo-2,6-difluorobenzoate, 11d

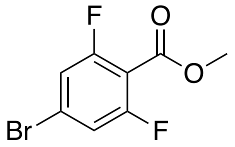

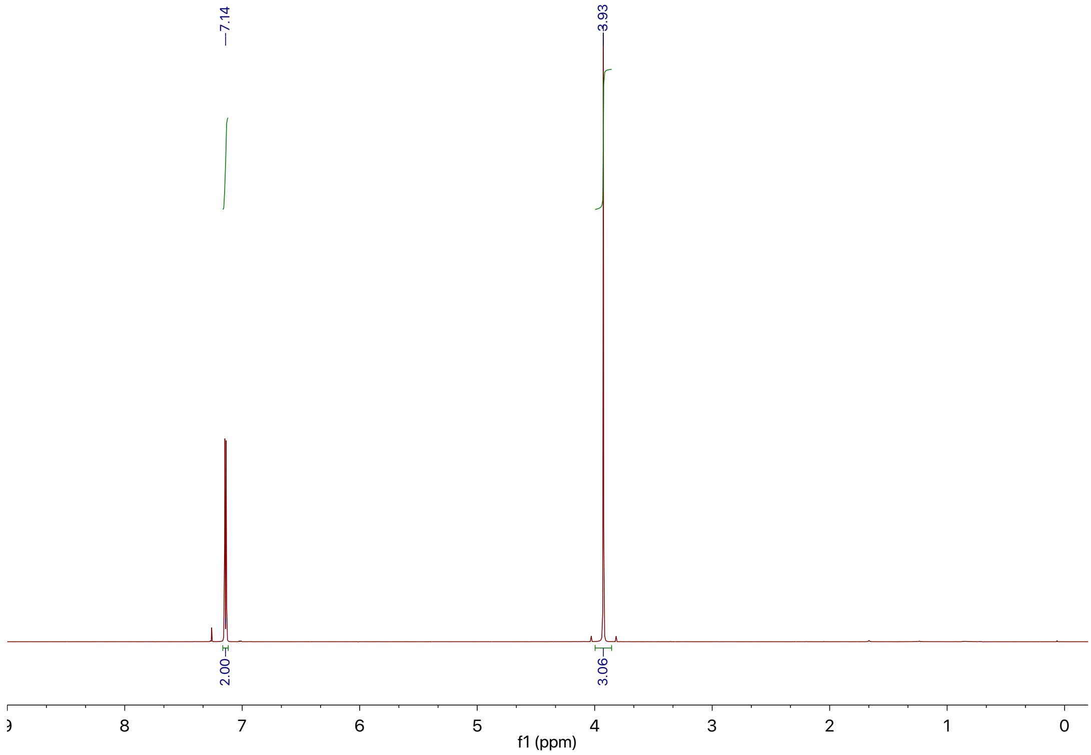

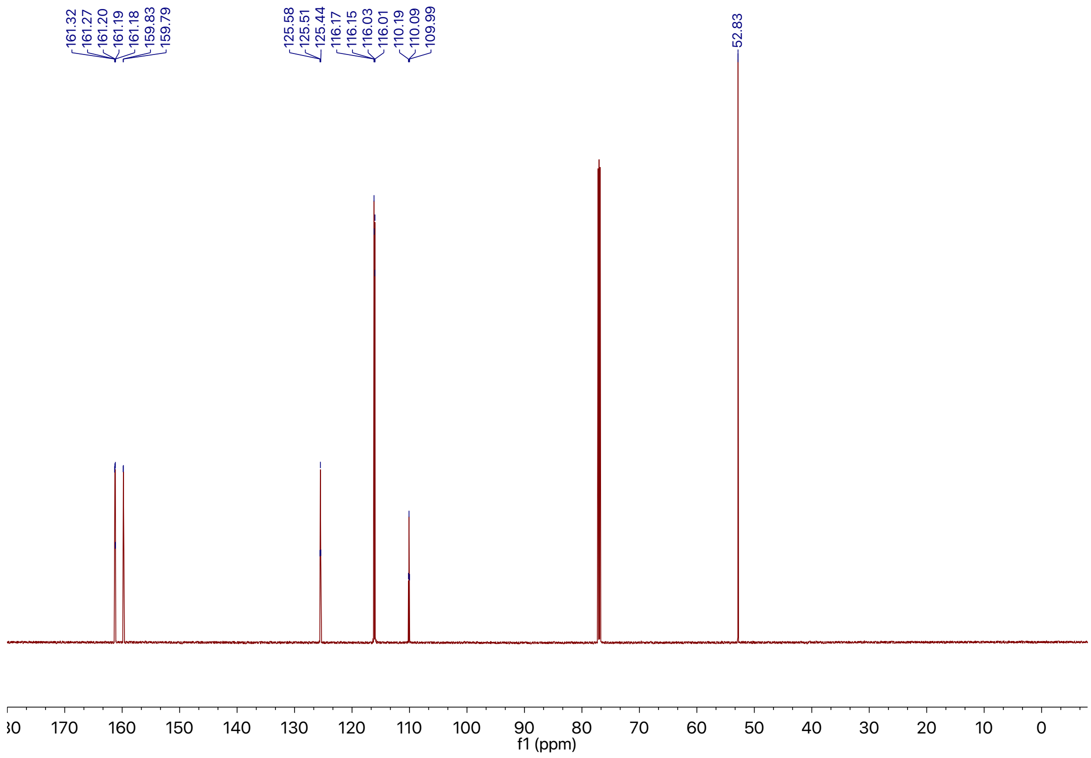

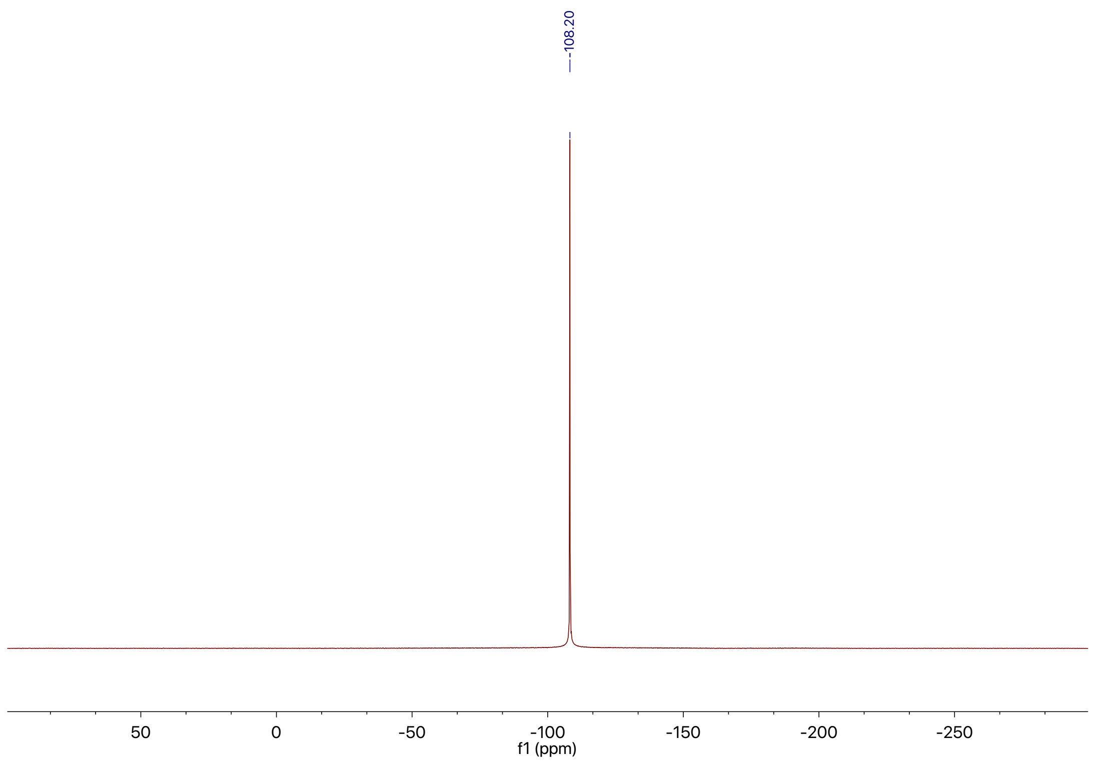
-108.20
50
0
-50
-100
-150
-200
-250
f1 (ppm)

Figure. S14. Chemical structure and NMR spectra of Methyl 4-bromo-3-chlorobenzoate, 11e

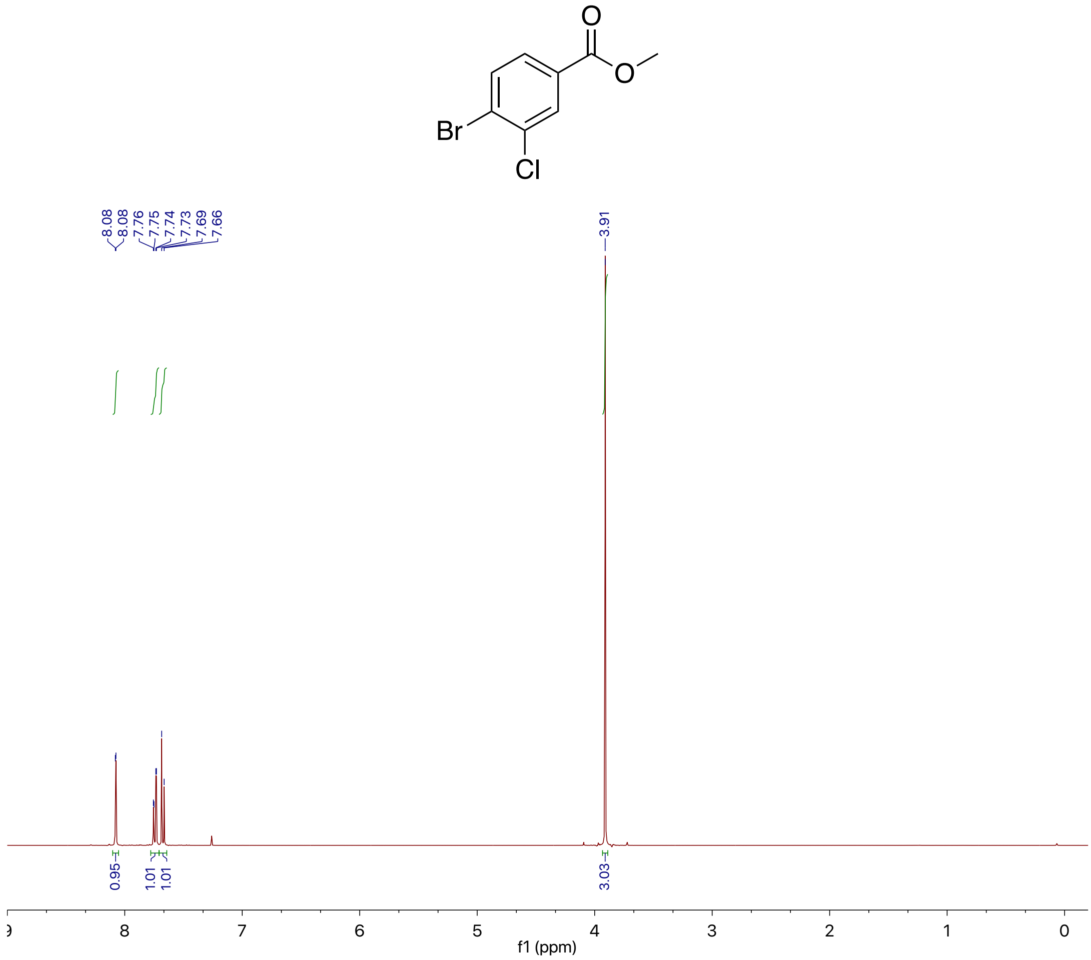

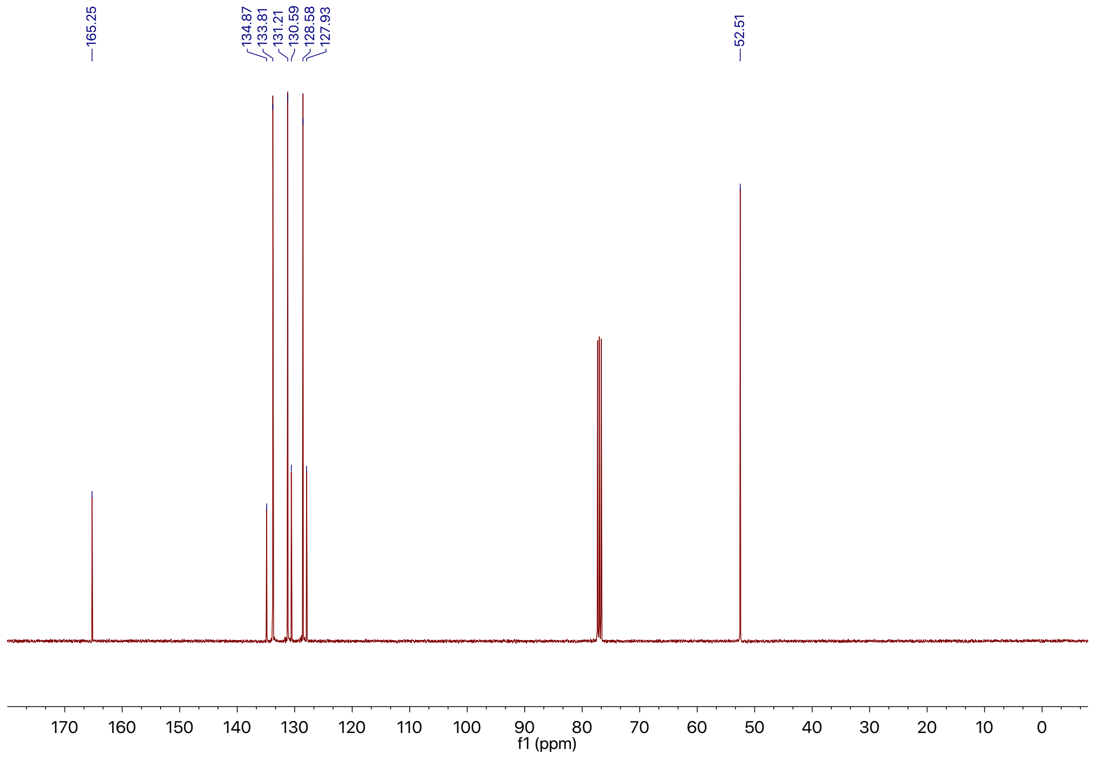

165.25
134.87
133.81
131.21
130.59
128.58
127.93
52.51
170 160 150 140 130 120 110 100 90 80 70 60 50 40 30 20 10 0
f1 (ppm)

Figure. S15. Chemical structure and NMR spectra of Methyl 5-bromopyridine-2-carboxylate, 11f

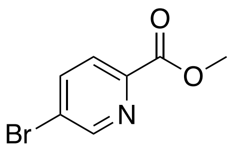

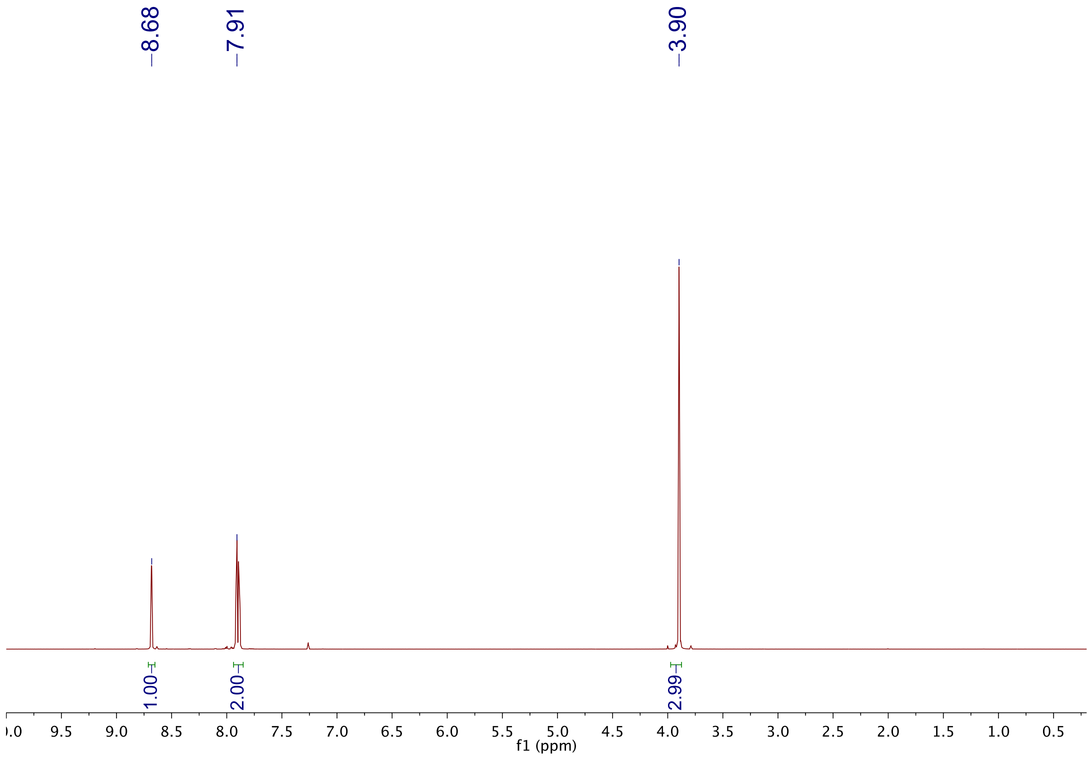

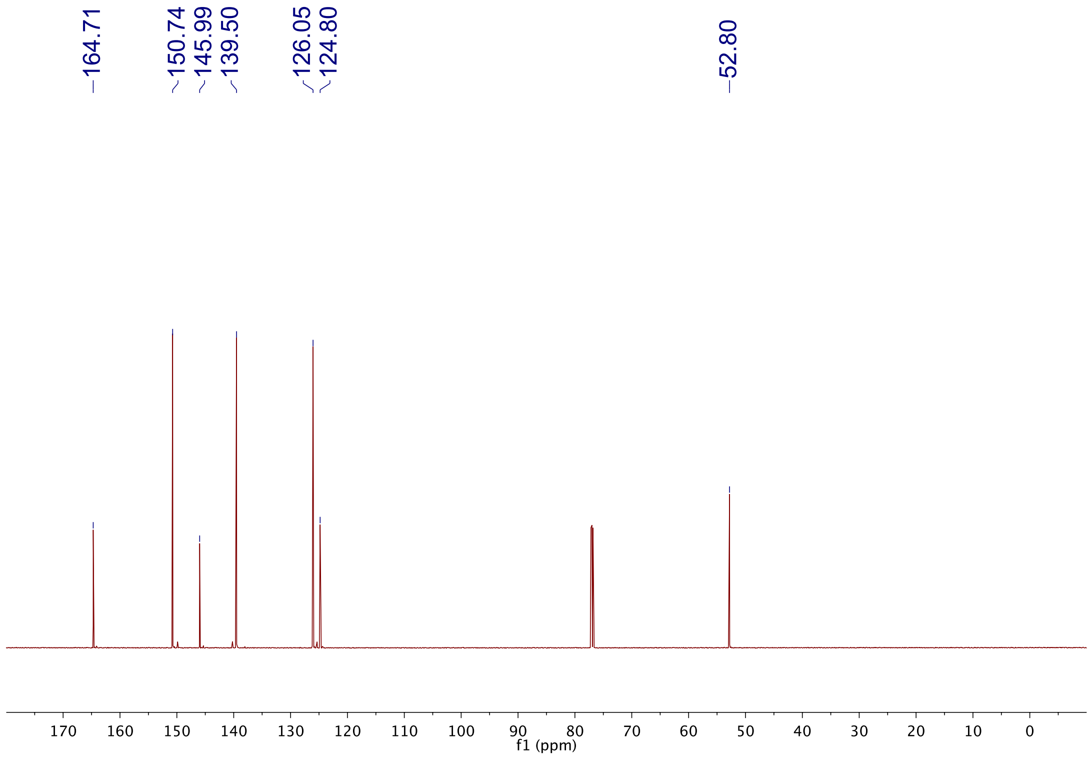

Figure. S16. Chemical structure and NMR spectra of Methyl 6-bromopyridine-3-carboxylate, 11g

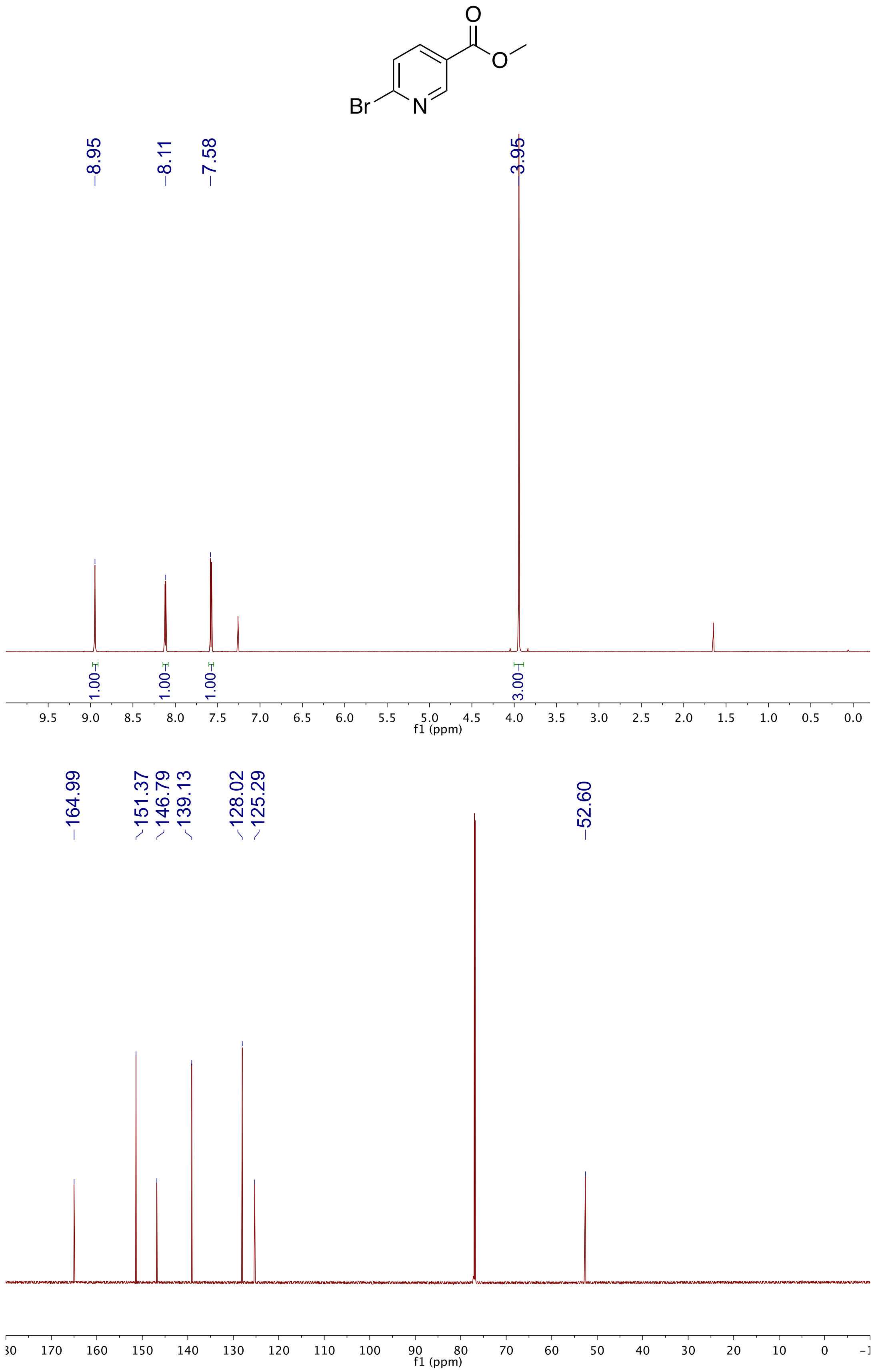

Figure. S17. Chemical structure and NMR spectra of Methyl 5-chloropyrazine-2-carboxylate, 11h

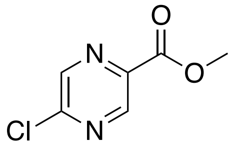

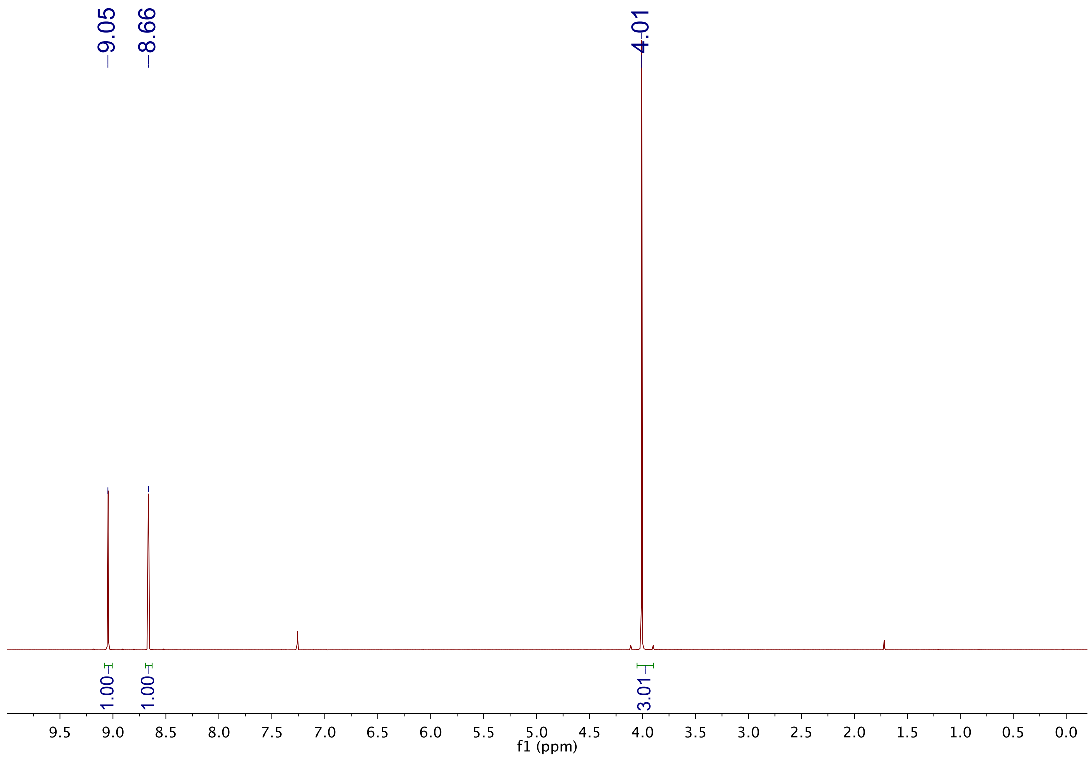

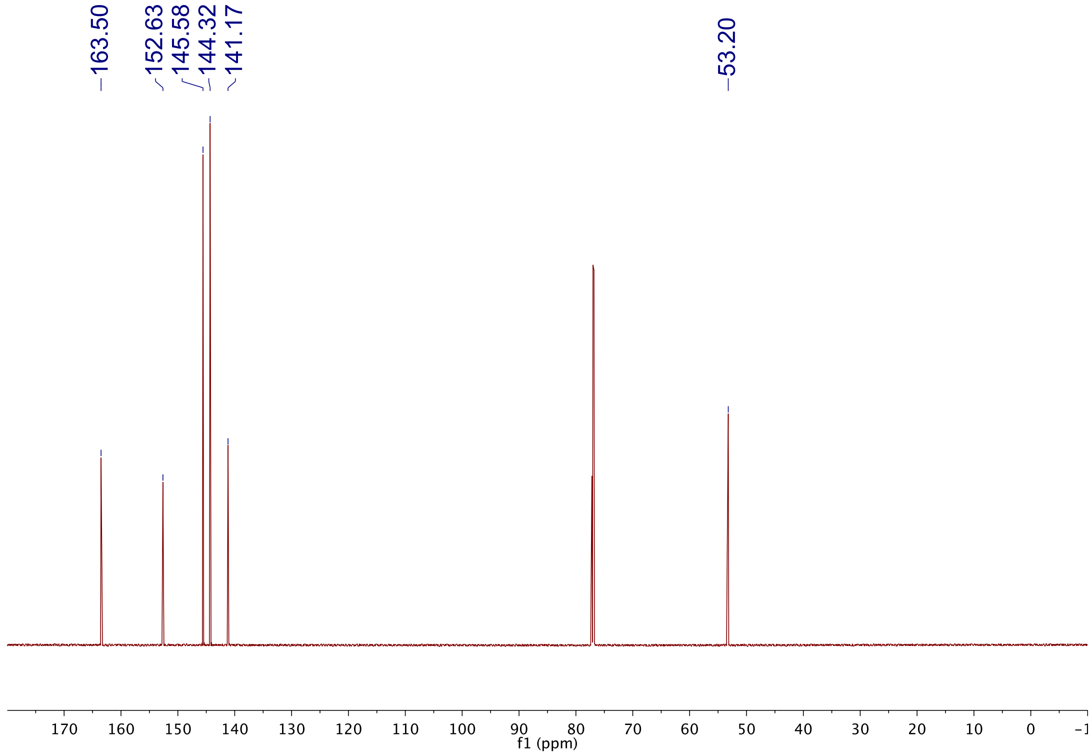

Figure. S18. Chemical structure and NMR spectra of Methyl 4-ethynyl-3-fluorobenzoate, 12a

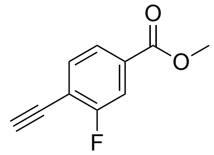

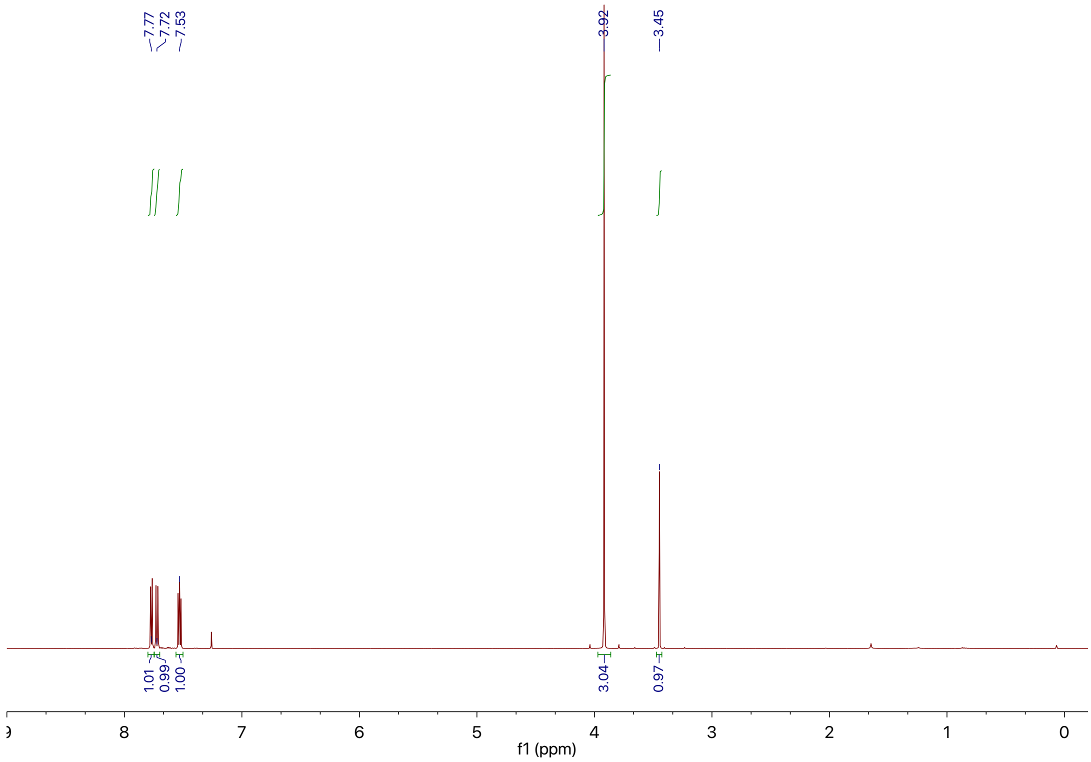

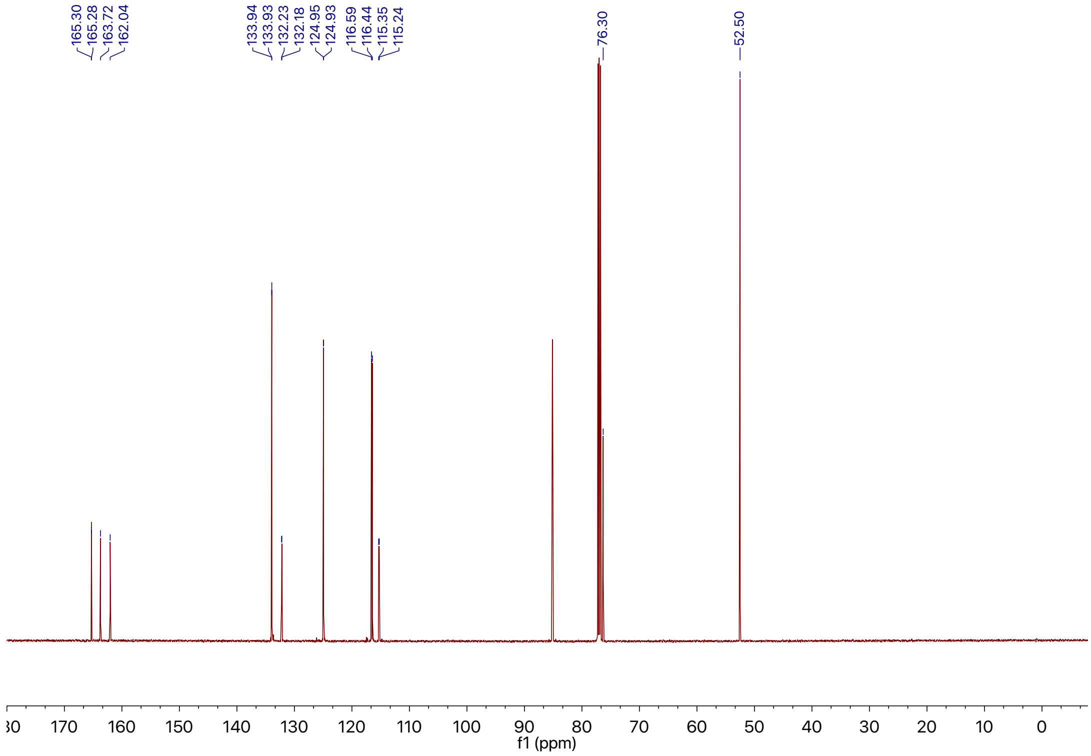

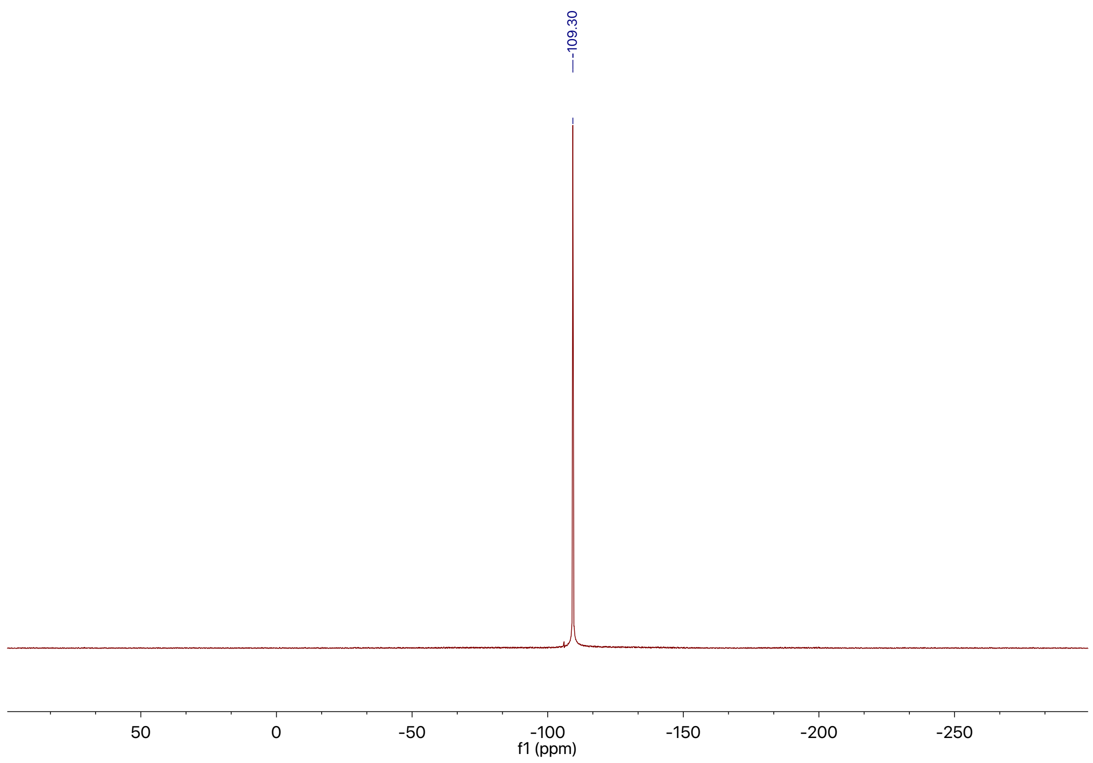

-109.30
50
0
-50
-100
-150
-200
-250
f1 (ppm)

Figure. S19. Chemical structure and NMR spectra of Methyl 4-ethynyl-2,6-difluorobenzoate, 12b

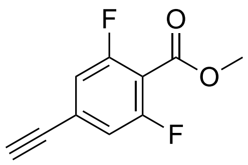

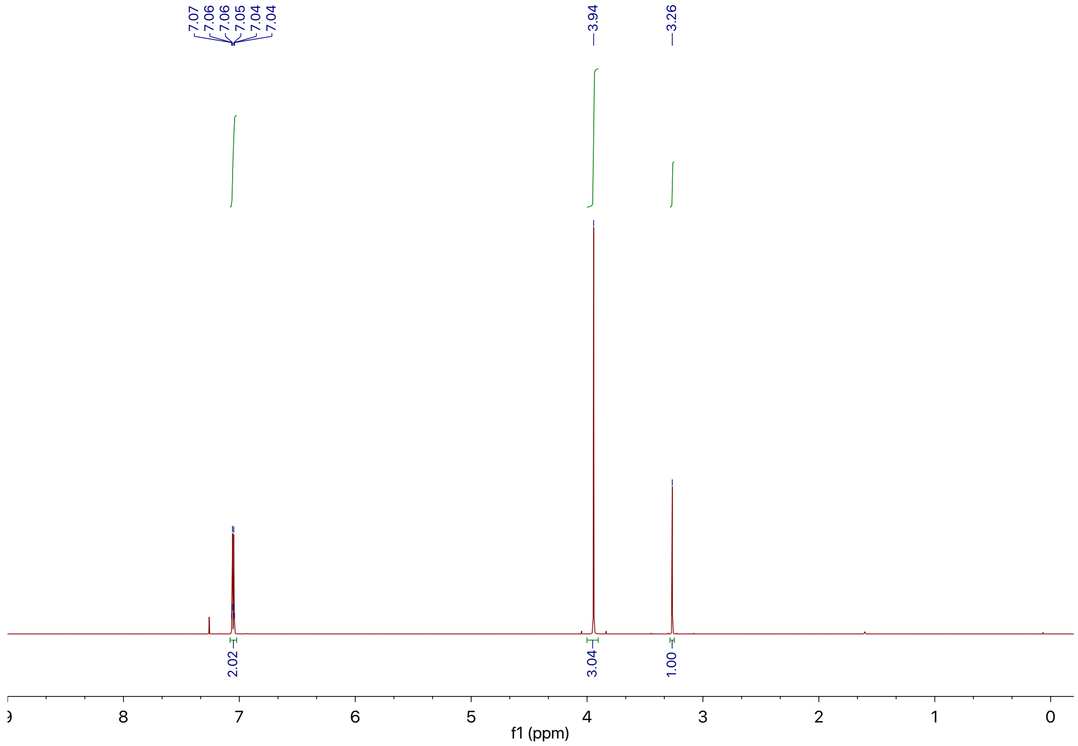

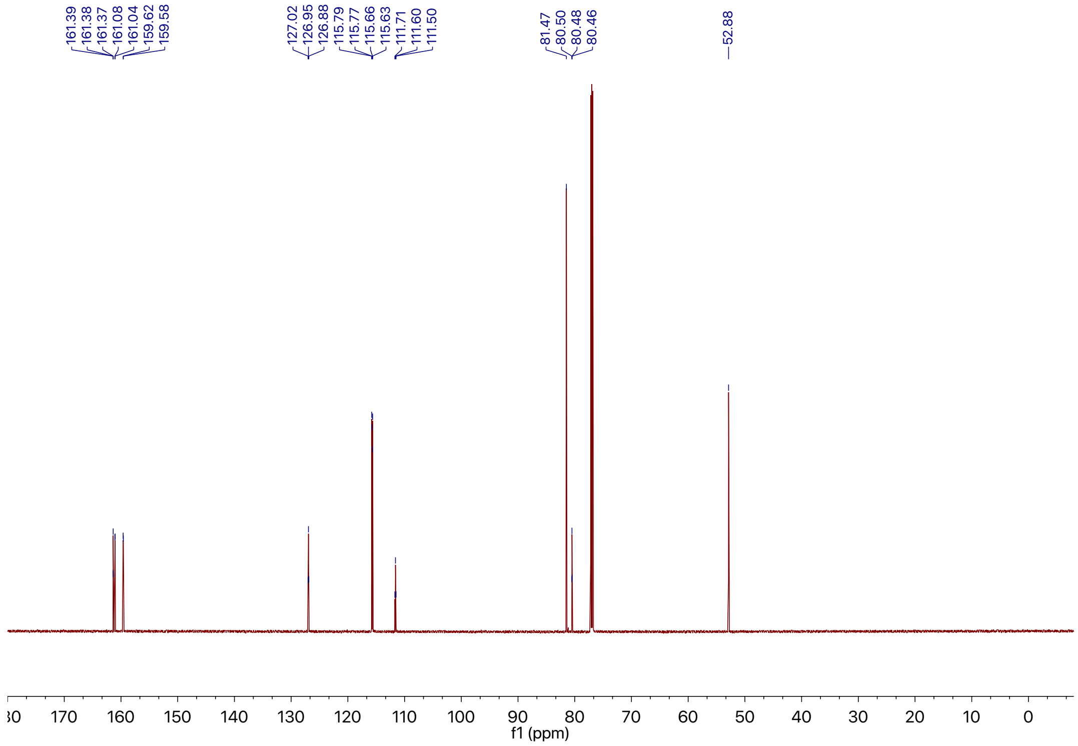

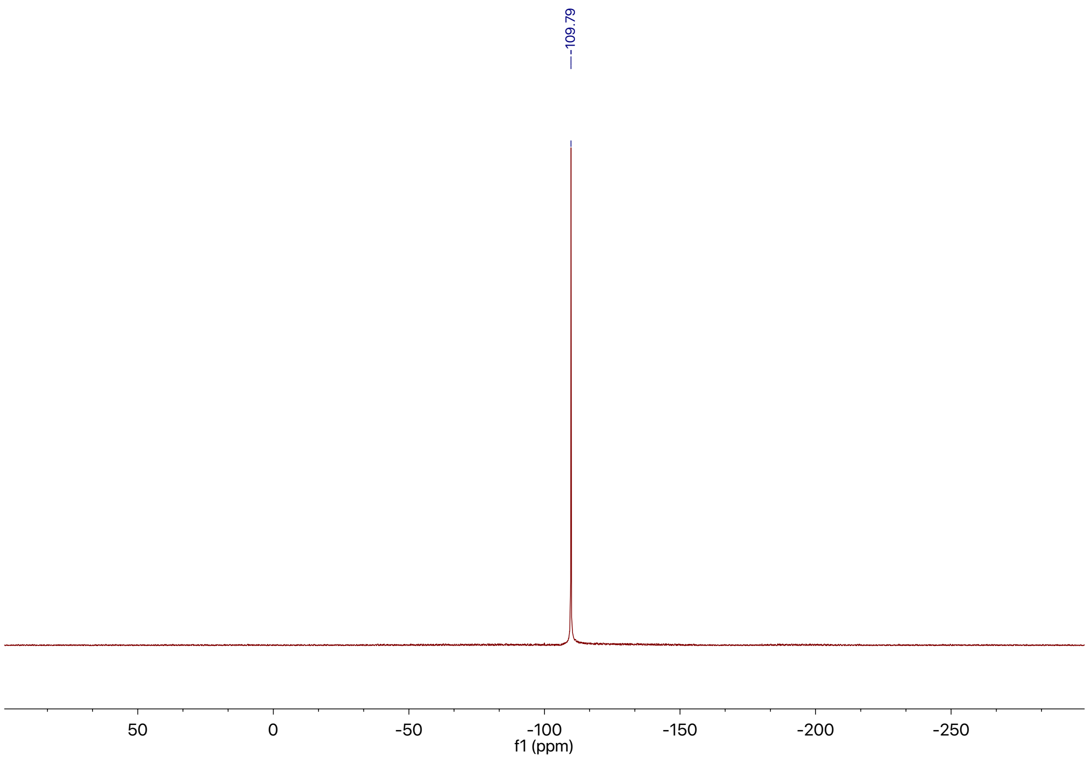
-109.79
50
0
-50
-100
-150
-200
-250
f1 (ppm)

Figure. S20. Chemical structure and NMR spectra of Methyl 4-ethynyl-3-chlorobenzoate, 12c

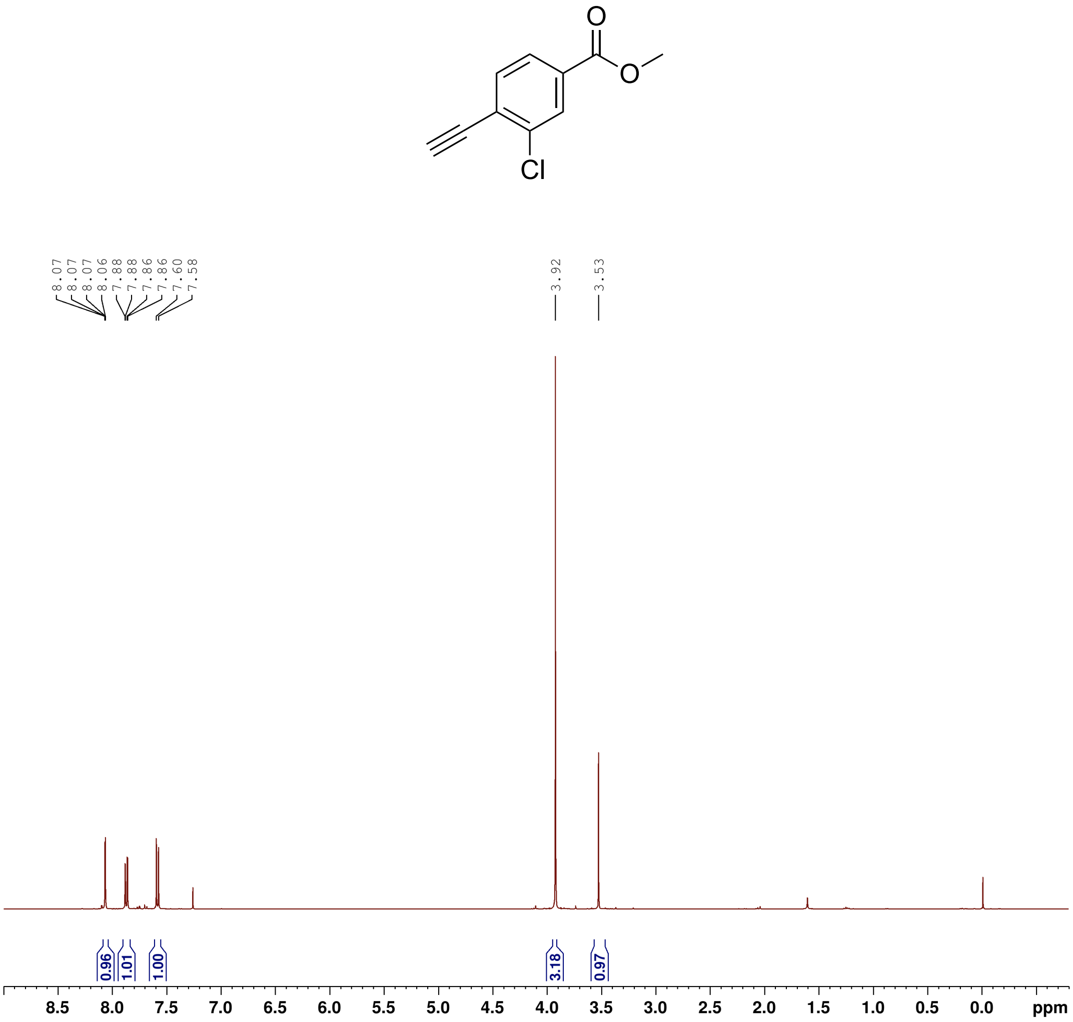

Figure. S21. Chemical structure and NMR spectra of 2-Fluoro-4-[2-(5,5,8,8-tetramethyl-5,6,7,8-tetrahydronaphthalen-2-yl)ethynyl]benzoic acid, 13

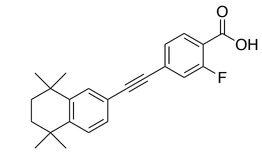

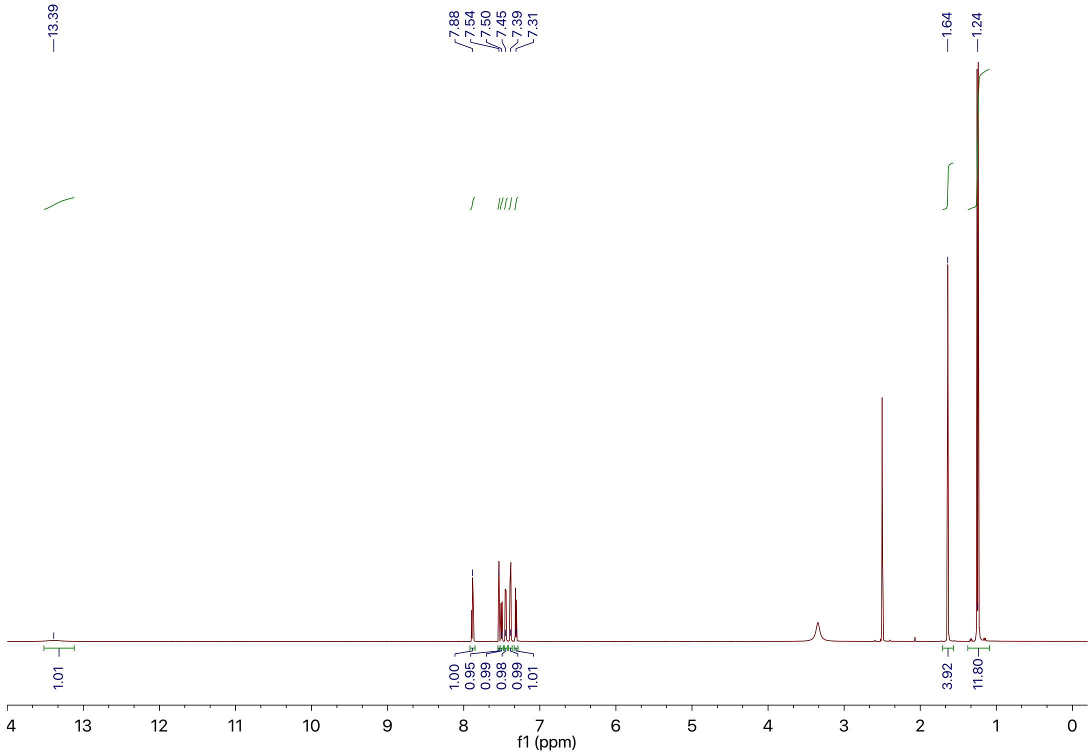

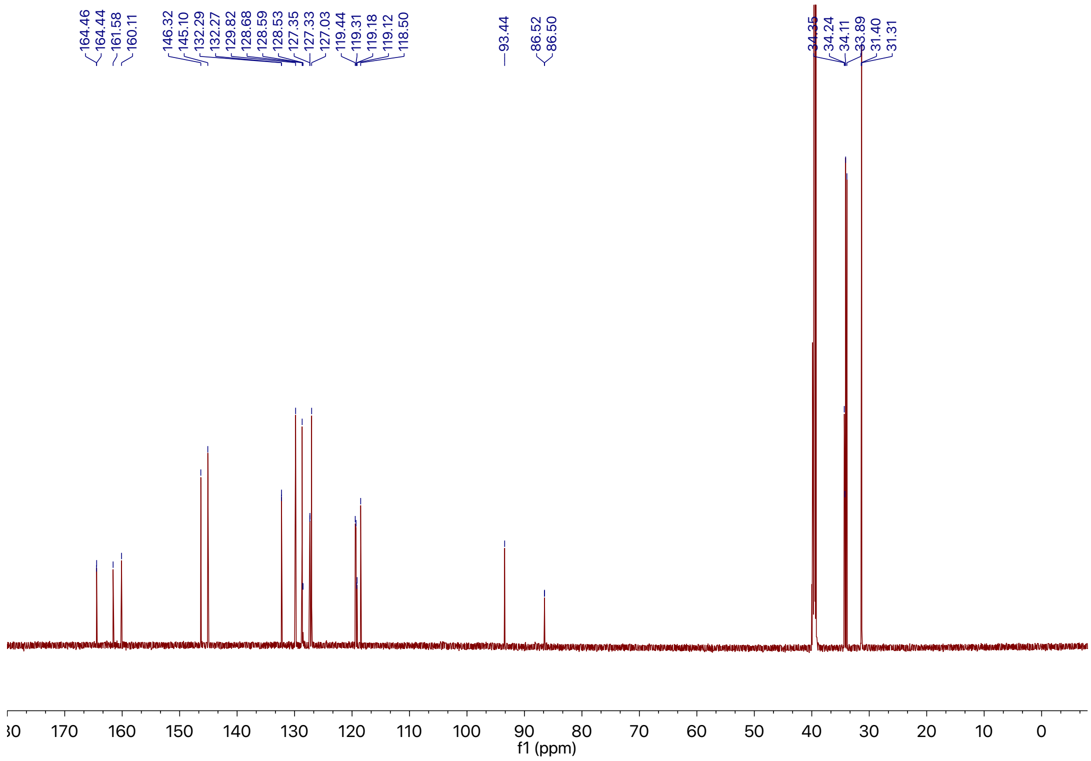

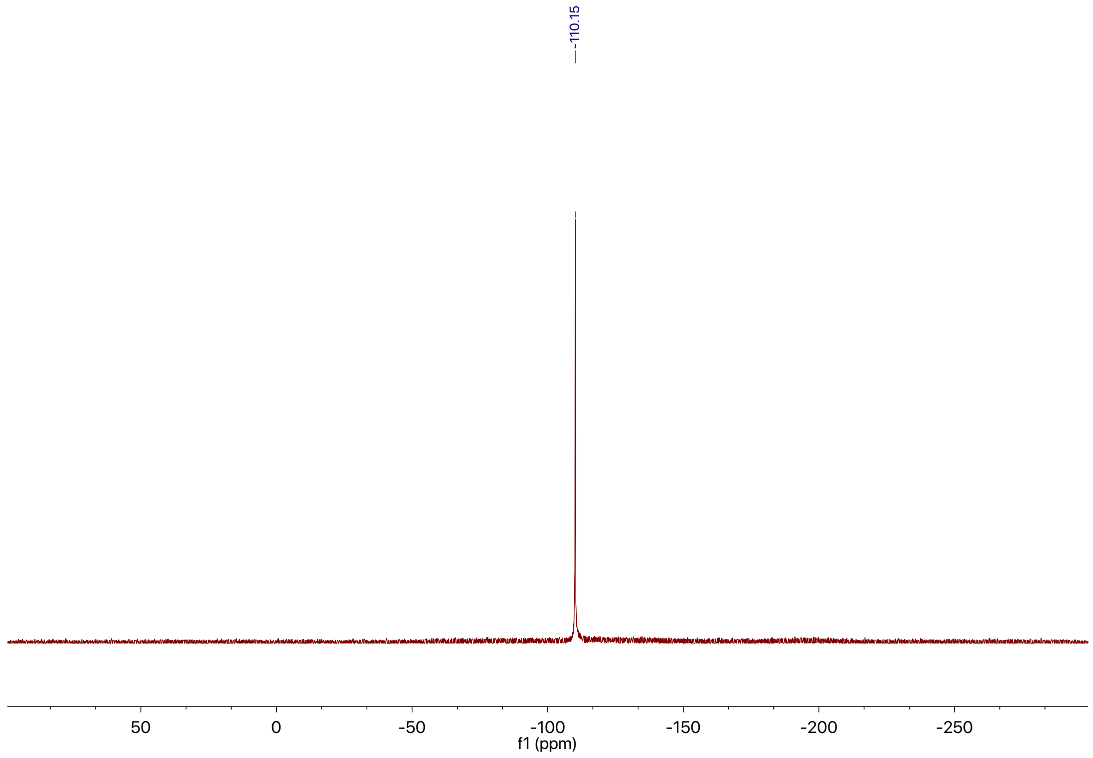
-110.15
50
0
-50
-100
-150
-200
-250
f1 (ppm)

Figure. S22. Chemical structure and NMR spectra of 3-Fluoro-4-[2-(5,5,8,8-tetramethyl-5,6,7,8-tetrahydronaphthalen-2-yl)ethynyl]benzoic acid, 14

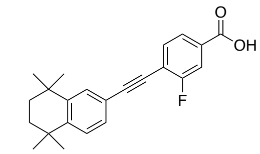

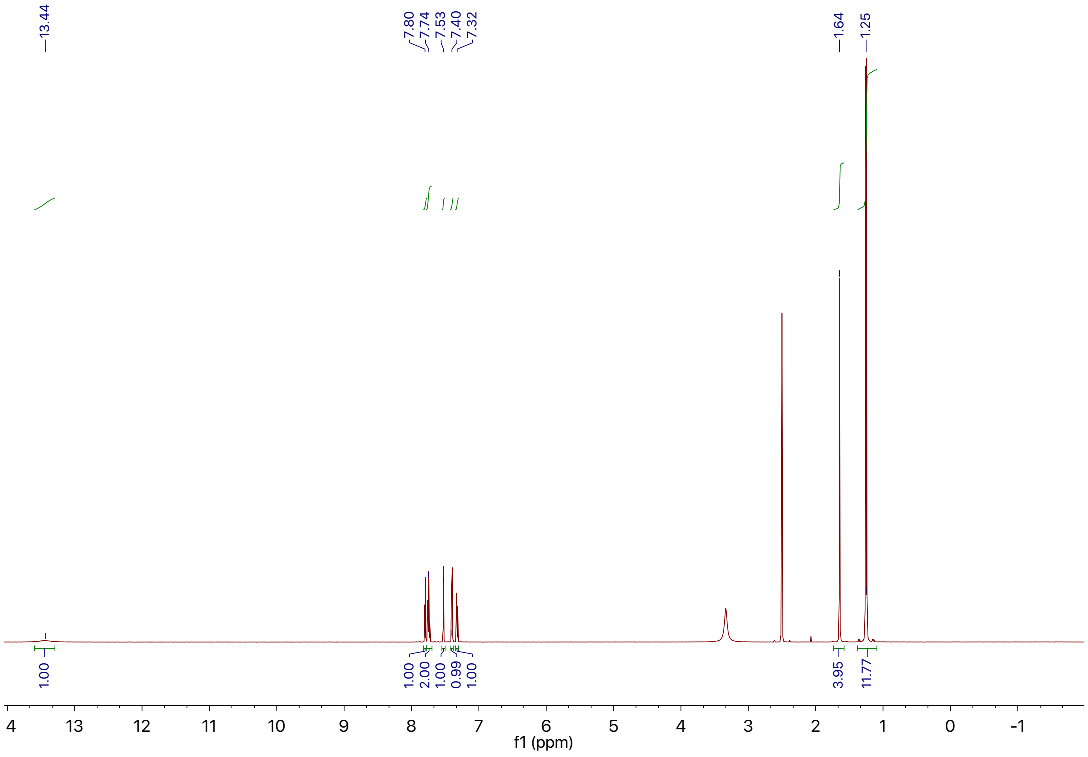

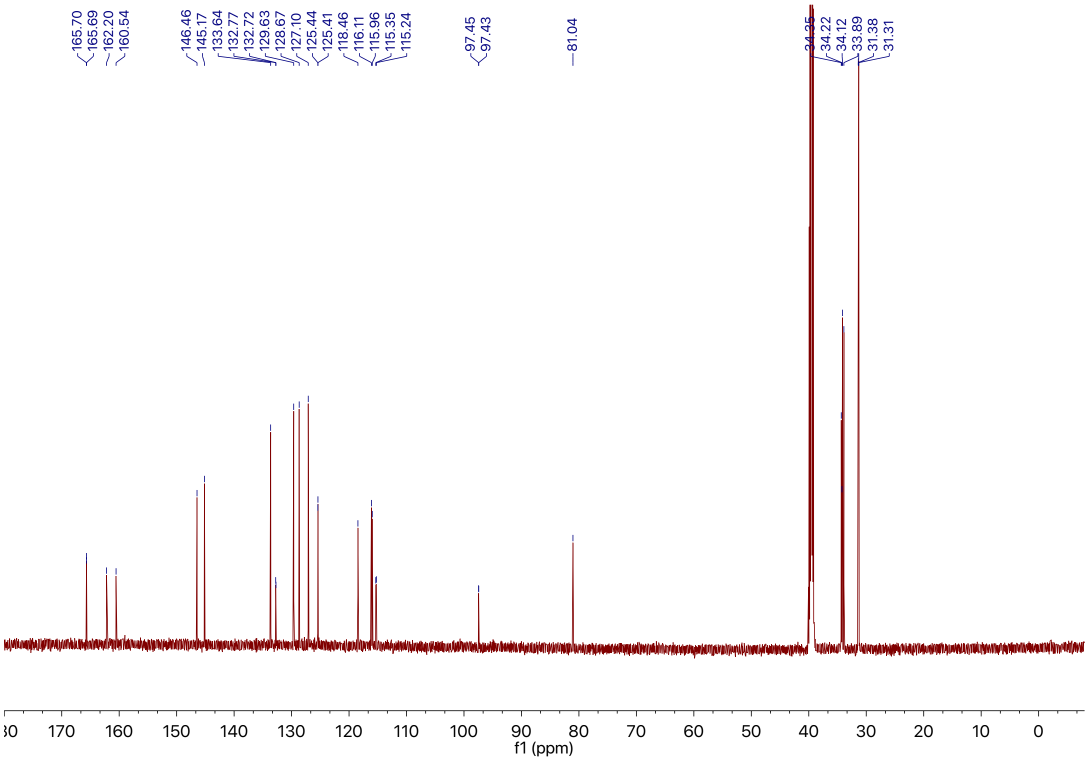

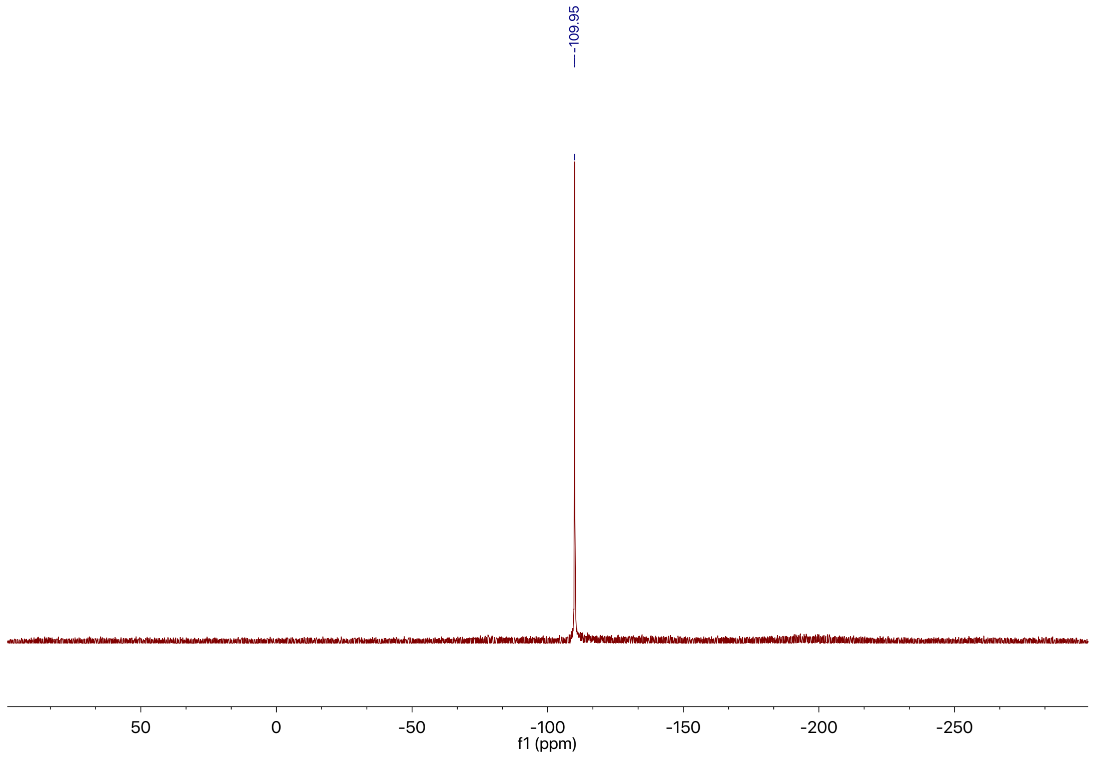
-109.95
50
0
-50
-100
-150
-200
-250
f1 (ppm)

Figure. S23. Chemical structure and NMR spectra of 2,6-Difluoro-4-[2-(5,5,8,8-tetramethyl-5,6,7,8-tetrahydronaphthalen-2-yl)ethynyl]benzoic acid, 15

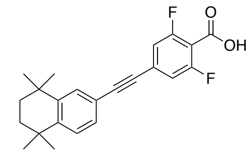

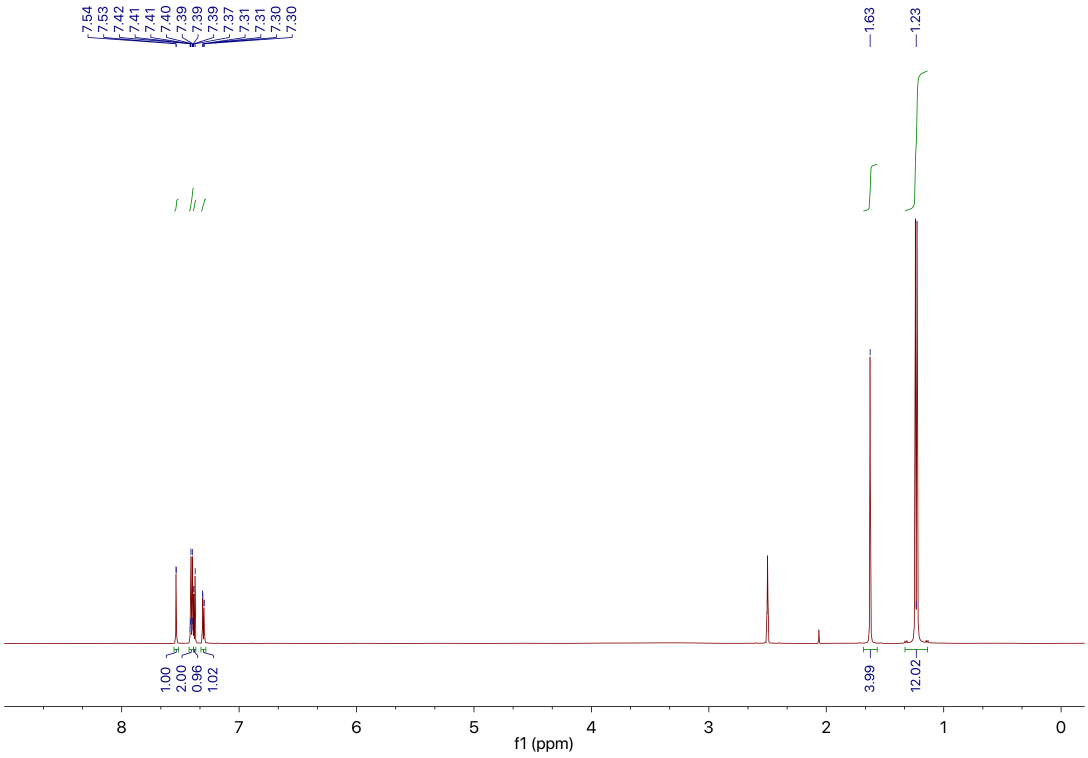

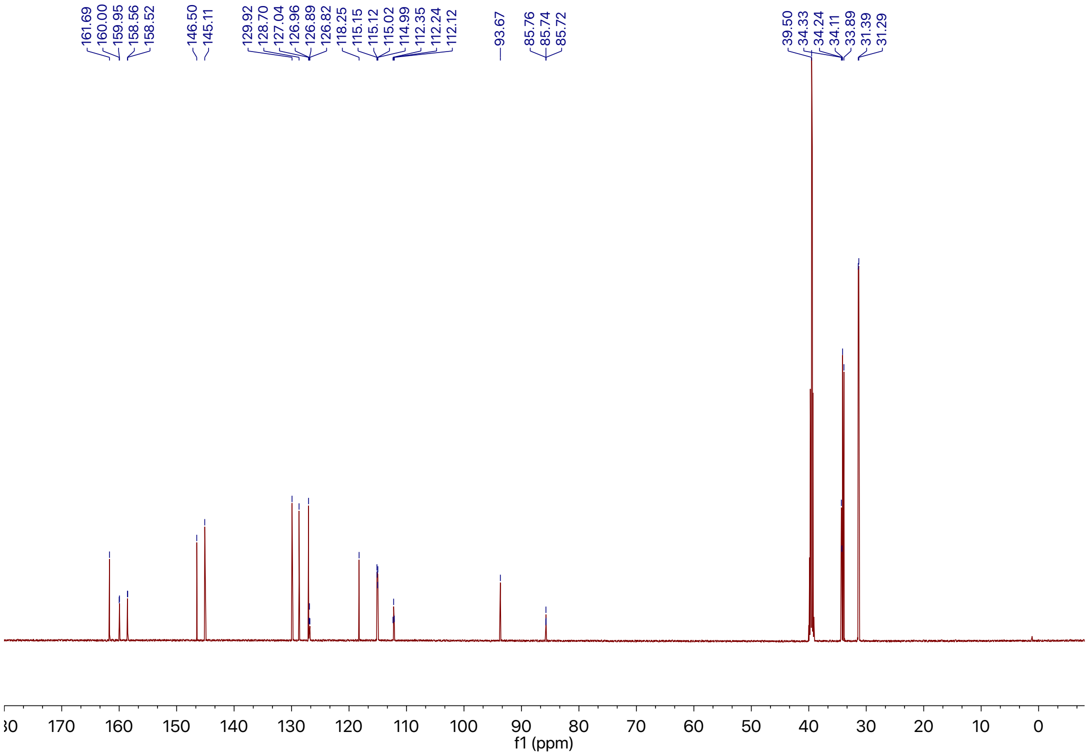

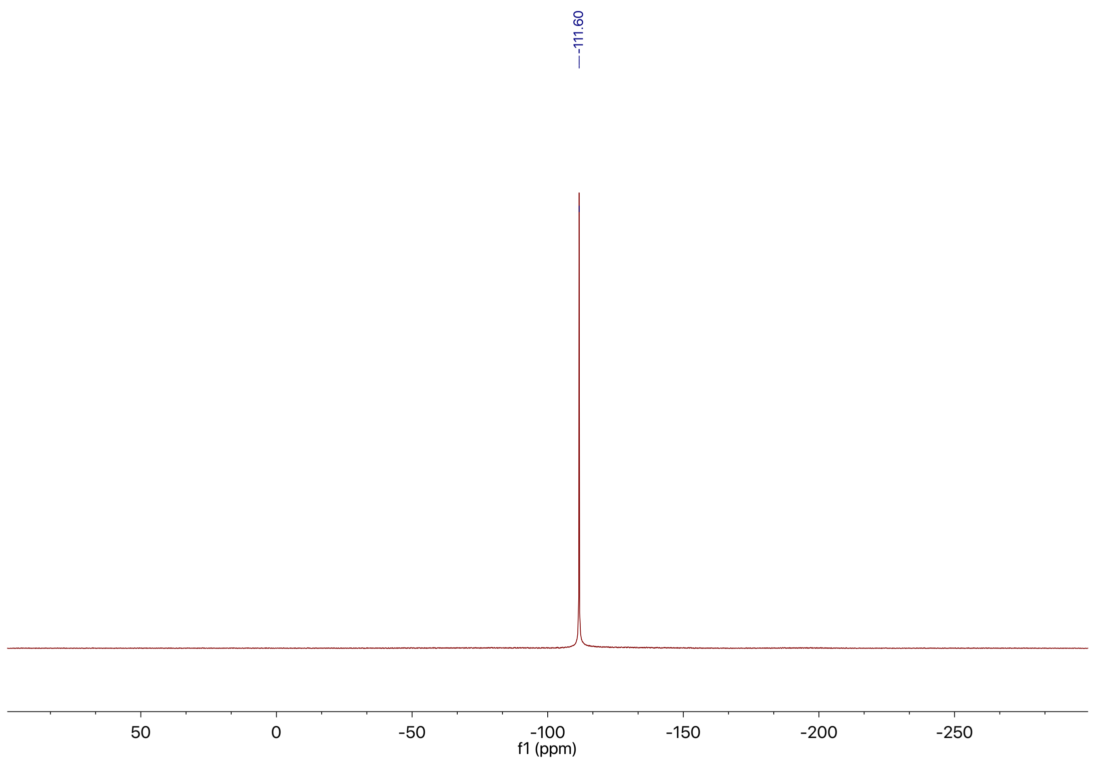
-111.60
50
0
-50
-100
-150
-200
-250
f1 (ppm)

Figure. S24. Chemical structure and NMR spectra of 3-Chloro-4-[2-(5,5,8,8-tetramethyl-5,6,7,8-tetrahydronaphthalen-2-yl)ethynyl]benzoic acid, 16

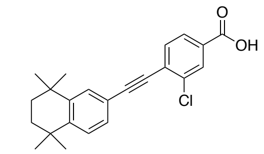

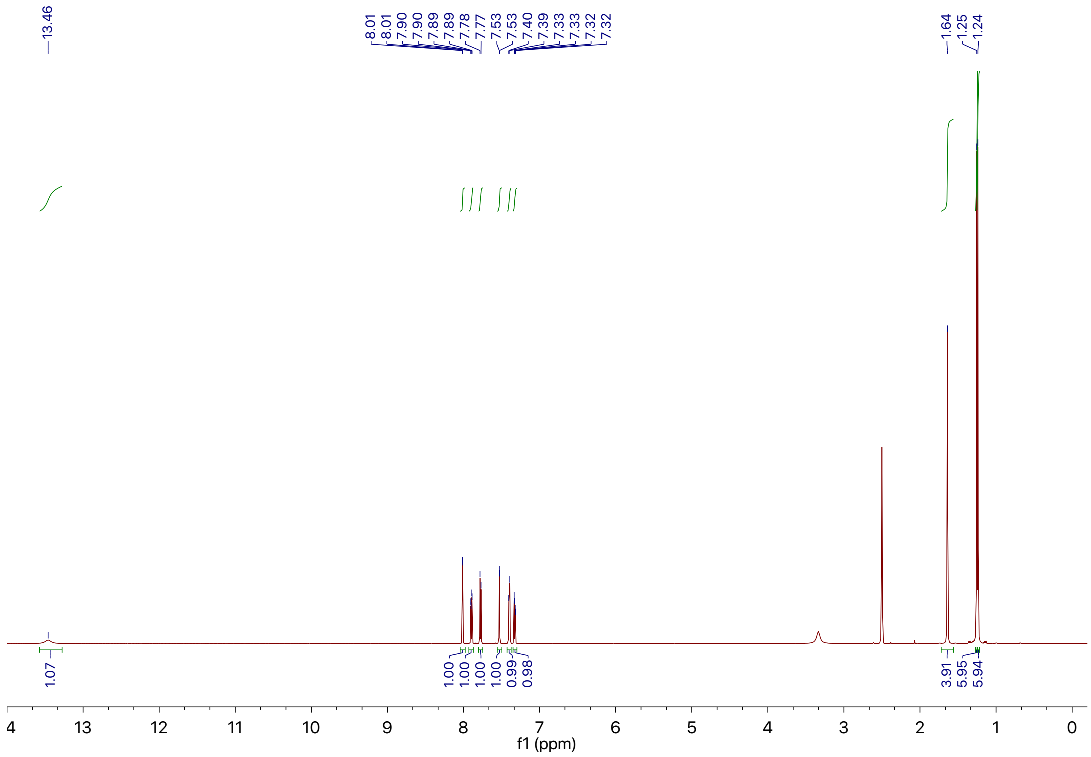

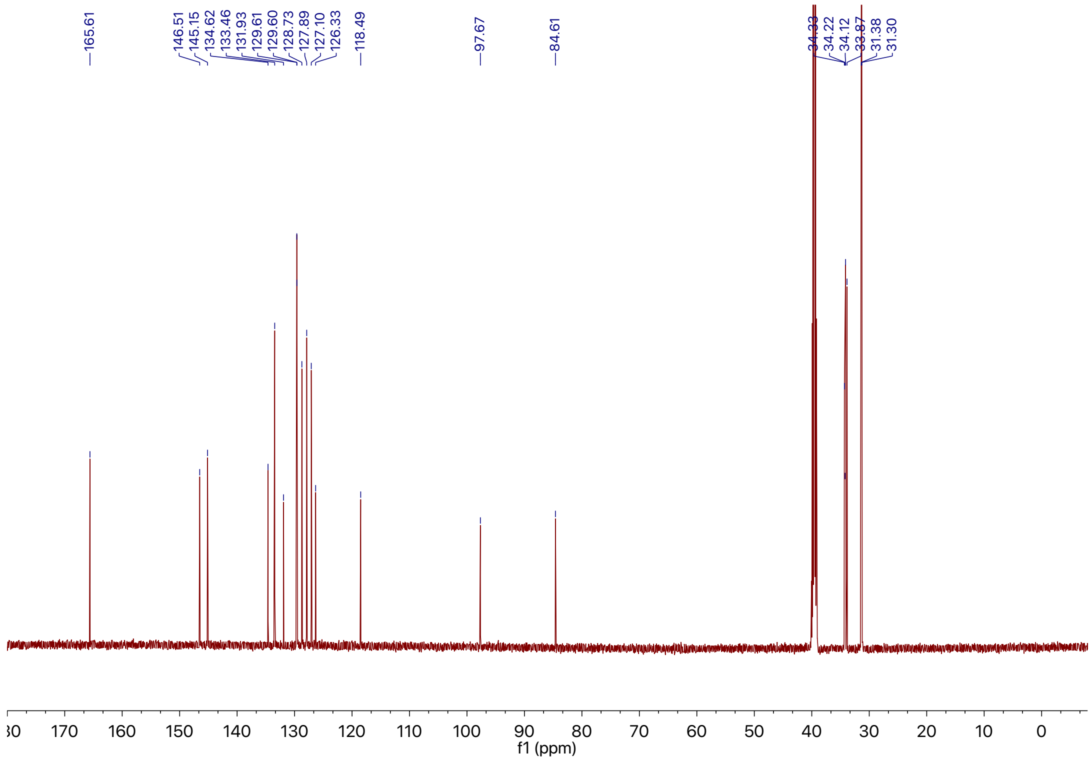

Figure. S25. Chemical structure and NMR spectra of 2-Fluoro-4-[2-(3,5,5,8,8-pentamethyl-5,6,7,8-tetrahydronaphthalen-2-yl)ethynyl]benzoic acid, 17

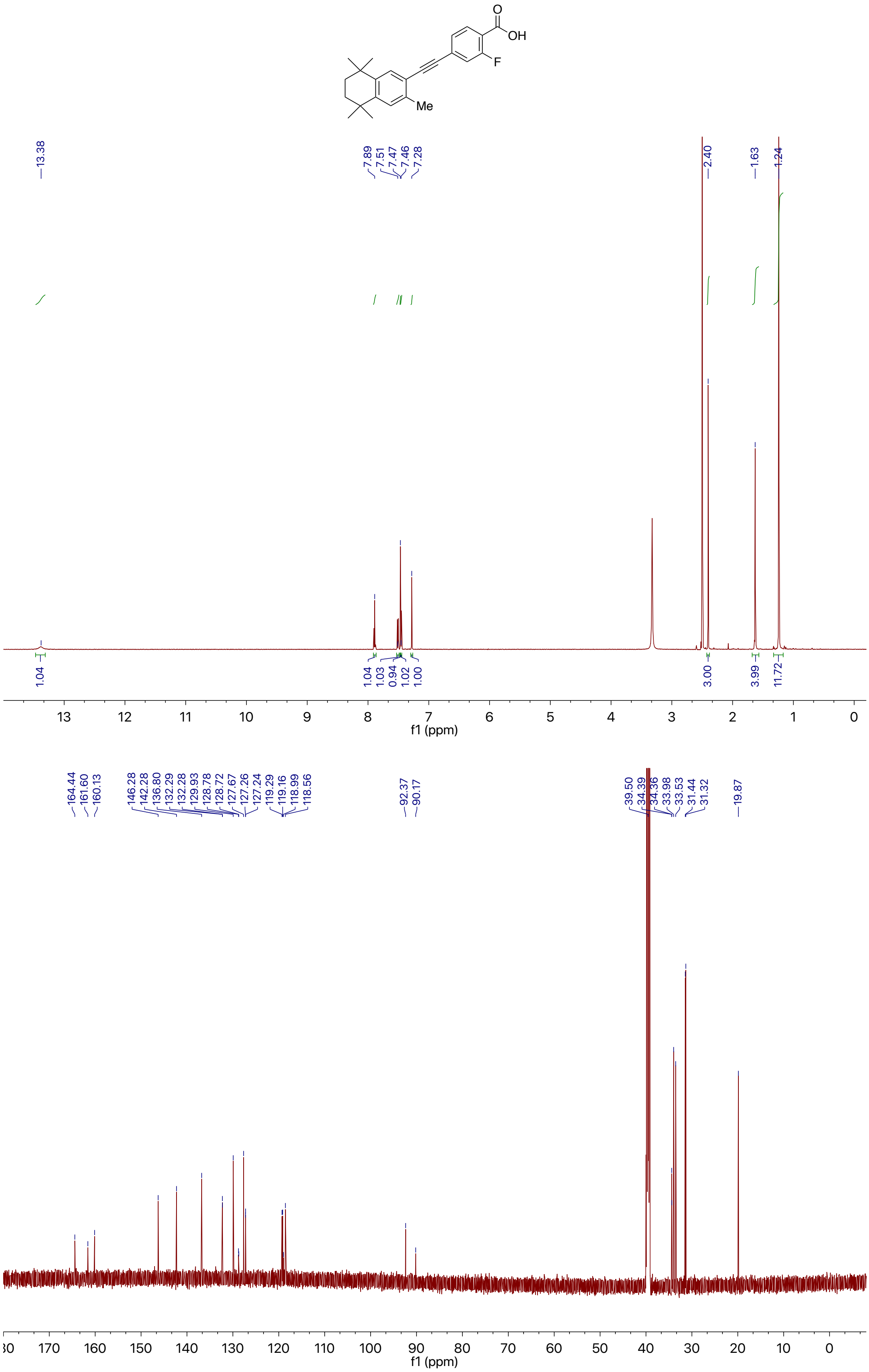

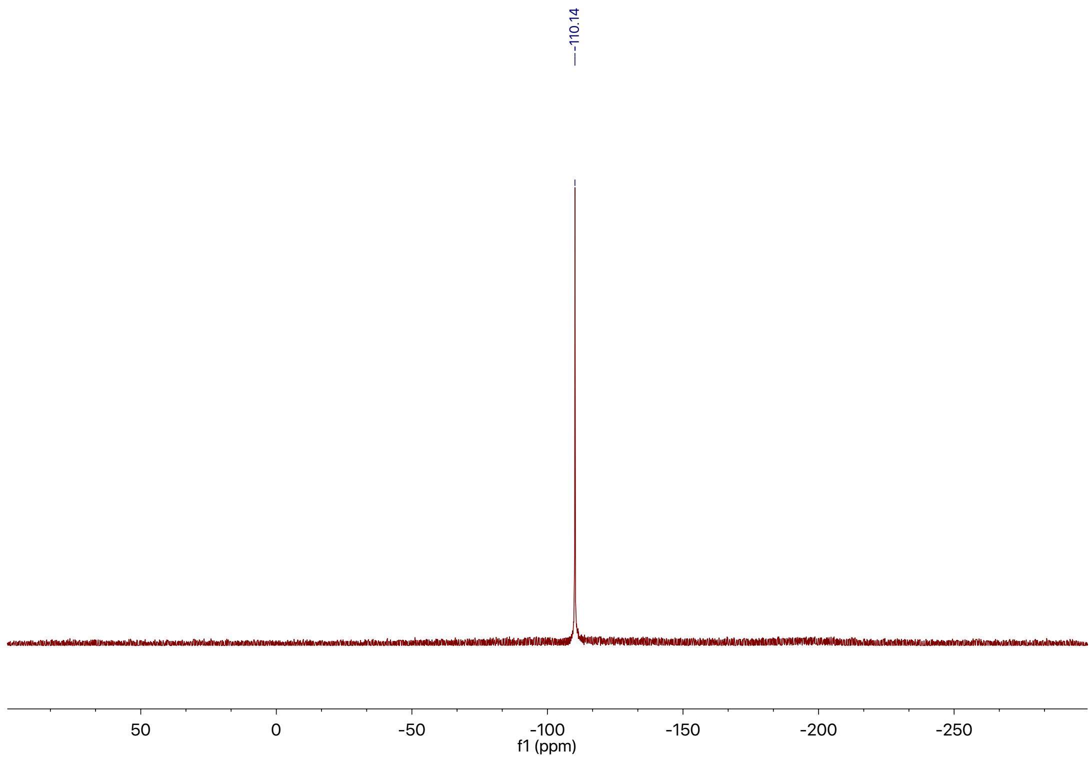
-110.14
50
0
-50
-100
-150
-200
-250
f1 (ppm)

Figure. S26. Chemical structure and NMR spectra of 3-Fluoro-4-[2-(3,5,5,8,8-pentamethyl-5,6,7,8-tetrahydronaphthalen-2-yl)ethynyl]benzoic acid, 18

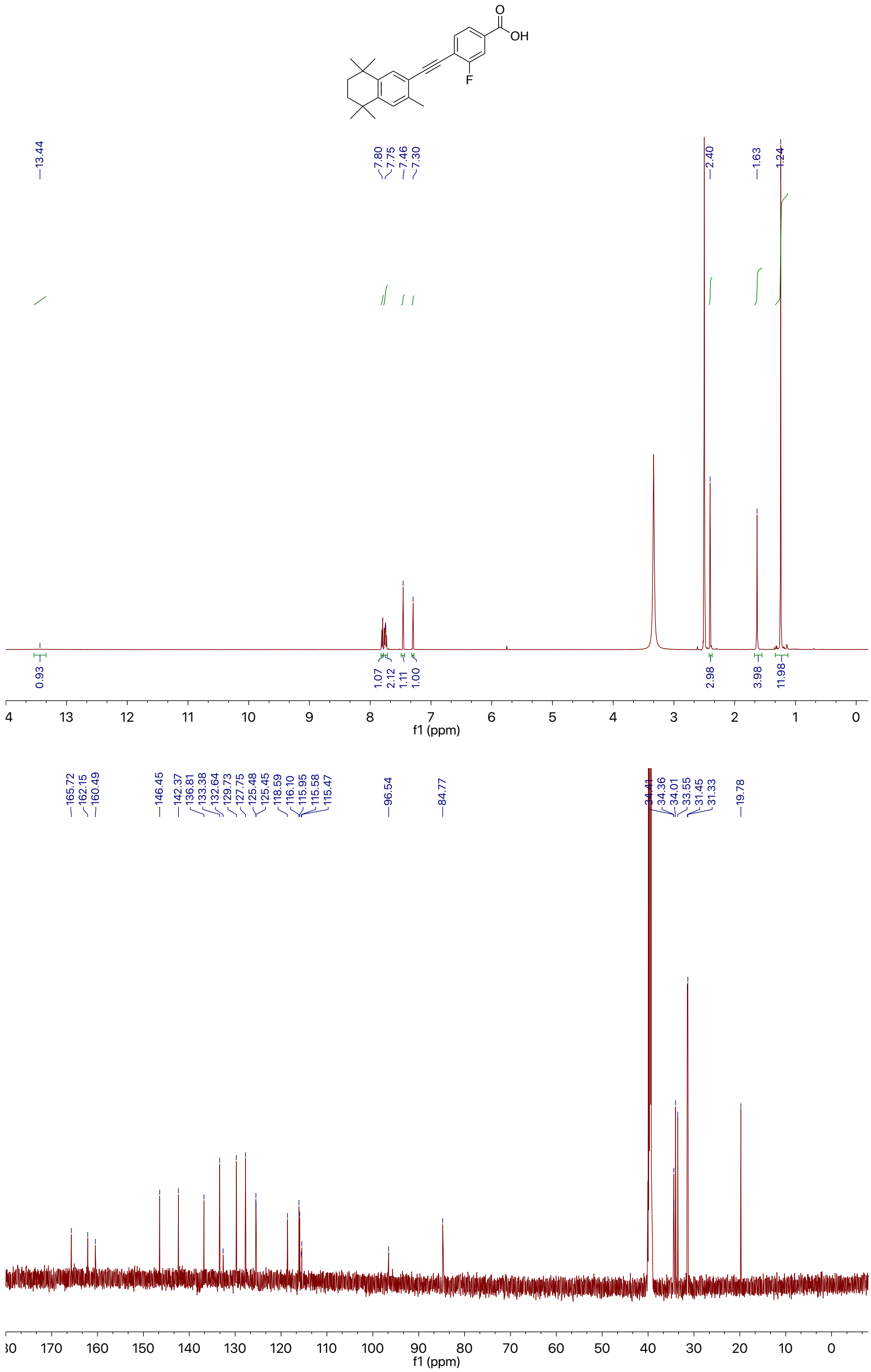

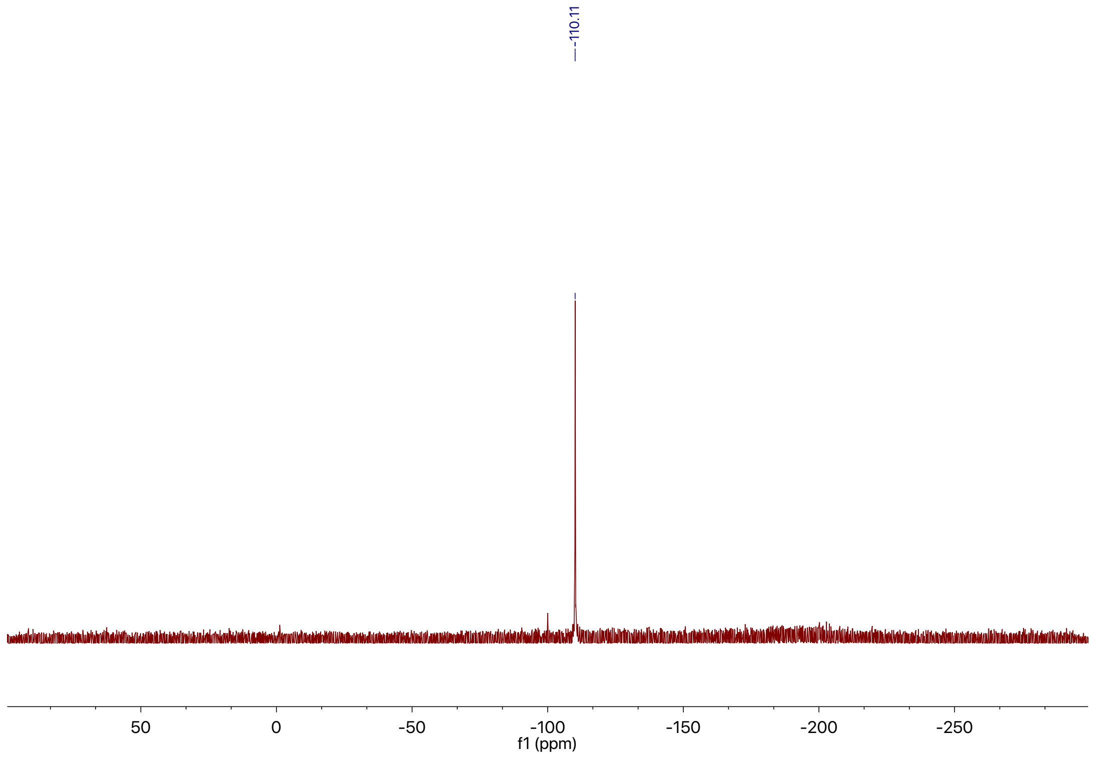

-110.11
50
0
-50
-100
-150
-200
-250
f1 (ppm)

Figure. S27. Chemical structure and NMR spectra of 2,6-Difluoro-4-[2-(3,5,5,8,8-pentamethyl-5,6,7,8-tetrahydronaphthalen-2-yl)ethynyl]benzoic acid, 19

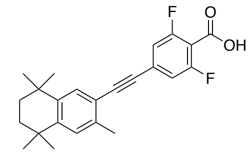

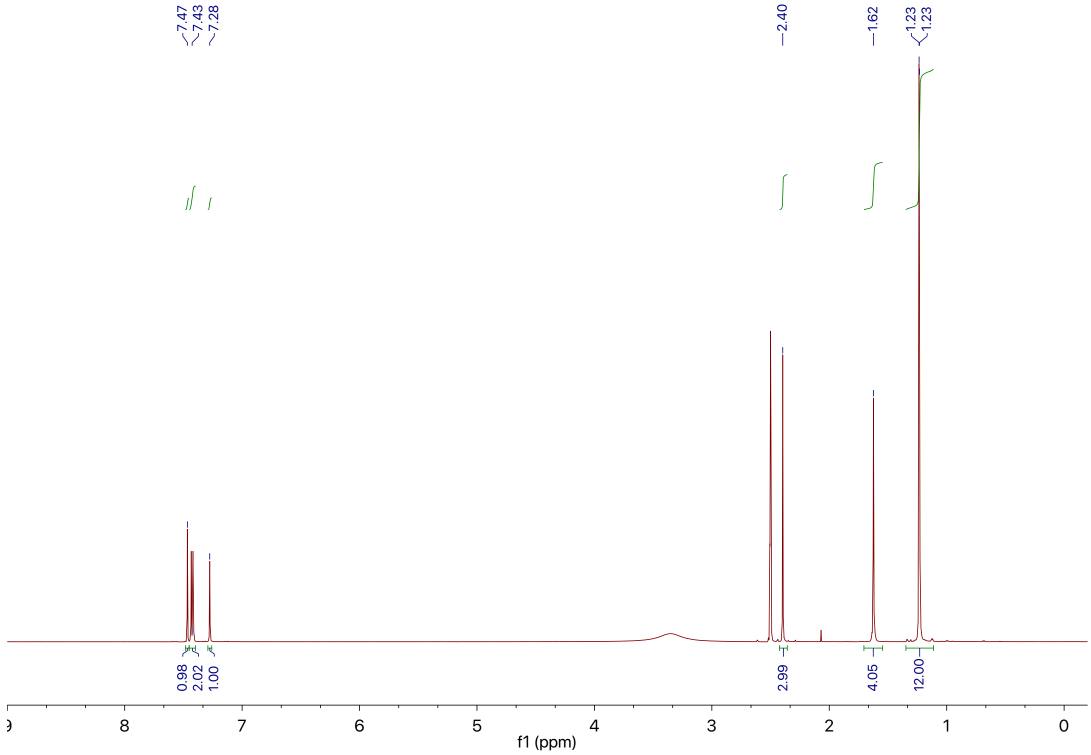

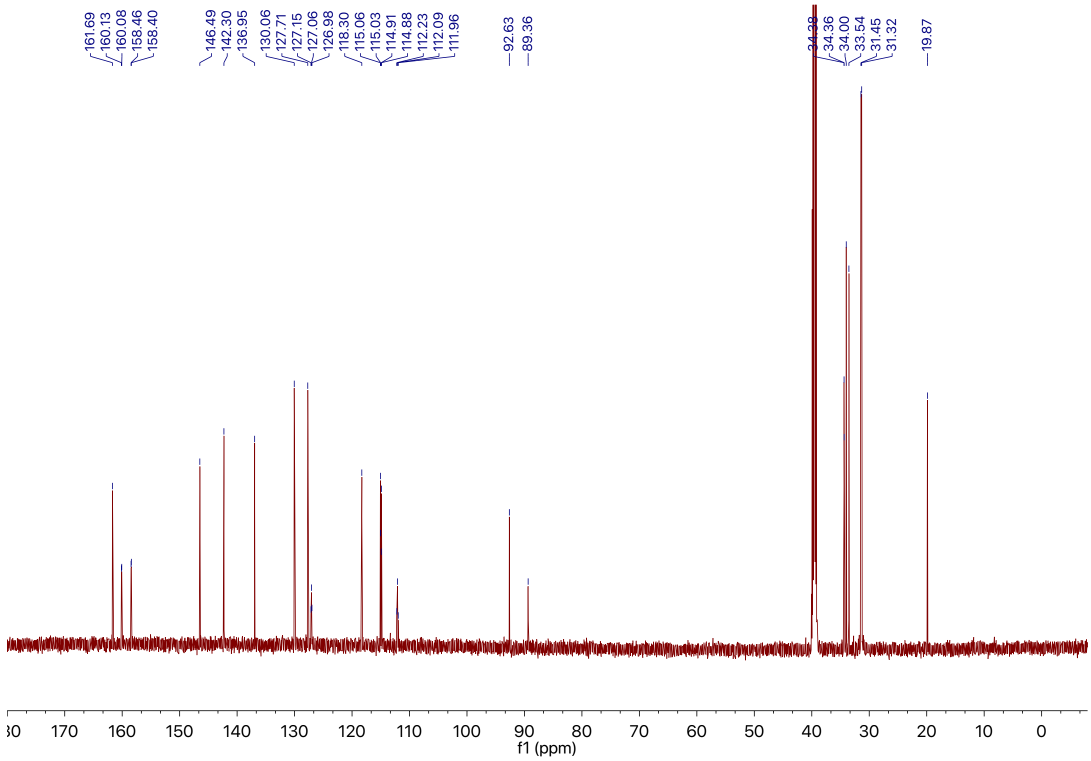

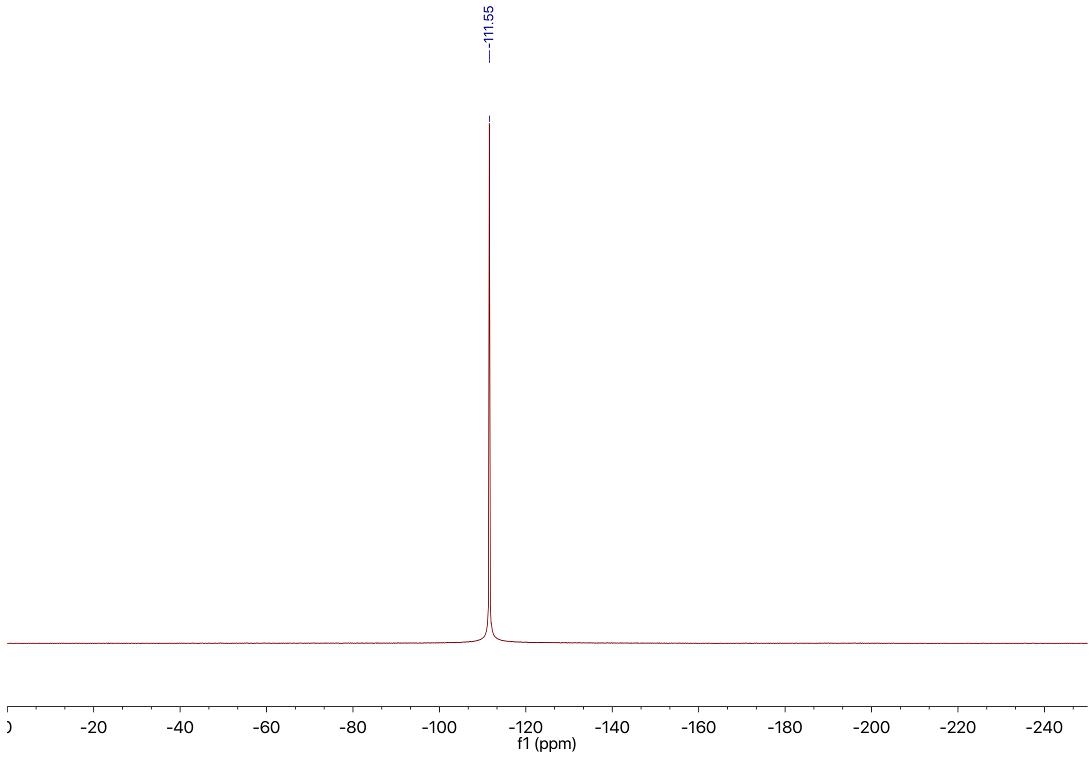
-111.55
0
-20
-40
-60
-80
-100
-120
-140
-160
-180
-200
-220
-240
f1 (ppm)

Figure. S28. Chemical structure and NMR spectra of 2-Fluoro-4-[2-(3-methoxy-5,5,8,8-tetramethyl-5,6,7,8-tetrahydronaphthalen-2-yl)ethynyl]benzoic acid, 20

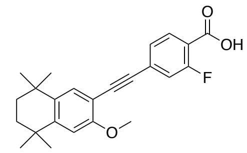

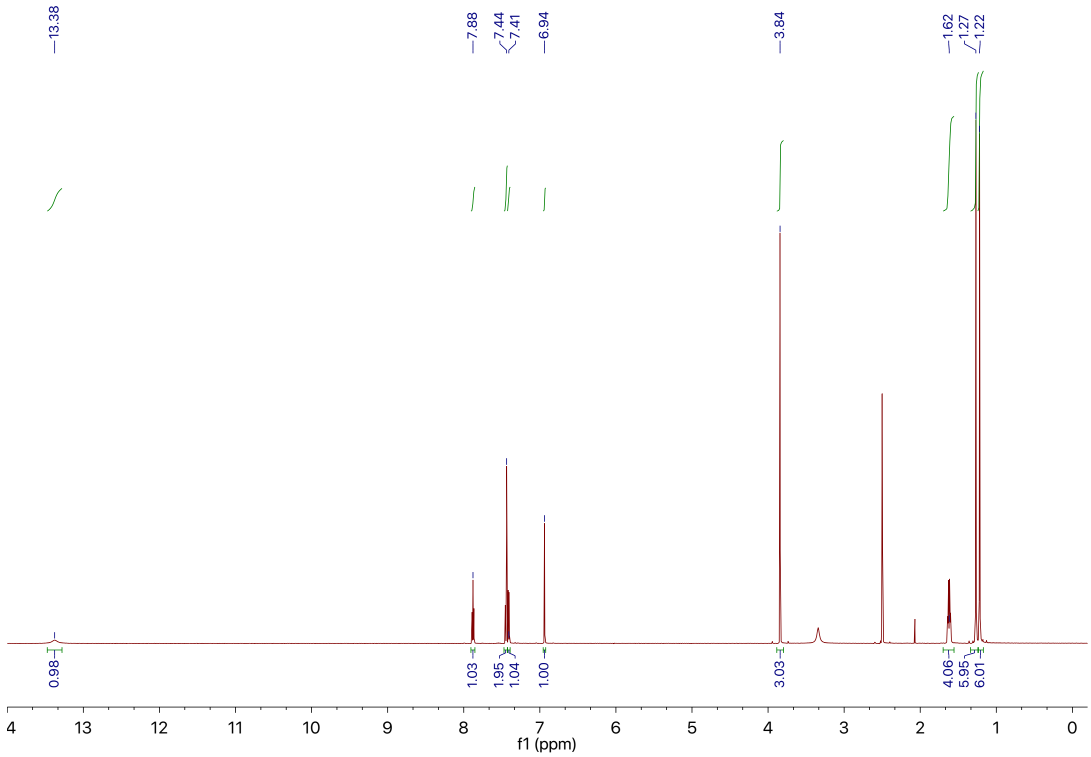

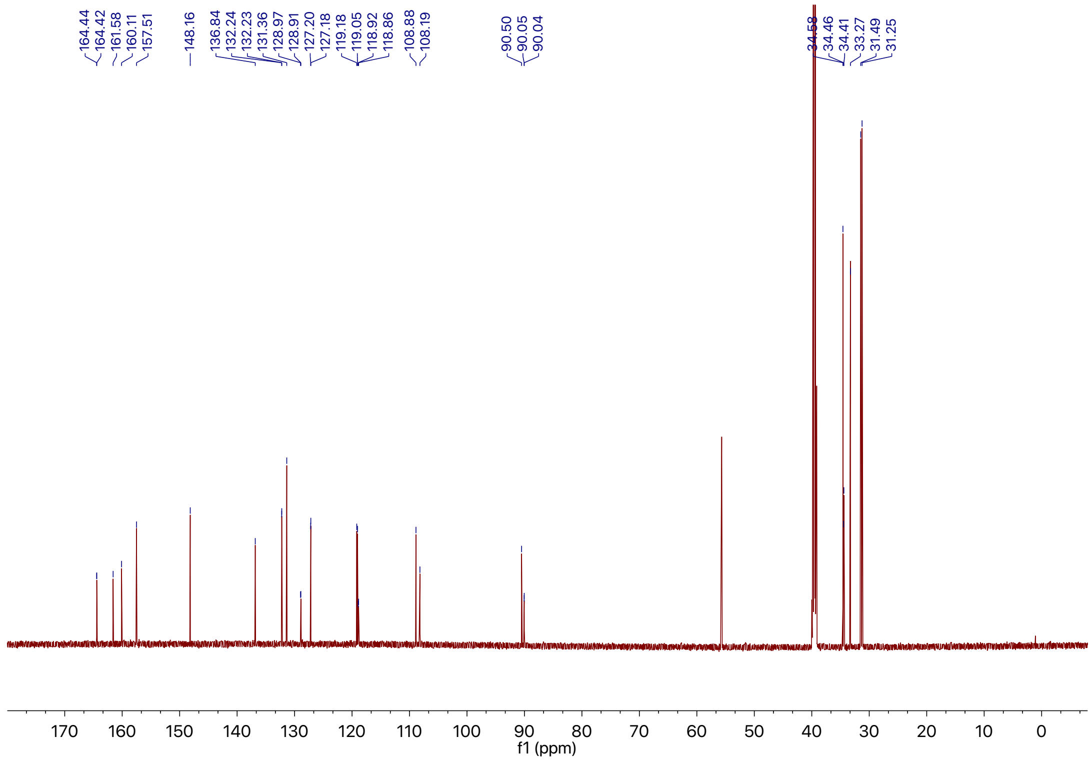

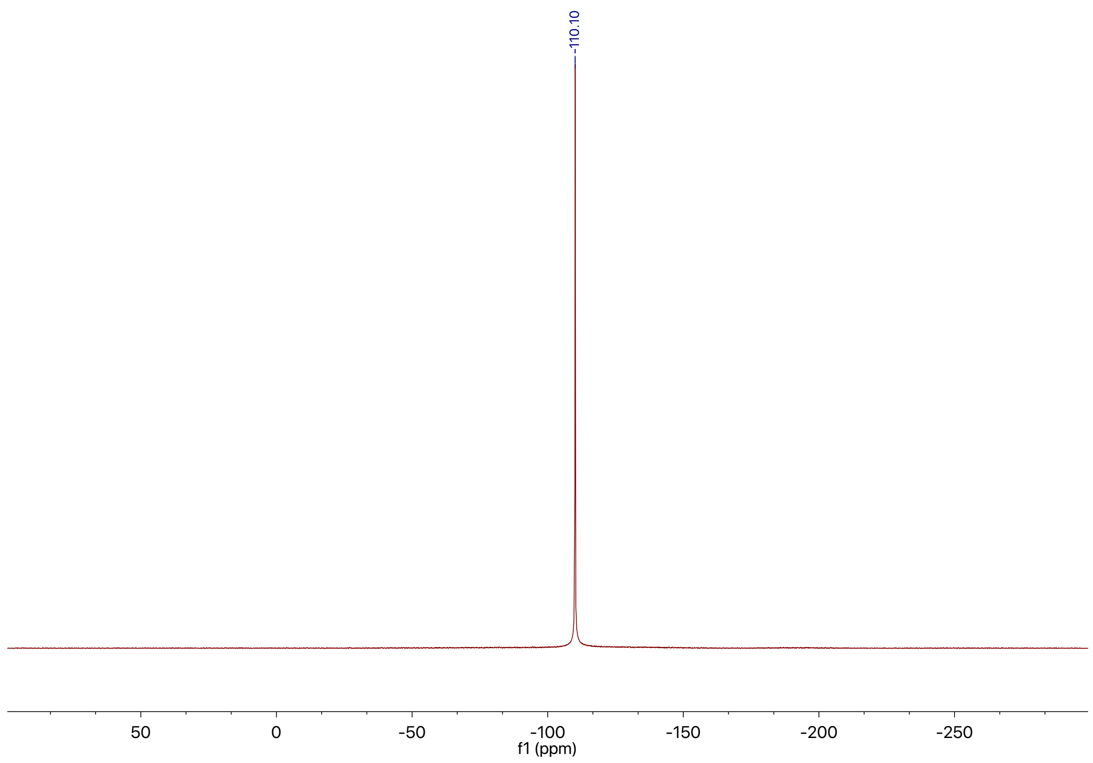

-110.10
50
0
-50
-100
-150
-200
-250
f1 (ppm)

Figure. S29. Chemical structure and NMR spectra of 3-Fluoro-4-[2-(3-methoxy-5,5,8,8-tetramethyl-5,6,7,8-tetrahydronaphthalen-2-yl)ethynyl]benzoic acid, 21

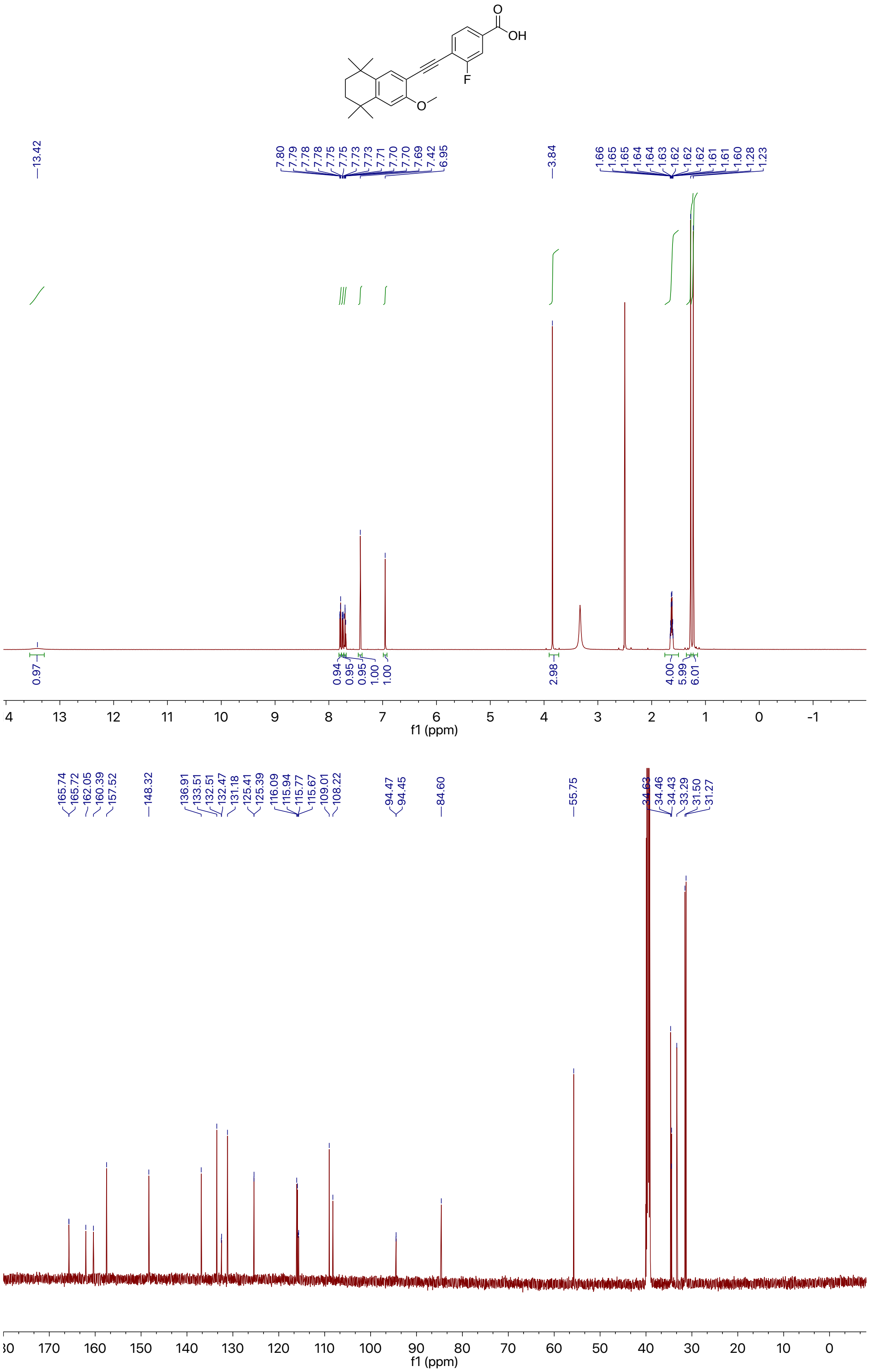

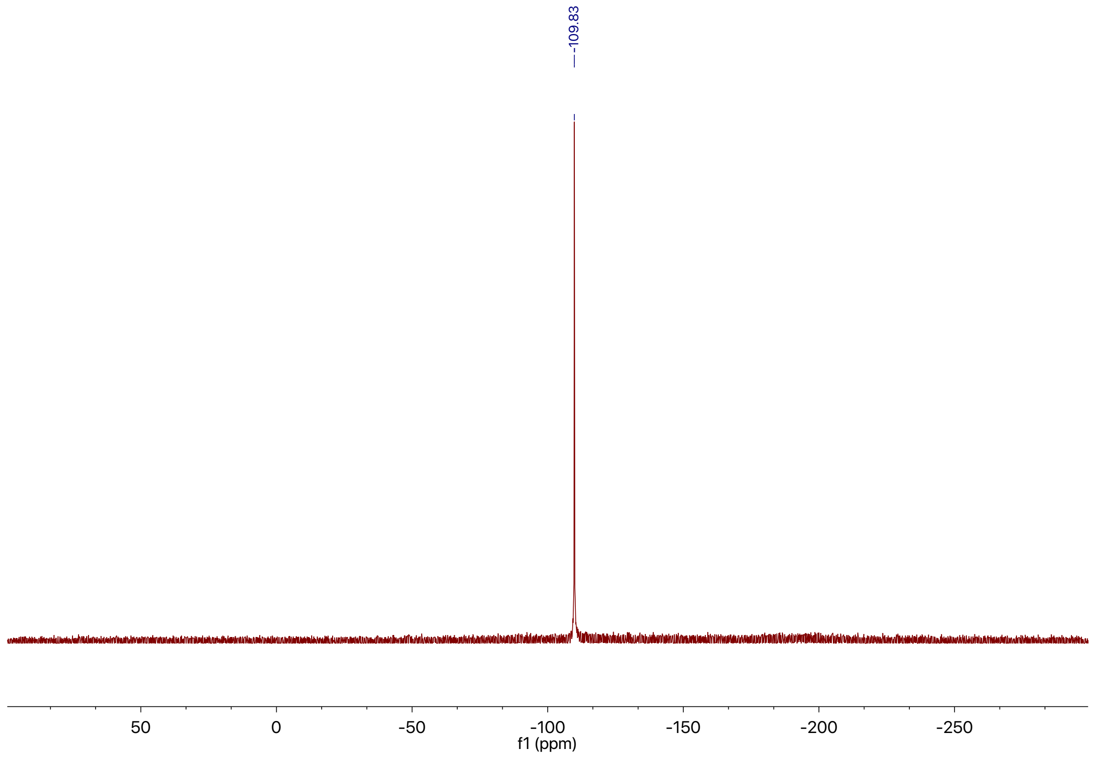
-109.83
50
0
-50
-100
-150
-200
-250
f1 (ppm)

Figure. S30. Chemical structure and NMR spectra of 2,6-Difluoro-4-[2-(3-methoxy-5,5,8,8-tetramethyl-5,6,7,8-tetrahydronaphthalen-2-yl)ethynyl]benzoic acid, 22

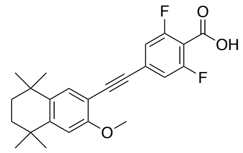

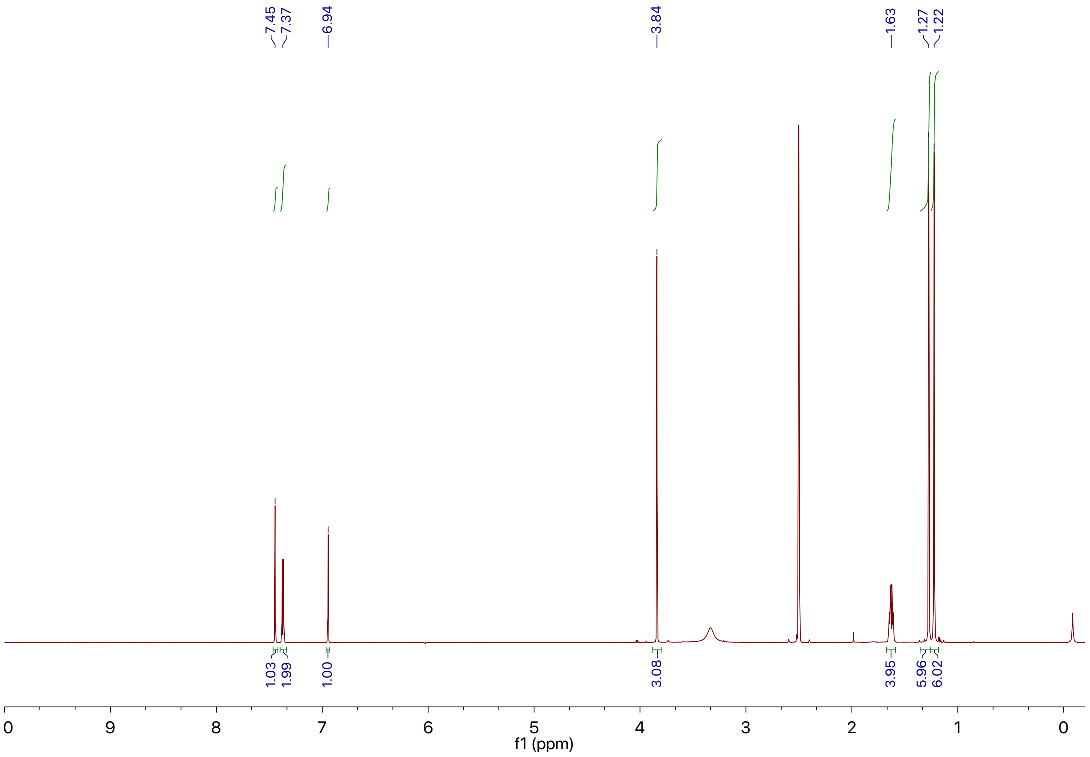

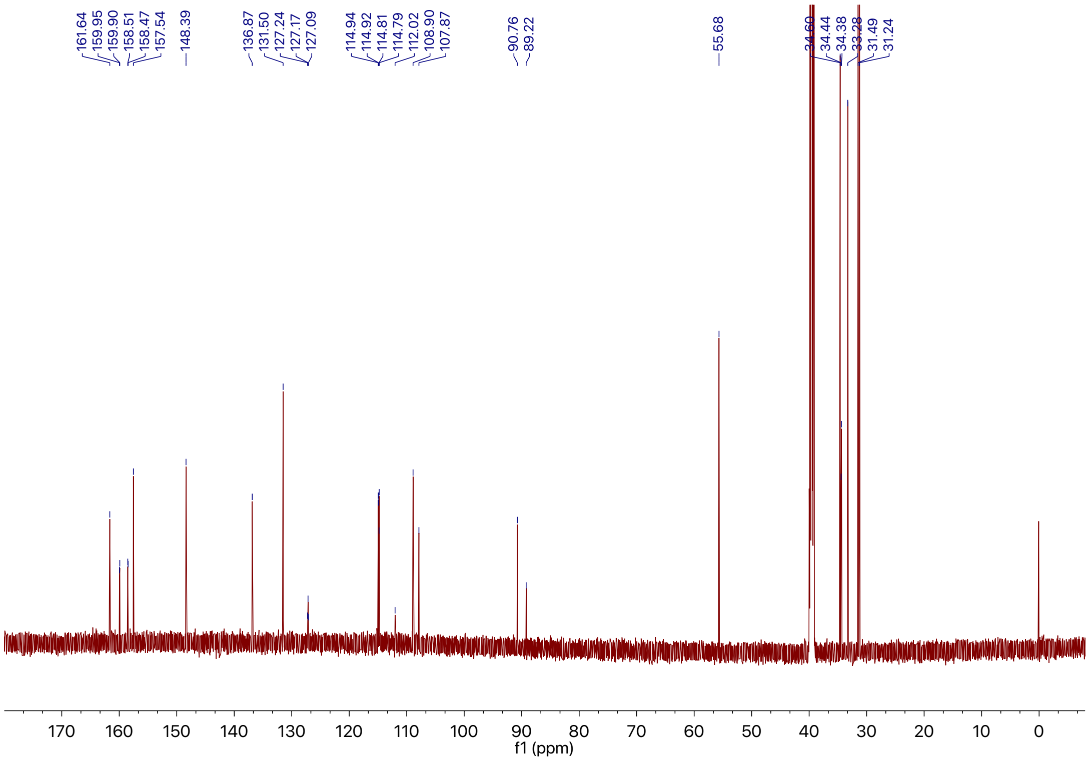

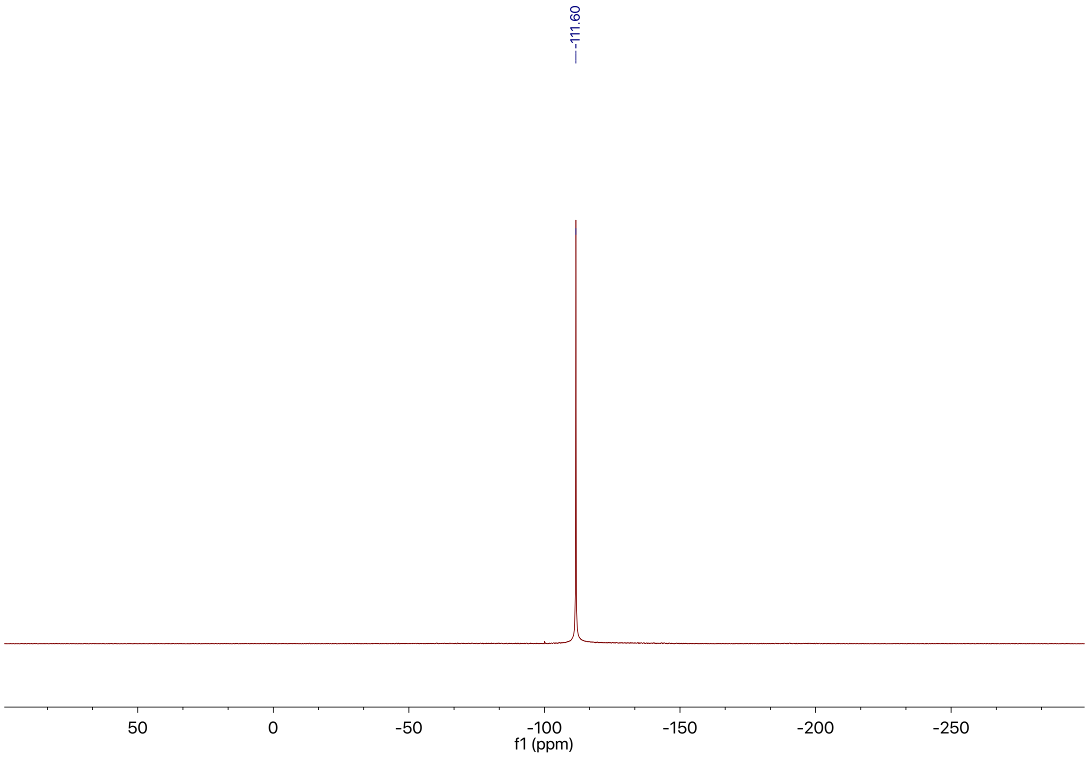

-111.60
50
0
-50
-100
-150
-200
-250
f1 (ppm)

Figure. S31. Chemical structure and NMR spectra of 6-[2-(5,5,8,8-Tetramethyl-5,6,7,8-tetrahydronaphthalen-2-yl)ethynyl]pyridine-3-carboxylic acid, 23

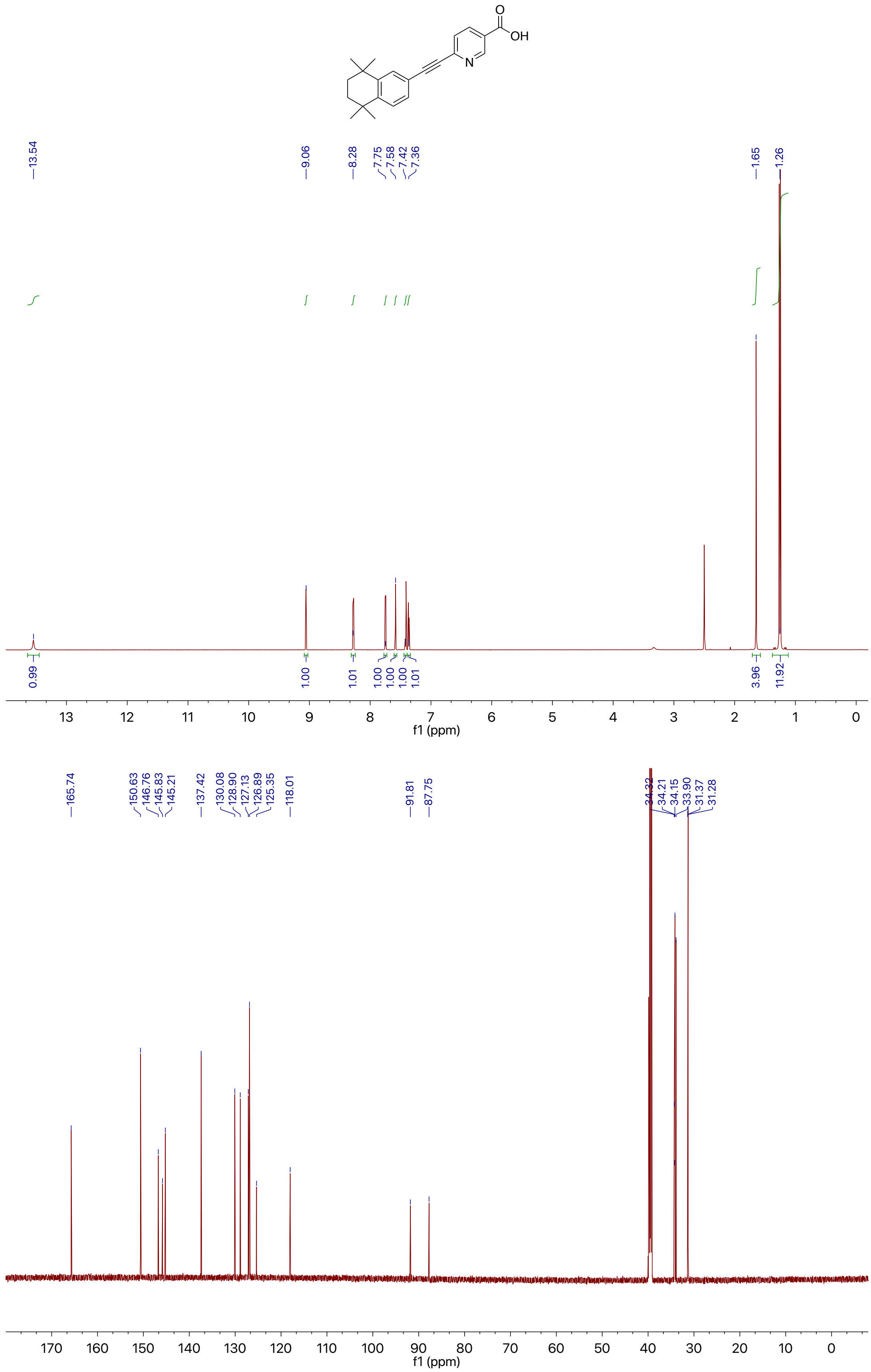

Figure. S32. Chemical structure and NMR spectra of 5-[2-(5,5,8,8-Tetramethyl-5,6,7,8-tetrahydronaphthalen-2-yl)ethynyl]pyridine-2-carboxylic acid, 24

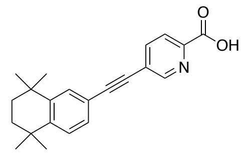

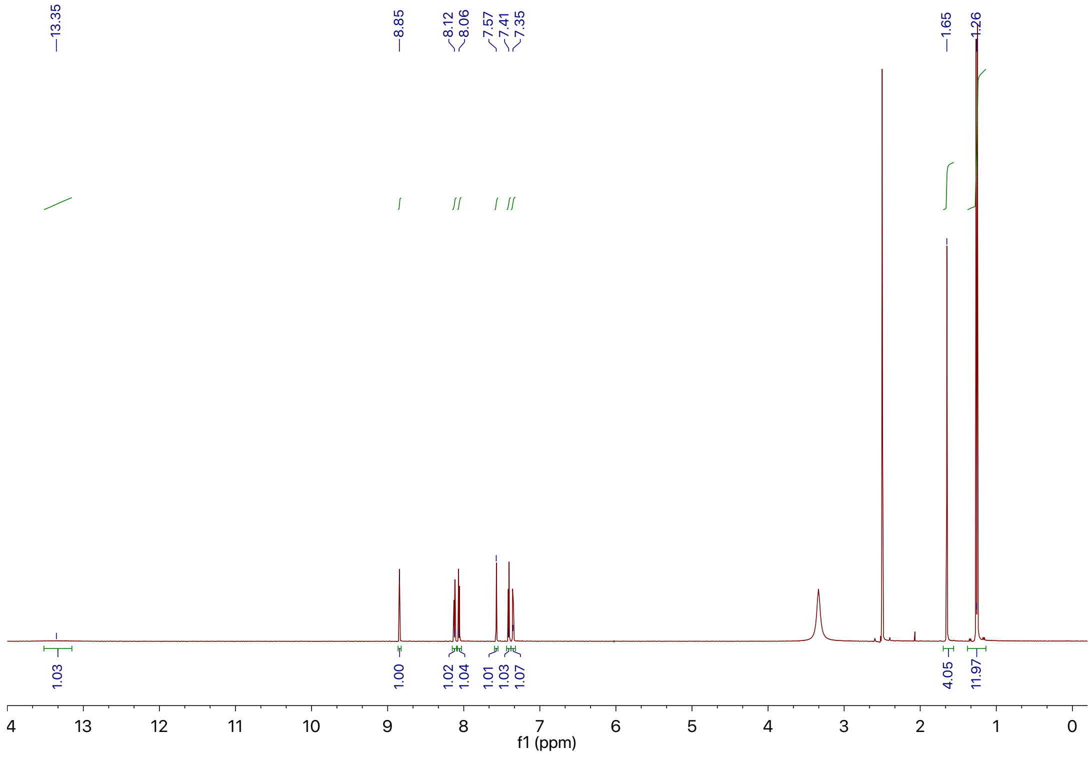

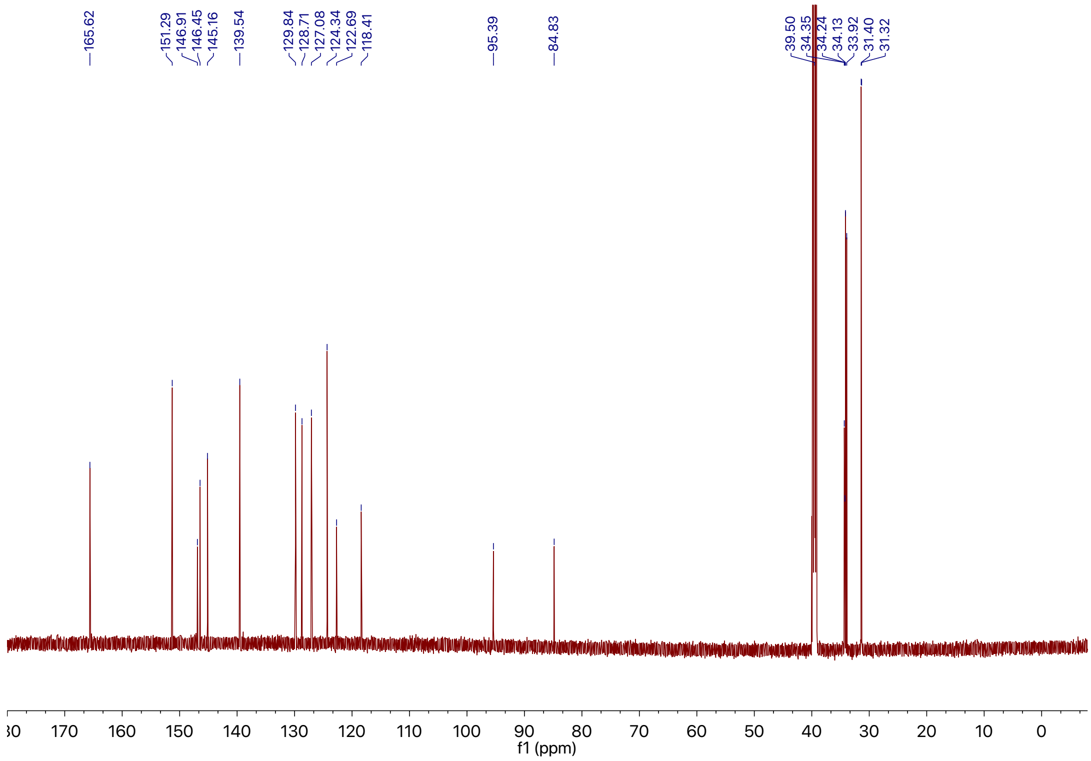

Figure. S33. Chemical structure and NMR spectra of 5-[2-(5,5,8,8-Tetramethyl-5,6,7,8-tetrahydronaphthalen-2-yl)ethynyl]pyrazine-2-carboxylic acid, 25

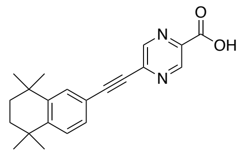

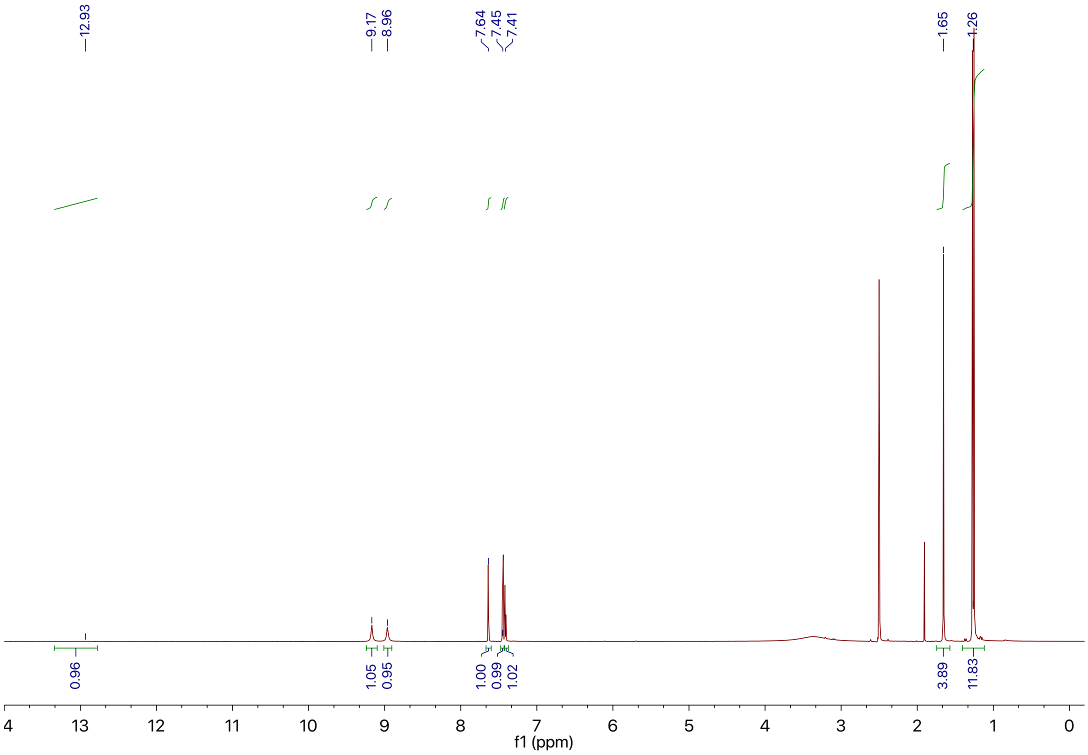

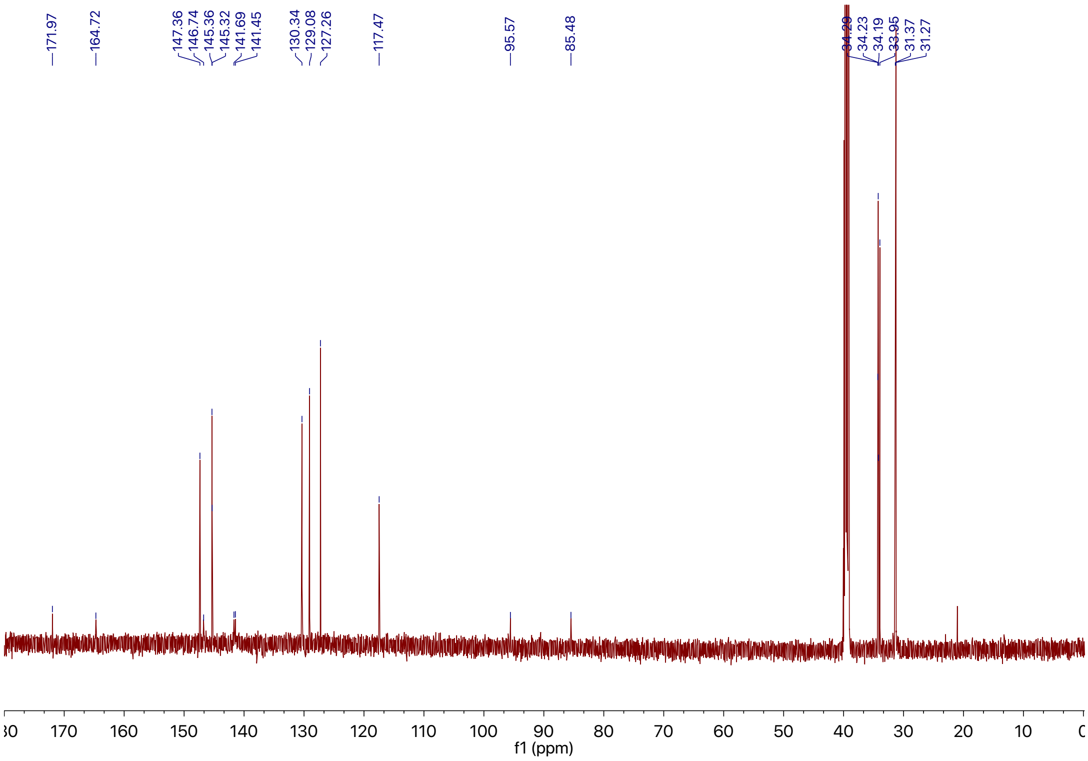

Figure. S34. Chemical structure and NMR spectra of 6-[2-(3,5,5,8,8-Pentamethyl-5,6,7,8-tetrahydronaphthalen-2-yl)ethynyl]pyridine-3-carboxylic acid, 26

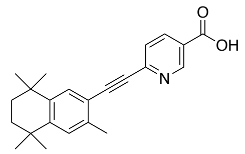

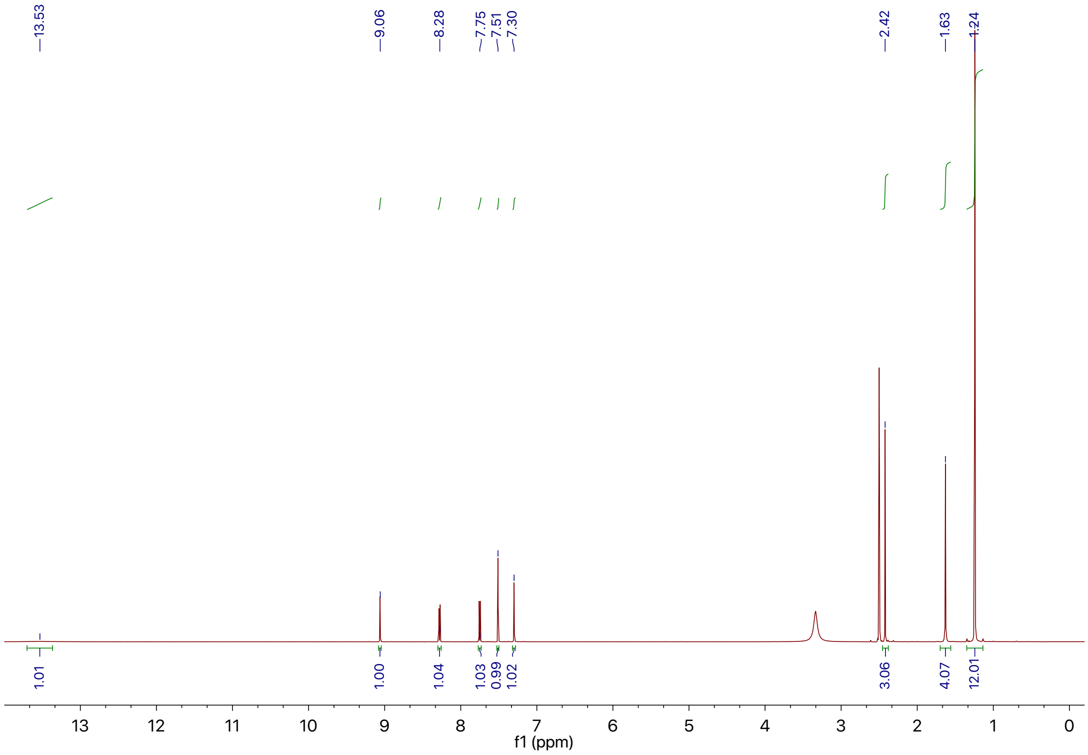

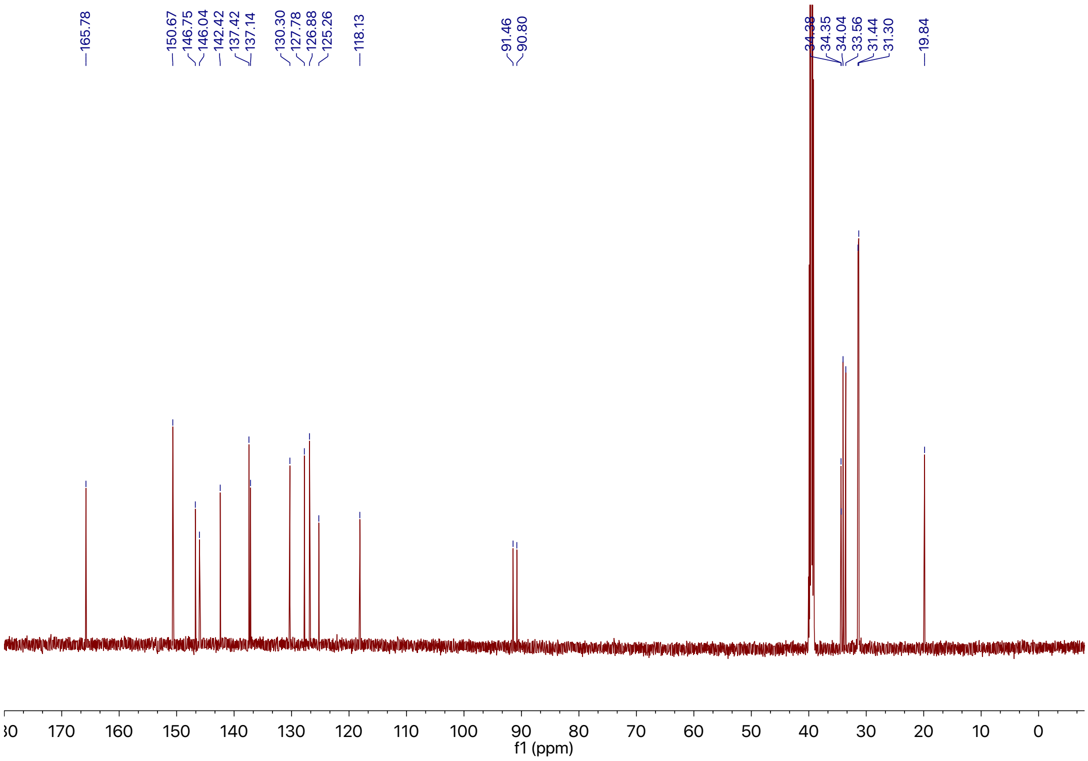

Figure. S35. Chemical structure and NMR spectra of 6-[2-(3-Methoxy-5,5,8,8-tetramethyl-5,6,7,8-tetrahydronaphthalen-2-yl)ethynyl]pyridine-3-carboxylic acid, 27

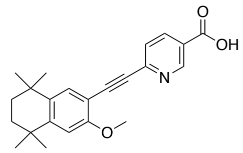

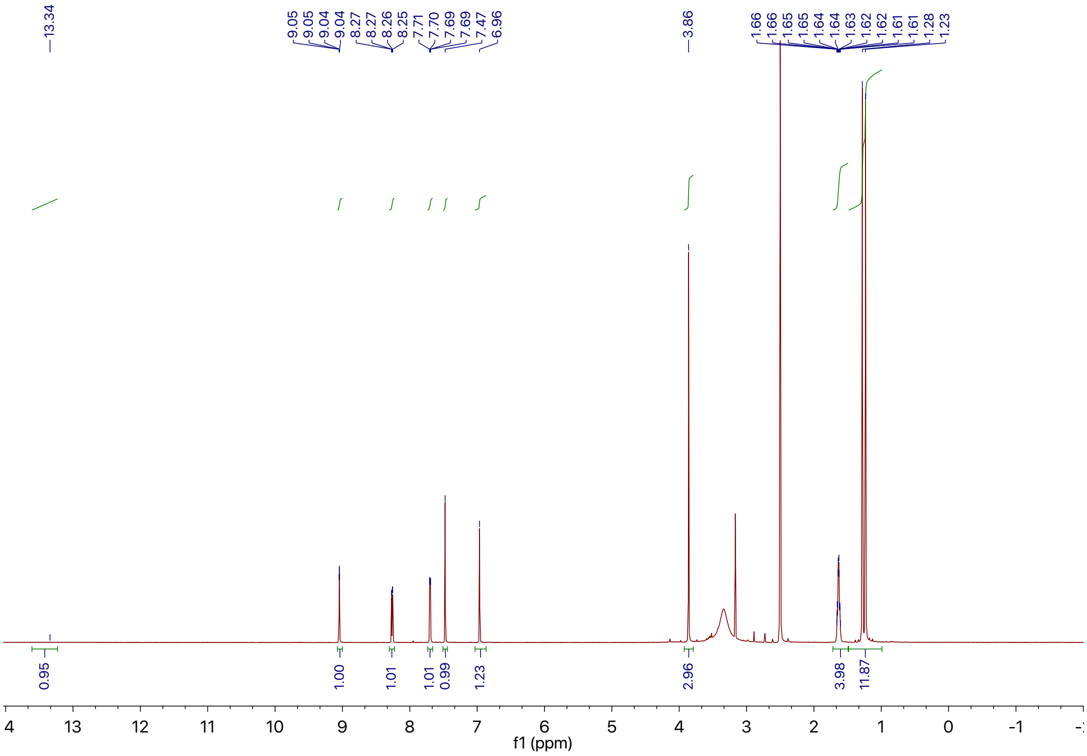

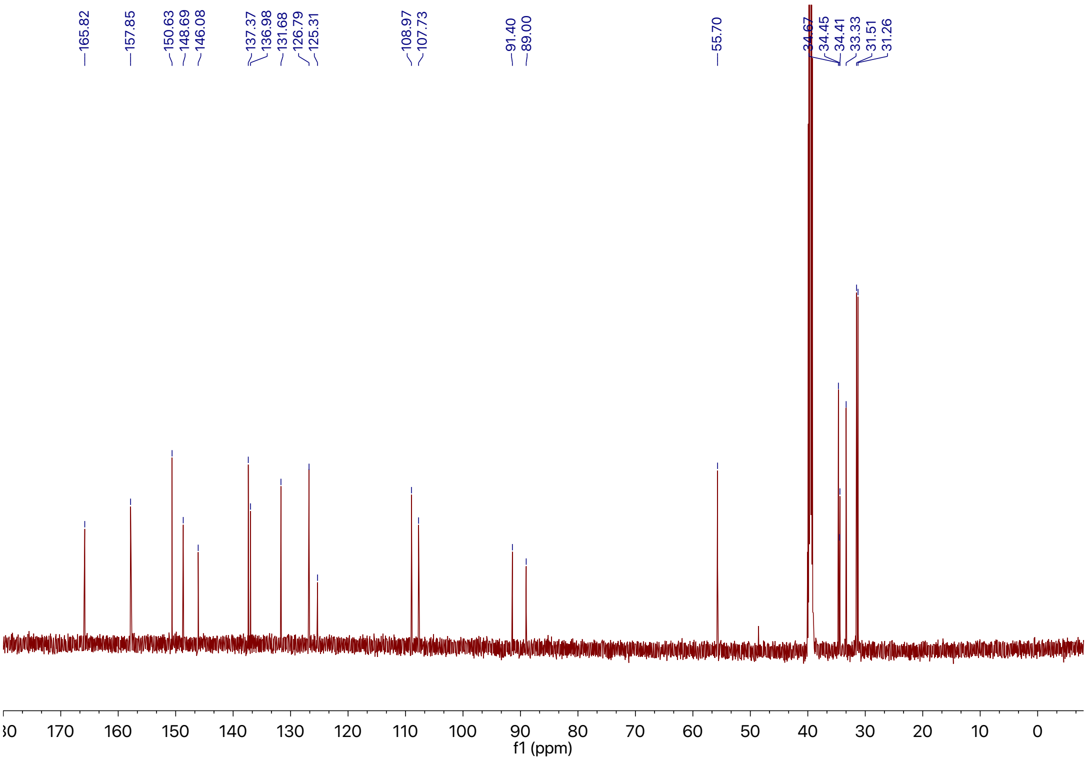

Figure. S36. Chemical structure and NMR spectra of 2-[2-(3,5,5,8,8-Pentamethyl-5,6,7,8-tetrahydronaphthalen-2-yl)ethynyl]pyrimidine-5-carboxylic acid, 28

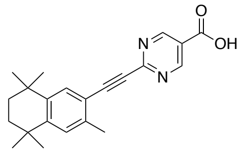

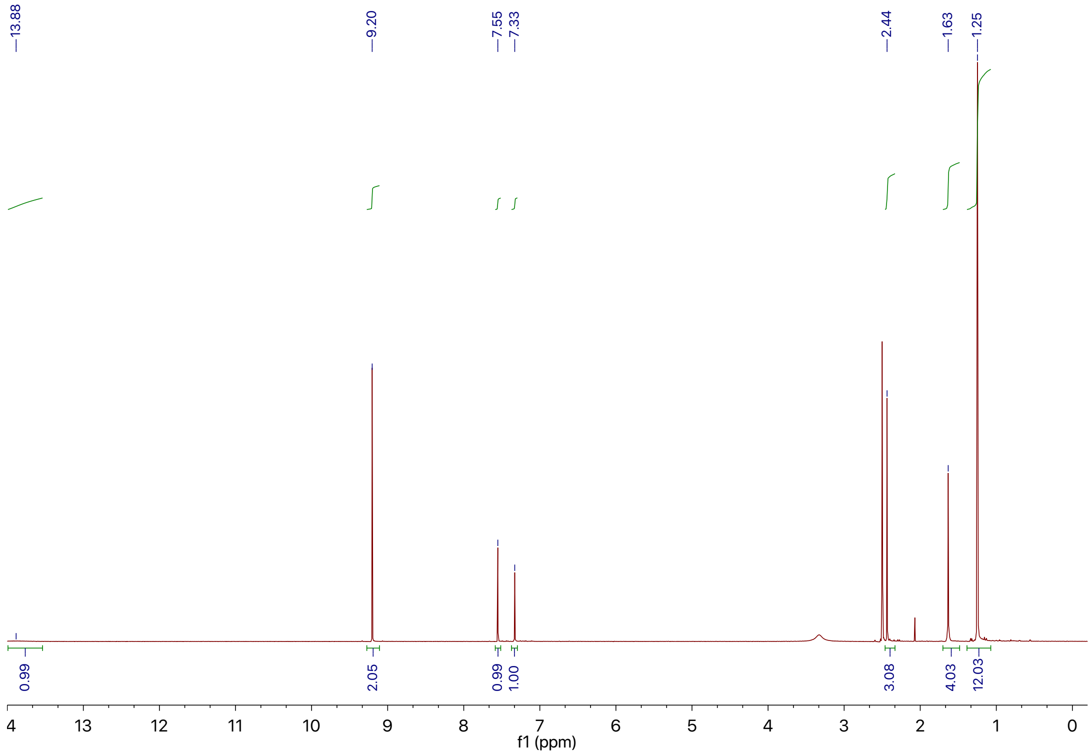

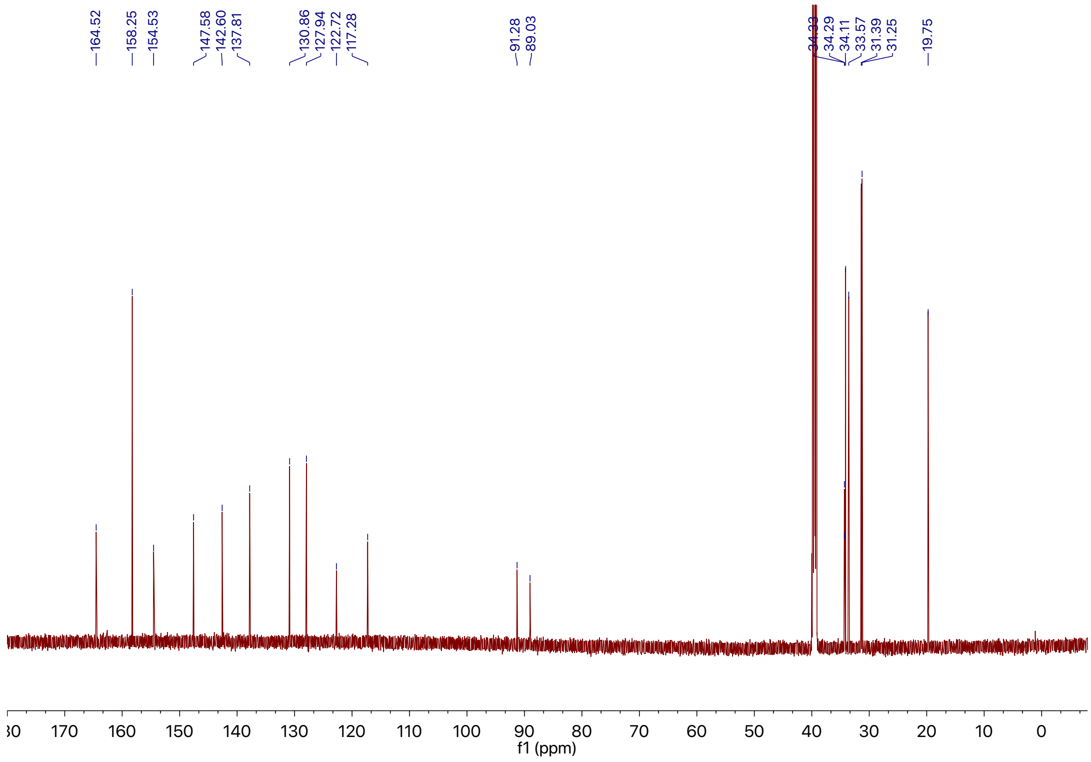

Figure. S37. Chemical structure and NMR spectra of 2-[2-(3-Methoxy-5,5,8,8-tetramethyl-5,6,7,8-tetrahydronaphthalen-2-yl)ethynyl]pyrimidine-5-carboxylic acid, 29

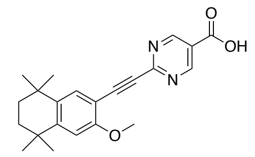

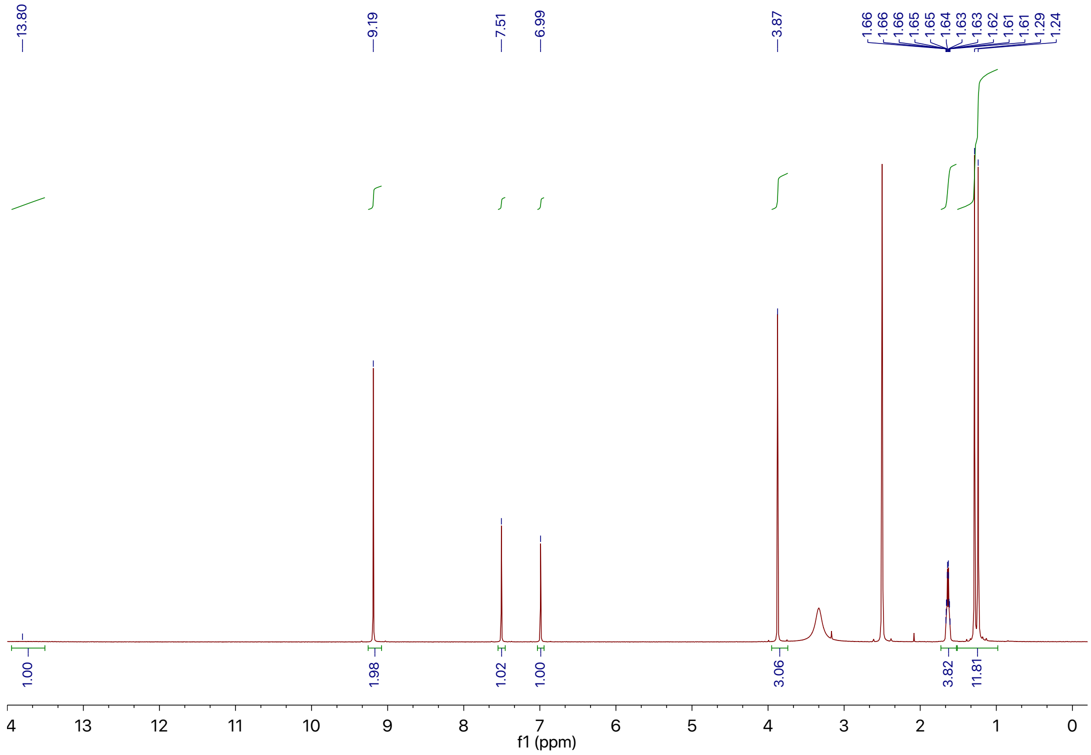

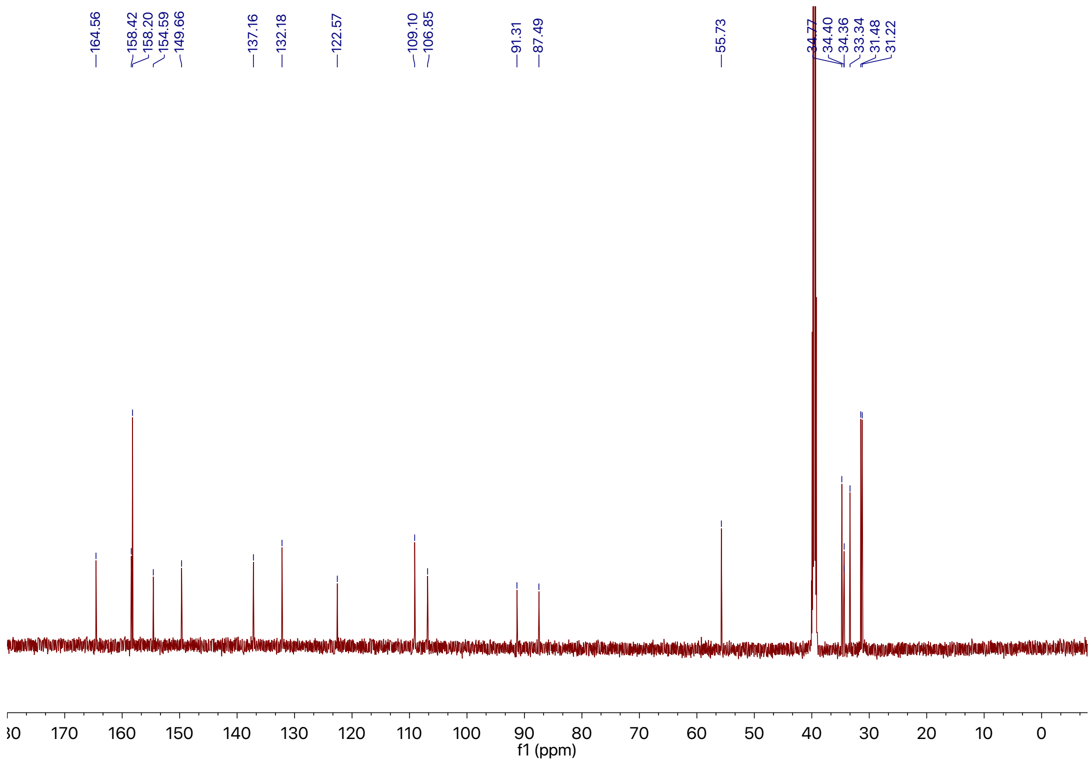

Figure. S38. Chemical structure and NMR spectra of 4-[2-(5,5,8,8-Tetramethyl-5,6,7,8-tetrahydroquinoxalin-2-yl)ethynyl]benzoic acid, 30

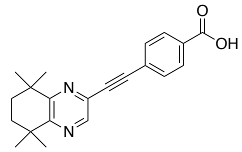

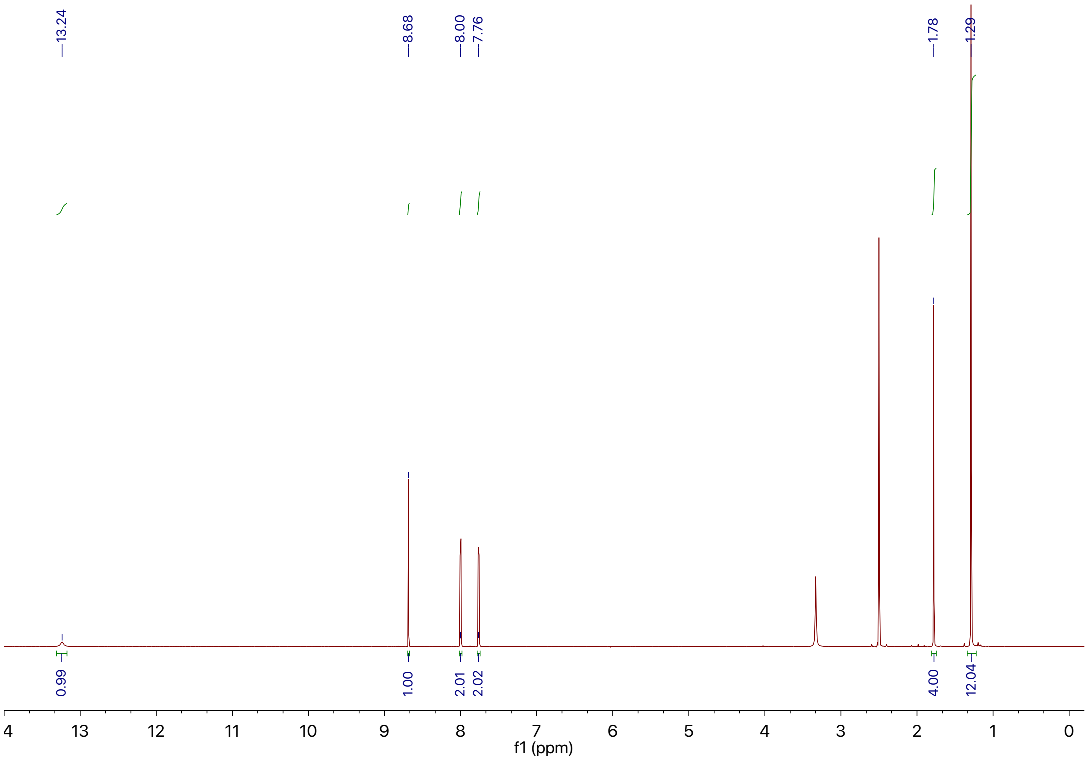

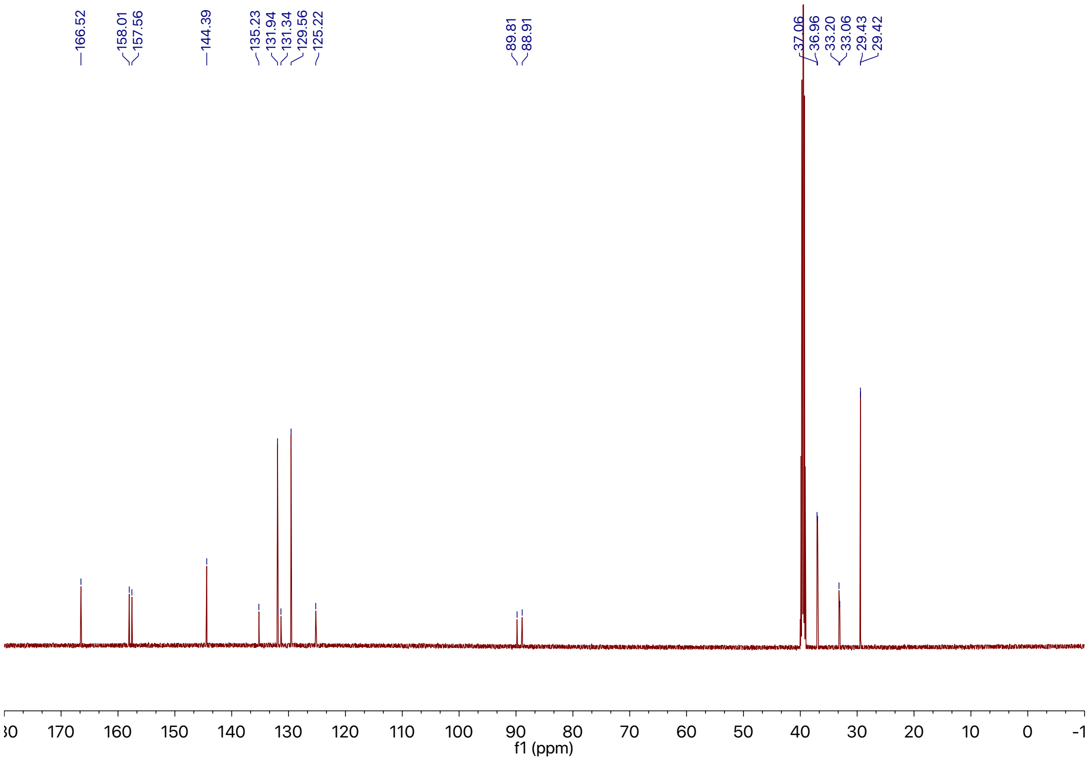

Figure. S39. Chemical structure and NMR spectra of 3-Fluoro-4-[2-(5,5,8,8-tetramethyl-5,6,7,8-tetrahydroquinoxalin-2-yl)ethynyl]benzoic acid, 31

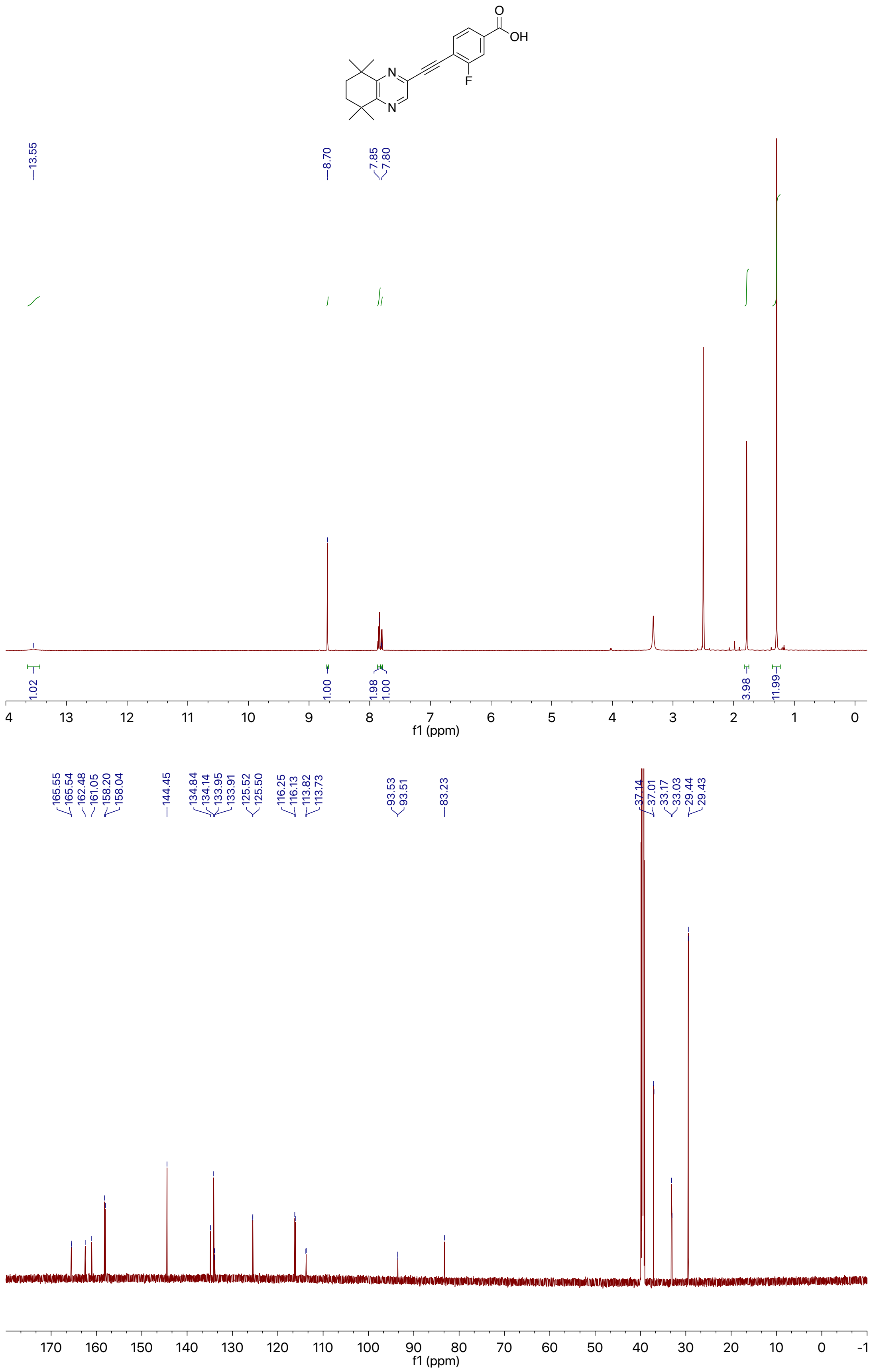

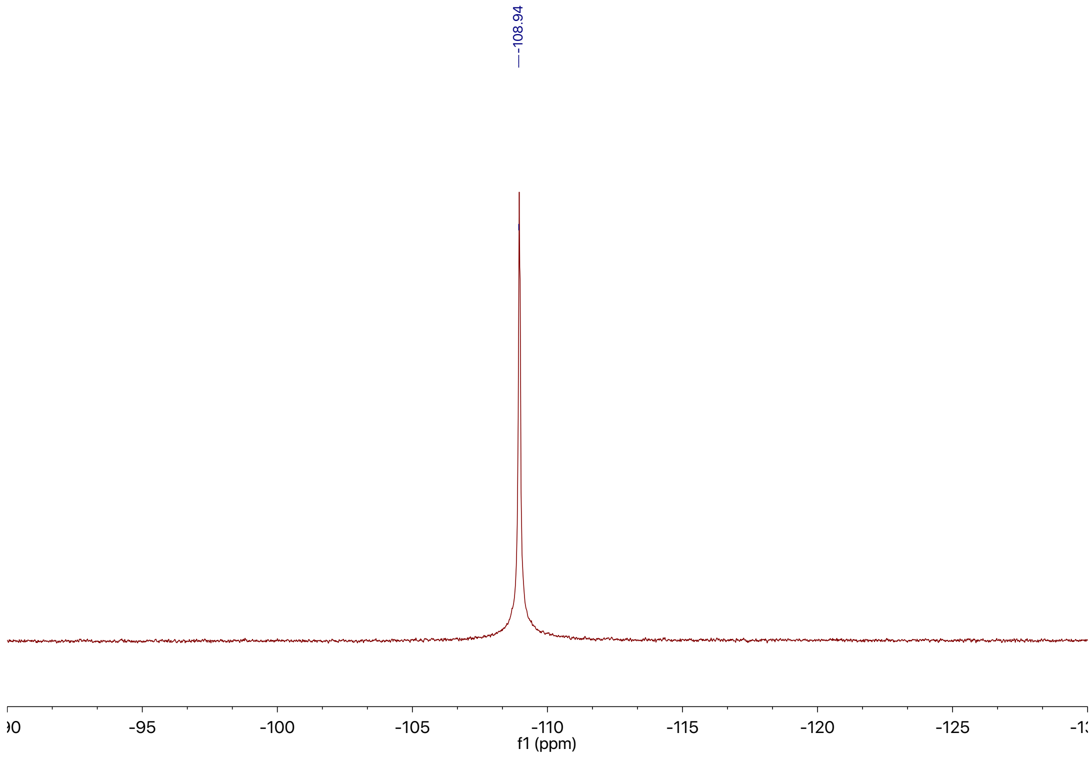

-108.94
-95
-100
-105
-110
-115
-120
-125
f1 (ppm)

Figure. S40. Chemical structure and NMR spectra of 1-(5,5,8,8-Tetramethyl-5,6,7,8-tetrahydroquinoxalin-2-yl)propan-1-ol, 32

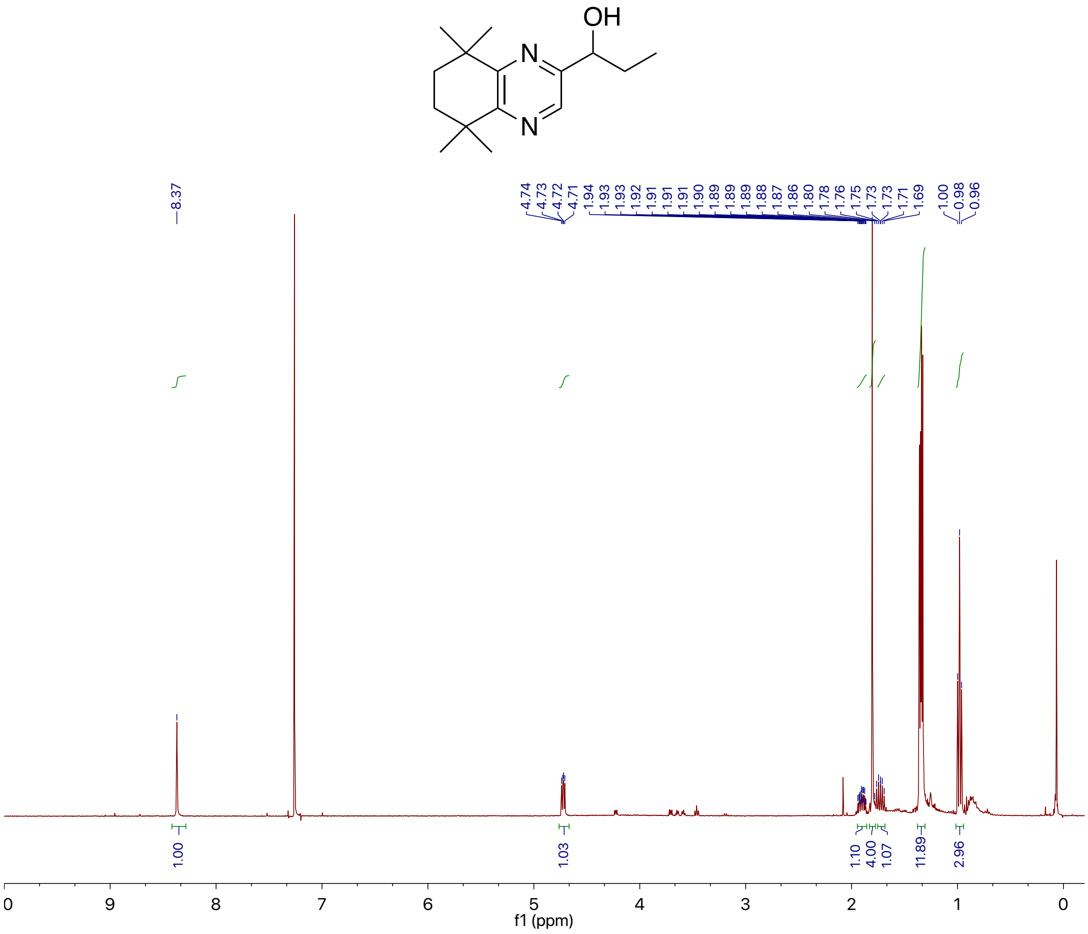

Figure. S41. Chemical structure and NMR spectra of 1-(5,5,8,8-Tetramethyl-5,6,7,8-tetrahydroquinoxalin-2-yl)propan-1-one, 34

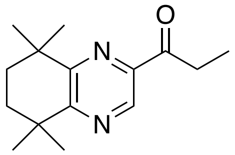

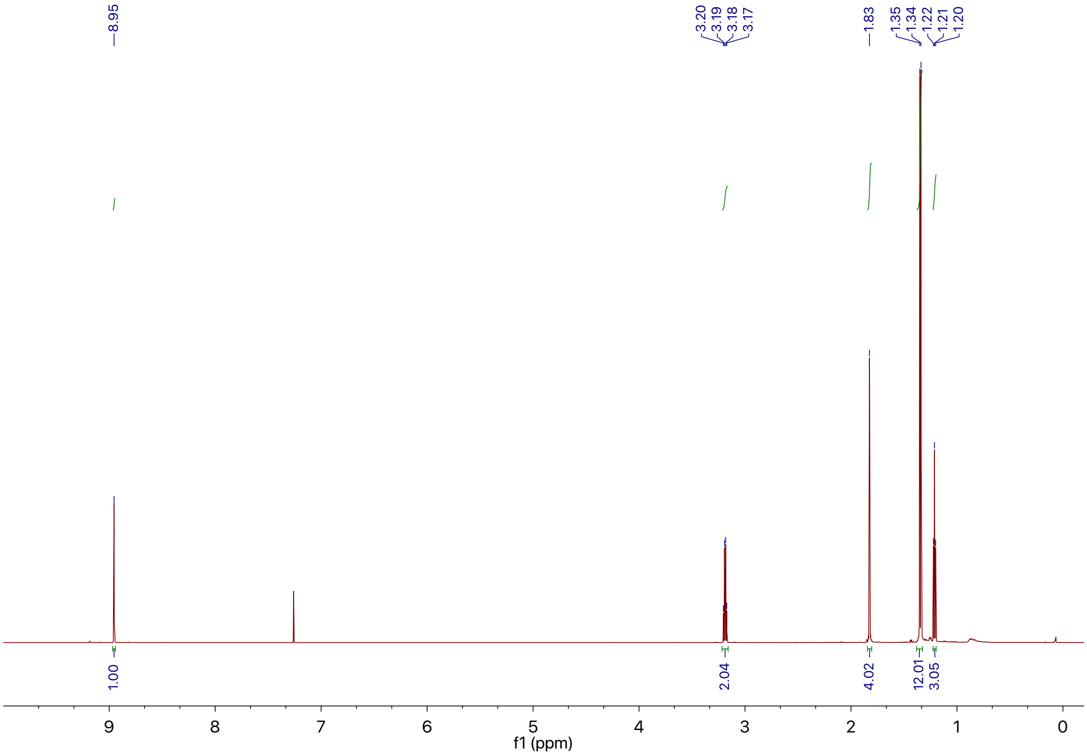

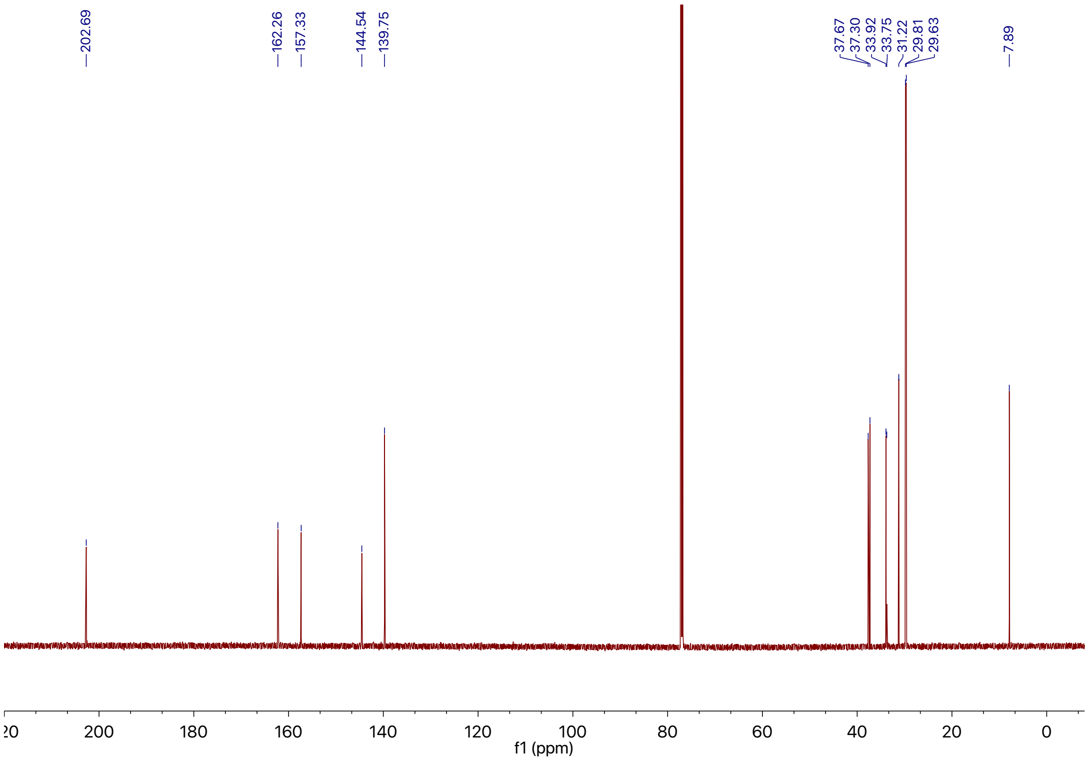

Figure. S42. Chemical structure and NMR spectra of 2-Bromo-1-(5,5,8,8-tetramethyl-5,6,7,8-tetrahydroquinoxalin-2-yl)propan-1-one, 35

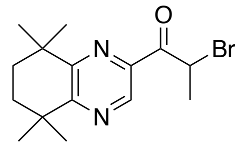

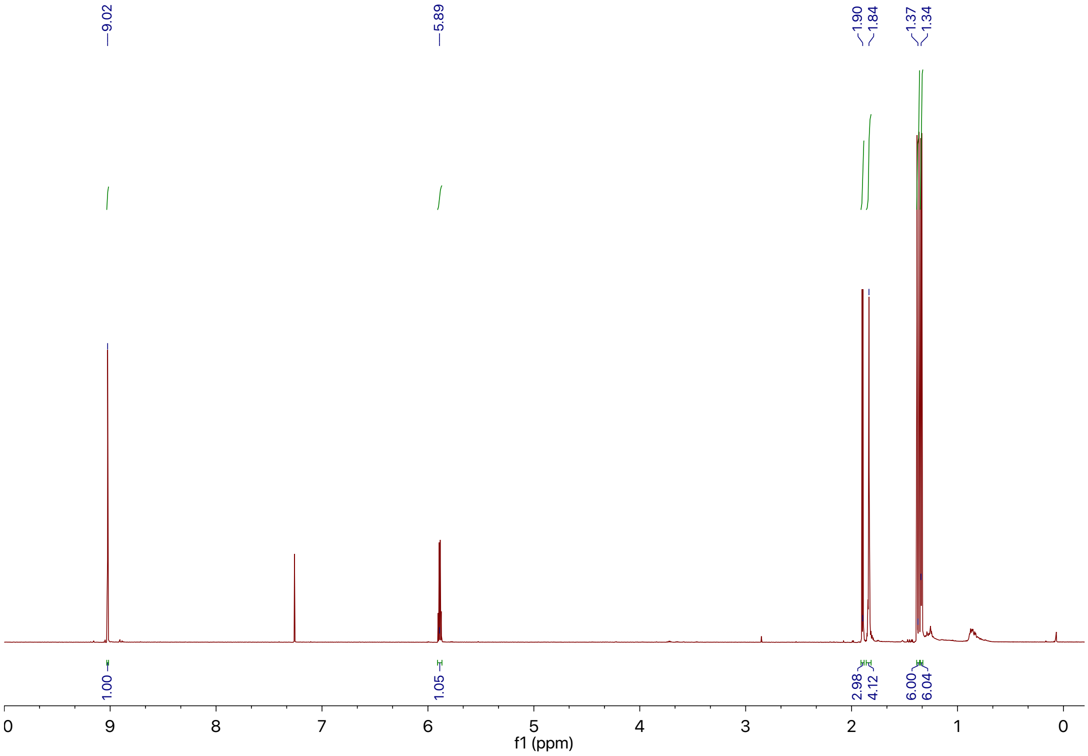

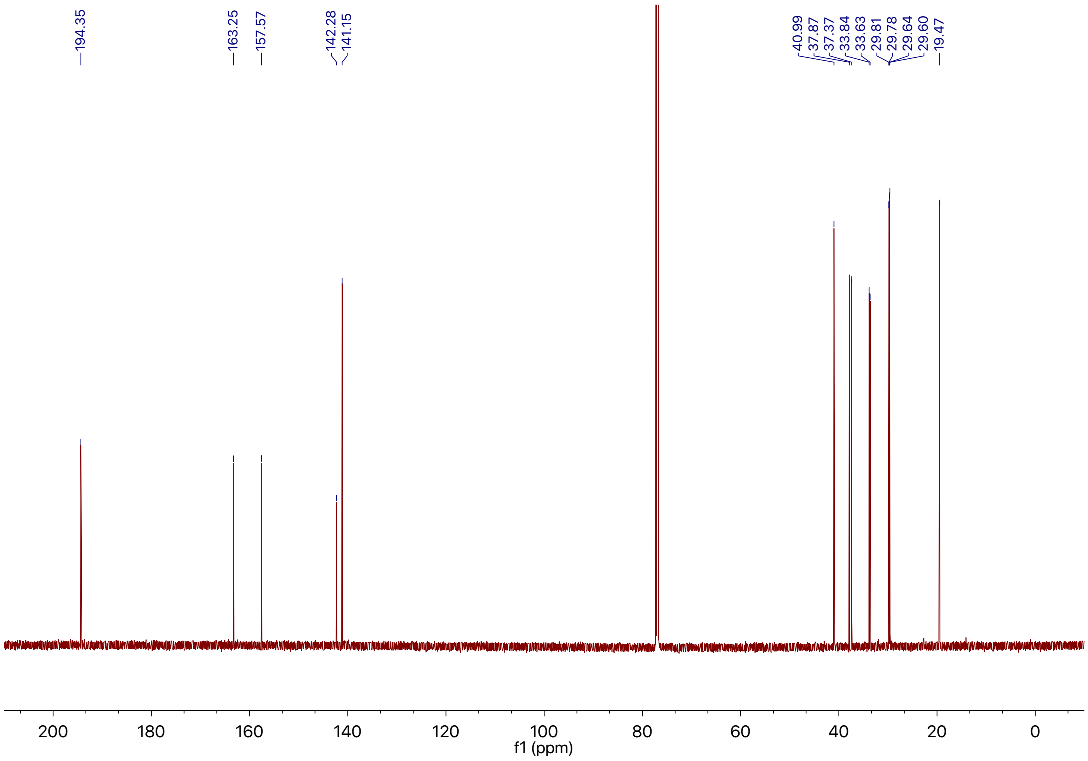

Figure. S43. Chemical structure and NMR spectra of Methyl 4-carbamothioylbenzoate, 36

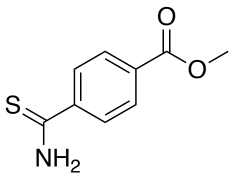

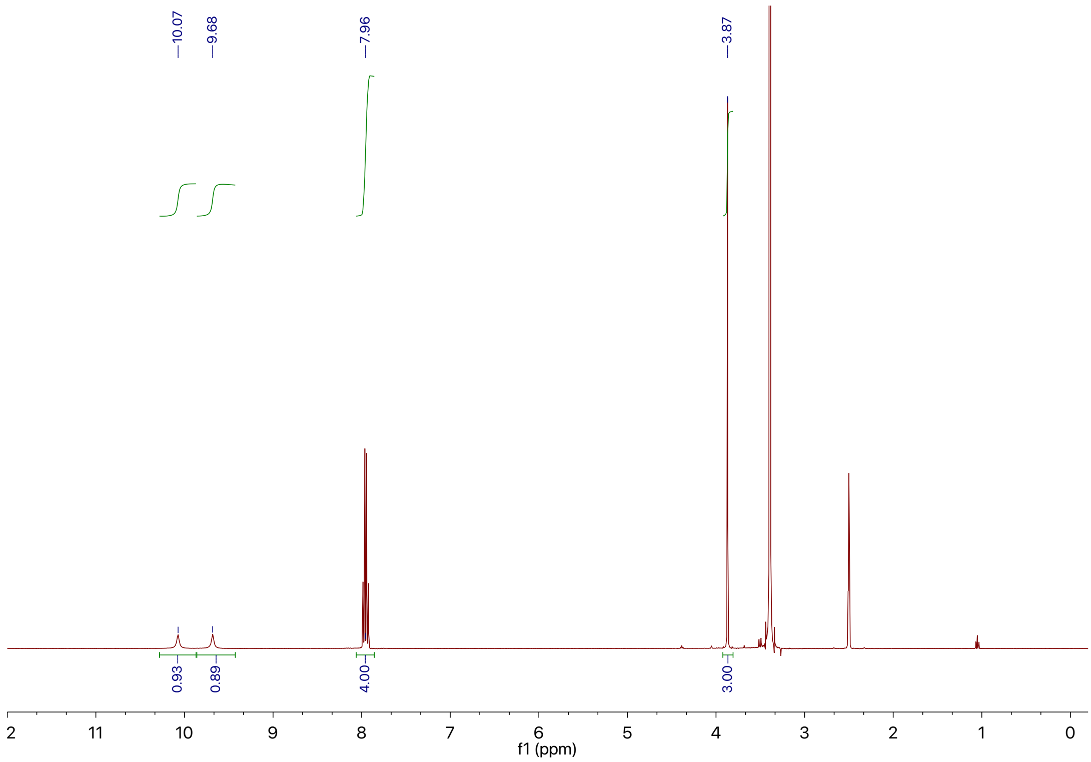

Figure. S44. Chemical structure and NMR spectra of Methyl 6-carbamothioylpyridine-3-carboxylate, 37

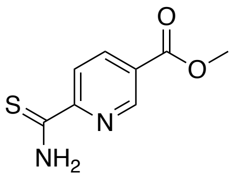

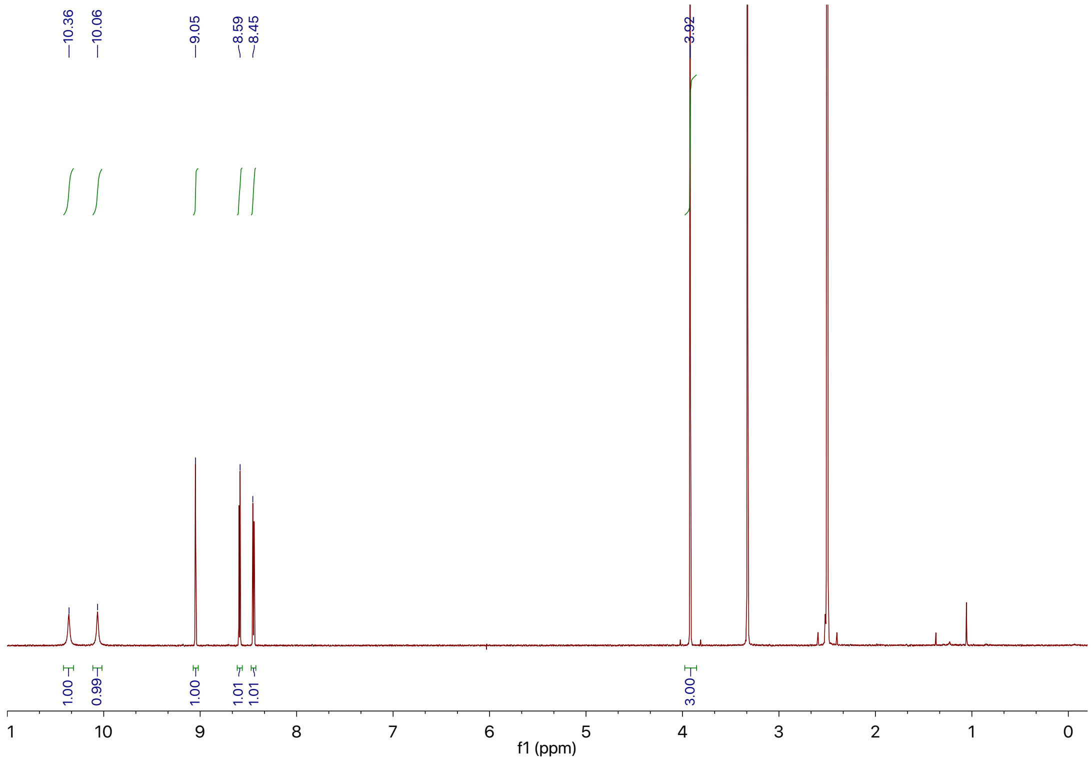

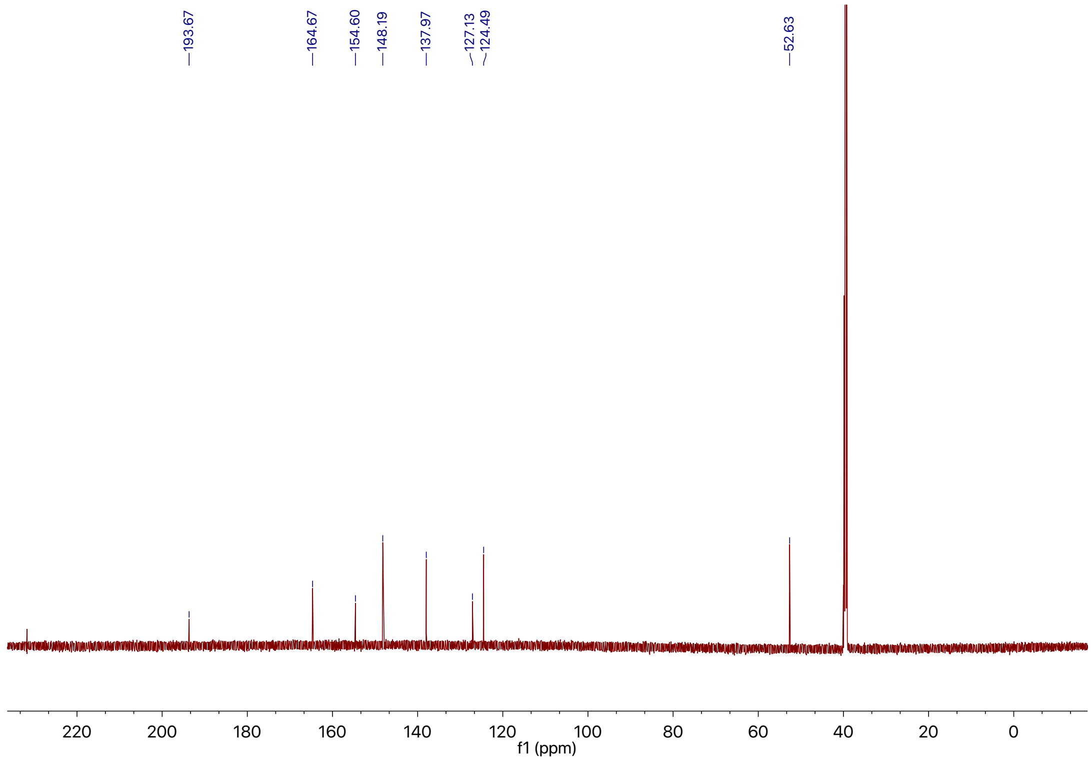

Figure. S45. Chemical structure and NMR spectra of Methyl 4-carbamothioyl-2-fluorobenzoate, 38

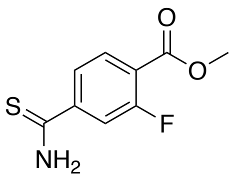

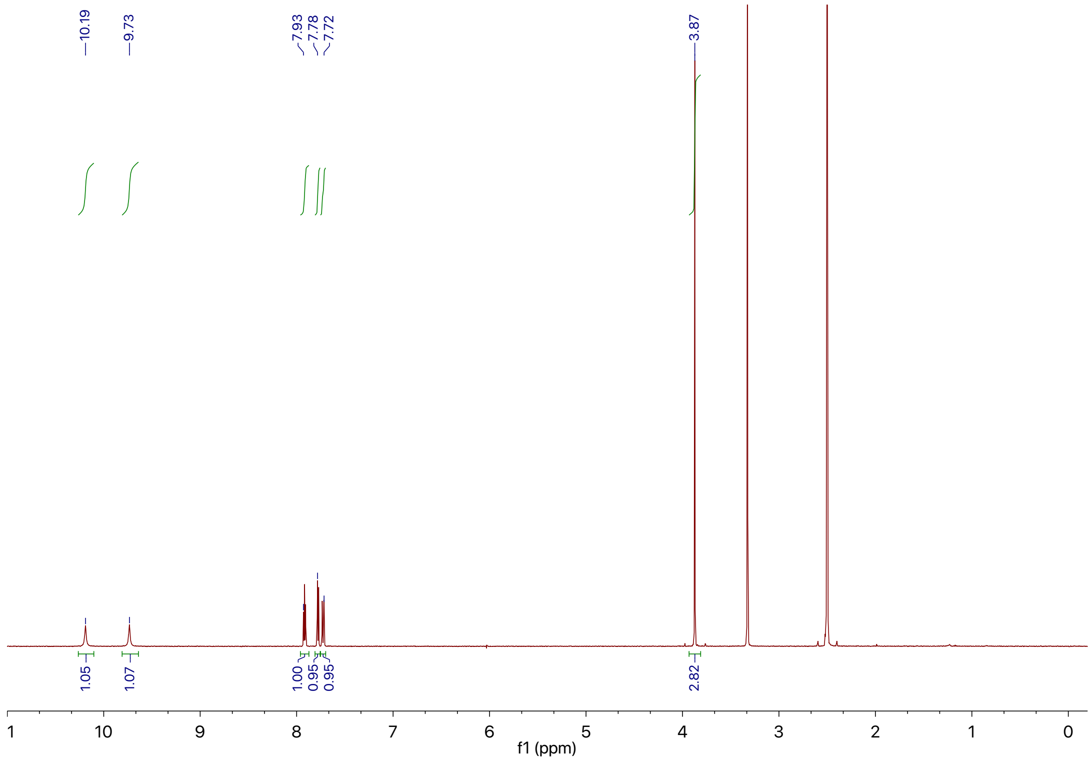

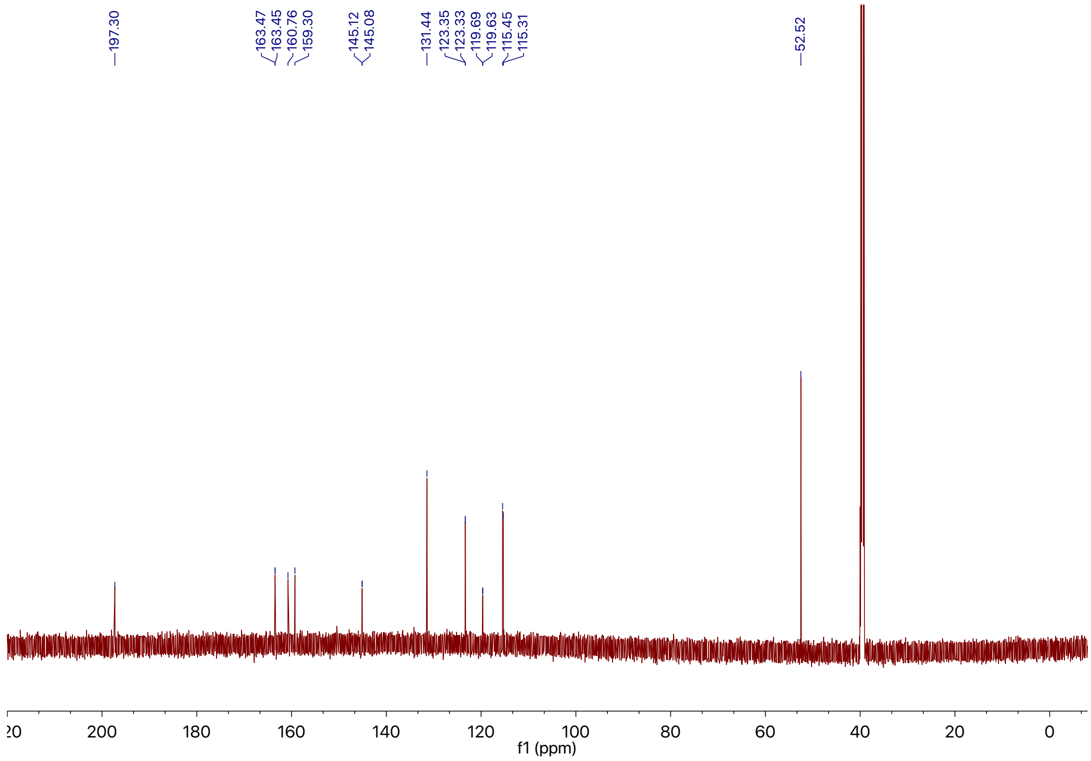

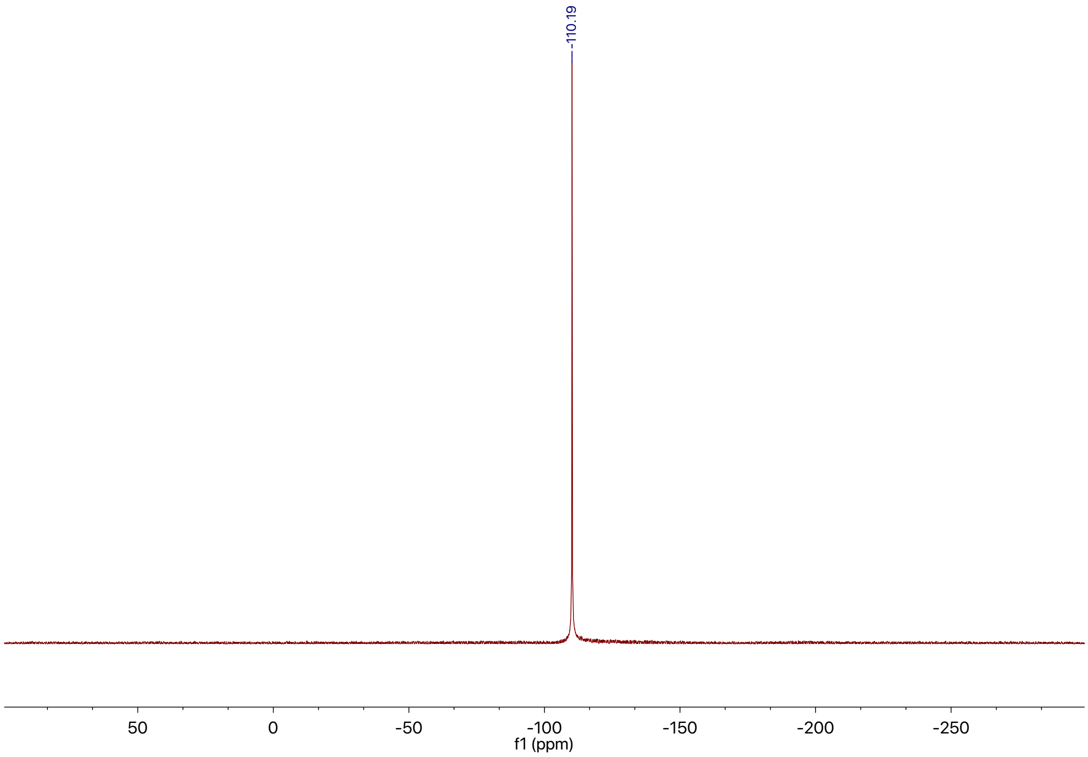

-110.19
50
0
-50
-100
-150
-200
-250
f1 (ppm)

Figure. S46. Chemical structure and NMR spectra of Methyl 4-carbamothioyl-3-fluorobenzoate, 39

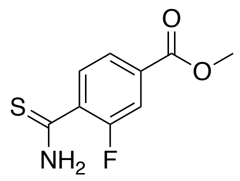

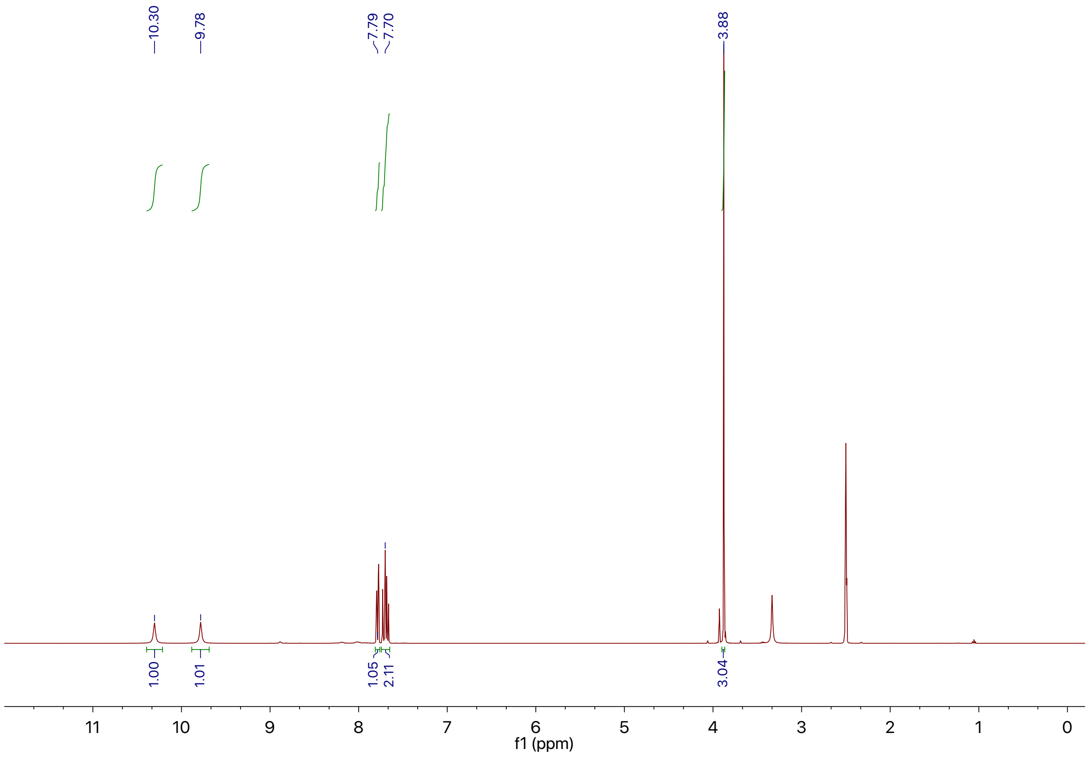

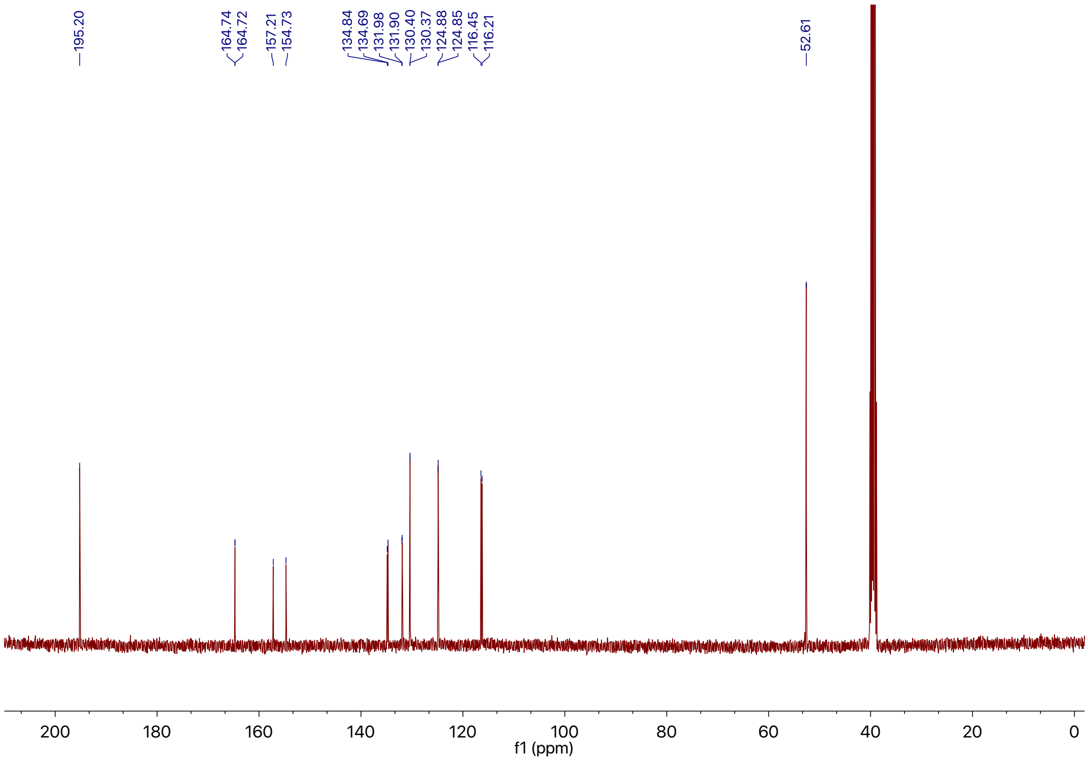

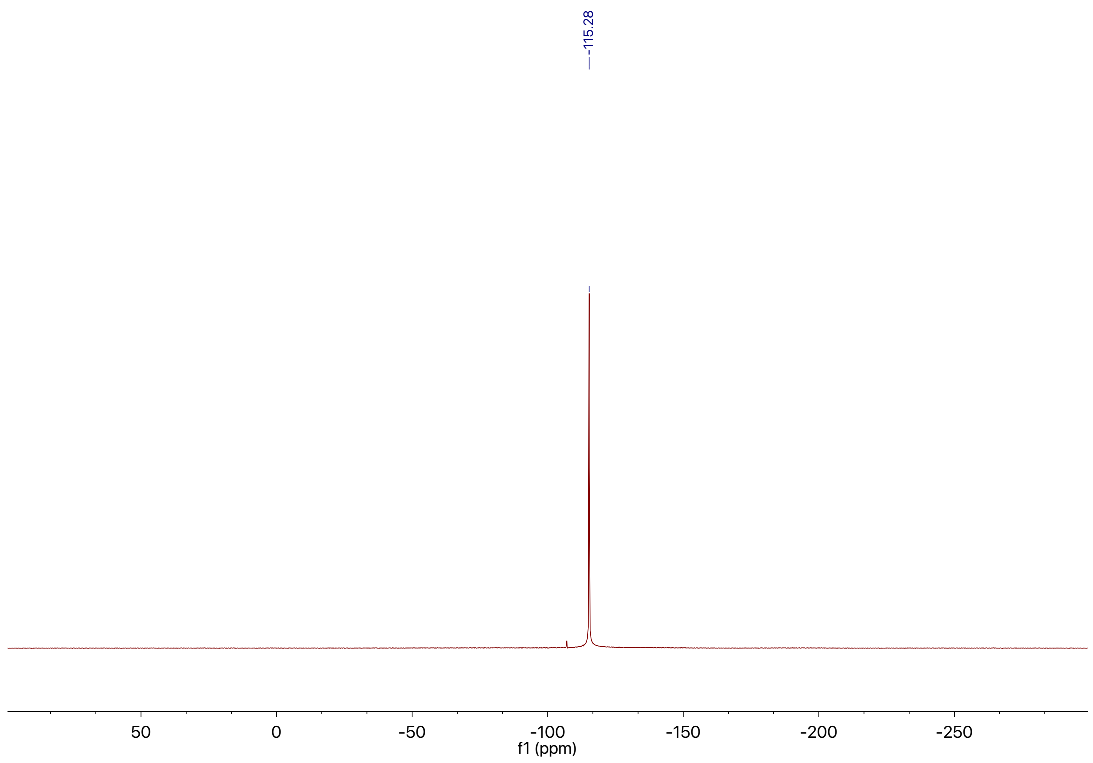
-115.28
50
0
-50
-100
-150
-200
-250
f1 (ppm)

Figure. S47. Chemical structure and NMR spectra of 4-[5-Methyl-4-(5,5,8,8-tetramethyl-5,6,7,8-tetrahydroquinoxalin-2-yl)-1,3-thiazol-2-yl]benzoic acid, 40

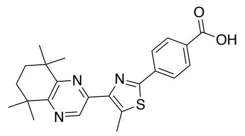

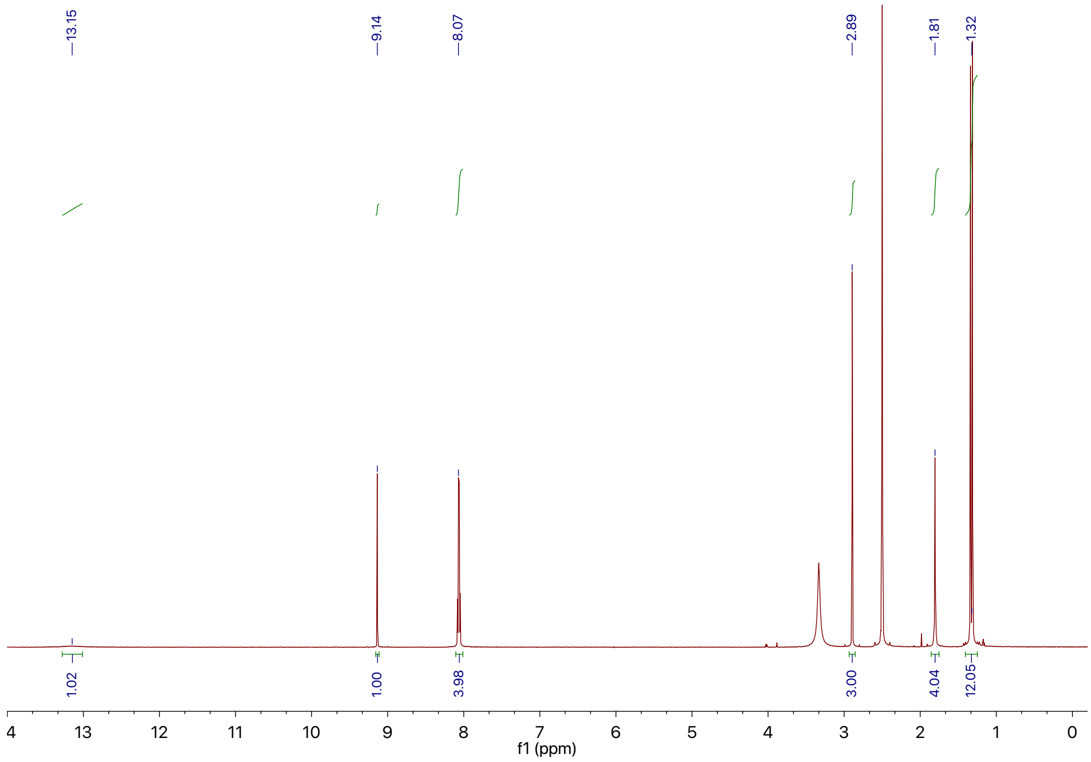

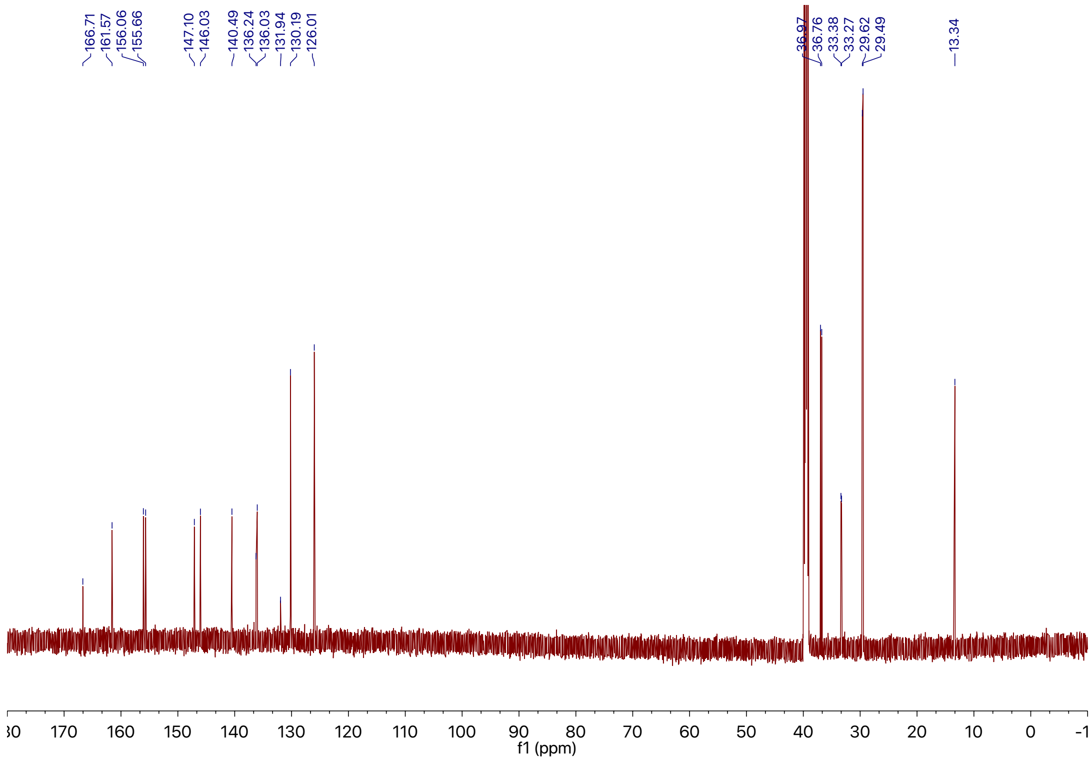

Figure. S48. Chemical structure and NMR spectra of 6-[5-Methyl-4-(5,5,8,8-tetramethyl-5,6,7,8-tetrahydroquinoxalin-2-yl)-1,3-thiazol-2-yl]pyridine-3-carboxylic acid, 41

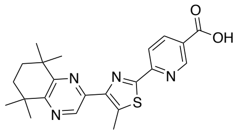

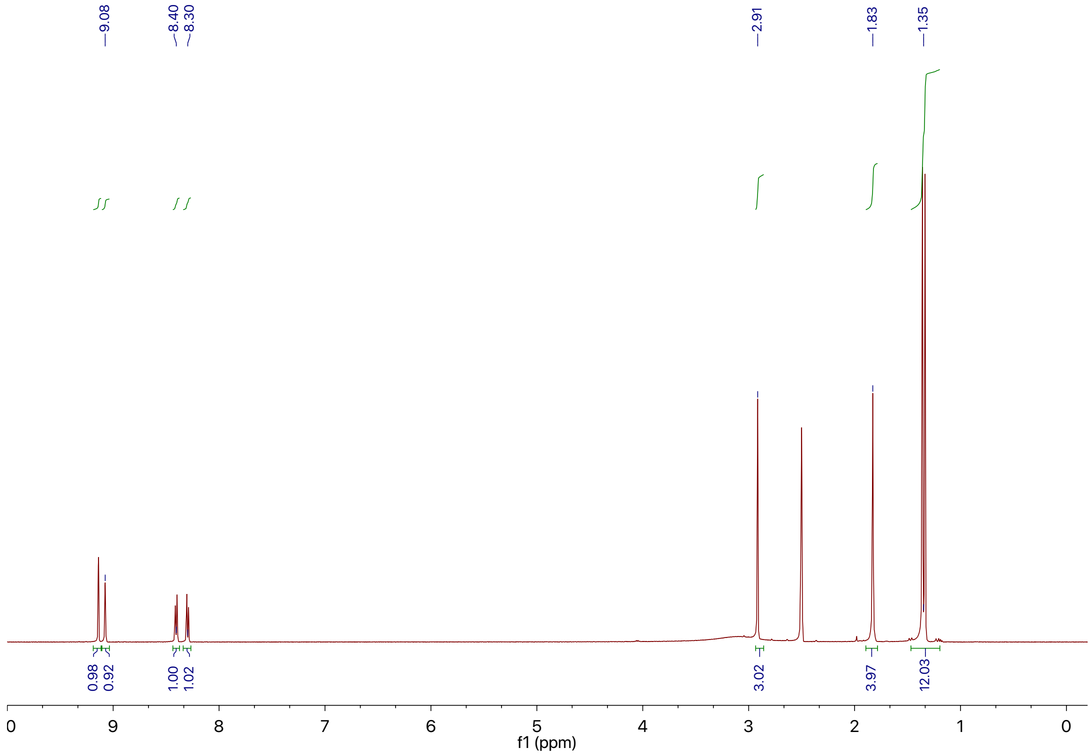

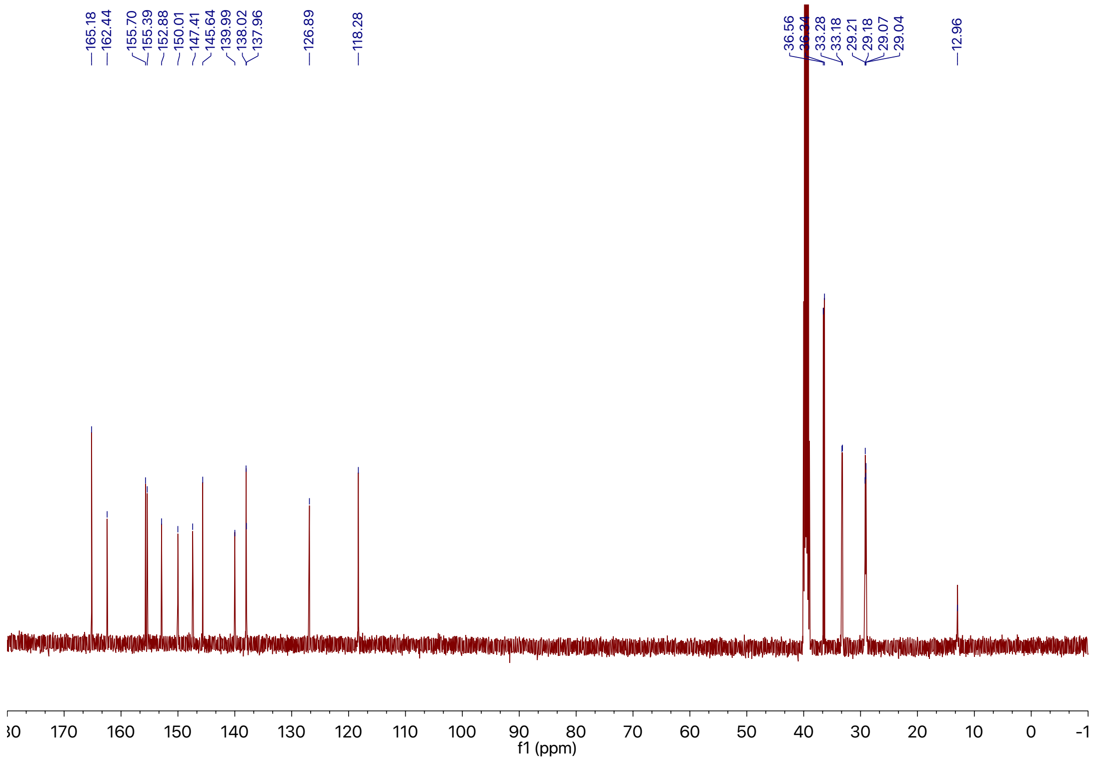

Figure. S49. Chemical structure and NMR spectra of 2-Fluoro-4-[5-methyl-4-(5,5,8,8-tetramethyl-5,6,7,8-tetrahydroquinoxalin-2-yl)-1,3-thiazol-2-yl]benzoic acid, 42

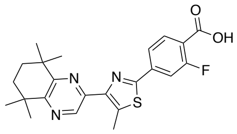

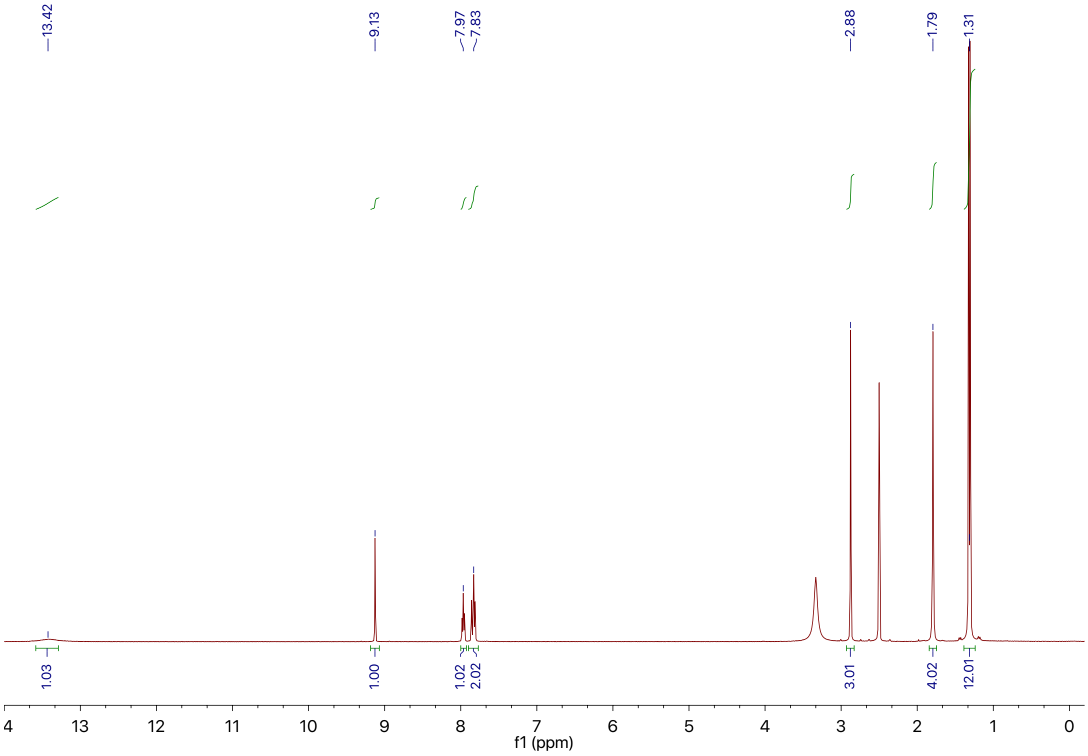

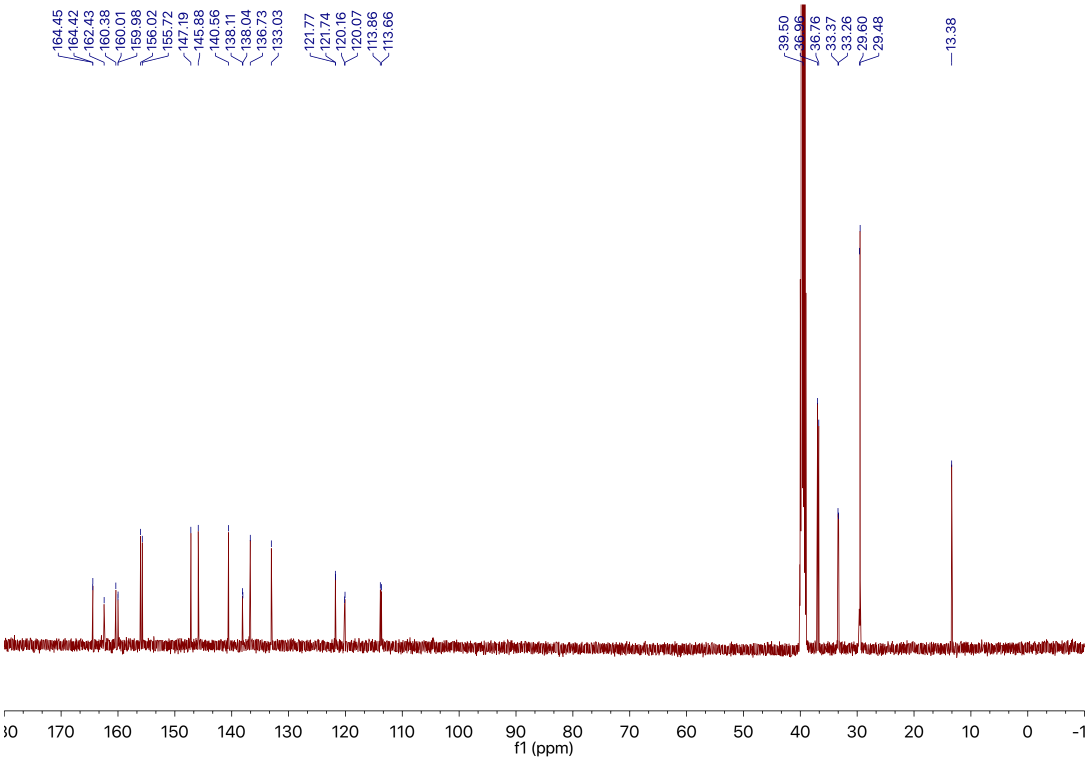

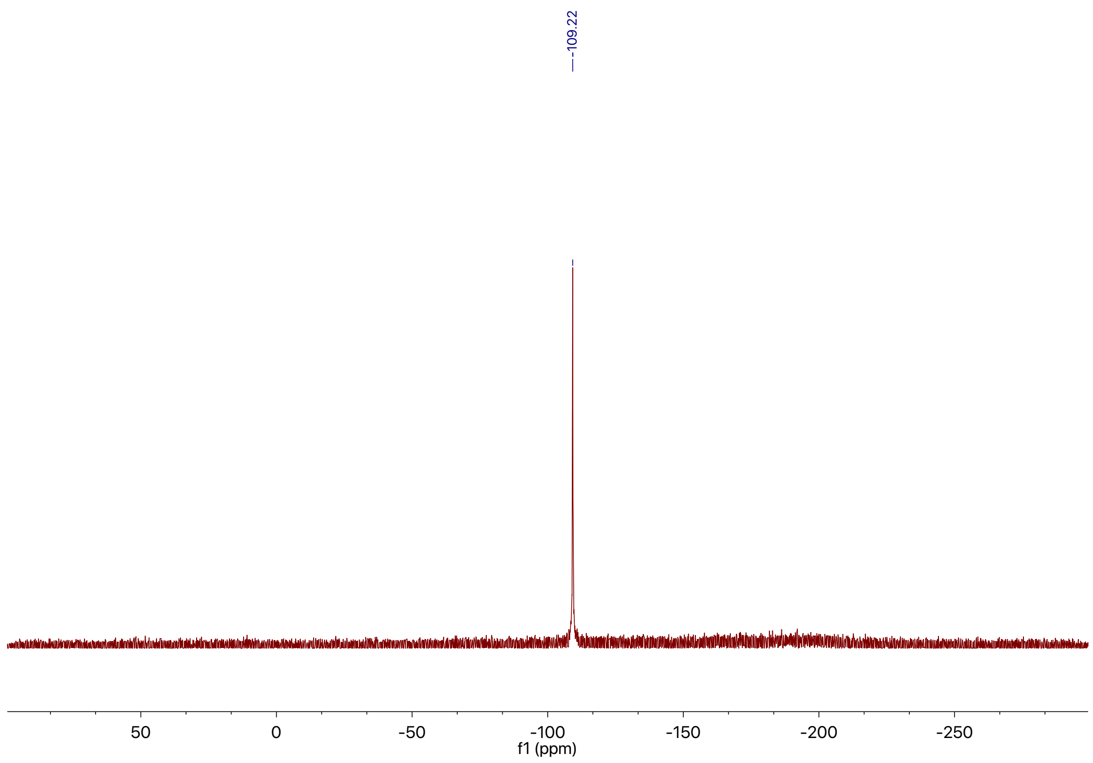

-109.22
50
0
-50
-100
-150
-200
-250
f1 (ppm)

Figure. S50. Chemical structure and NMR spectra of 3-Fluoro-4-[5-methyl-4-(5,5,8,8-tetramethyl-5,6,7,8-tetrahydroquinoxalin-2-yl)-1,3-thiazol-2-yl]benzoic acid, 43

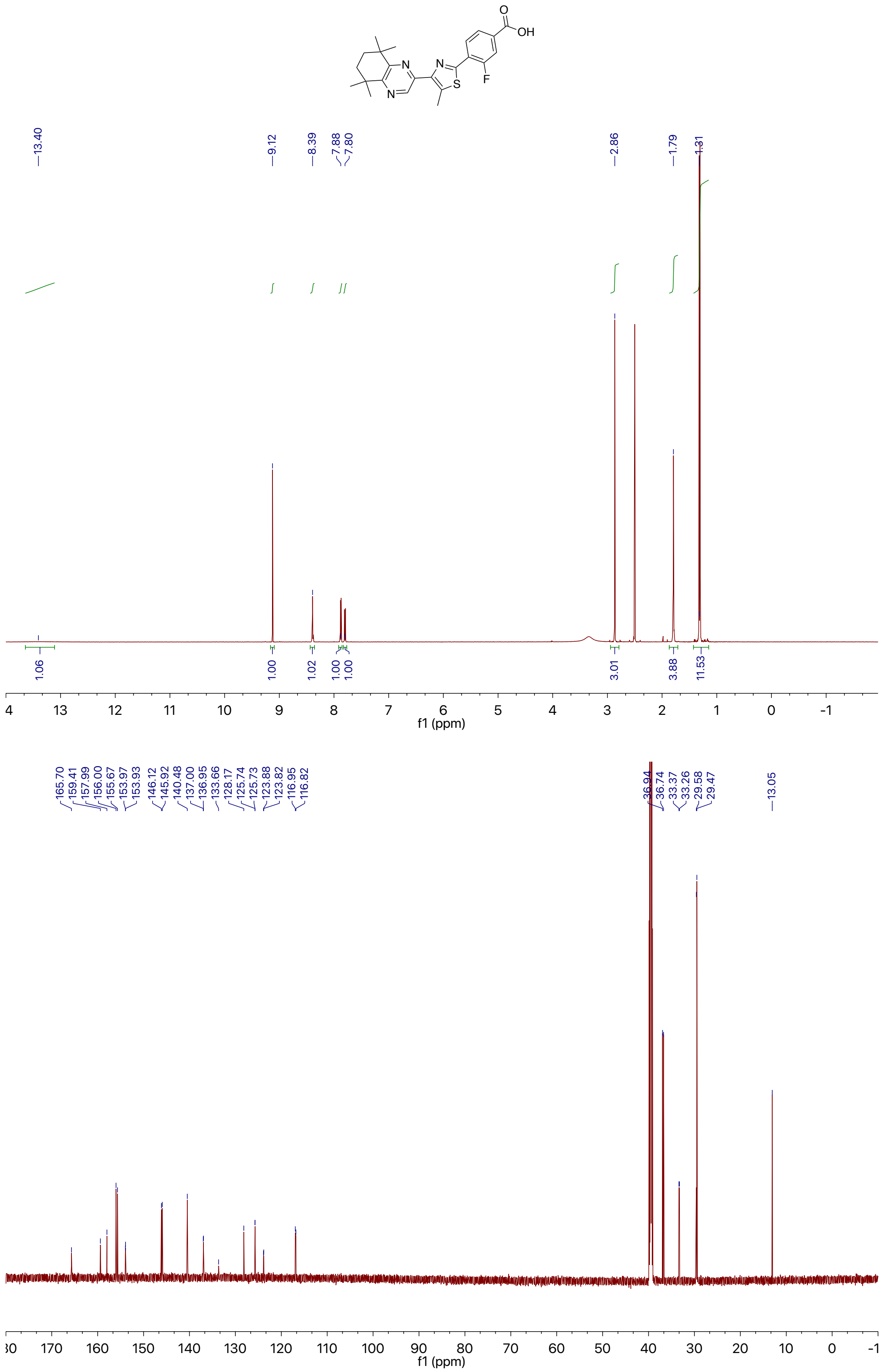

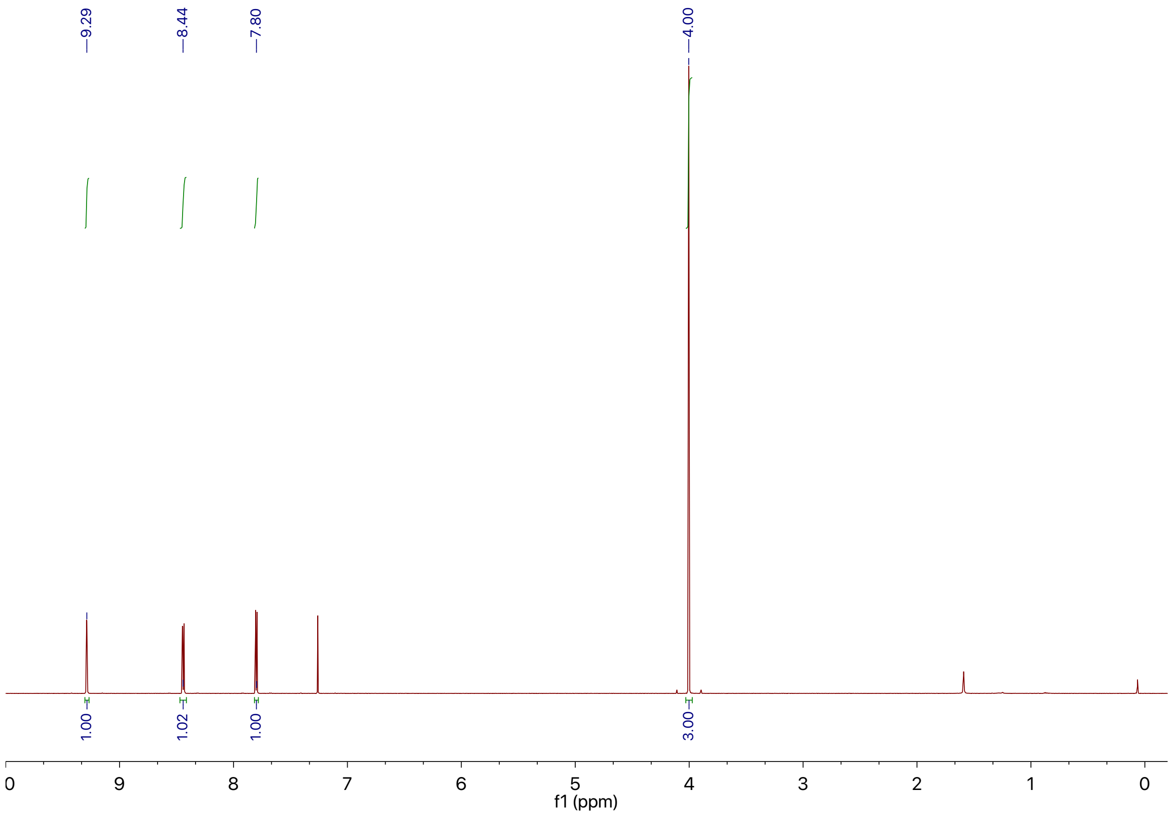
9.29
8.44
7.80
4.00
1.00
1.02
1.00
3.00
0
9
8
7
6
5
4
3
2
1
0
f1 (ppm)

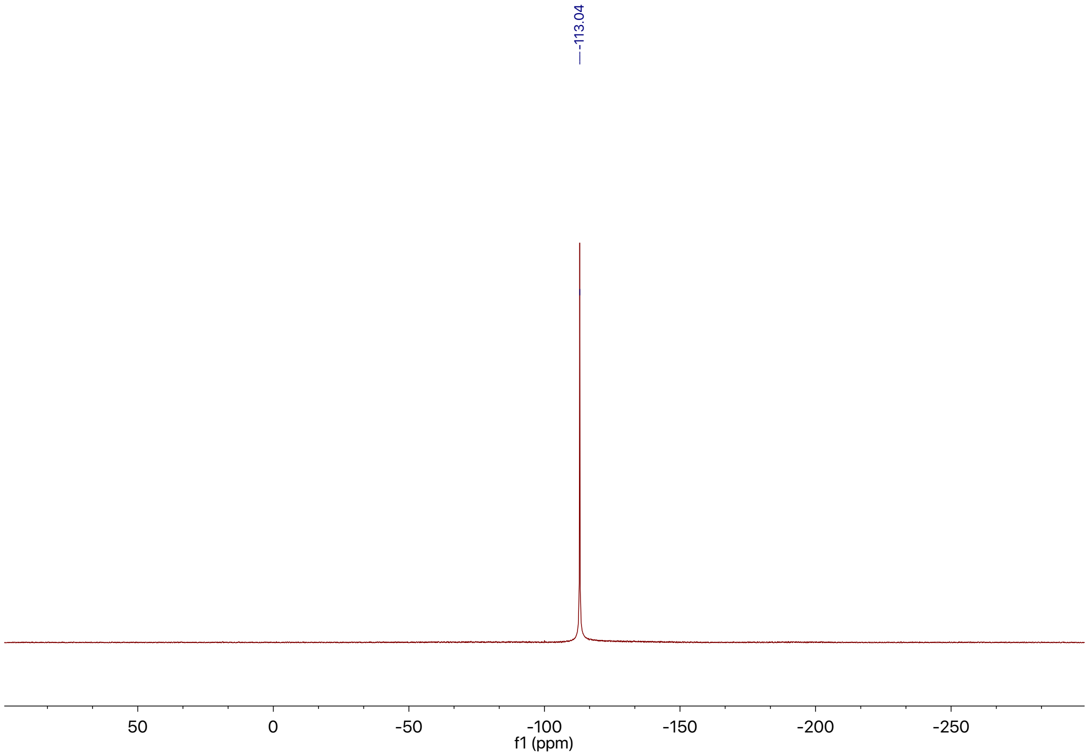
-113.04
50
0
-50
-100
-150
-200
-250
f1 (ppm)

Figure. S51. Chemical structure and NMR spectra of methyl 6-cyanopyridine-3-carboxylate

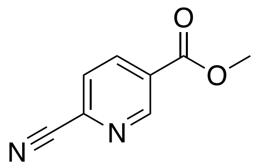

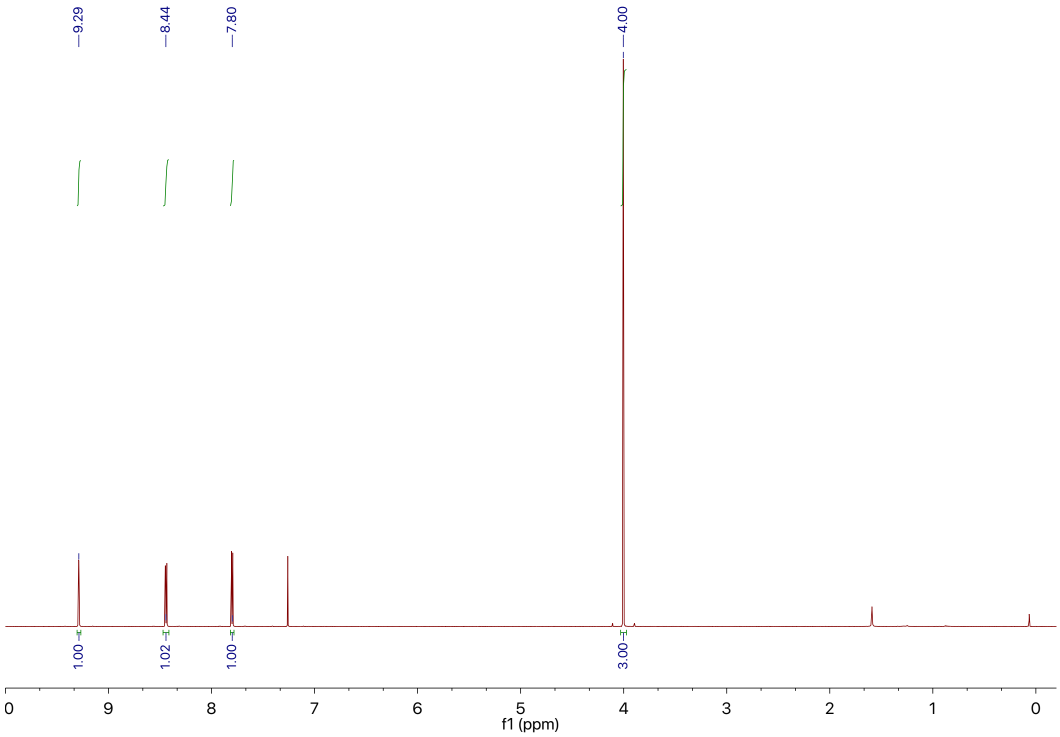

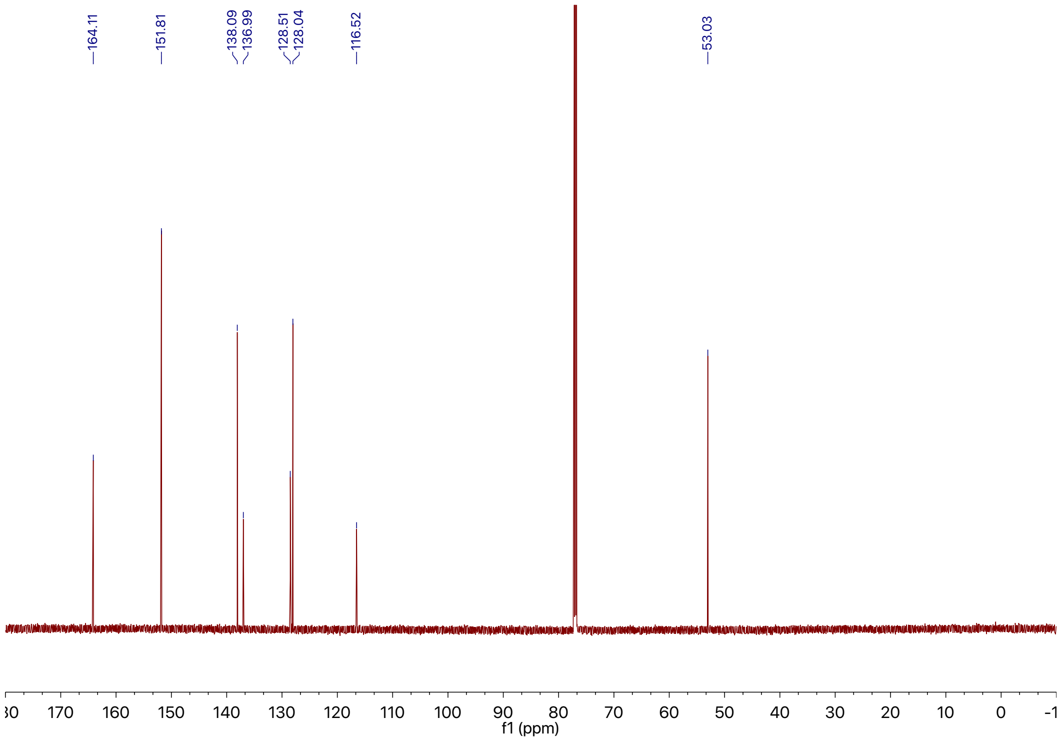

3. Fluorescence competition assays

Table S1. Binding affinities between synthetic retinoids and RARα and RARγ.

All values are reported to three significant figures. Binding affinities for compounds **20** and **21** were undeterminable using the assay method used. SD, standard deviation. Compounds **25**, **29** and **41** displayed non-competitive binding with DC271 towards either RARα and RARγ.

Compound	Binding K_D (± SD) / nM	
	RARα	RARγ
EC23	57.5 (± 7.06)	59.2 (± 9.46)
GZ25	688 (± 233)	335 (± 105)
13	13.6 (± 3.05)	18.6 (± 8.83)
14	30.9 (± 2.95)	16 (± 4.15)
15	0.37 (± 0.28)	22.7 (± 1.36)
16	73.7 (± 32.7)	24.2 (± 4.82)
17	28.4 (± 14.3)	20.6 (± 3.36)
18	18.1 (± 8.33)	18.6 (± 6.87)
19	5.72 (± 0.768)	33.7 (± 5.01)
20	Undeterminable	
21	Undeterminable	
22	105 (± 18)	355 (± 52.7)
23	15.8 (± 4.65)	24.3 (± 7.93)
24	87.1 (± 15.2)	254 (± 26.8)
25	927 (± 60.7)	Non-competitive Binding
26	48.3 (± 4.07)	90.5 (± 5.99)
27	1820 (± 367)	2150 (± 670)
28	420 (± 86.6)	750 (± 71.4)
29	Non-competitive Binding	
30	155 (± 14.2)	217 (± 19.1)
31	203 (± 44.5)	142 (± 37.3)
40	559 (± 208)	655 (± 114)
41	Non-competitive Binding	
42	162 (± 31.3)	133 (± 66.1)
43	2580 (± 647)	2710 (± 2080)

4. Molecular docking and dynamics simulations

Table S2. ChemScores and binding free energies between synthetic retinoids and RARα

All values are reported to three significant figures. ChemScore for compound **41** was undeterminable using the computational methods used. SD, standard deviation.

Compound	ChemScore	ΔG_{ESMACS} (± SD) / kcal·mol^{-1}
EC23	58.2	-56.1 (± 0.207)
GZ25	Undeterminable	-61.0 (± 0.199)
14	55.5	-59.1 (± 0.305)
15	54.8	-56.5 (± 0.294)
16	56.1	-60.0 (± 0.261)
17	58.8	-60.6 (± 0.212)
18	57.9	-57.5 (± 0.177)
19	55.6	-59.4 (± 0.167)
20	55.8	-58.9 (± 0.206)
21	56.0	-57.5 (± 0.155)
22	53.1	-59.4 (± 0.206)
23	56.7	-56.3 (± 0.216)
24	56.0	-52.7 (± 0.192)
25	54.6	-52.8 (± 0.247)
26	58.2	-55.1 (± 0.207)
27	55.7	-56.6 (± 0.241)
28	53.1	-56.5 (± 0.255)
29	49.7	-56.9 (± 0.379)
30	54.6	-54.4 (± 0.180)
31	53.4	-54.7 (± 0.111)
40	55.9	-56.7 (± 0.157)
41	Undeterminable	-53.3 (± 1.120)
42	53.6	-58.1 (± 0.324)
43	53.8	-56.5 (± 0.321)

Table S3. ChemScores and binding free energies between synthetic retinoids and RARβ

All values are reported to three significant figures. ChemScore for compound **41** was undeterminable using the computational methods used. SD, standard deviation.

Compound	ChemScore	ΔG_{ESMACS} (± SD) / kcal·mol^{-1}
EC23	56.2	-59.0 (± 0.339)
GZ25	57.0	-63.0 (± 0.210)
13	55.9	-59.6 (± 0.360)
14	55.3	-57.5 (± 0.142)
15	55.3	-53.9 (± 0.204)
16	57.6	-58.6 (± 0.327)
17	57.6	-60.5 (± 0.419)
18	57.8	-58.8 (± 0.203)
19	57.3	-60.4 (± 0.293)
20	57.3	-59.8 (± 0.243)
21	57.2	-59.2 (± 0.215)
22	55.3	-60.4 (± 0.147)
23	54.4	-55.6 (± 0.347)
24	54.4	-56.8 (± 0.213)
25	51.9	-52.1 (± 0.350)
26	56.1	-57.7 (± 0.322)
27	55.7	-56.2 (± 0.196)
28	51.6	-56.2 (± 0.597)
29	51.0	-54.7 (± 0.378)
30	52.6	-56.3 (± 0.271)
31	52.1	-53.8 (± 0.318)
40	55.2	-60.8 (± 0.184)
41	Undeterminable	-57.0 (± 1.370)
42	53.9	-61.9 (± 0.188)
43	54.3	-53.8 (± 0.318)

Table S4. ChemScores and binding free energies between synthetic retinoids and RARγ.

All values are reported to three significant figures. ChemScore for compound **41** was undeterminable using the computational methods used. SD, standard deviation.

Compound	ChemScore	ΔG_{ESMACS} (± SD) / kcal·mol^{-1}
EC23	58.2	-59.1 (± 0.359)
GZ25	60.0	-62.7 (± 0.229)
13	59.6	-59.2 (± 0.381)
14	57.5	-56.7 (± 0.455)
15	57.8	-53.1 (± 0.193)
16	59.1	-56.3 (± 0.343)
17	61.2	-60.5 (± 0.475)
18	60.3	-58.8 (± 0.222)
19	59.0	-60.4 (± 0.282)
20	61.5	-60.2 (± 0.431)
21	60.0	-58.0 (± 0.307)
22	55.1	-60.7 (± 0.287)
23	57.4	-56.3 (± 0.458)
24	58.0	-55.8 (± 0.770)
25	55.7	-50.7 (± 0.551)
26	58.4	-56.9 (± 0.436)
27	60.1	-57.3 (± 0.445)
28	54.8	-56.9 (± 0.640)
29	54.5	-56.0 (± 0.515)
30	56.4	-56.1 (± 0.588)
31	54.8	-53.6 (± 0.219)
40	53.8	-60.1 (± 0.172)
41	Undeterminable	-55.9 (± 1.400)
42	54.4	-62.1 (± 0.239)
43	53.3	-53.6 (± 0.219)

5. Multiple sequence alignments

Figure S50. Multiple sequence alignment of human Retinoic Acid Receptors, RARα$^{182-417}$, RARβ$^{182-417}$ and RARγ$^{182-417}$

Identical residues across the RAR isoforms are highlight in black, bold text. RAR isoform-specific residues (Figure. 7) are highlighted as black boxes with white text. RARα secondary structure between residues 182-417 shown as α-helices (α1-12) and β-sheets (black arrows, β1-2). Figure produced using ESPript 3.0 (2).

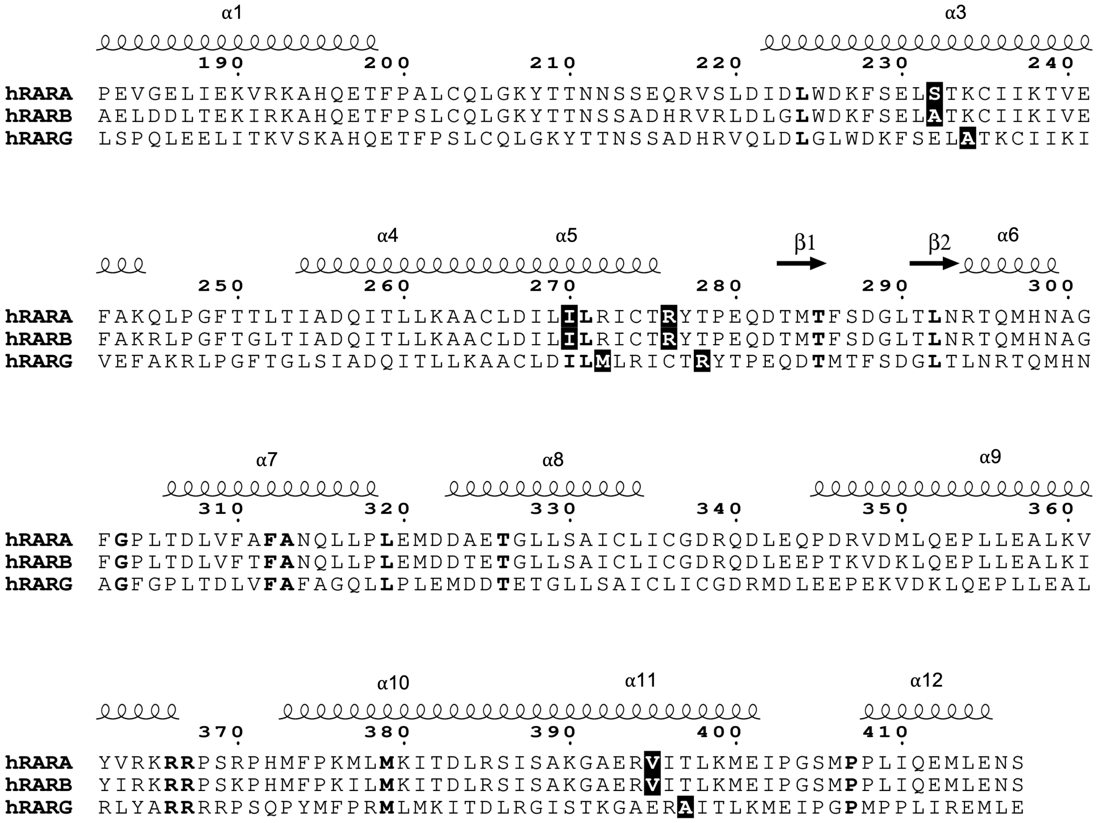

7. References

(1) Fife, W. K. Regioselective Cyanation of Pyridine N-Oxides with Trimethylsilanecarbonitrile: A Modified Reissert-Henze Reaction. *J. Org. Chem.* **1983**, *48*, 1375–1377.

(2) Robert, X., Gouet, P., Deciphering key features in protein structures with the new ENDscript server. *Nucleic Acids Research*. **2014,** *42*, *W320–W324*.
